# Supplementary figures and images for: Knowledge enhanced bottom-up affordance grounding for robotic interaction (part 2 of 2)
Source: PeerJ Comput Sci. 2024 Jul 5;10:e2097. doi: 10.7717/peerj-cs.2097 (PMC11232630; doi:10.7717/peerj-cs.2097)

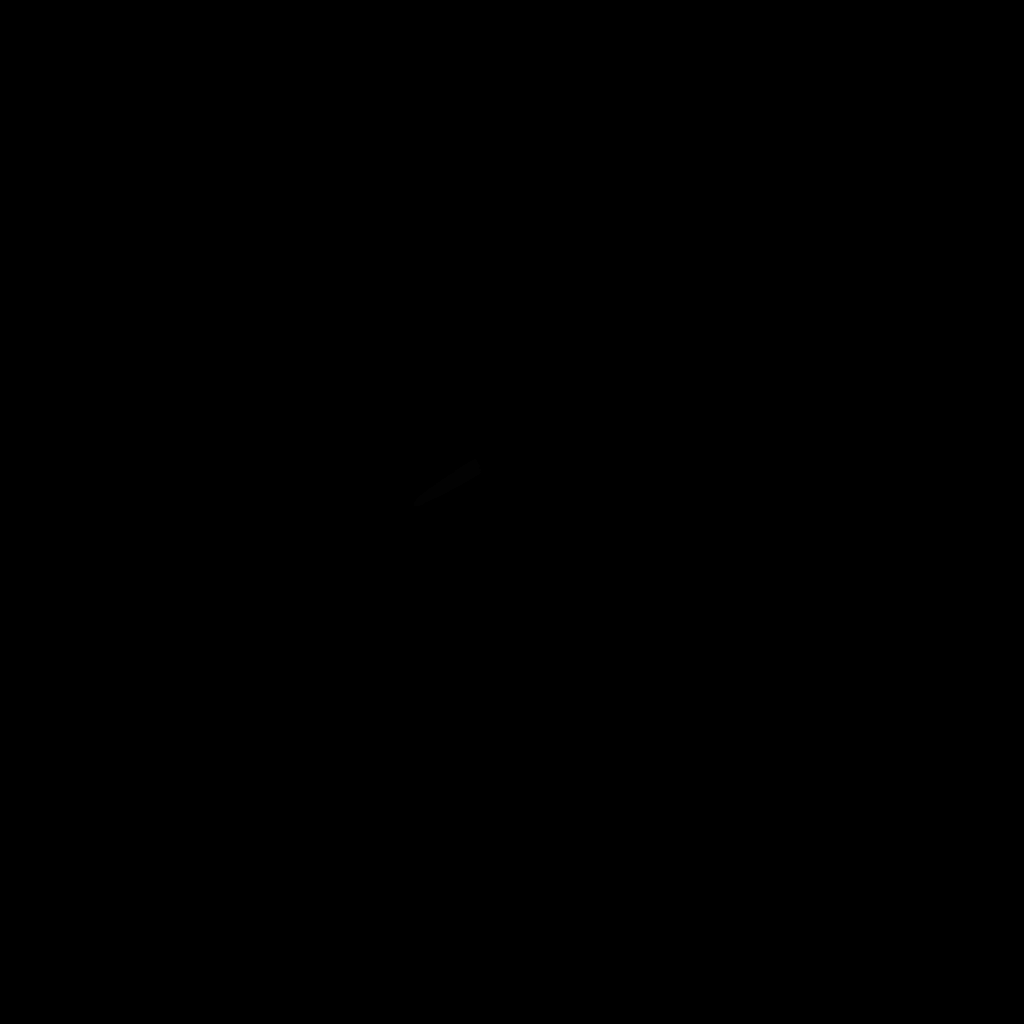

Supplement: Supplemental Information 1 [file peerj-cs-10-2097-s001.zip › IIT-AFF VL/masks/03_00000192.png]

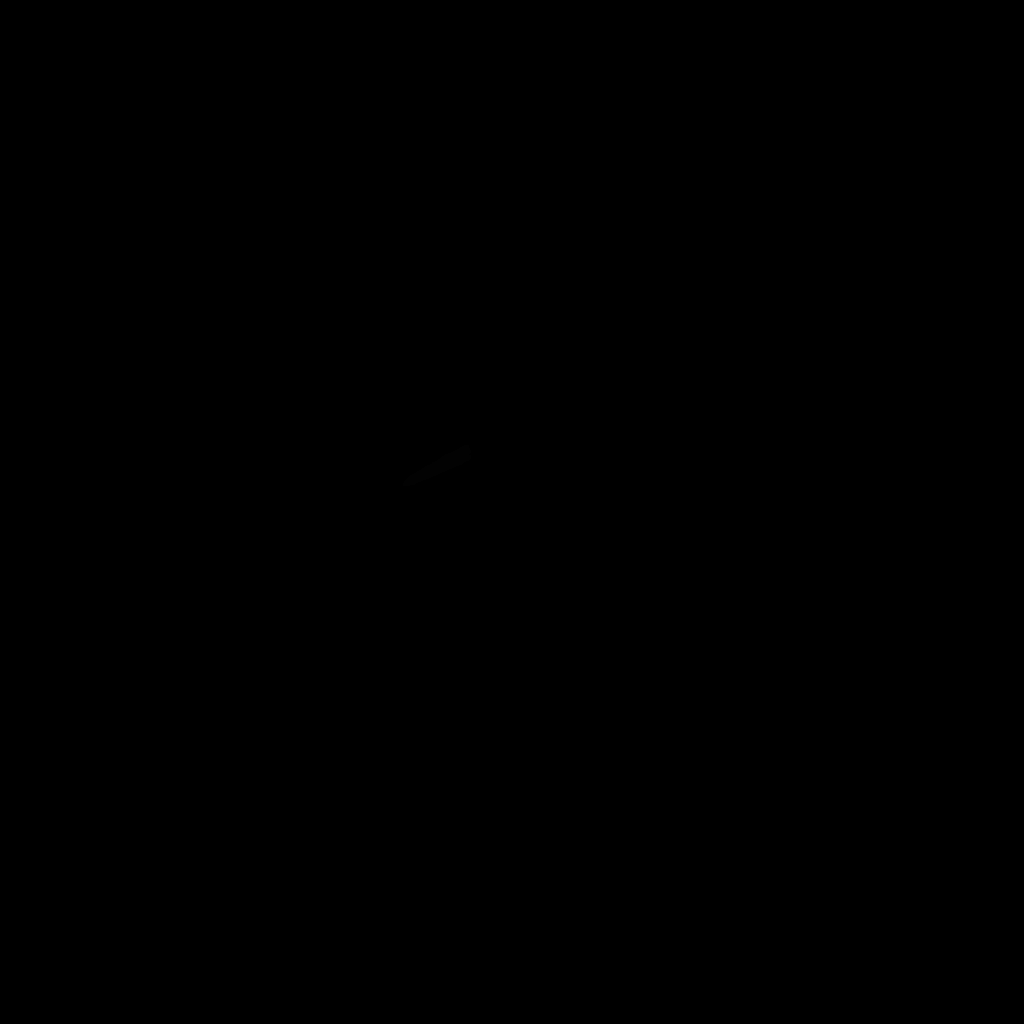

Supplement: Supplemental Information 1 [file peerj-cs-10-2097-s001.zip › IIT-AFF VL/masks/03_00000221.png]

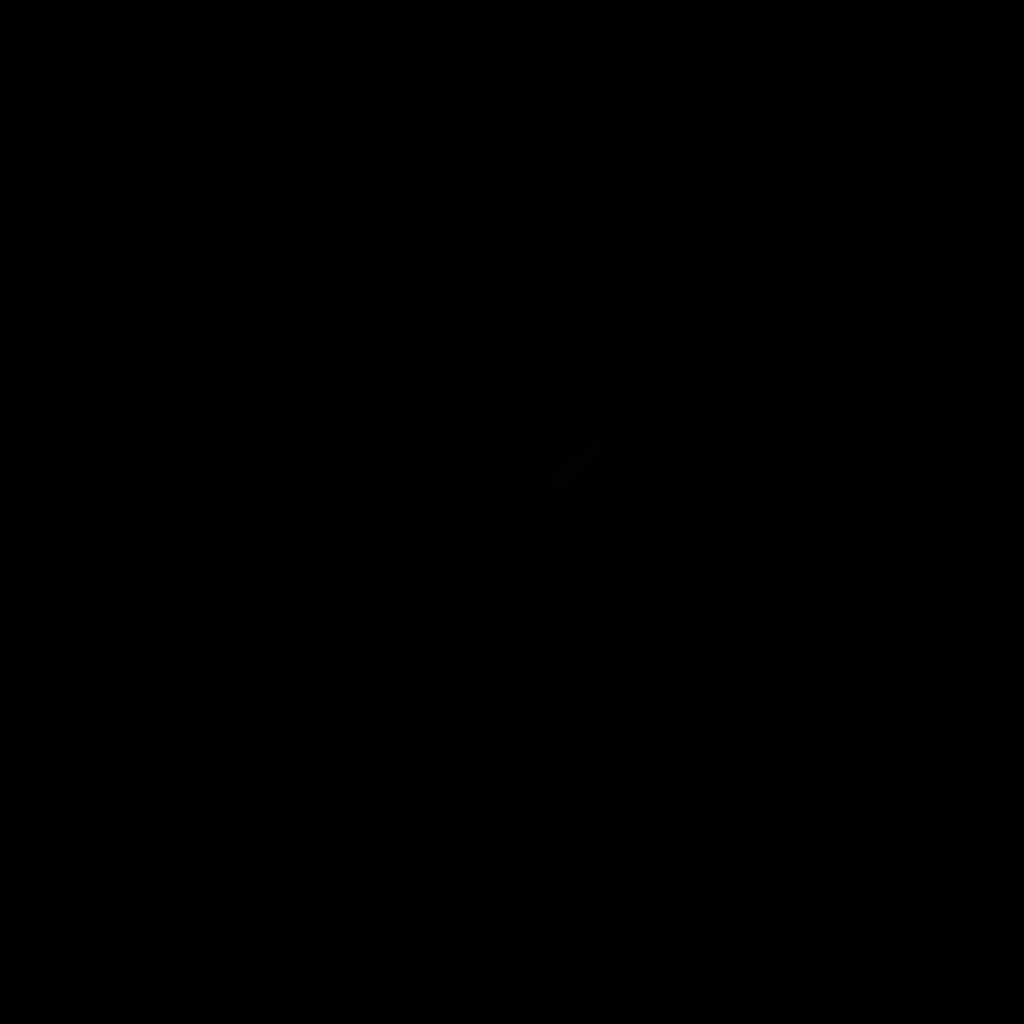

Supplement: Supplemental Information 1 [file peerj-cs-10-2097-s001.zip › IIT-AFF VL/masks/03_00000256.png]

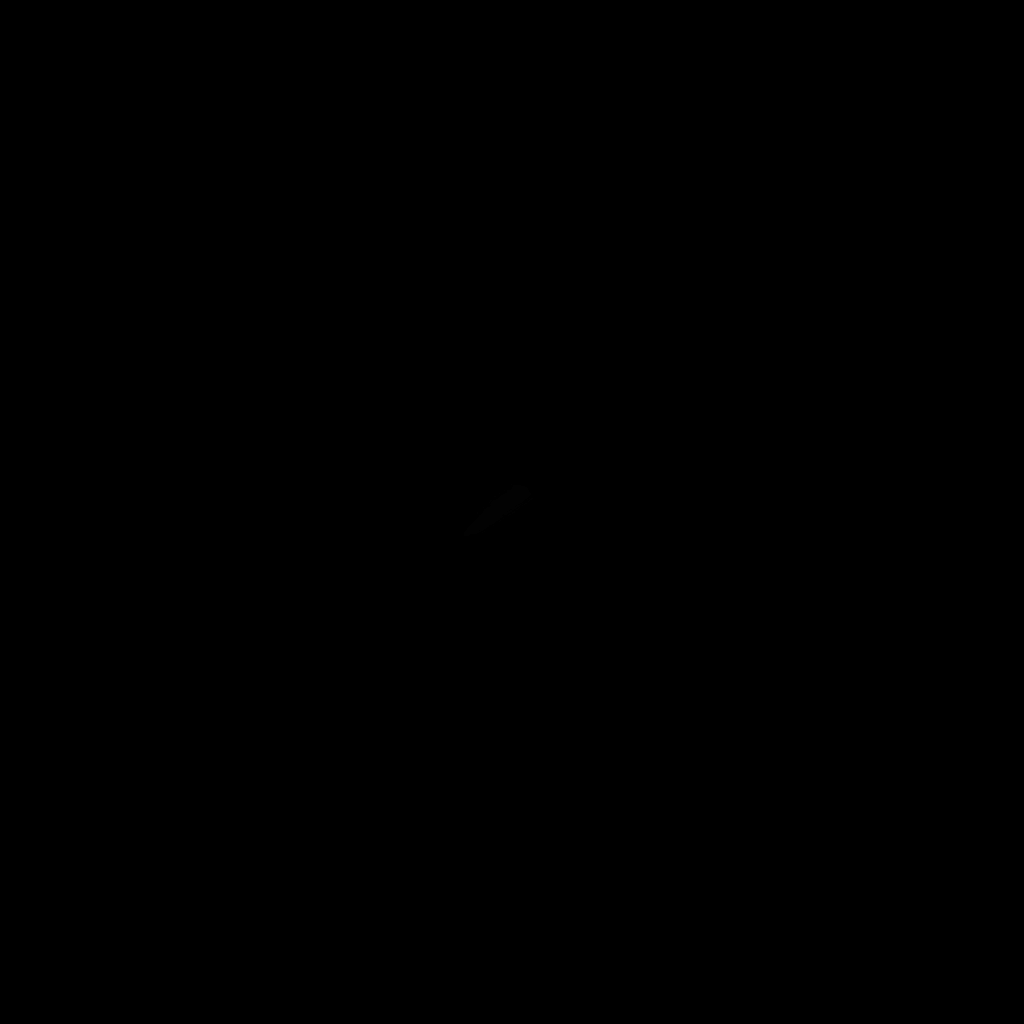

Supplement: Supplemental Information 1 [file peerj-cs-10-2097-s001.zip › IIT-AFF VL/masks/03_00000282.png]

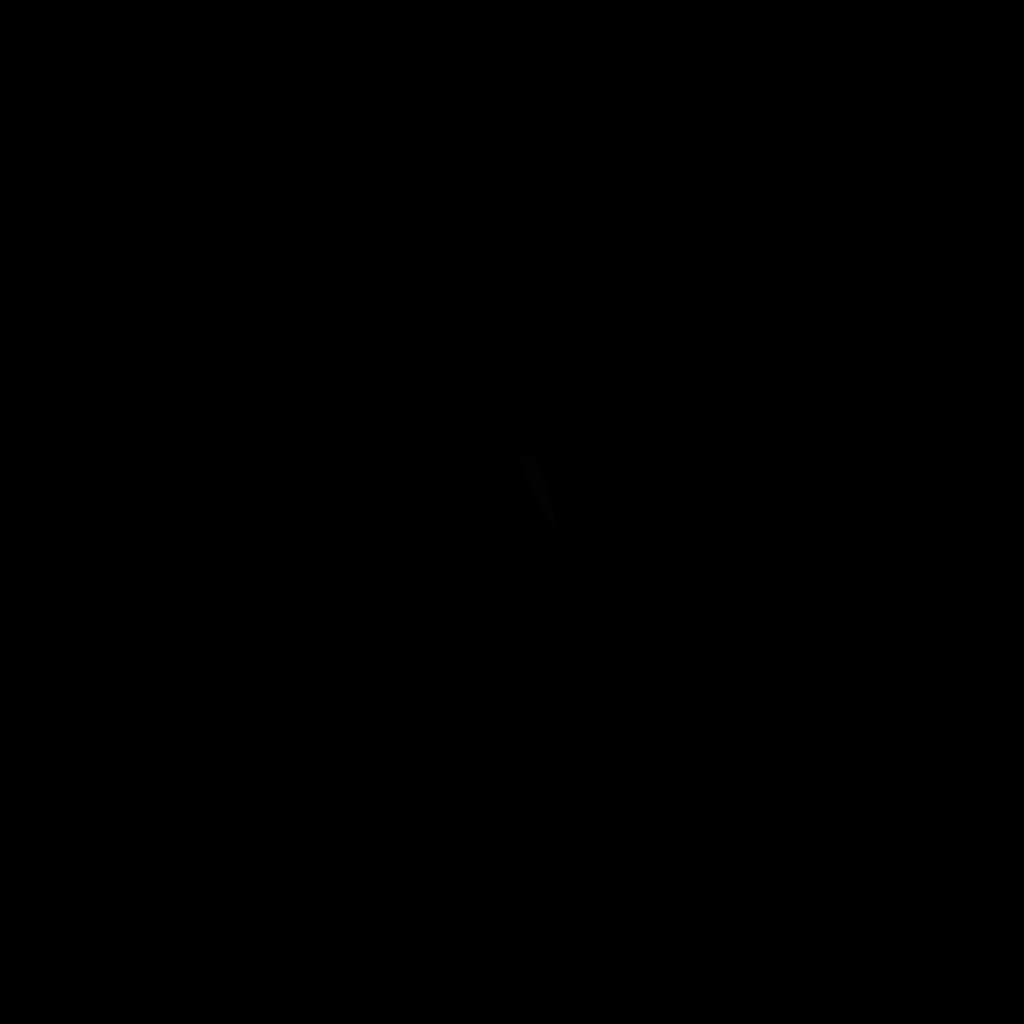

Supplement: Supplemental Information 1 [file peerj-cs-10-2097-s001.zip › IIT-AFF VL/masks/03_00000302.png]

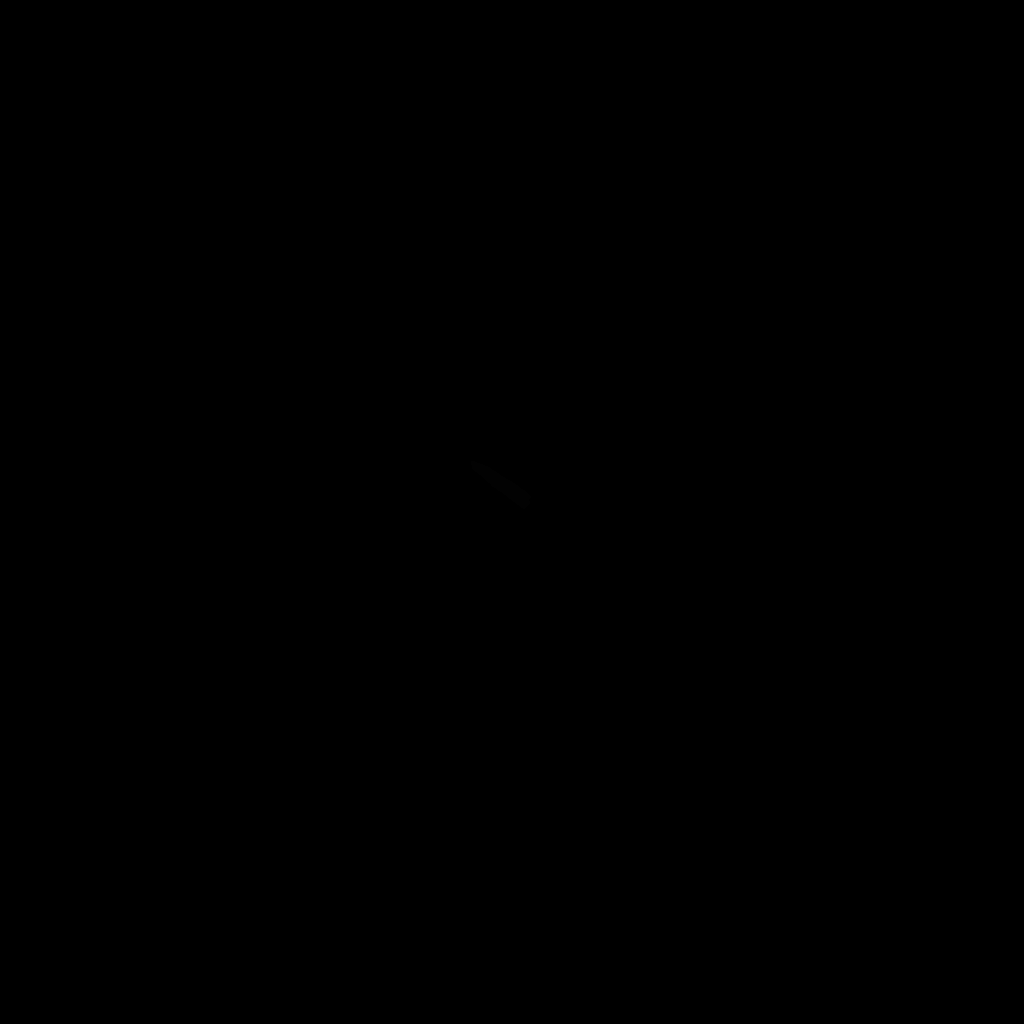

Supplement: Supplemental Information 1 [file peerj-cs-10-2097-s001.zip › IIT-AFF VL/masks/03_00000326.png]

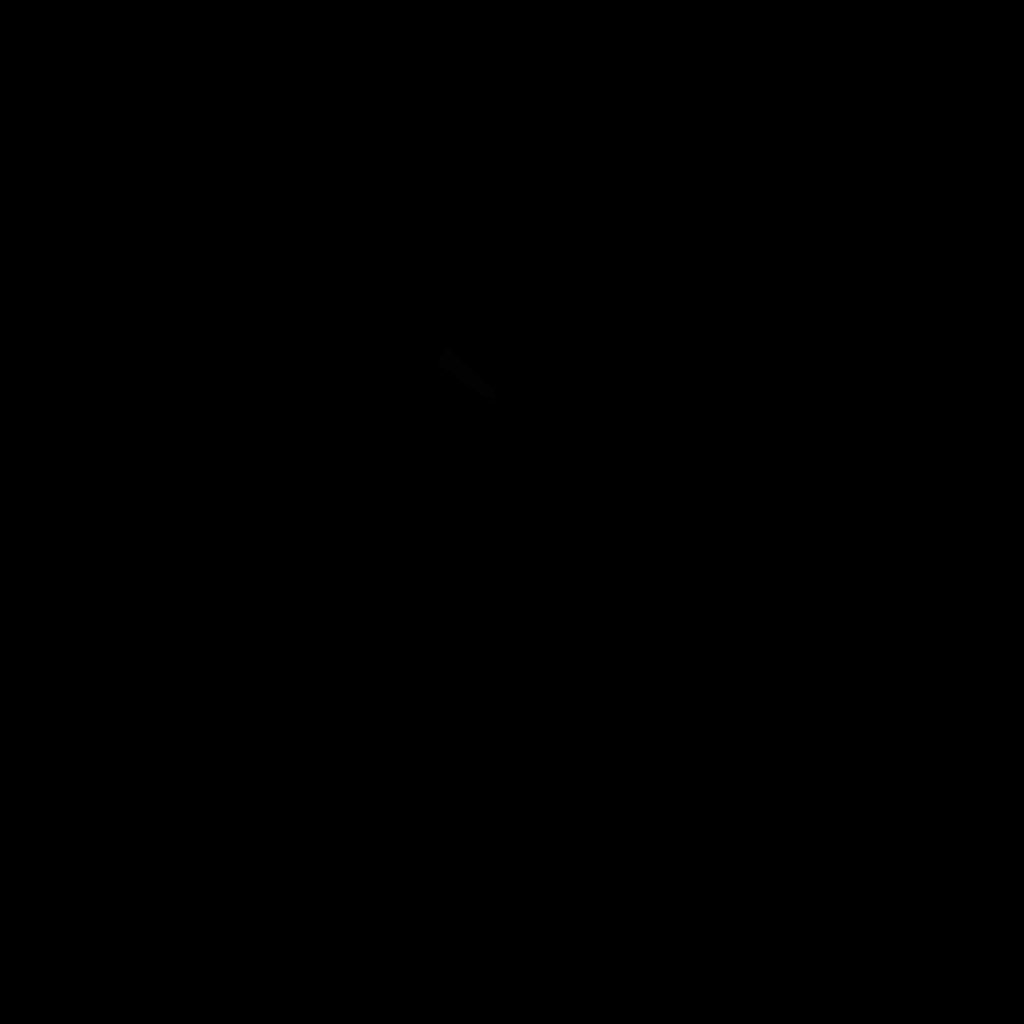

Supplement: Supplemental Information 1 [file peerj-cs-10-2097-s001.zip › IIT-AFF VL/masks/03_00000351.png]

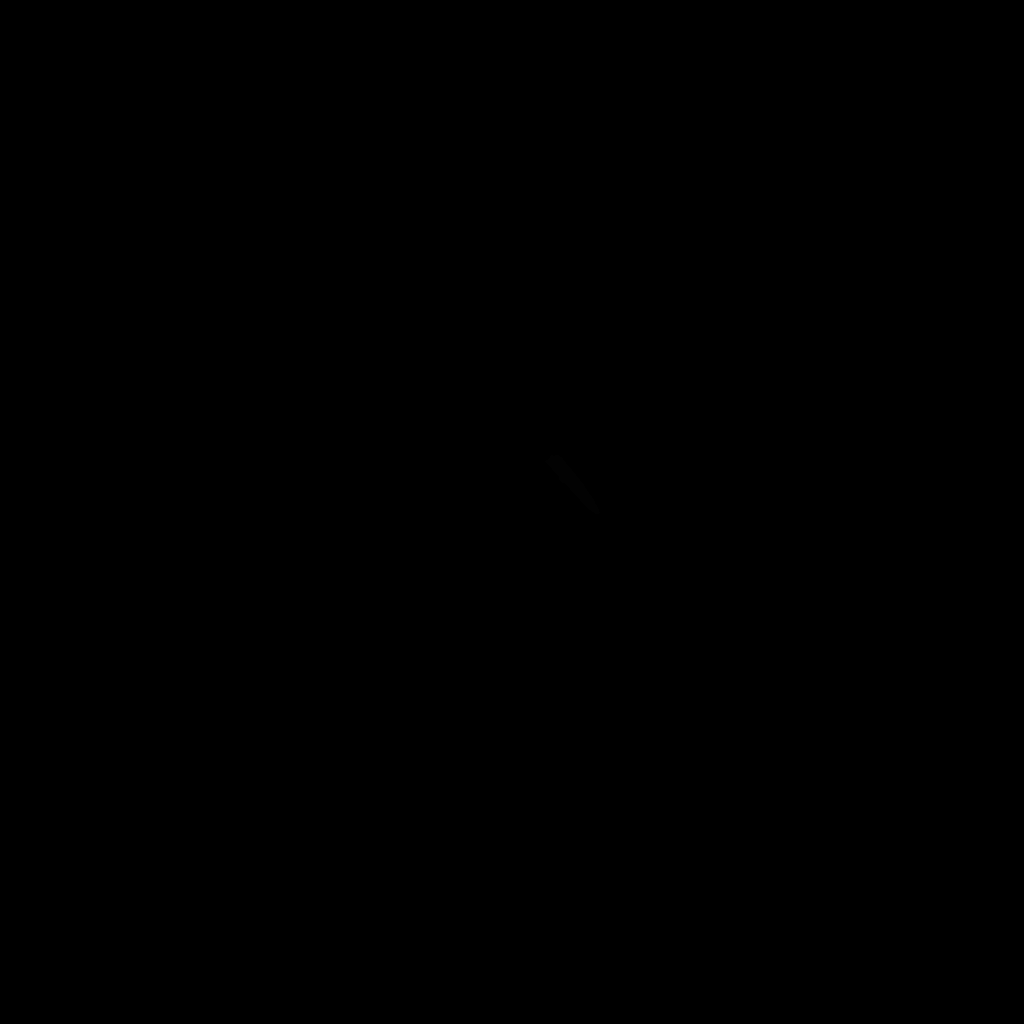

Supplement: Supplemental Information 1 [file peerj-cs-10-2097-s001.zip › IIT-AFF VL/masks/03_00000374.png]

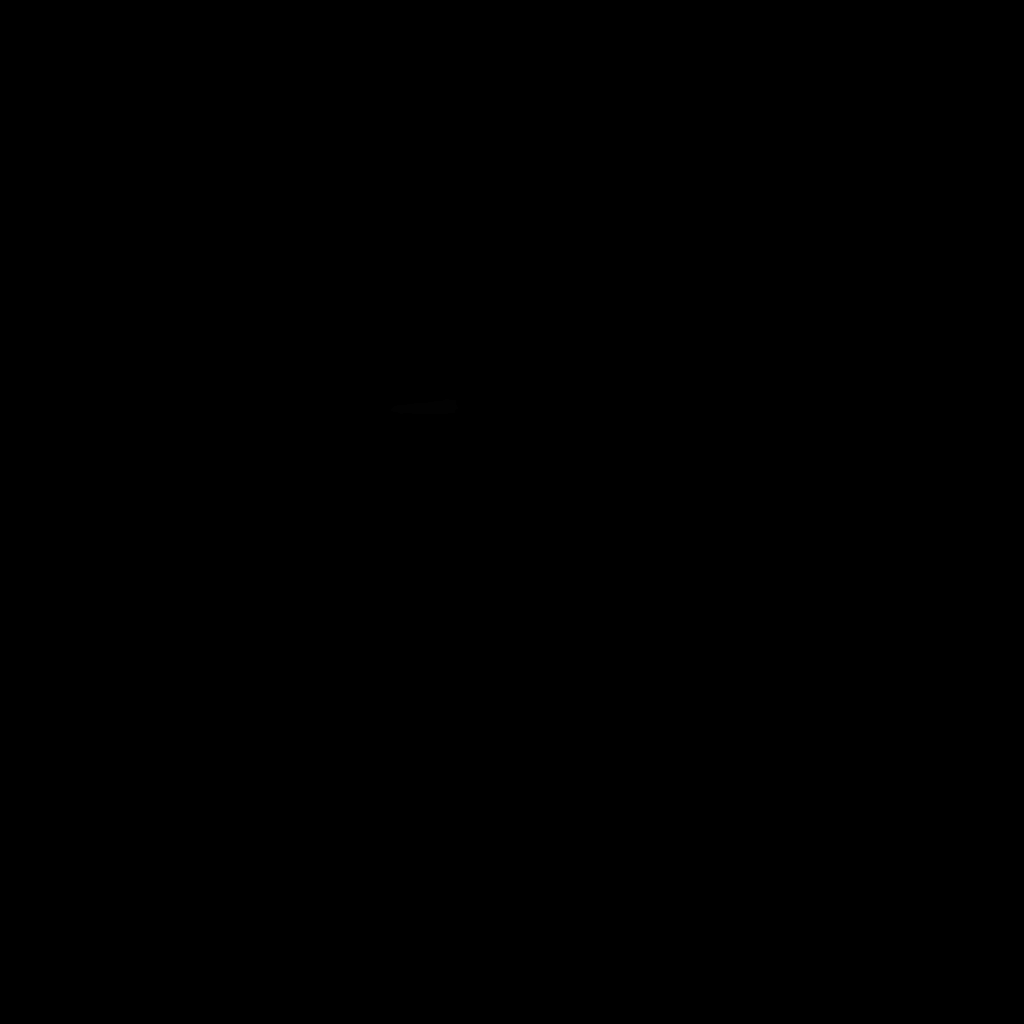

Supplement: Supplemental Information 1 [file peerj-cs-10-2097-s001.zip › IIT-AFF VL/masks/03_00000403.png]

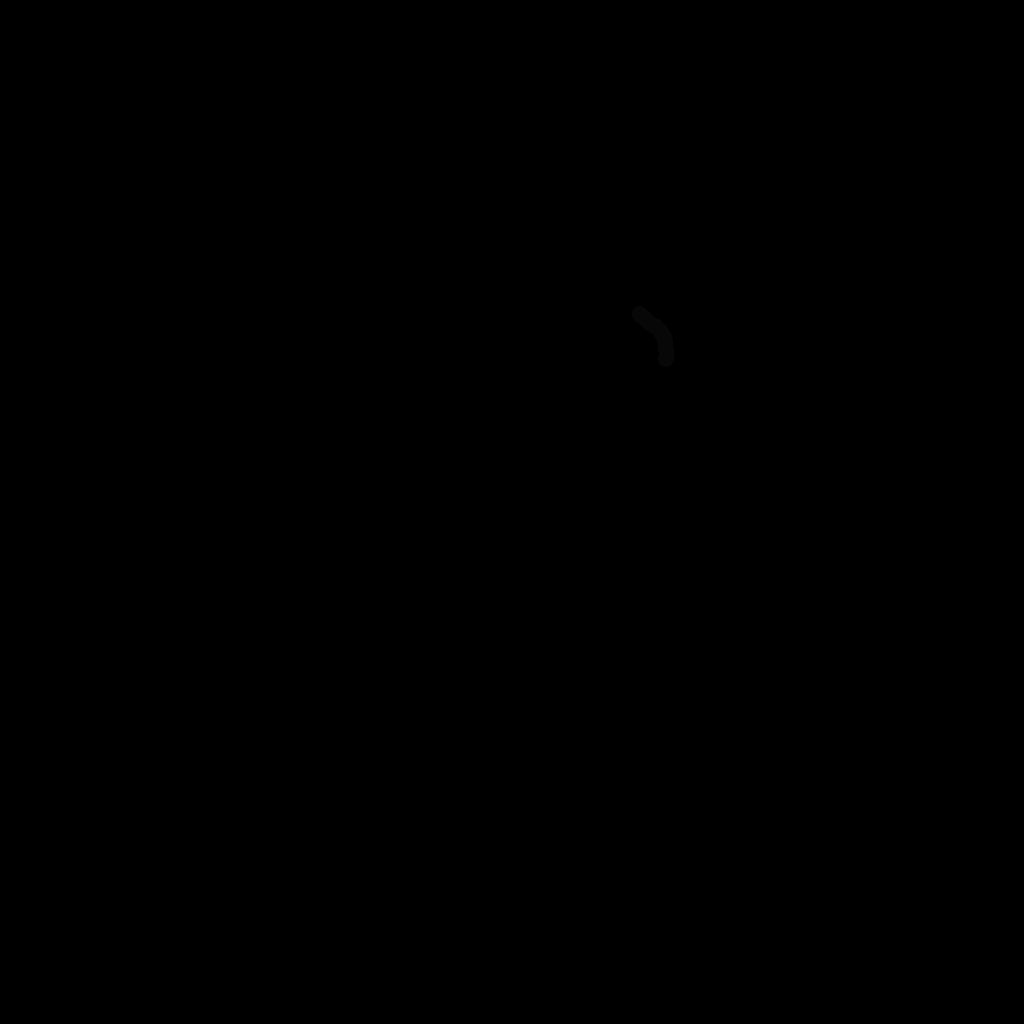

Supplement: Supplemental Information 1 [file peerj-cs-10-2097-s001.zip › IIT-AFF VL/masks/03_00000427.png]

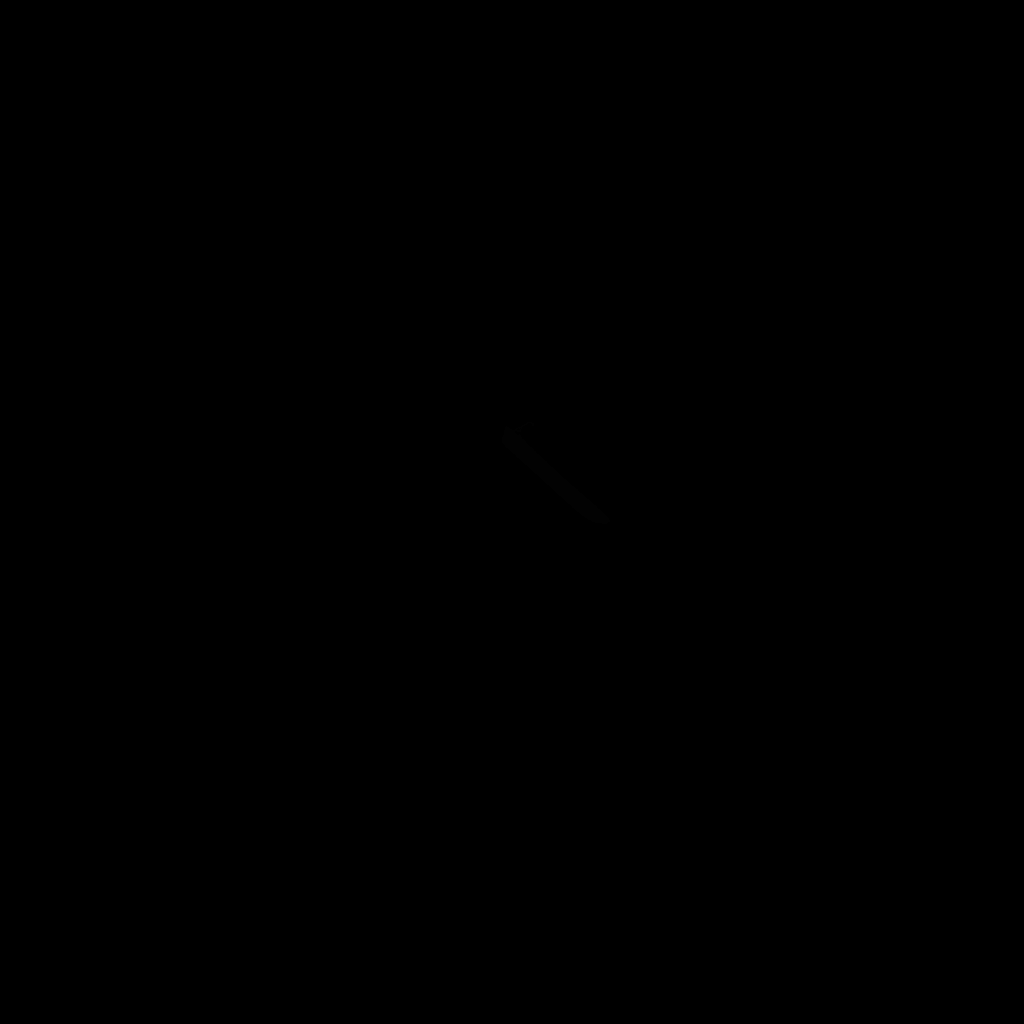

Supplement: Supplemental Information 1 [file peerj-cs-10-2097-s001.zip › IIT-AFF VL/masks/03_00000446.png]

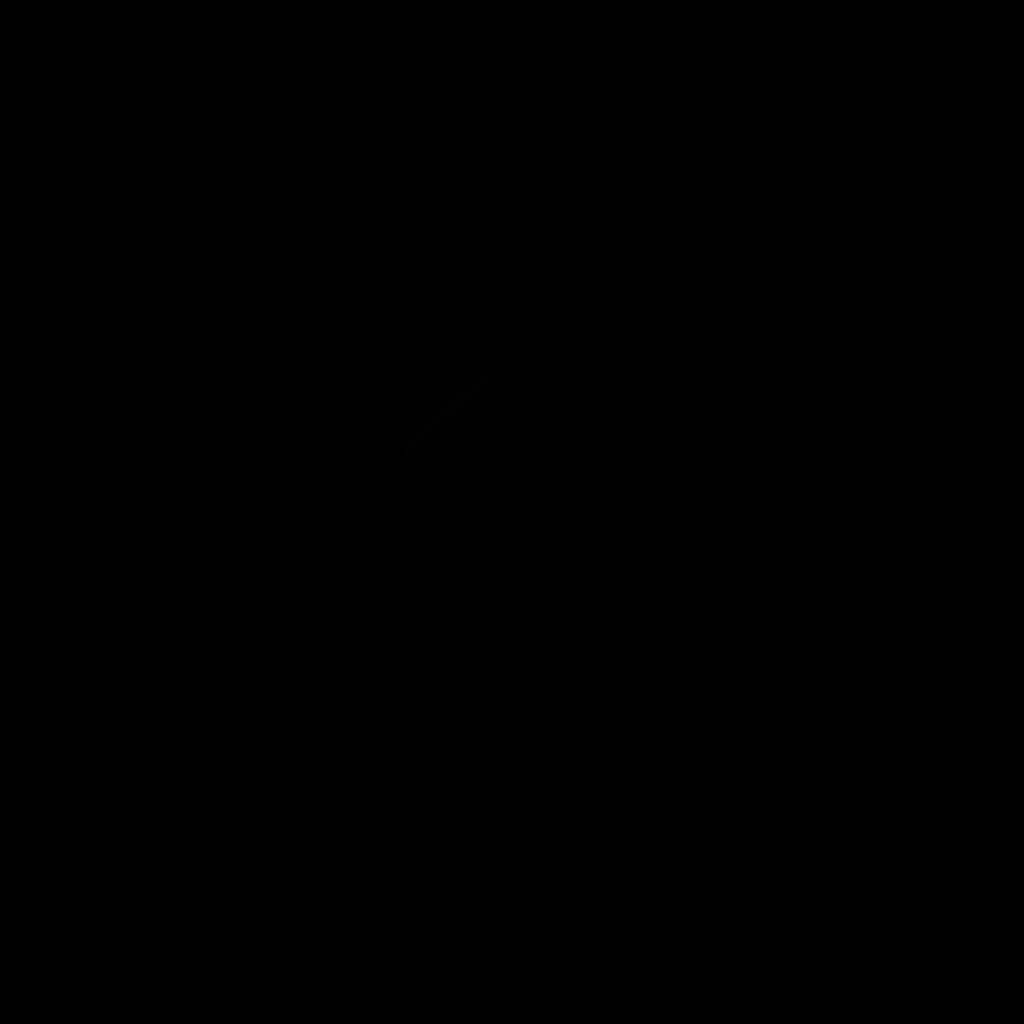

Supplement: Supplemental Information 1 [file peerj-cs-10-2097-s001.zip › IIT-AFF VL/masks/03_00000466.png]

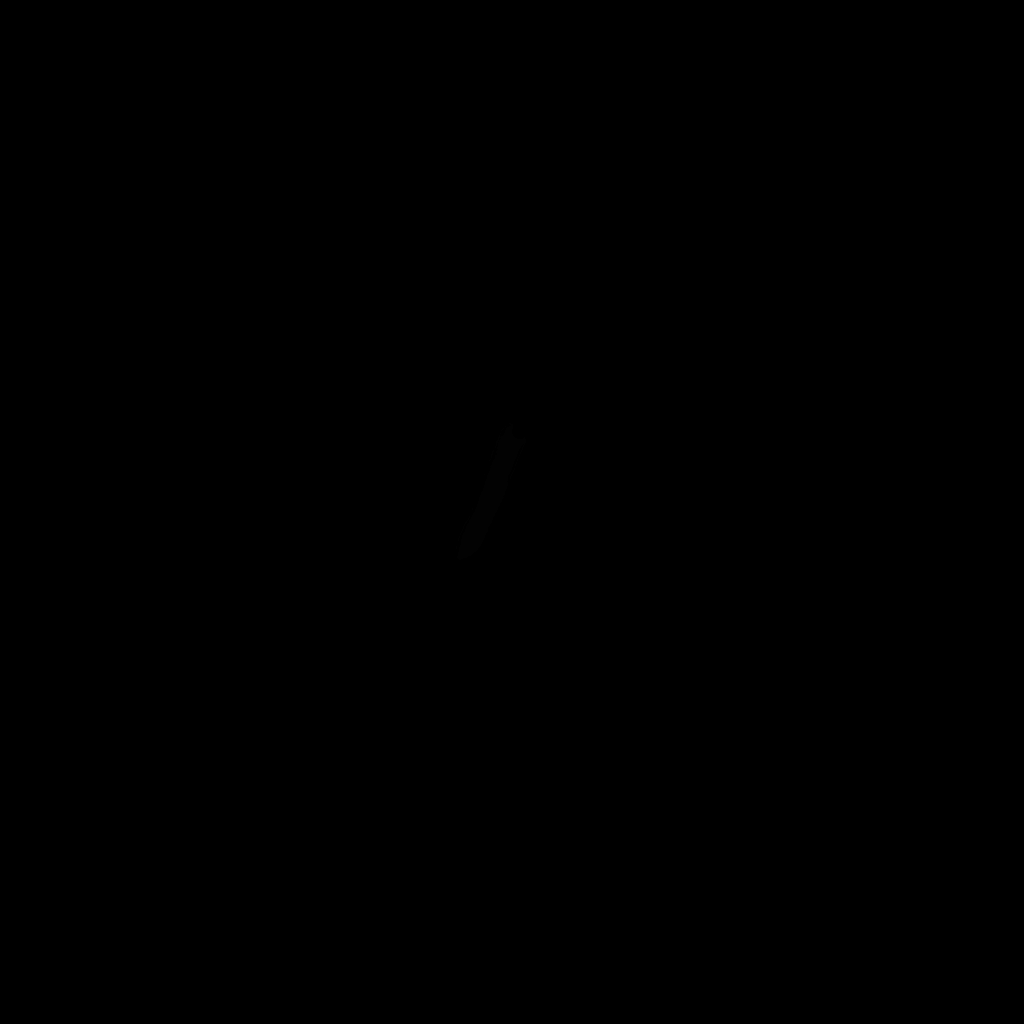

Supplement: Supplemental Information 1 [file peerj-cs-10-2097-s001.zip › IIT-AFF VL/masks/03_00000483.png]

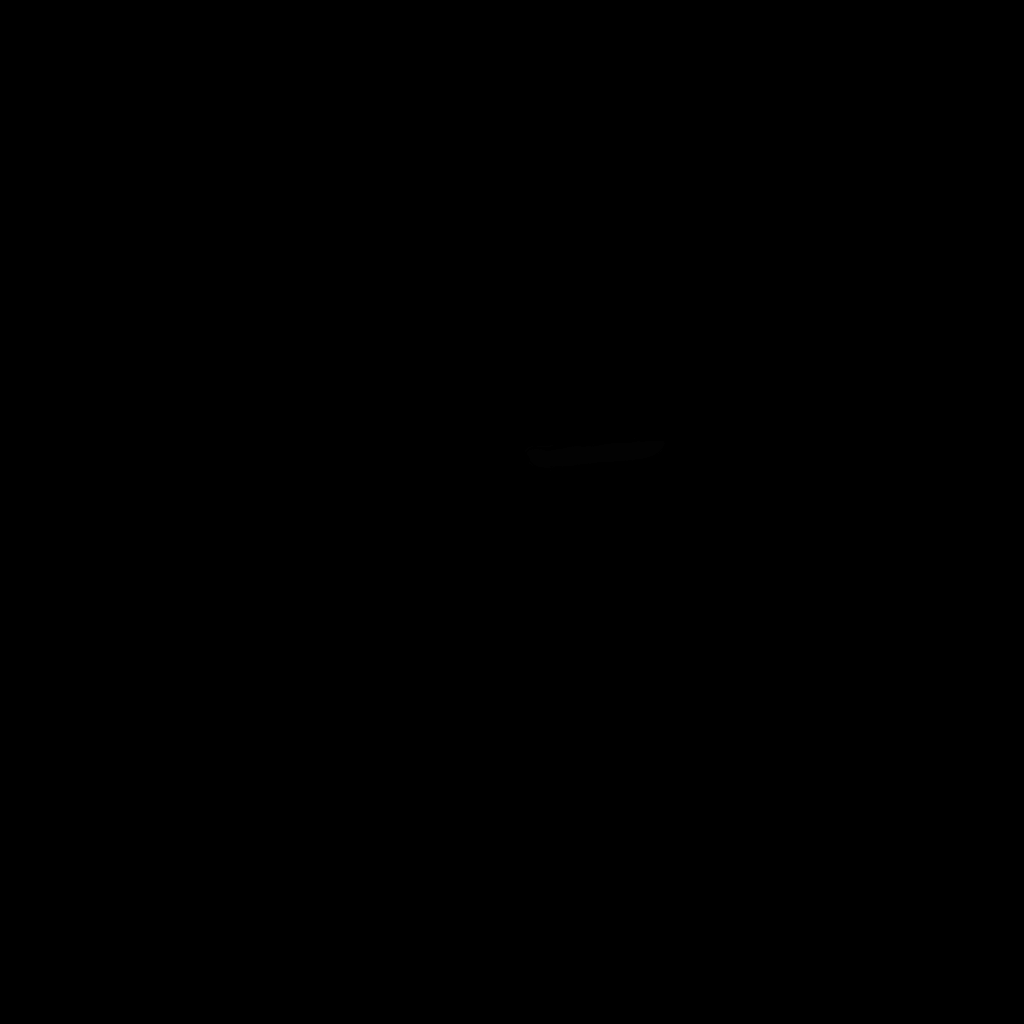

Supplement: Supplemental Information 1 [file peerj-cs-10-2097-s001.zip › IIT-AFF VL/masks/03_00000509.png]

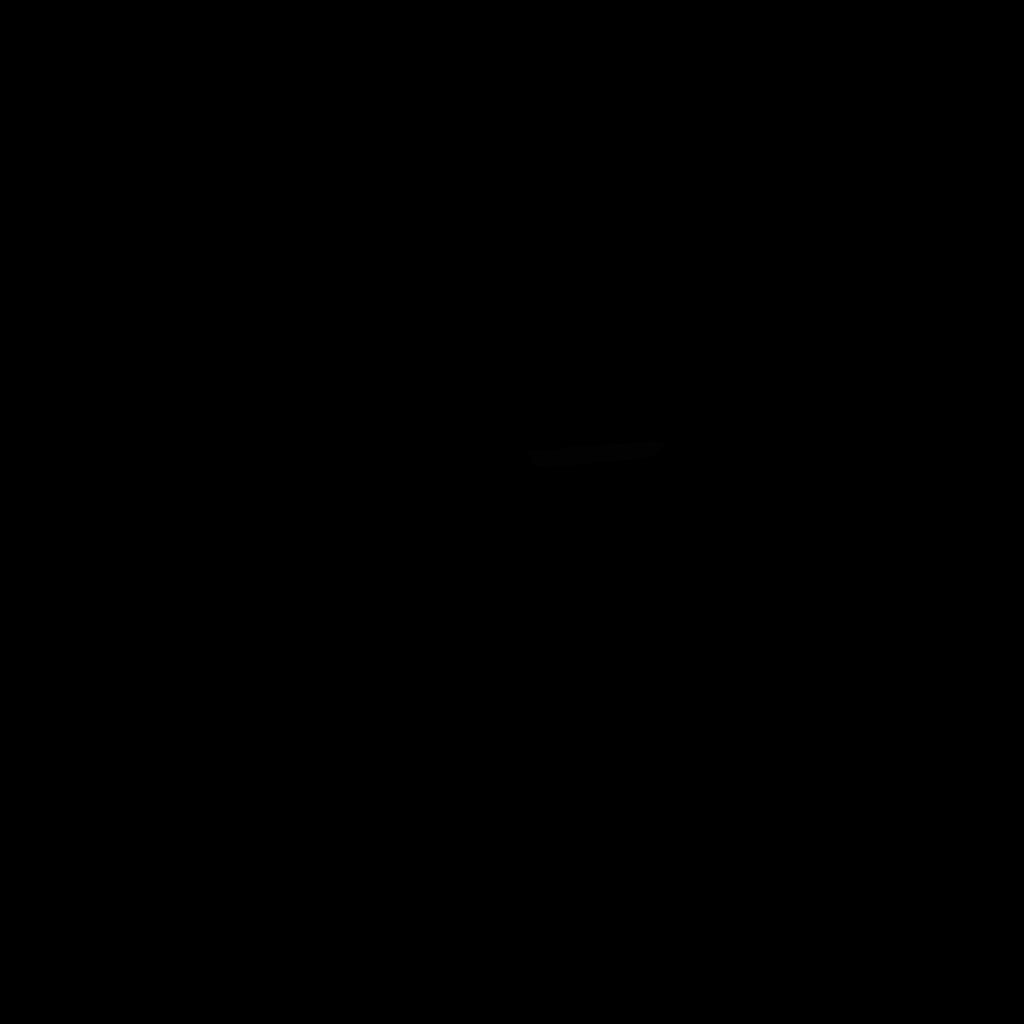

Supplement: Supplemental Information 1 [file peerj-cs-10-2097-s001.zip › IIT-AFF VL/masks/03_00000517.png]

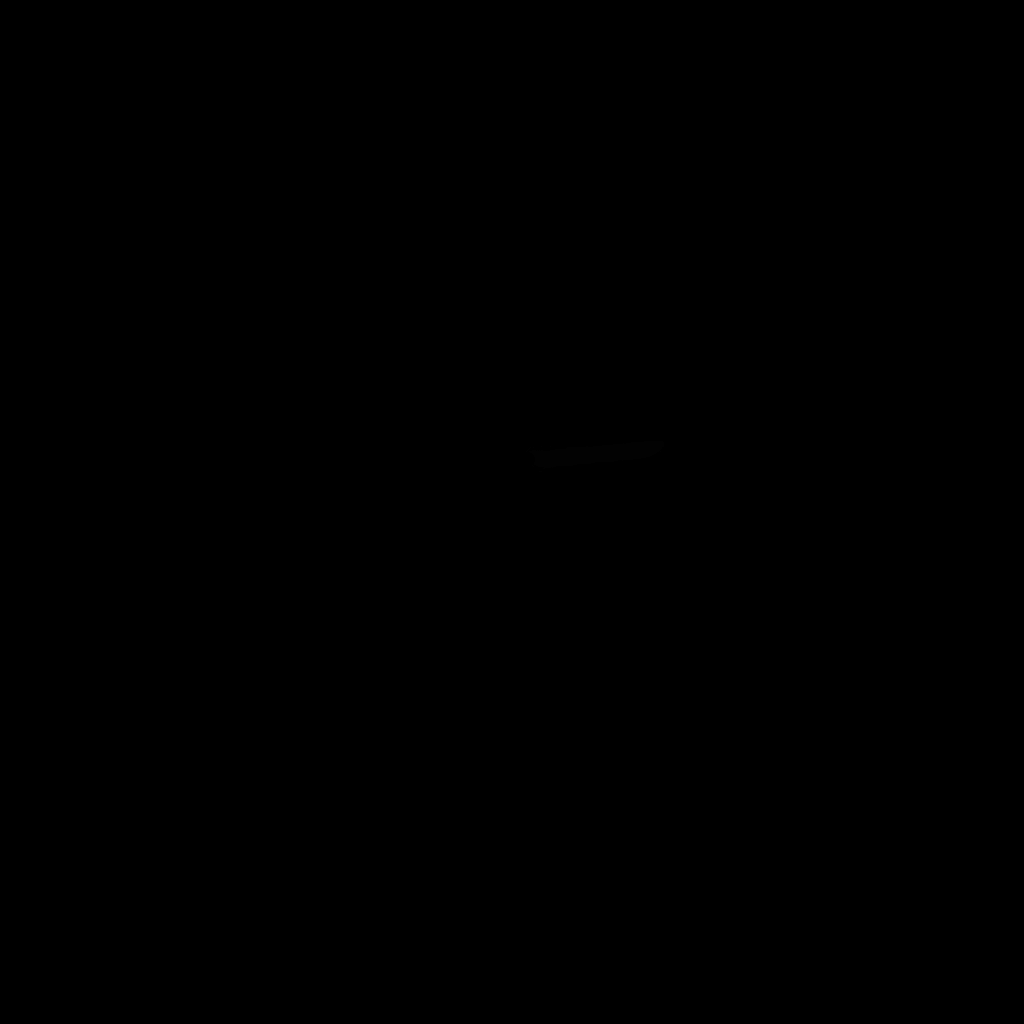

Supplement: Supplemental Information 1 [file peerj-cs-10-2097-s001.zip › IIT-AFF VL/masks/03_00000542.png]

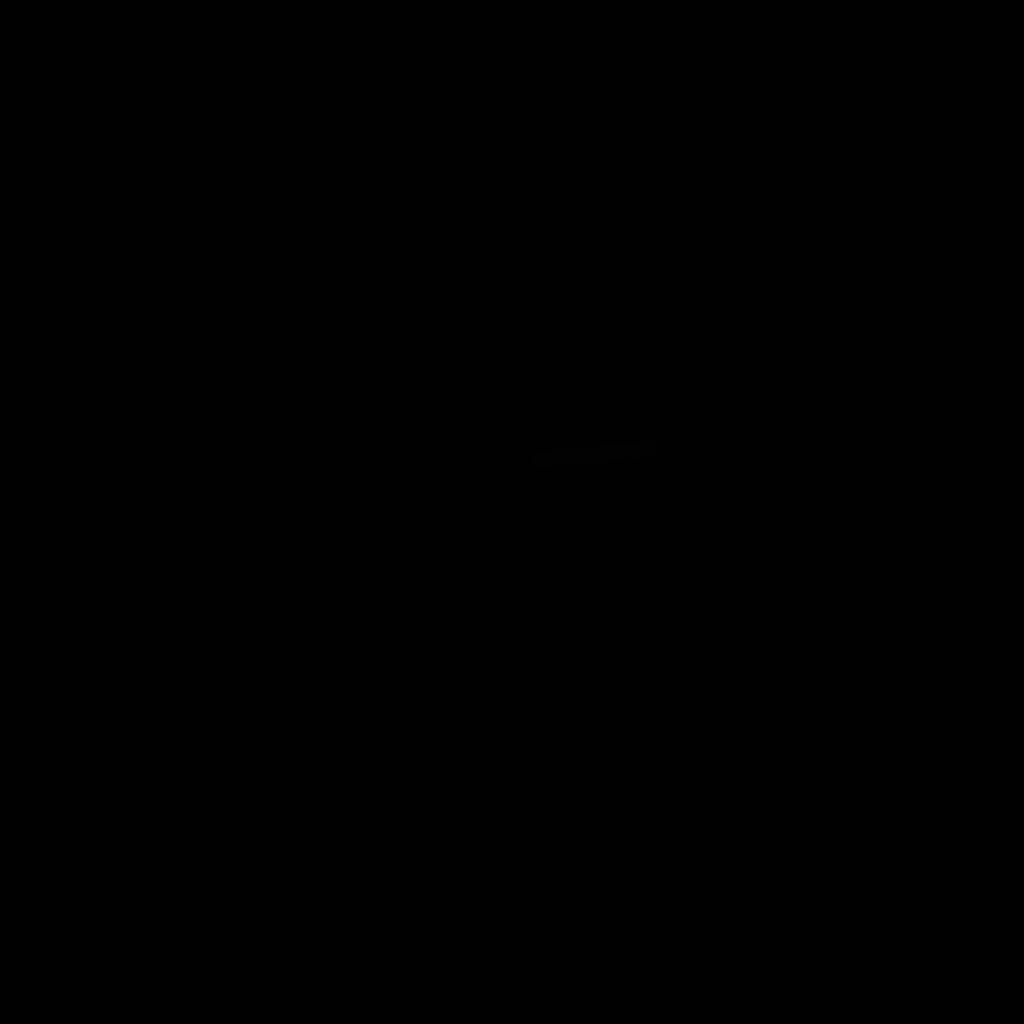

Supplement: Supplemental Information 1 [file peerj-cs-10-2097-s001.zip › IIT-AFF VL/masks/03_00000557.png]

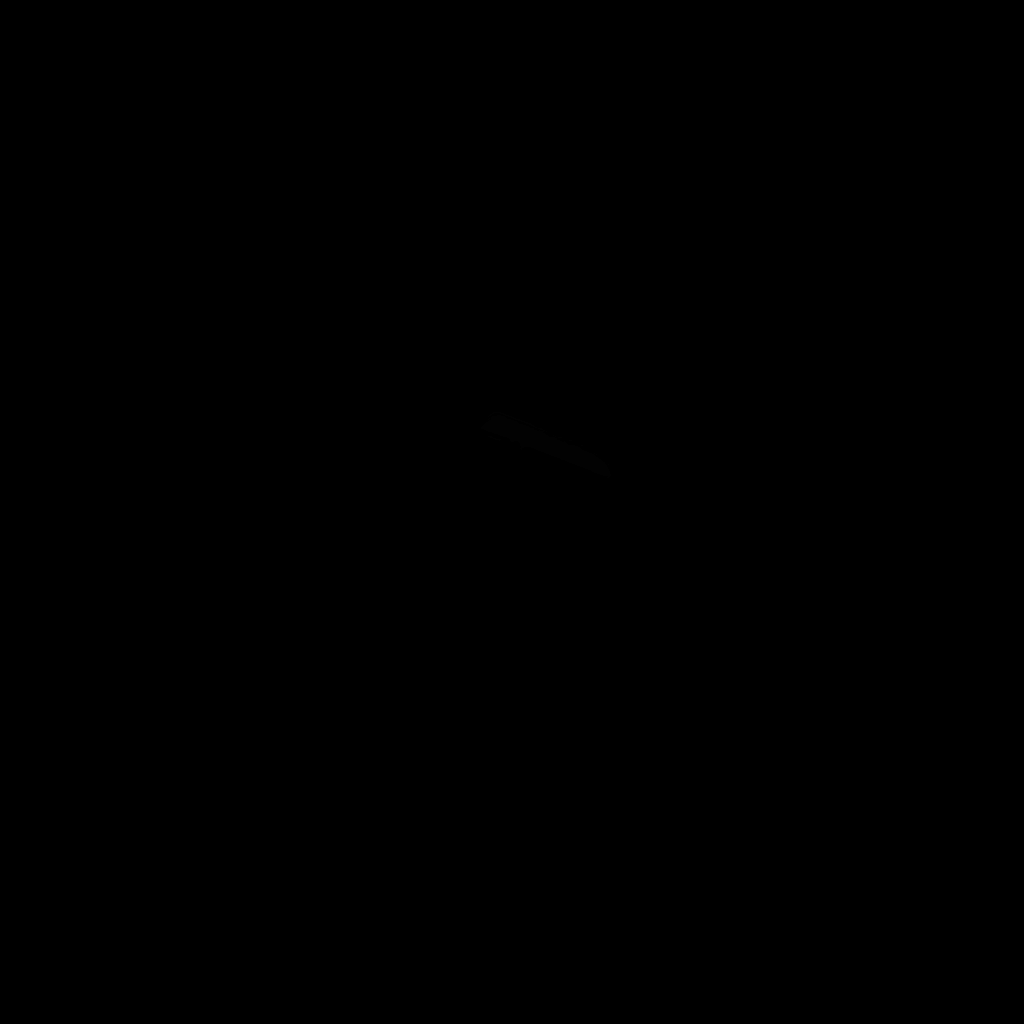

Supplement: Supplemental Information 1 [file peerj-cs-10-2097-s001.zip › IIT-AFF VL/masks/03_00000602.png]

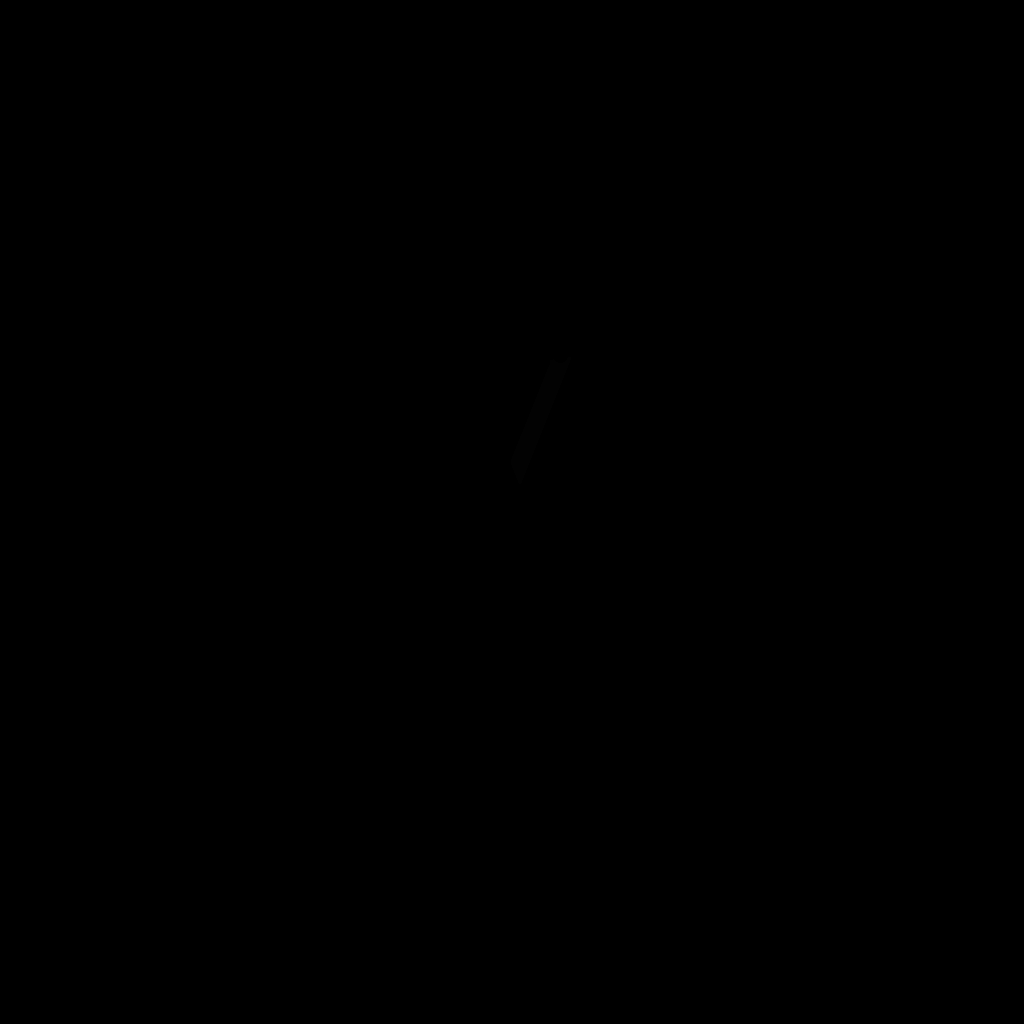

Supplement: Supplemental Information 1 [file peerj-cs-10-2097-s001.zip › IIT-AFF VL/masks/03_00000622.png]

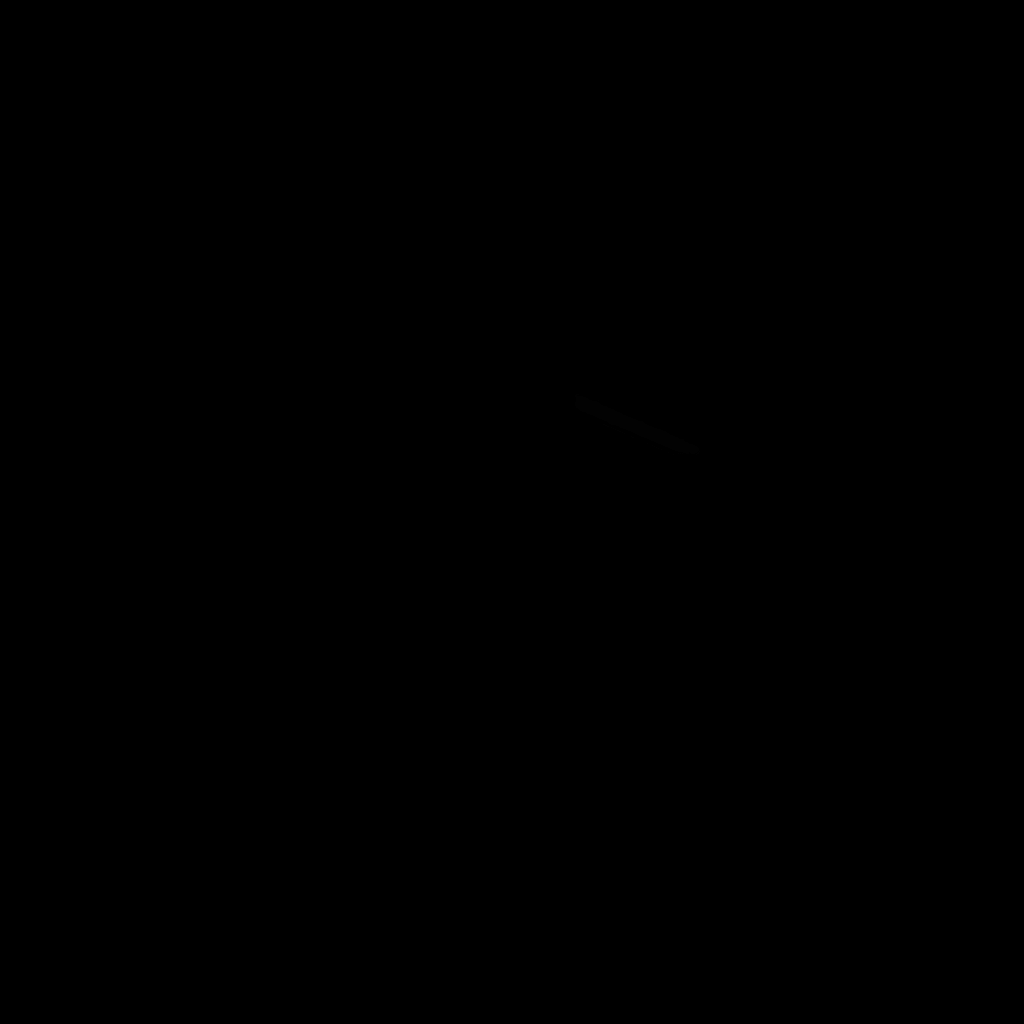

Supplement: Supplemental Information 1 [file peerj-cs-10-2097-s001.zip › IIT-AFF VL/masks/03_00000646.png]

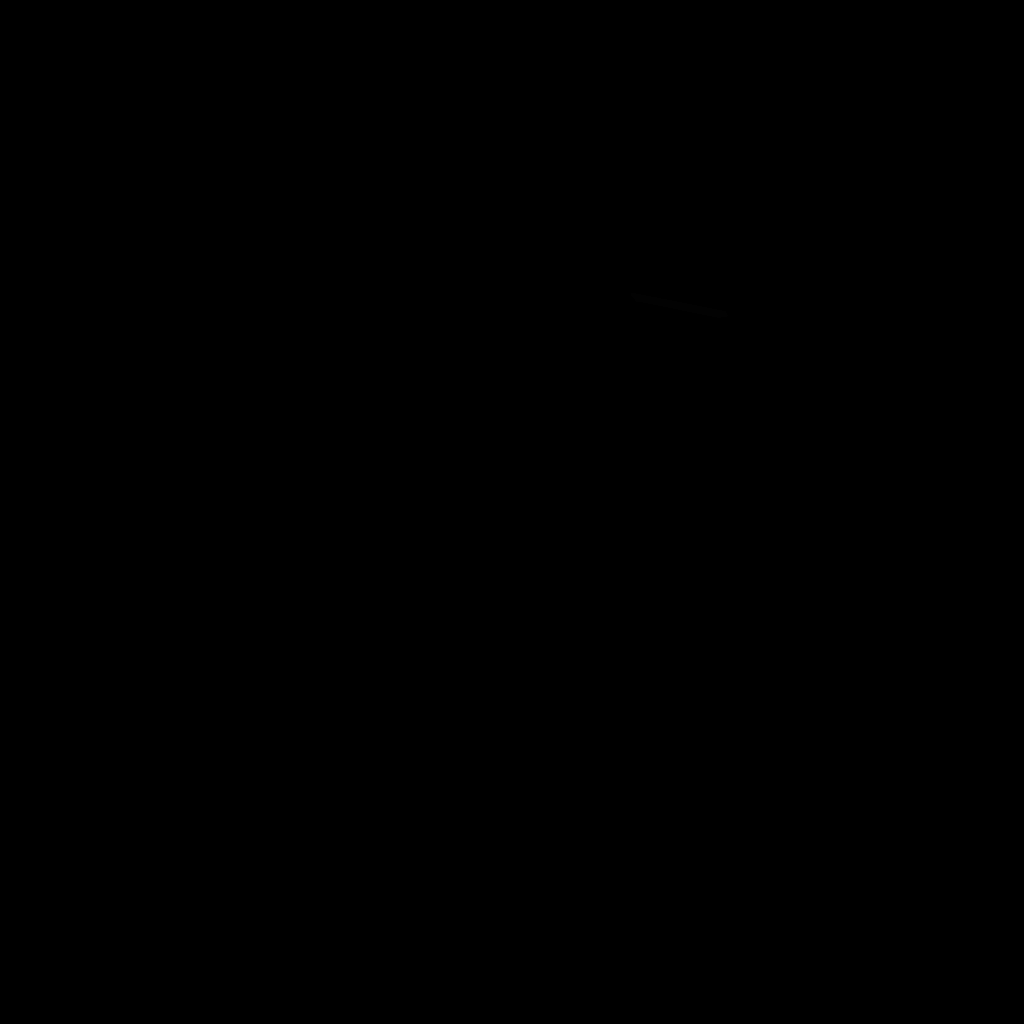

Supplement: Supplemental Information 1 [file peerj-cs-10-2097-s001.zip › IIT-AFF VL/masks/03_00000664.png]

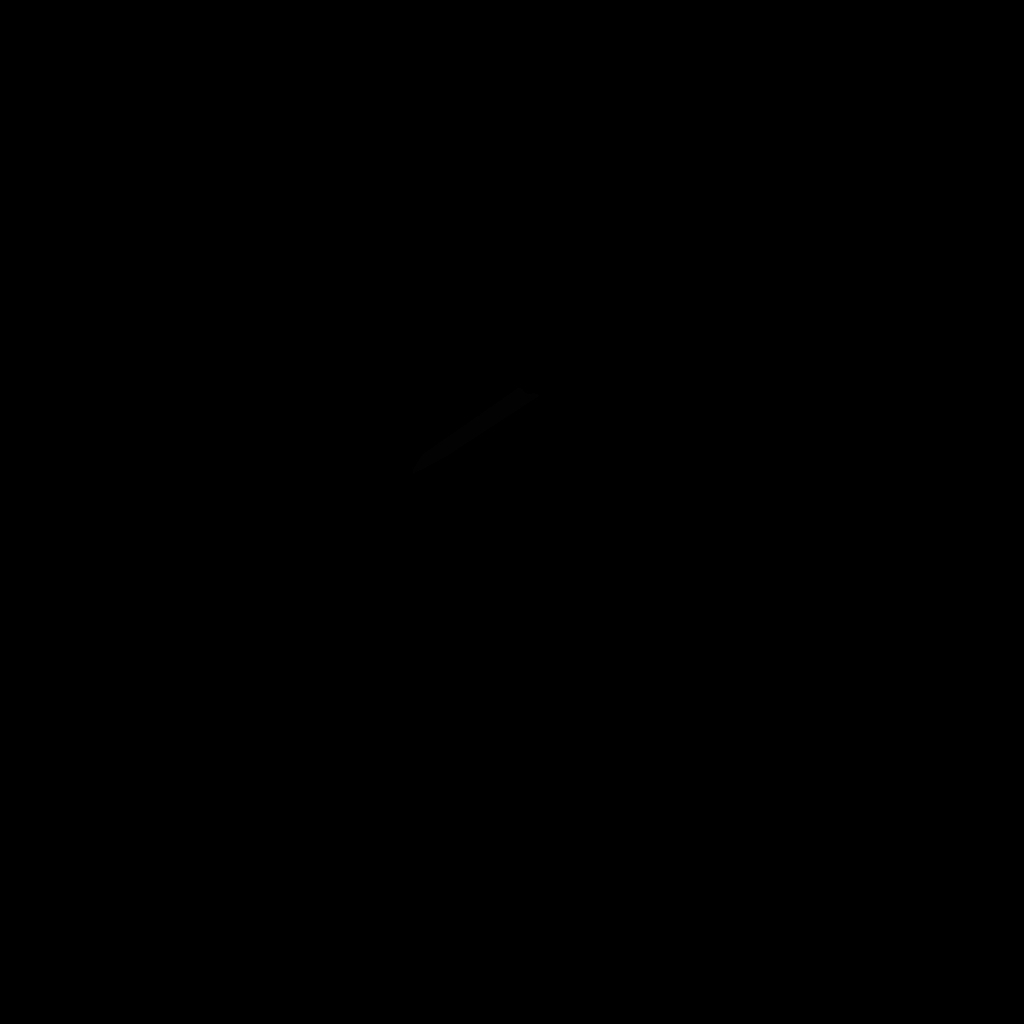

Supplement: Supplemental Information 1 [file peerj-cs-10-2097-s001.zip › IIT-AFF VL/masks/03_00000677.png]

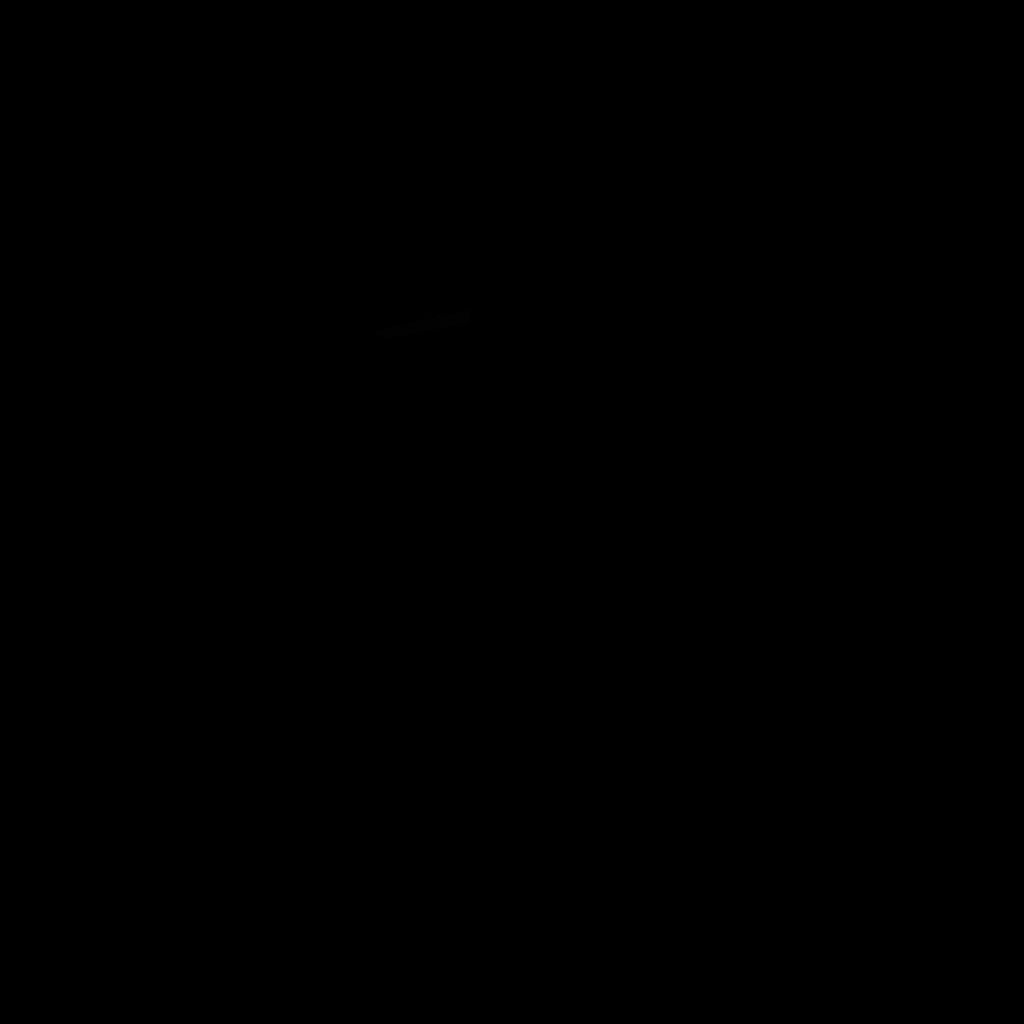

Supplement: Supplemental Information 1 [file peerj-cs-10-2097-s001.zip › IIT-AFF VL/masks/03_00000700.png]

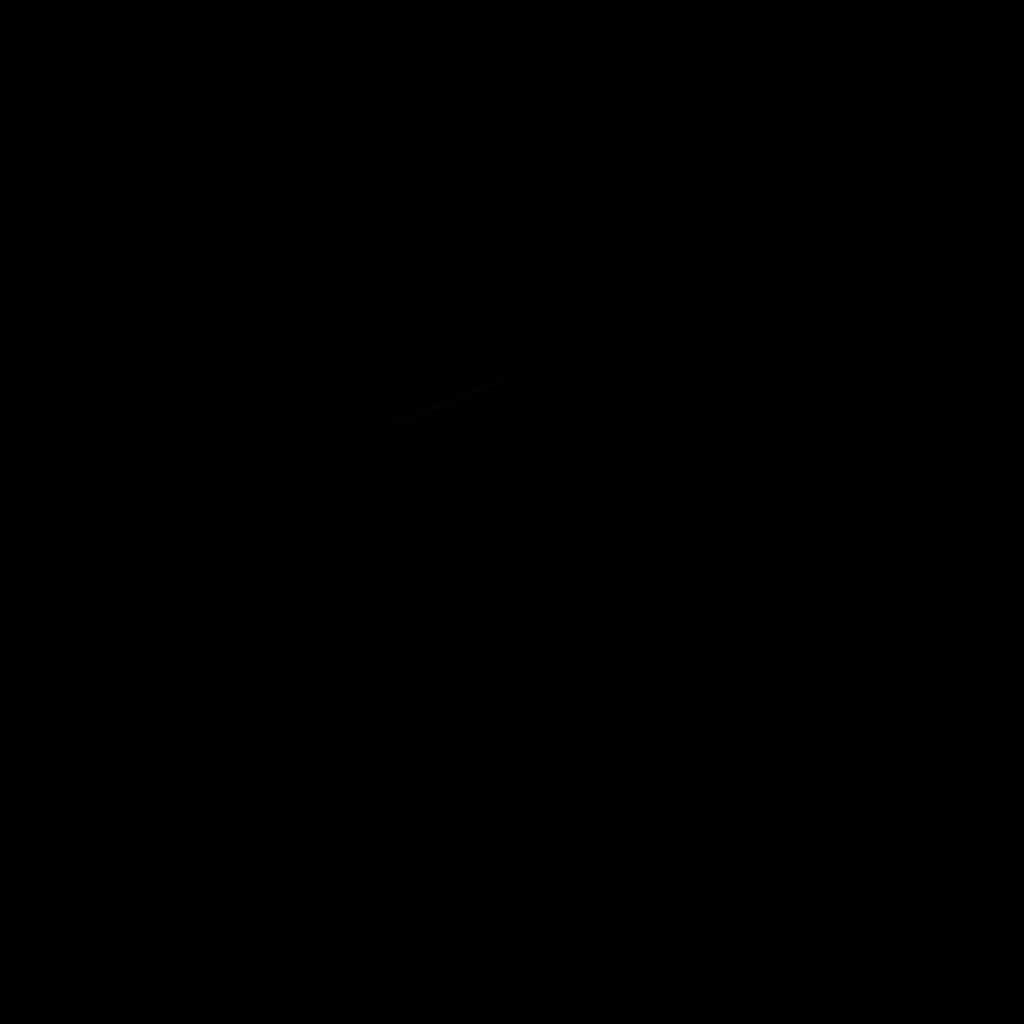

Supplement: Supplemental Information 1 [file peerj-cs-10-2097-s001.zip › IIT-AFF VL/masks/03_00000714.png]

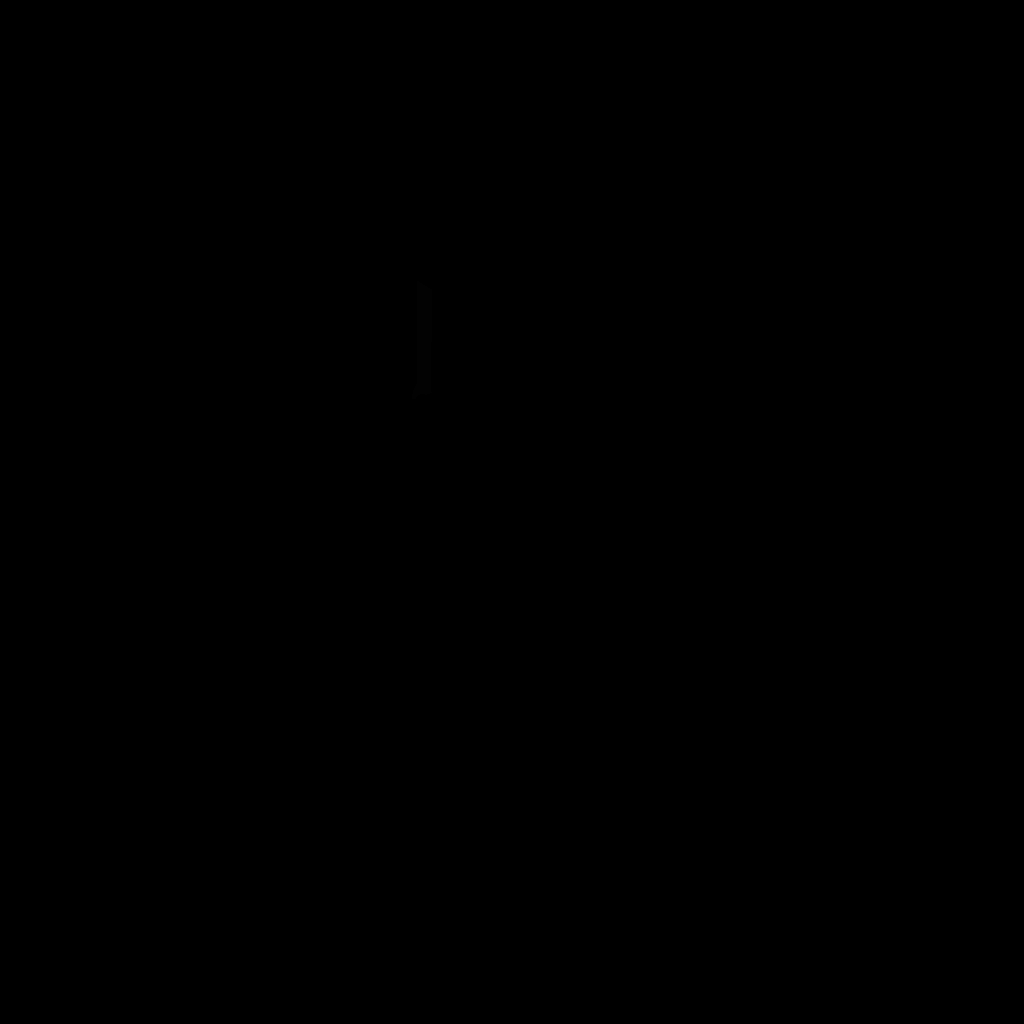

Supplement: Supplemental Information 1 [file peerj-cs-10-2097-s001.zip › IIT-AFF VL/masks/03_00000741.png]

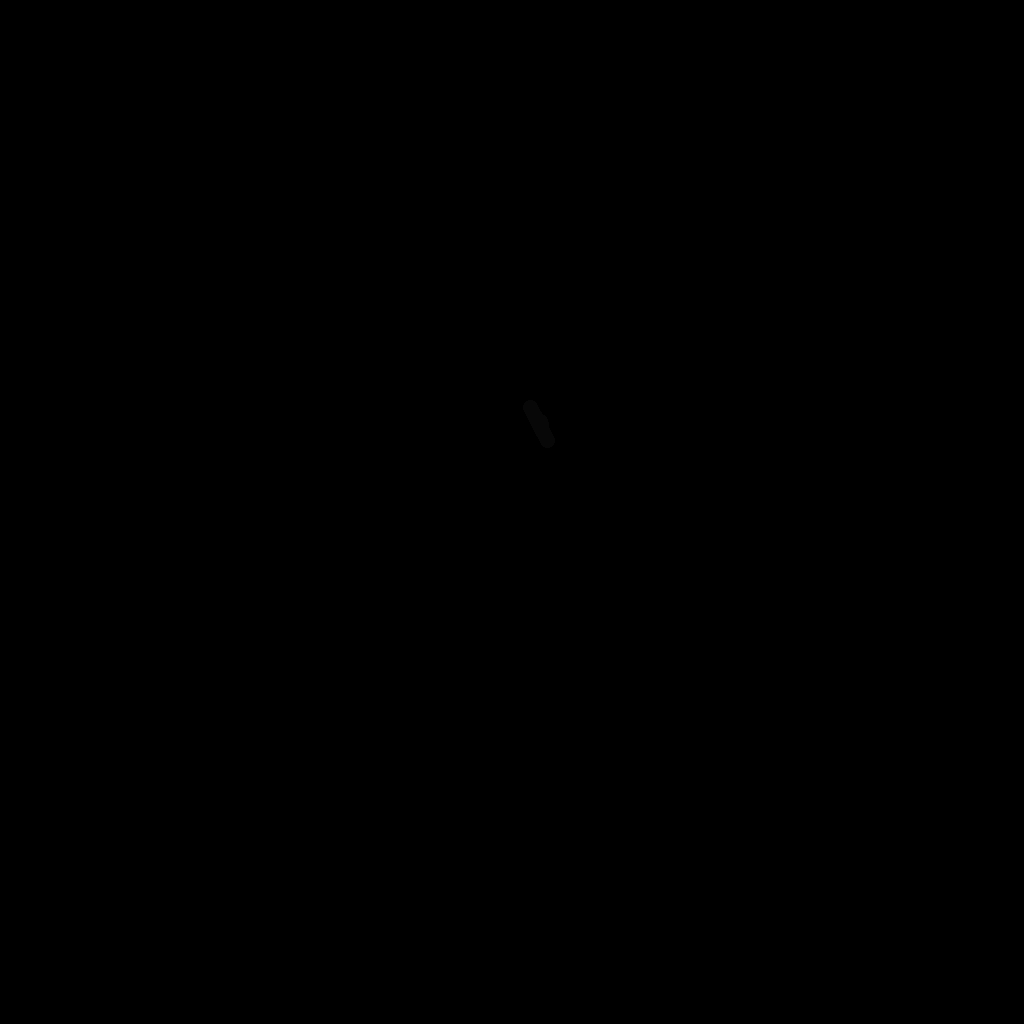

Supplement: Supplemental Information 1 [file peerj-cs-10-2097-s001.zip › IIT-AFF VL/masks/03_00000794.png]

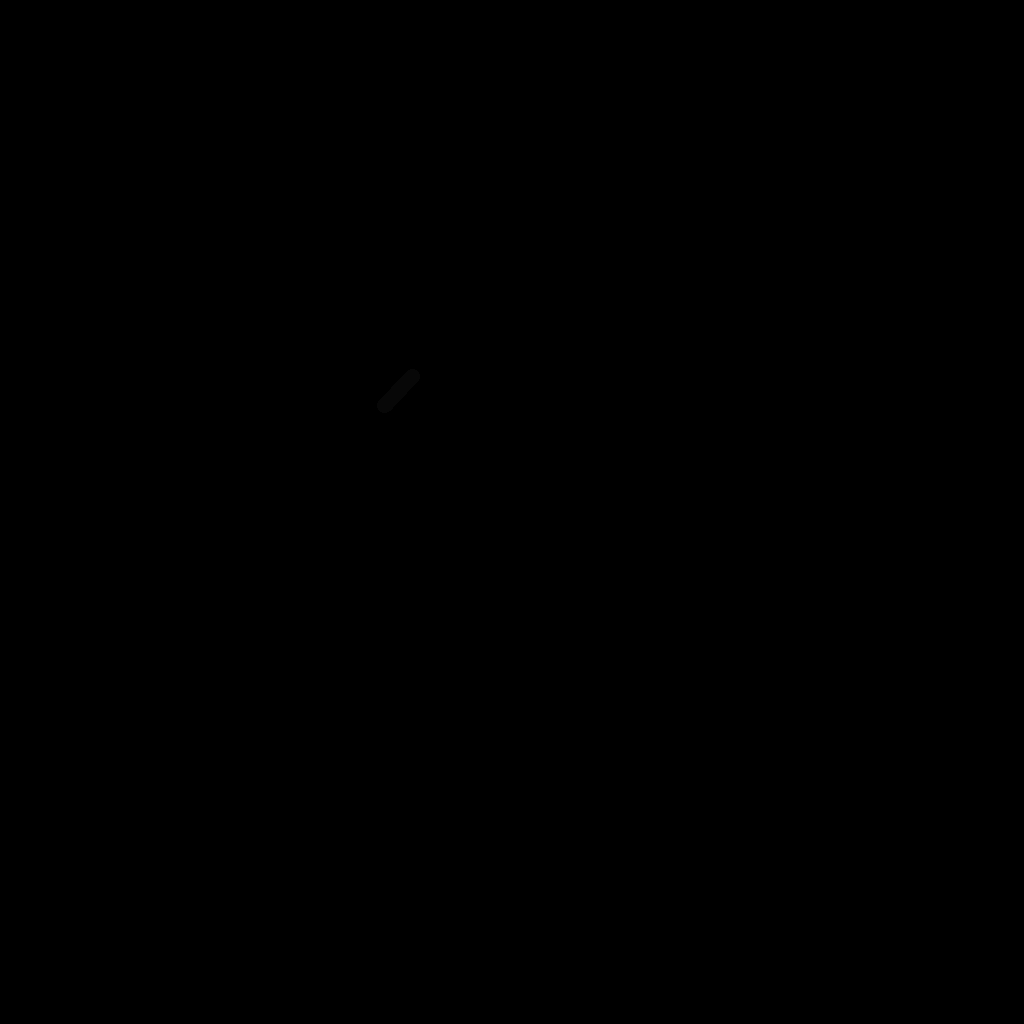

Supplement: Supplemental Information 1 [file peerj-cs-10-2097-s001.zip › IIT-AFF VL/masks/03_00000834.png]

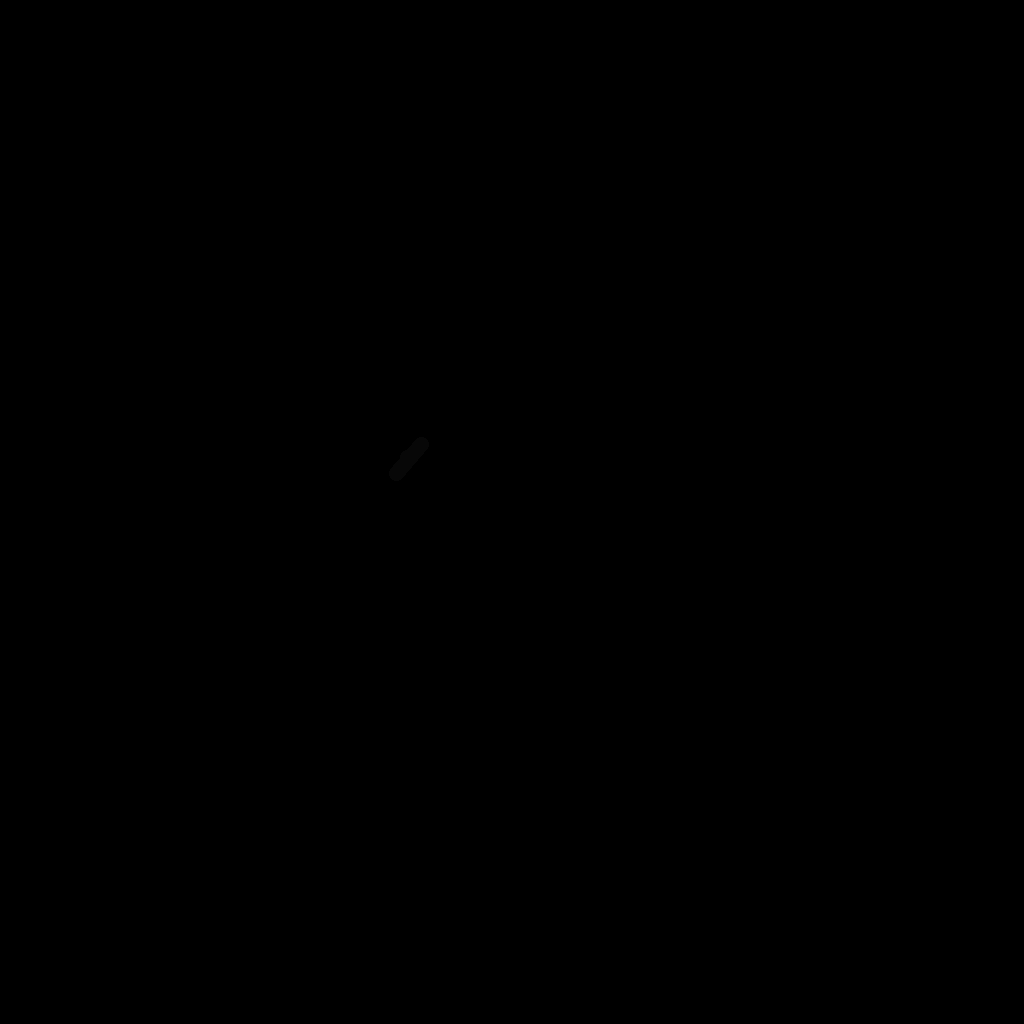

Supplement: Supplemental Information 1 [file peerj-cs-10-2097-s001.zip › IIT-AFF VL/masks/03_00000867.png]

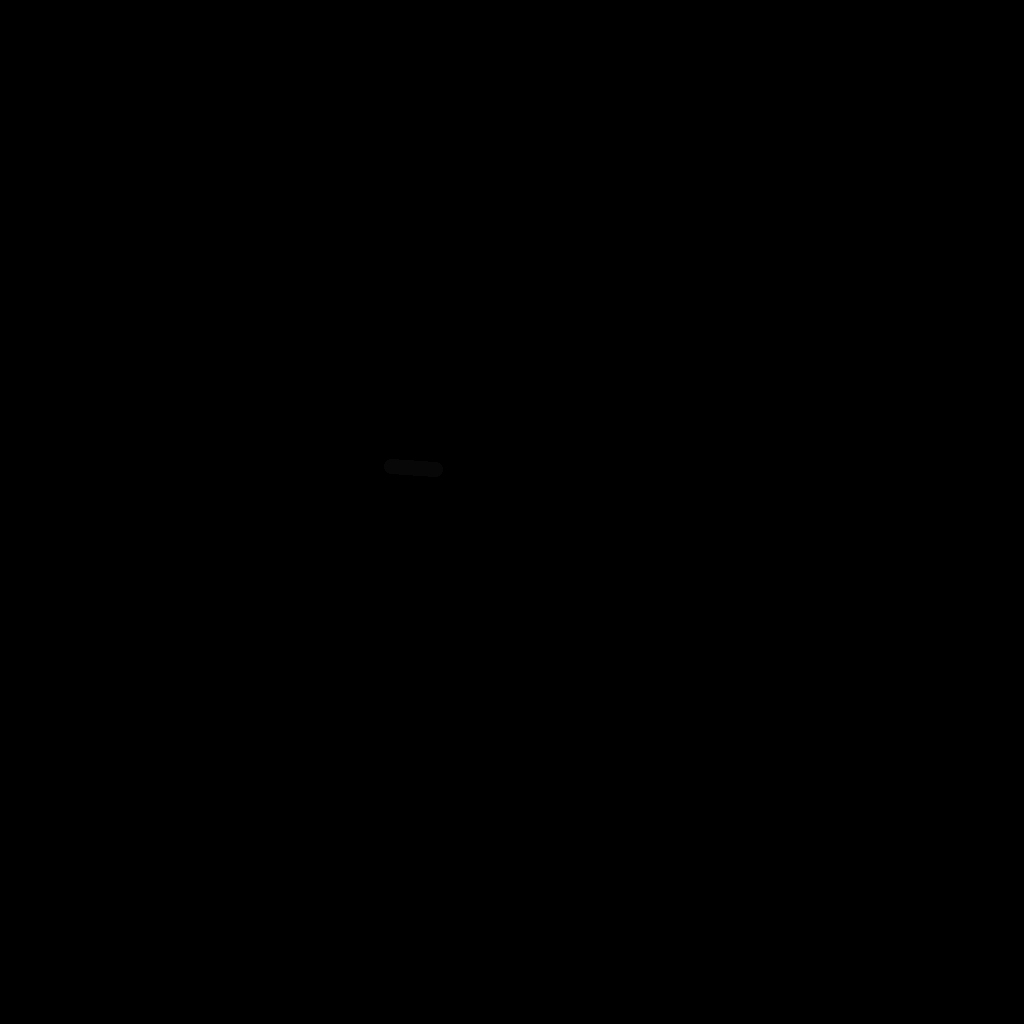

Supplement: Supplemental Information 1 [file peerj-cs-10-2097-s001.zip › IIT-AFF VL/masks/03_00000901.png]

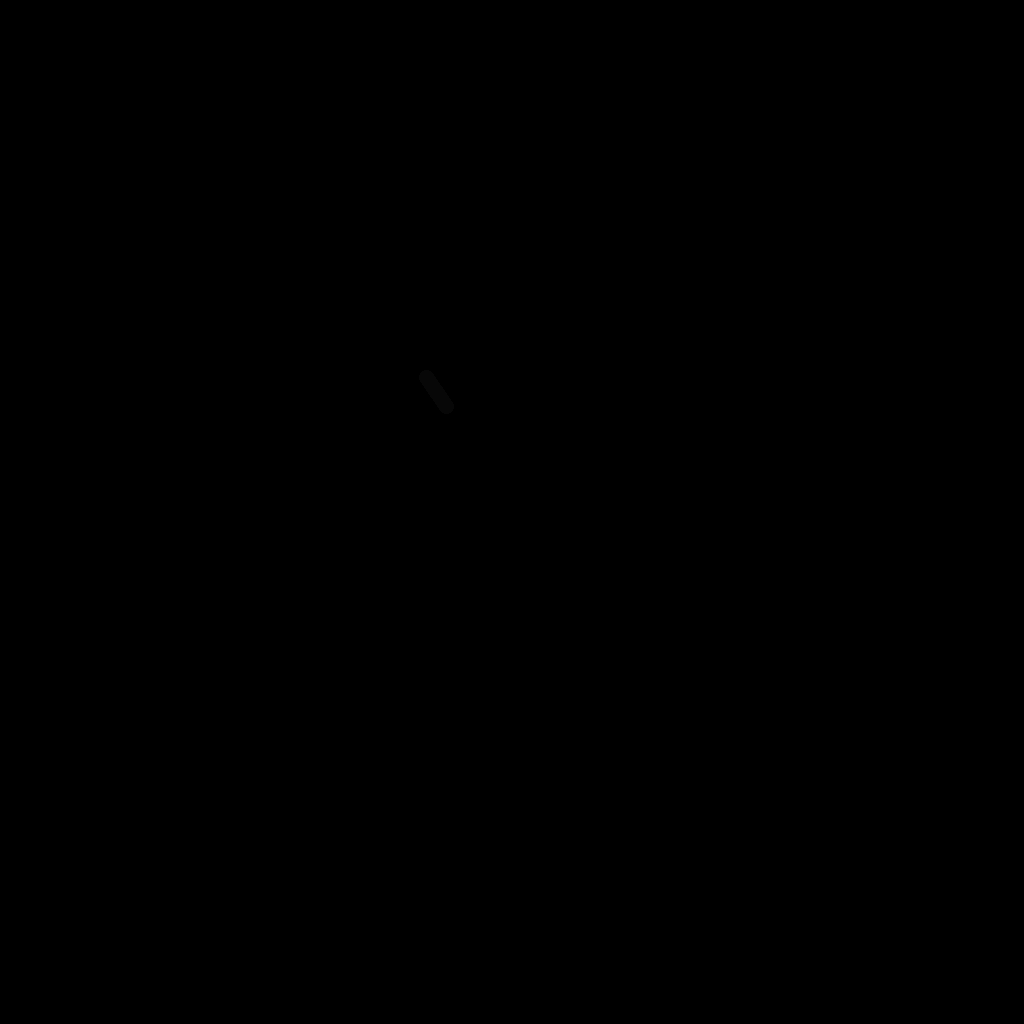

Supplement: Supplemental Information 1 [file peerj-cs-10-2097-s001.zip › IIT-AFF VL/masks/03_00000969.png]

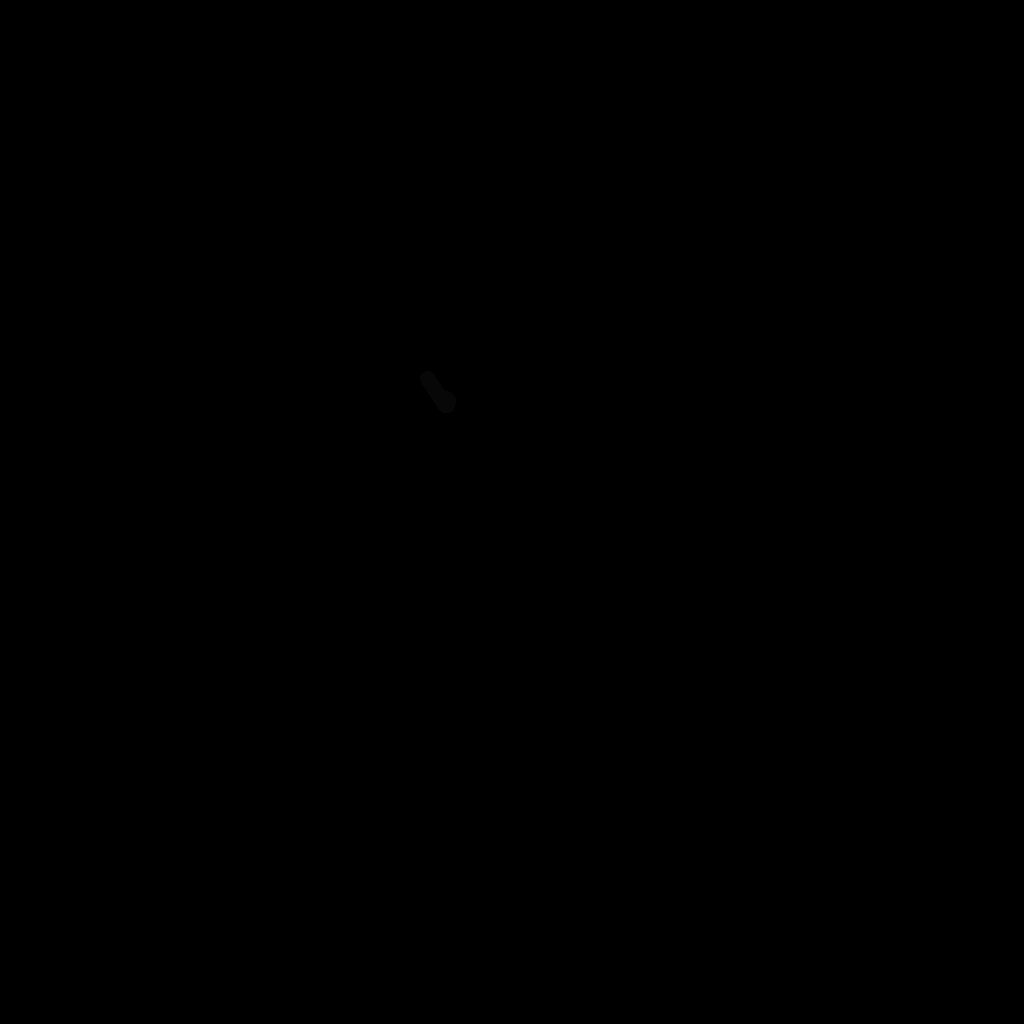

Supplement: Supplemental Information 1 [file peerj-cs-10-2097-s001.zip › IIT-AFF VL/masks/04_00000030.png]

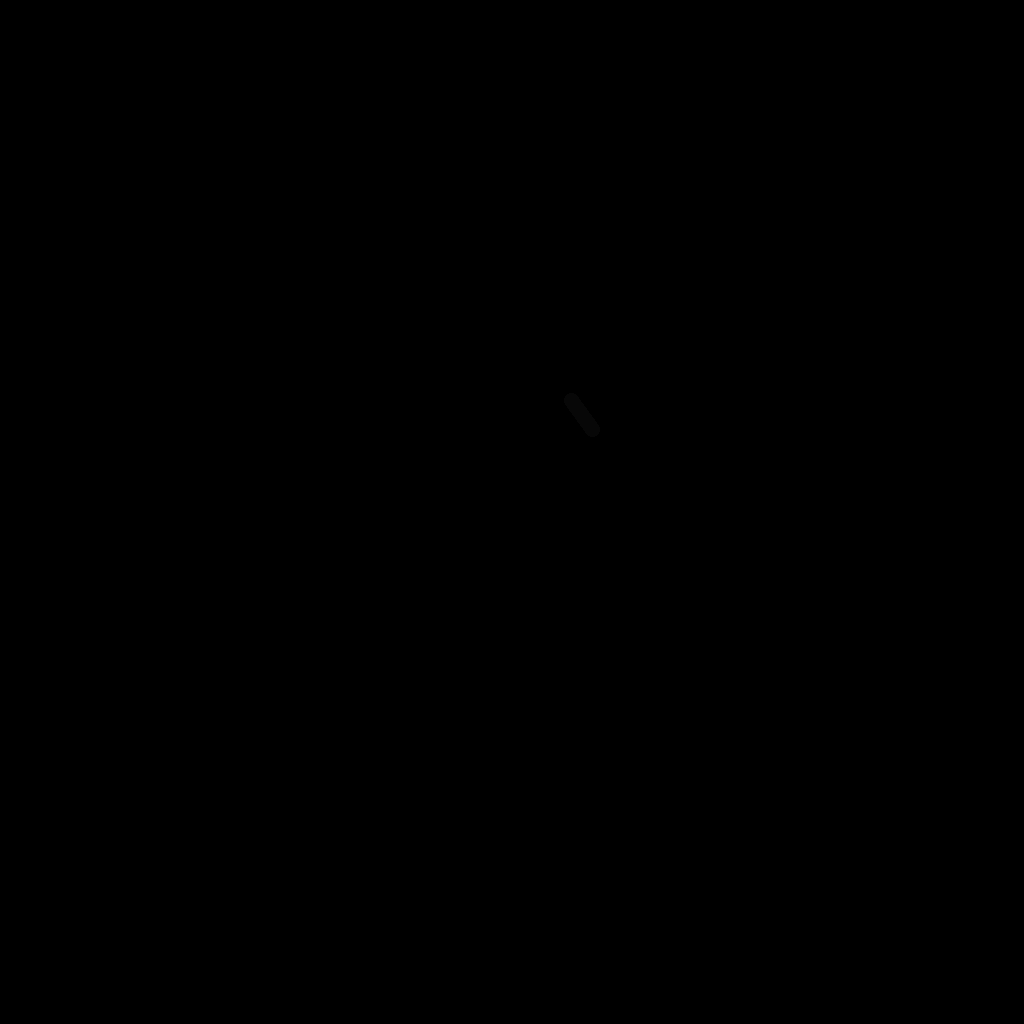

Supplement: Supplemental Information 1 [file peerj-cs-10-2097-s001.zip › IIT-AFF VL/masks/04_00000066.png]

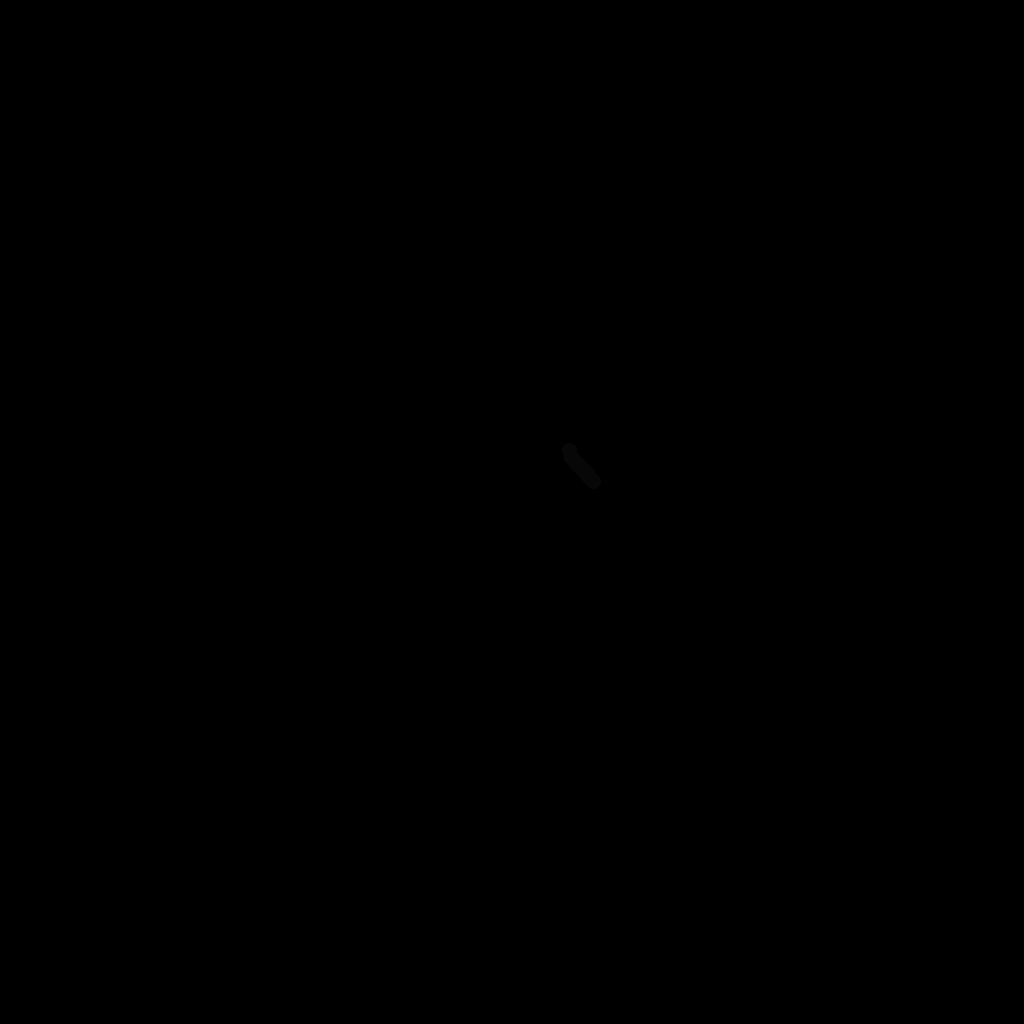

Supplement: Supplemental Information 1 [file peerj-cs-10-2097-s001.zip › IIT-AFF VL/masks/04_00000083.png]

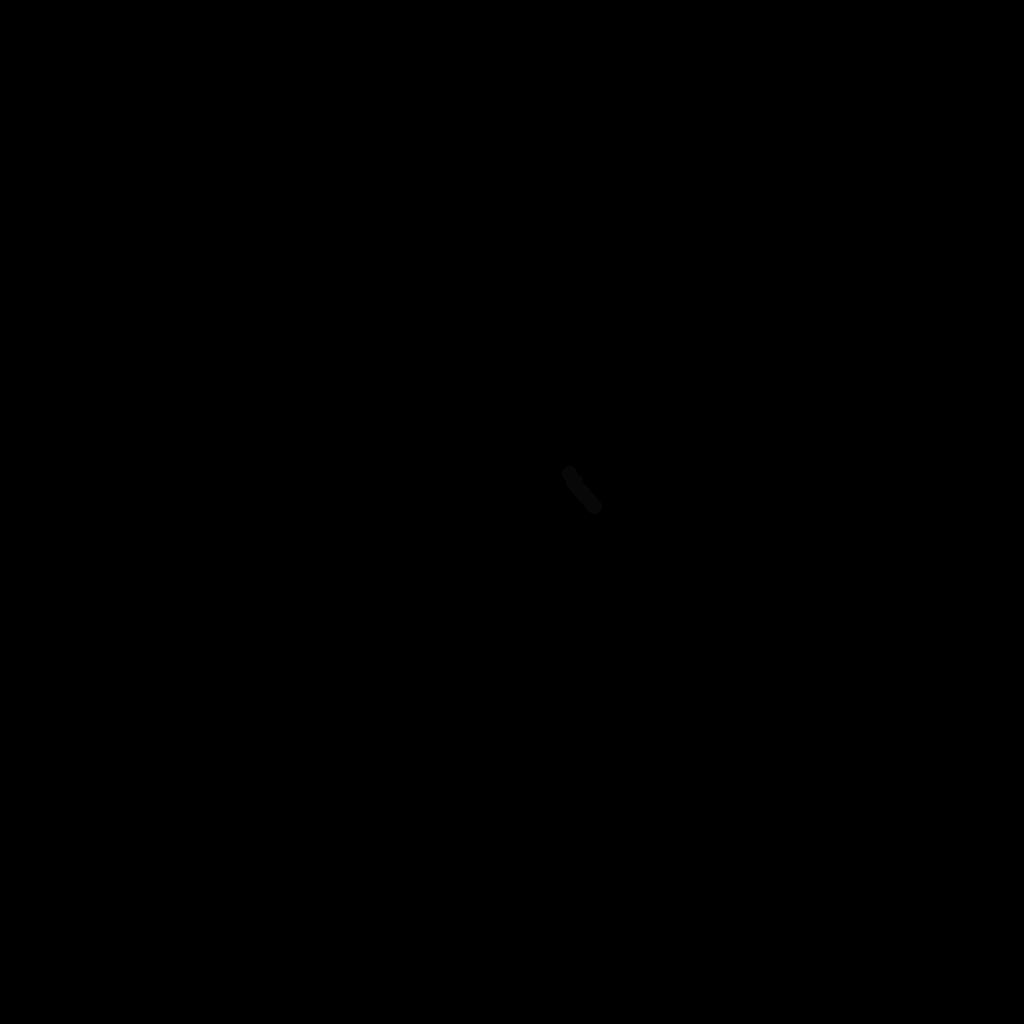

Supplement: Supplemental Information 1 [file peerj-cs-10-2097-s001.zip › IIT-AFF VL/masks/04_00000086.png]

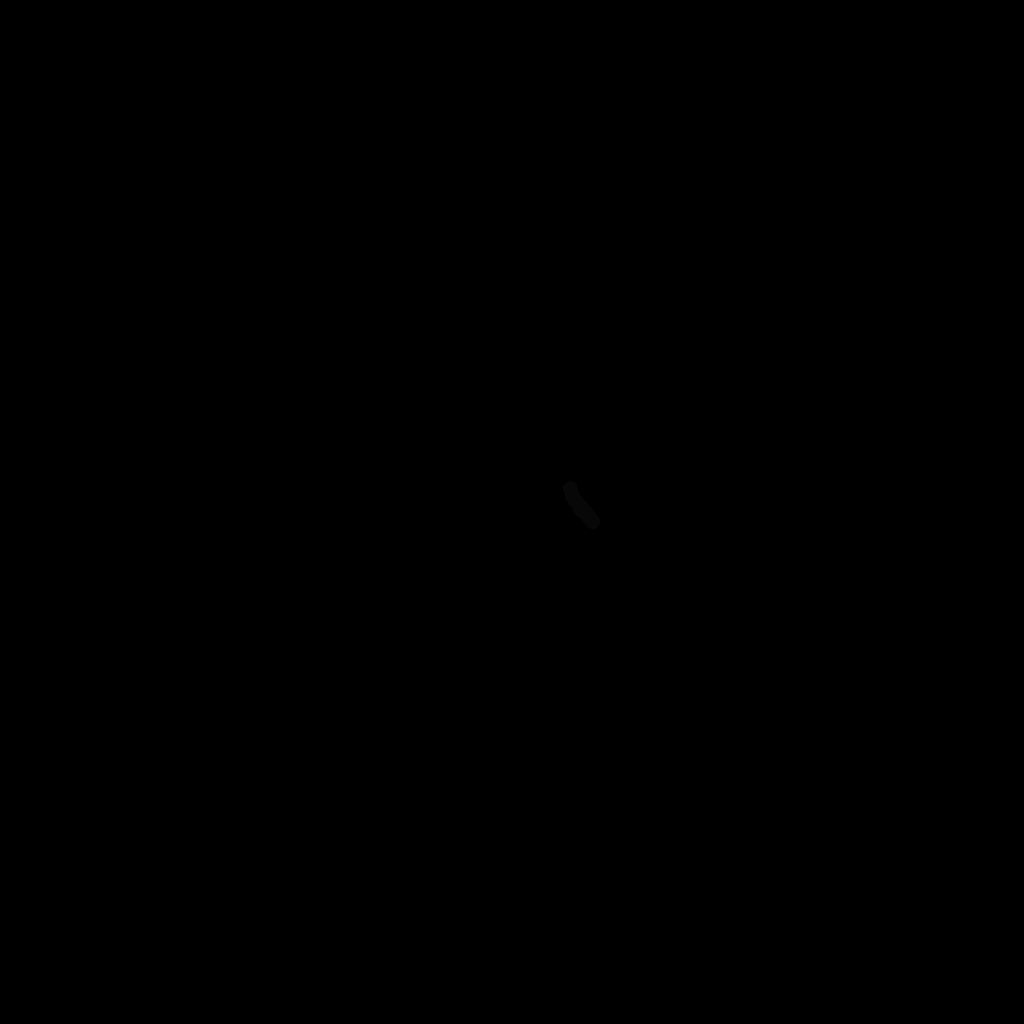

Supplement: Supplemental Information 1 [file peerj-cs-10-2097-s001.zip › IIT-AFF VL/masks/04_00000090.png]

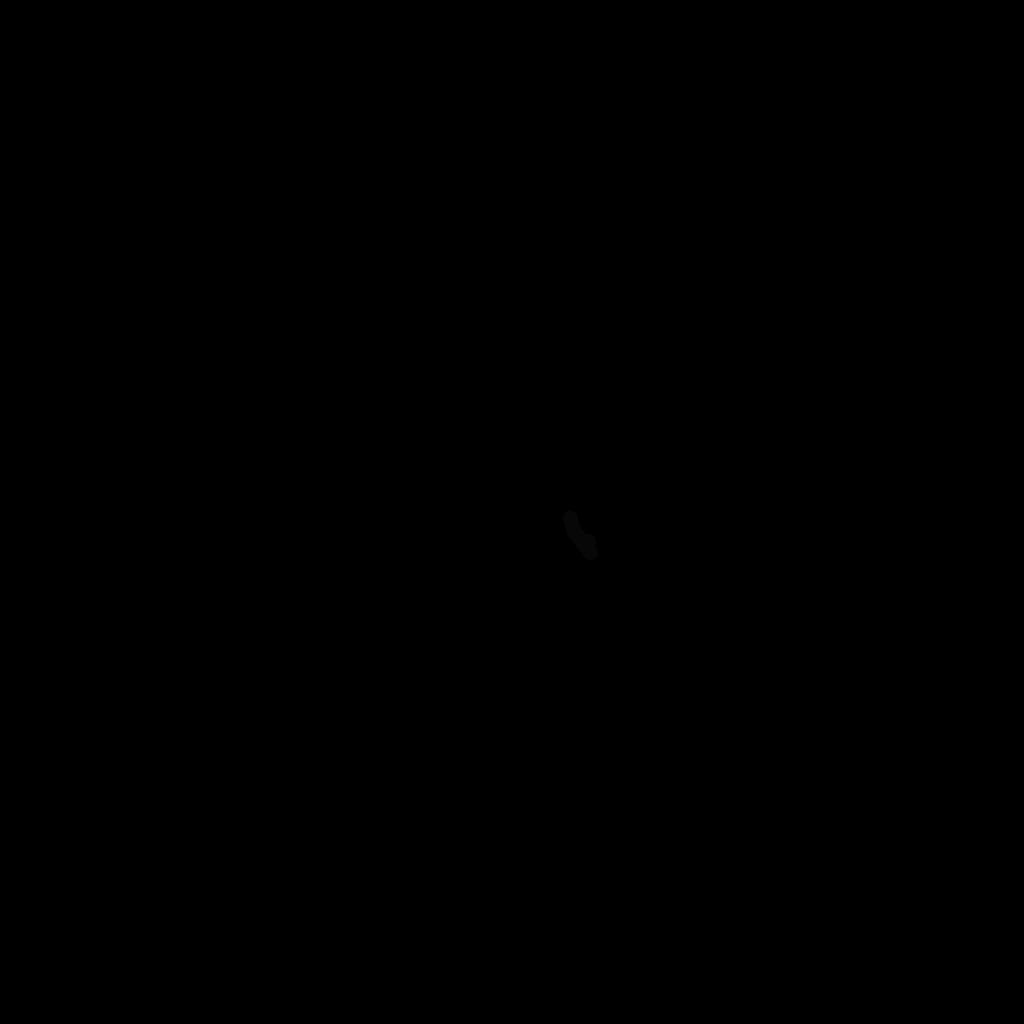

Supplement: Supplemental Information 1 [file peerj-cs-10-2097-s001.zip › IIT-AFF VL/masks/04_00000094.png]

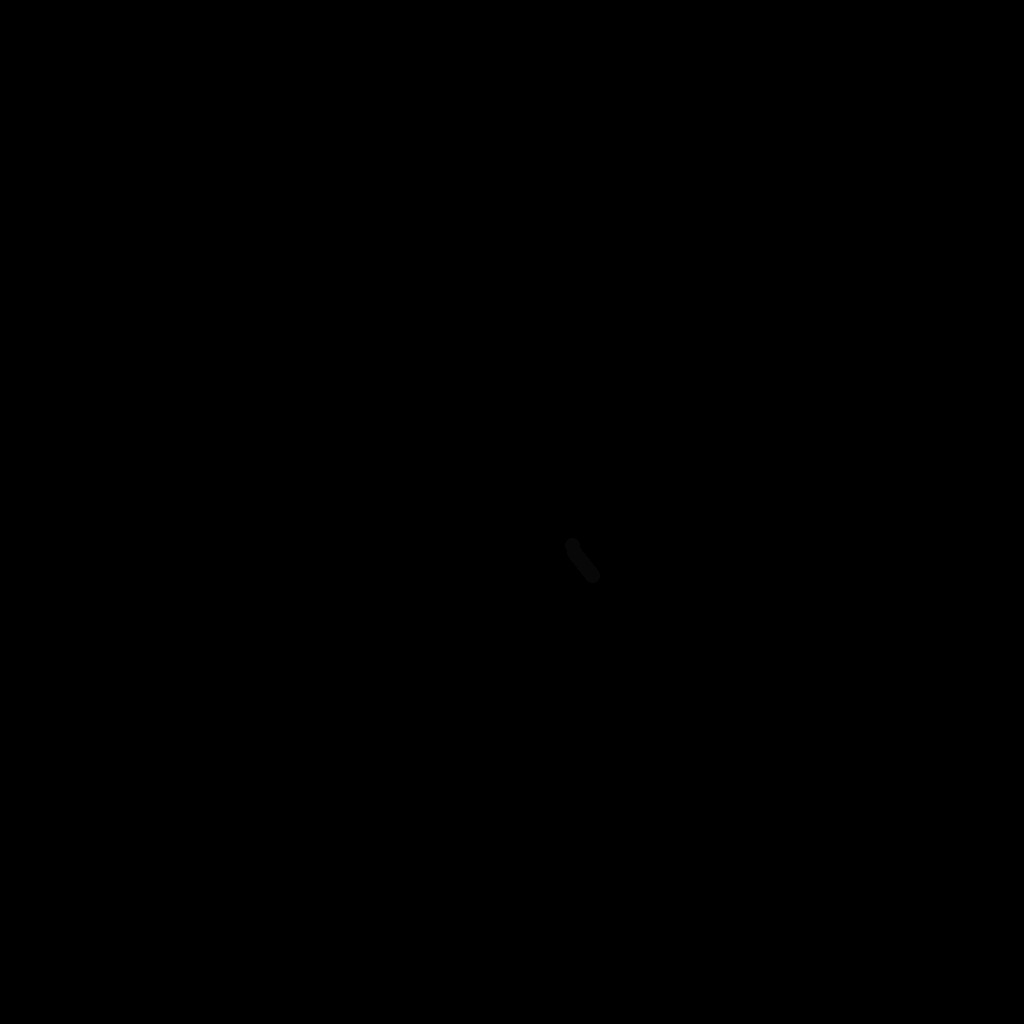

Supplement: Supplemental Information 1 [file peerj-cs-10-2097-s001.zip › IIT-AFF VL/masks/04_00000098.png]

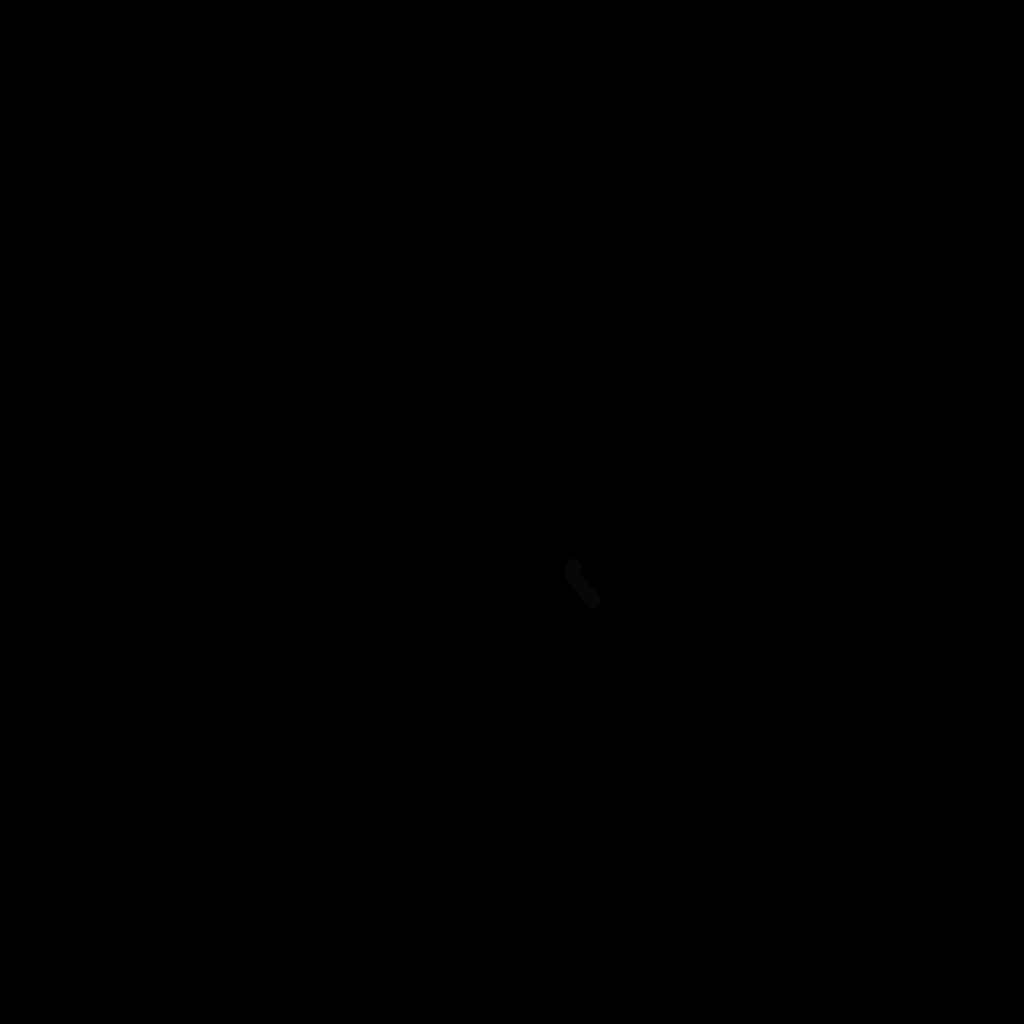

Supplement: Supplemental Information 1 [file peerj-cs-10-2097-s001.zip › IIT-AFF VL/masks/04_00000102.png]

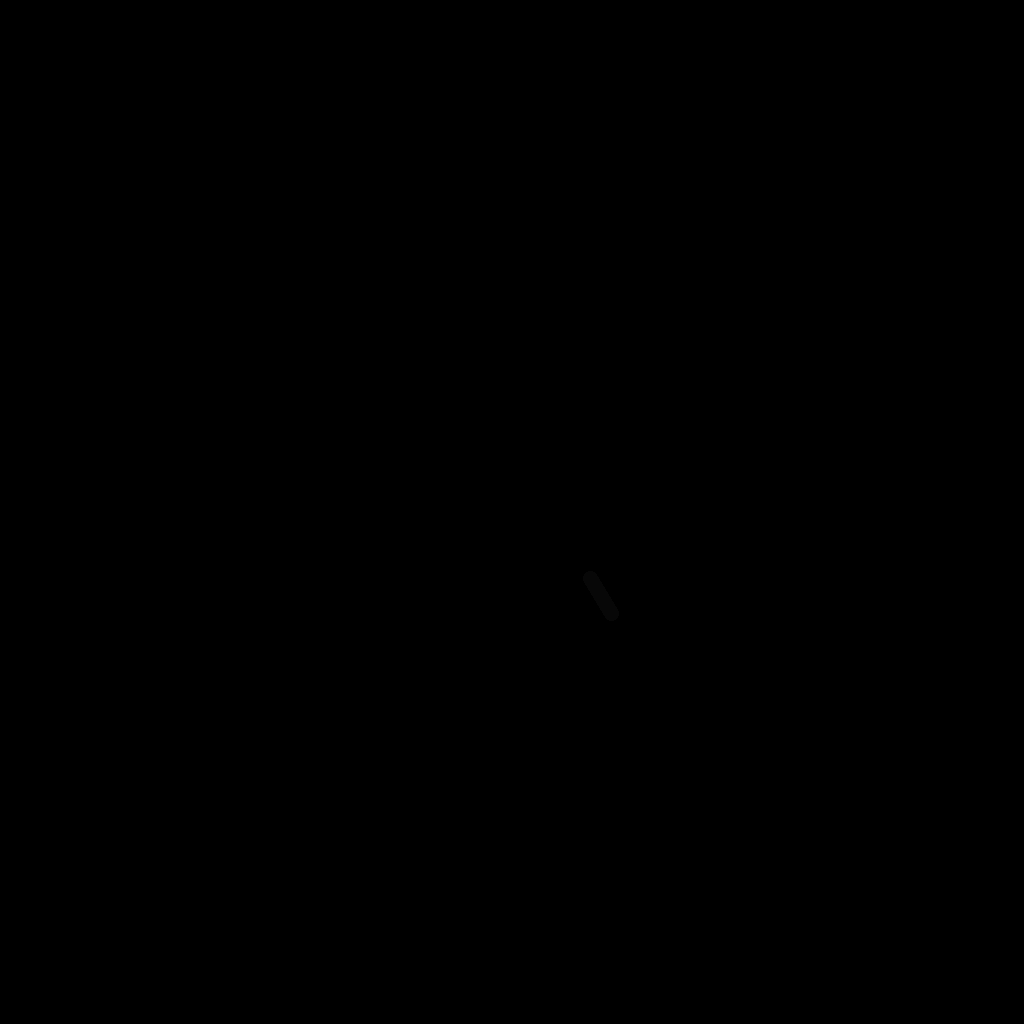

Supplement: Supplemental Information 1 [file peerj-cs-10-2097-s001.zip › IIT-AFF VL/masks/04_00000106.png]

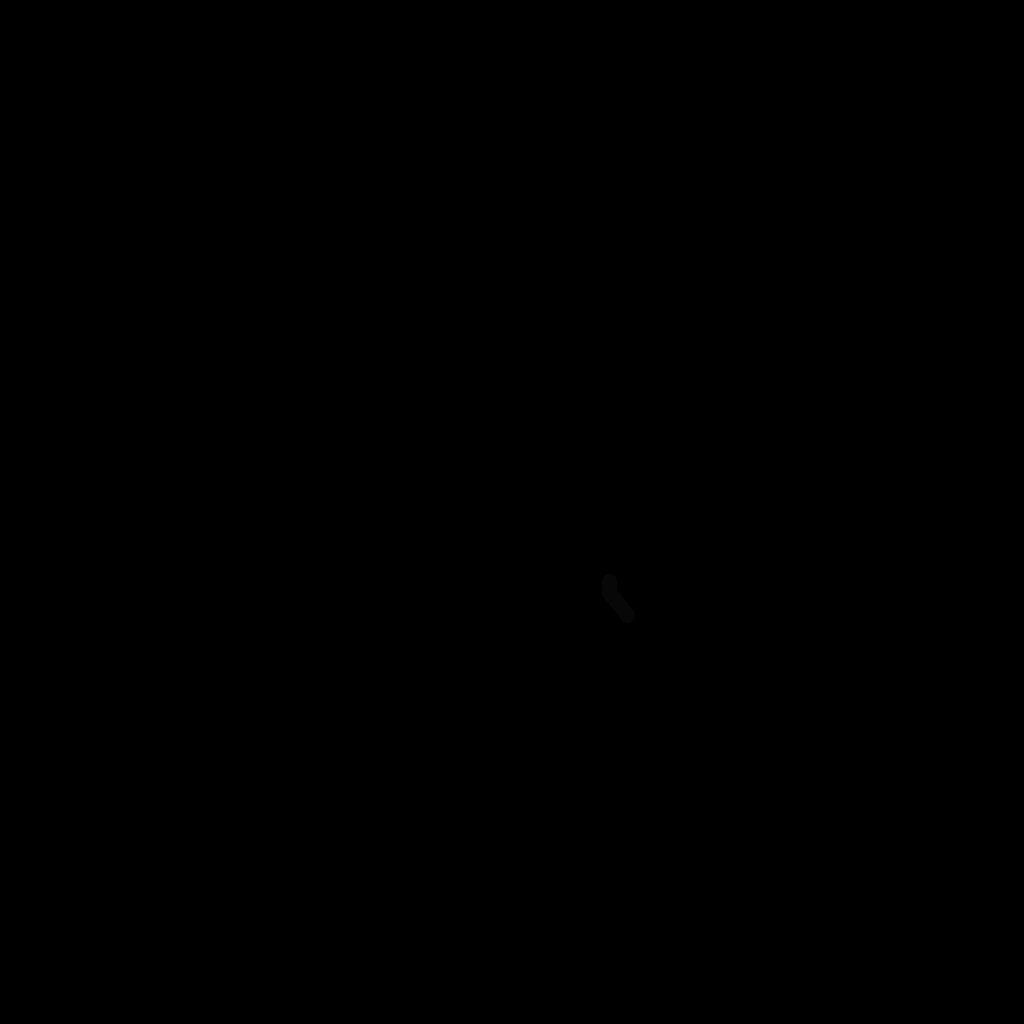

Supplement: Supplemental Information 1 [file peerj-cs-10-2097-s001.zip › IIT-AFF VL/masks/04_00000110.png]

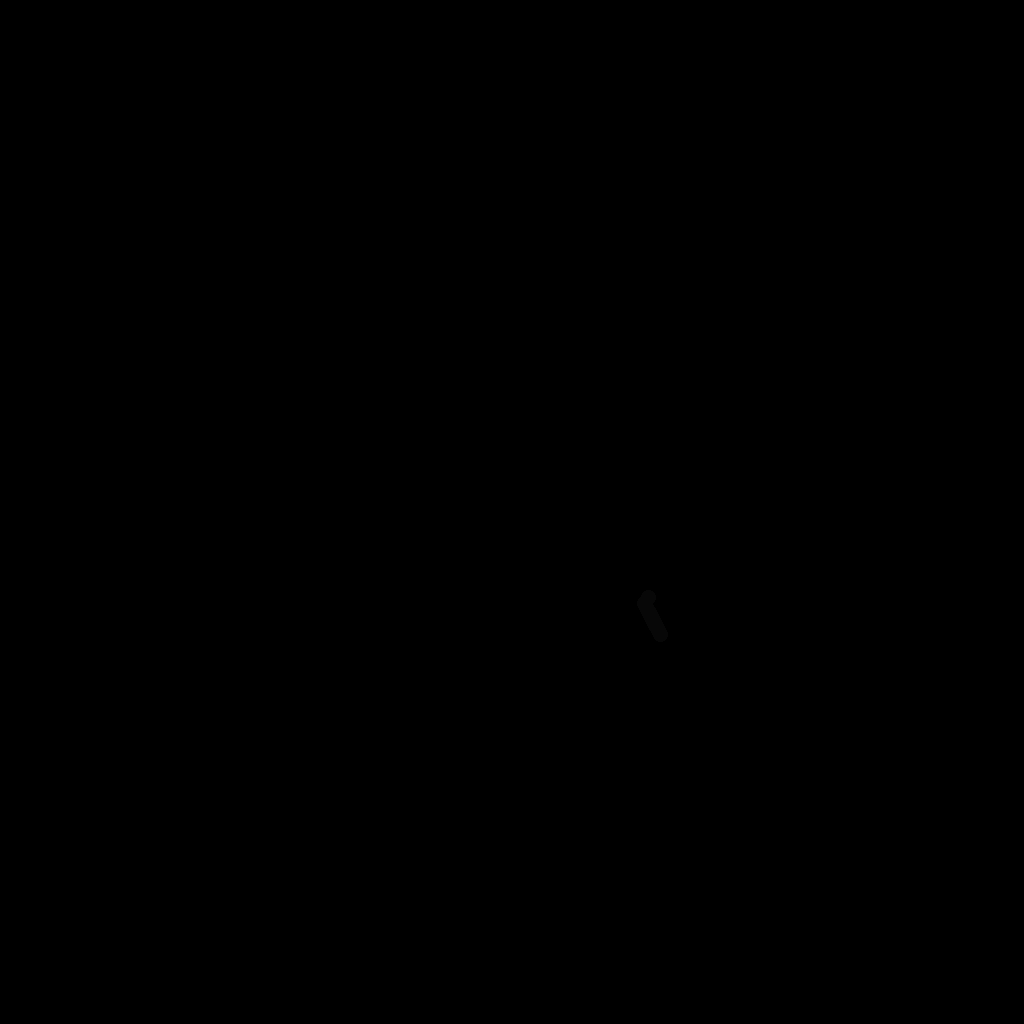

Supplement: Supplemental Information 1 [file peerj-cs-10-2097-s001.zip › IIT-AFF VL/masks/04_00000114.png]

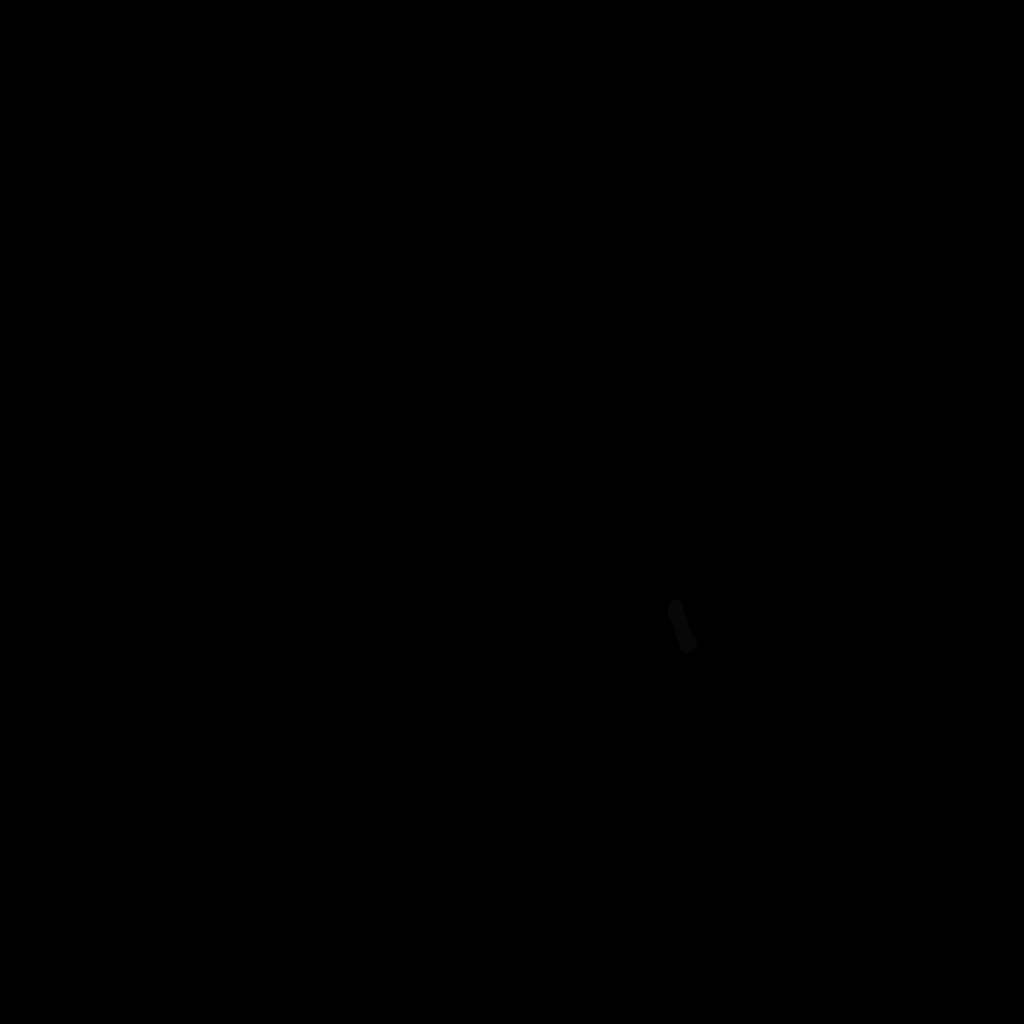

Supplement: Supplemental Information 1 [file peerj-cs-10-2097-s001.zip › IIT-AFF VL/masks/04_00000118.png]

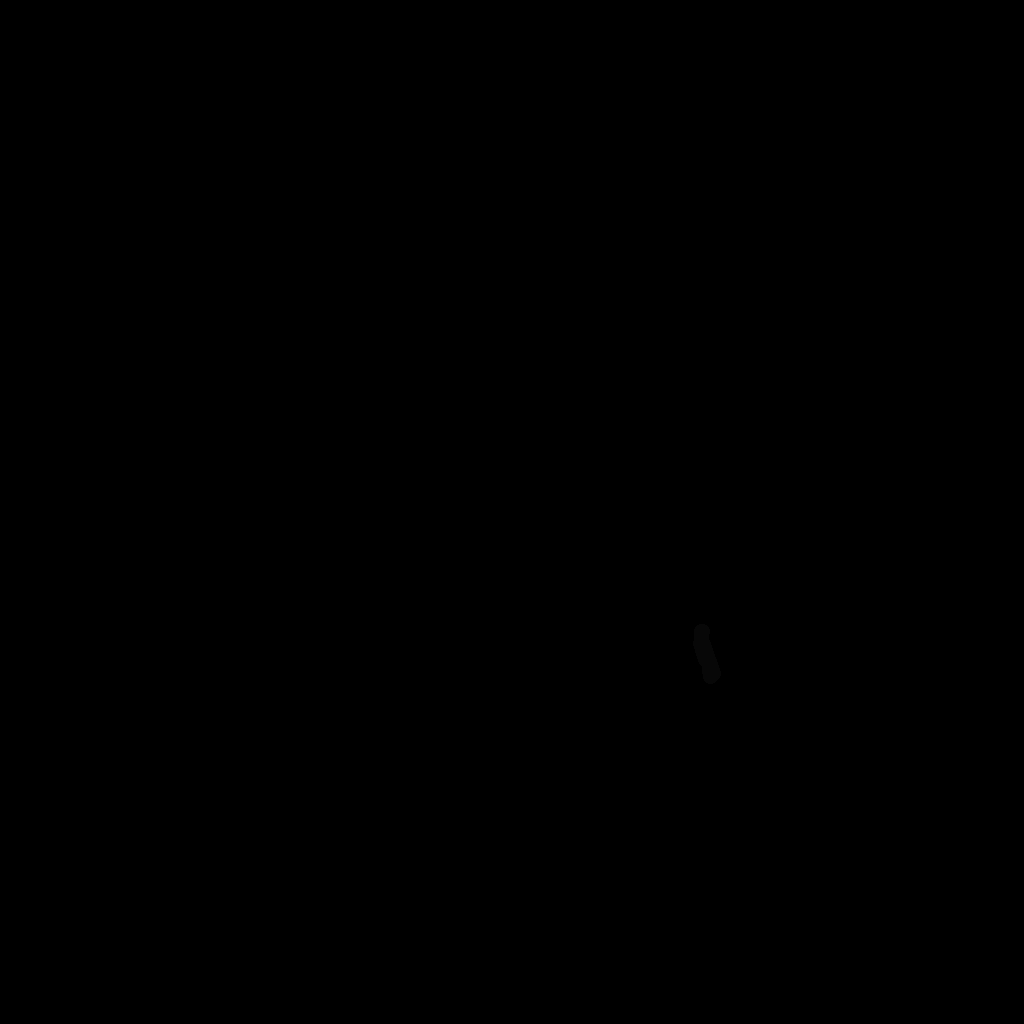

Supplement: Supplemental Information 1 [file peerj-cs-10-2097-s001.zip › IIT-AFF VL/masks/04_00000122.png]

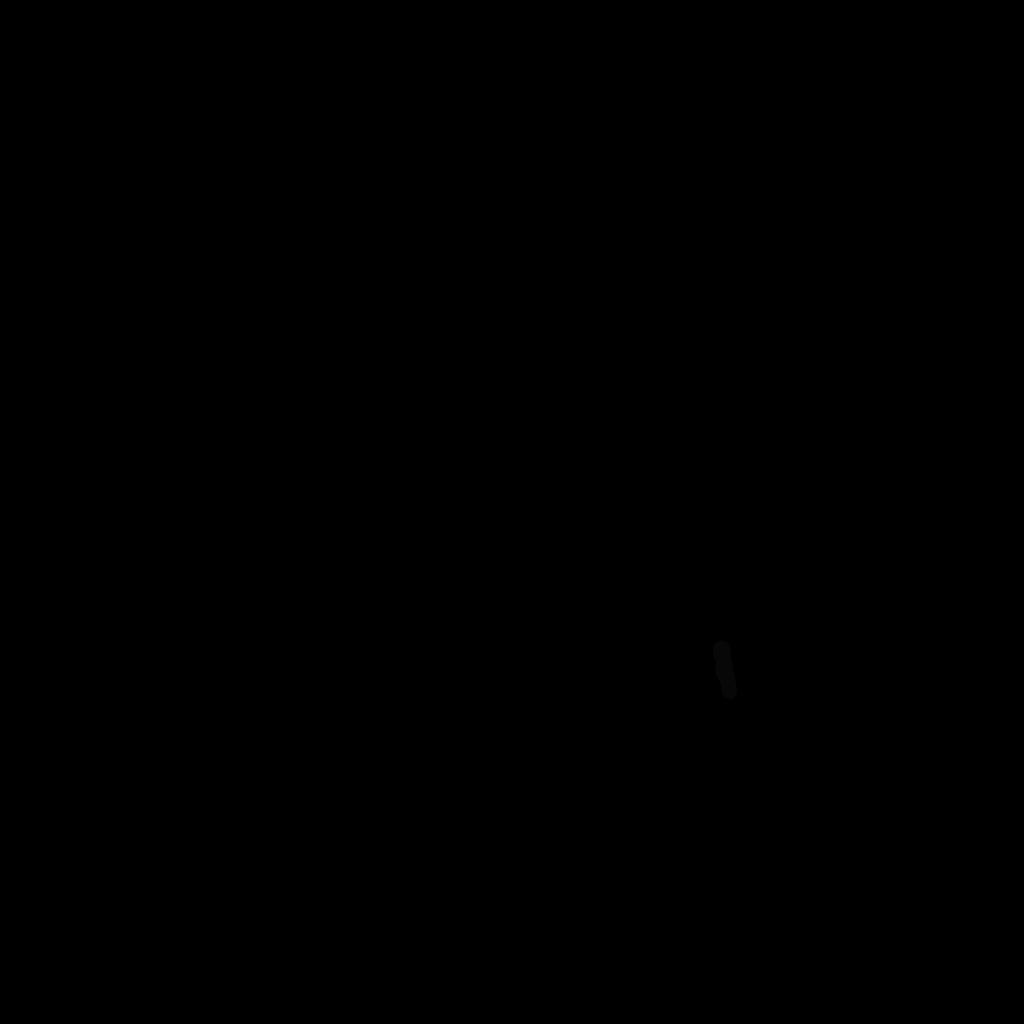

Supplement: Supplemental Information 1 [file peerj-cs-10-2097-s001.zip › IIT-AFF VL/masks/04_00000126.png]

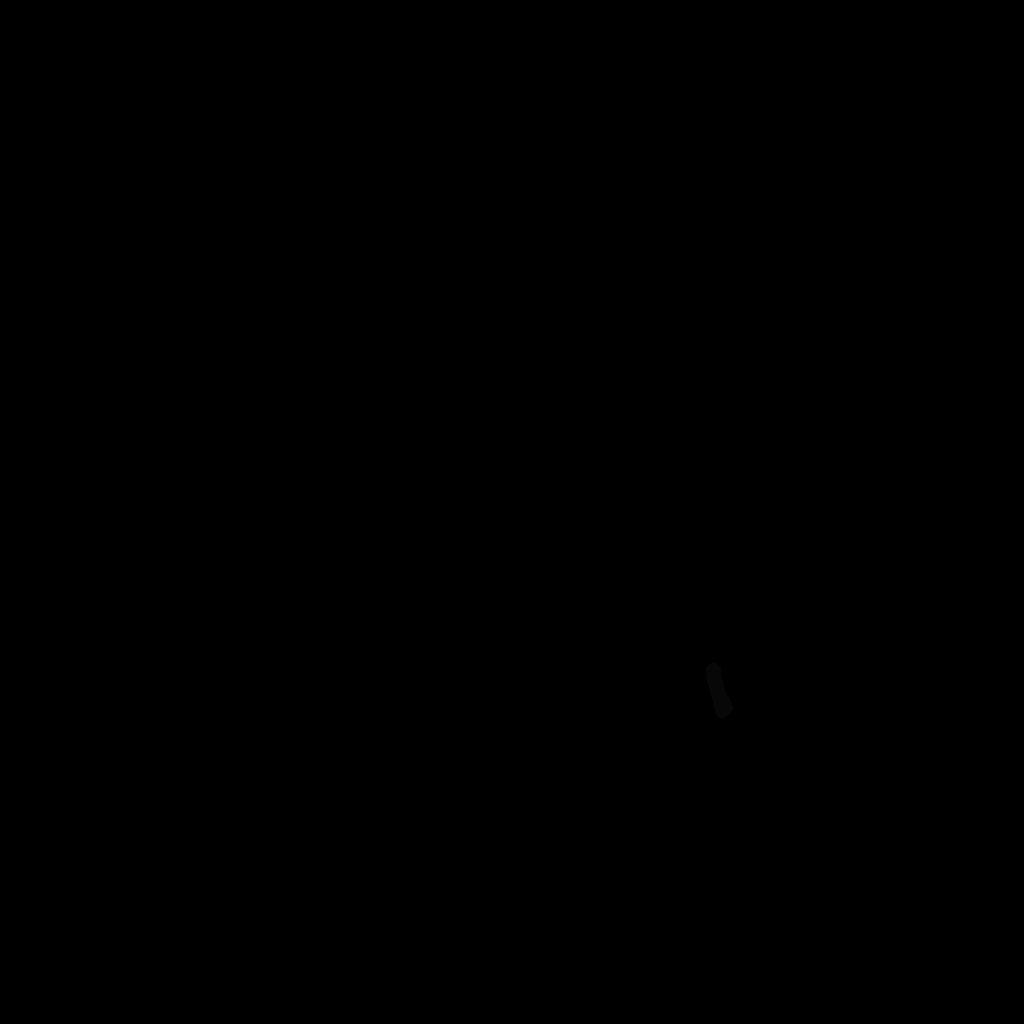

Supplement: Supplemental Information 1 [file peerj-cs-10-2097-s001.zip › IIT-AFF VL/masks/04_00000130.png]

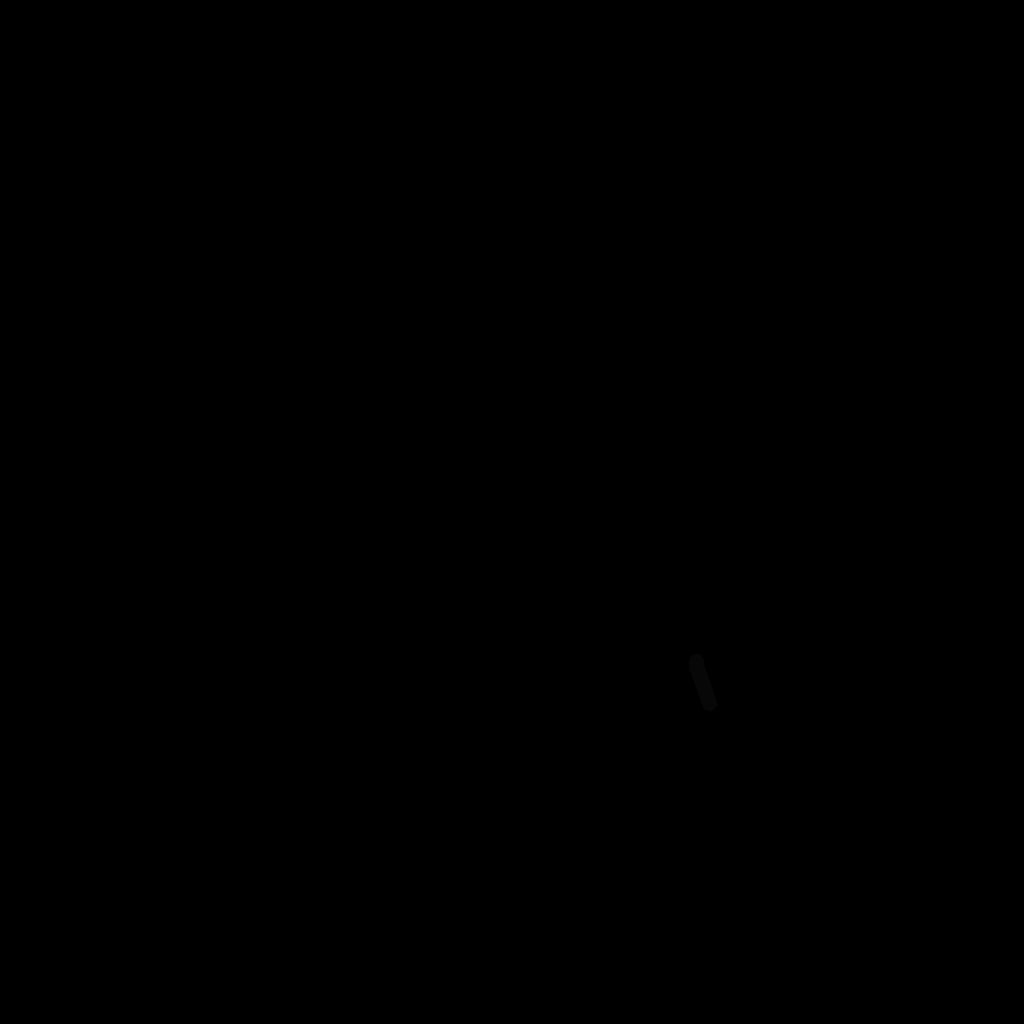

Supplement: Supplemental Information 1 [file peerj-cs-10-2097-s001.zip › IIT-AFF VL/masks/04_00000134.png]

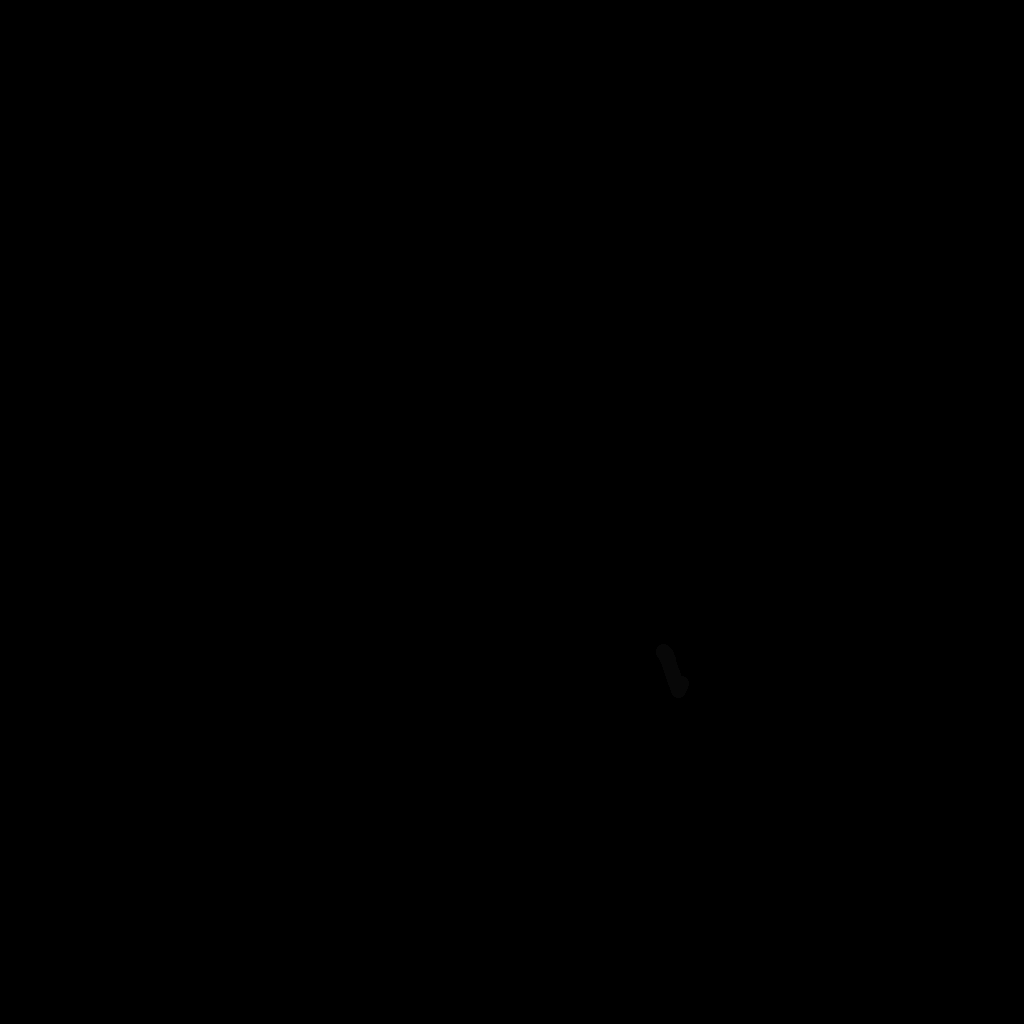

Supplement: Supplemental Information 1 [file peerj-cs-10-2097-s001.zip › IIT-AFF VL/masks/04_00000138.png]

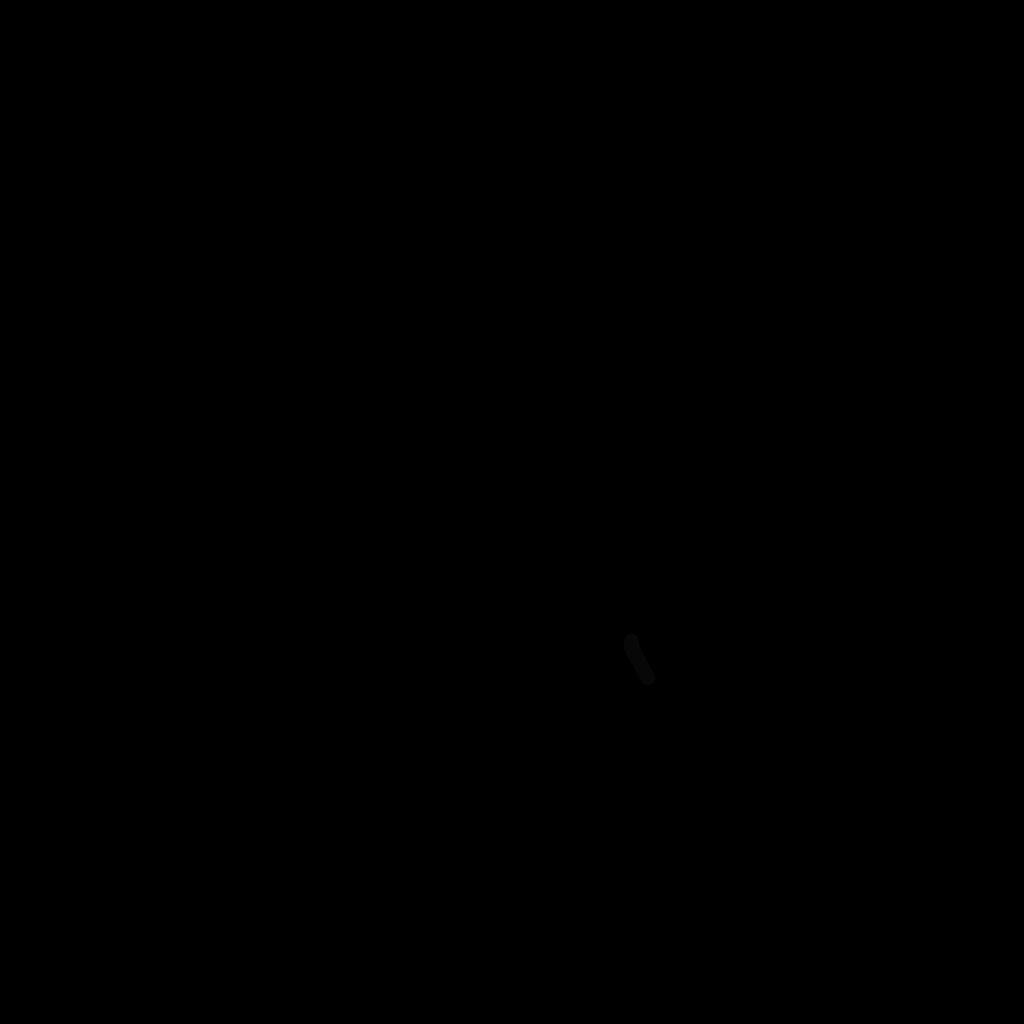

Supplement: Supplemental Information 1 [file peerj-cs-10-2097-s001.zip › IIT-AFF VL/masks/04_00000142.png]

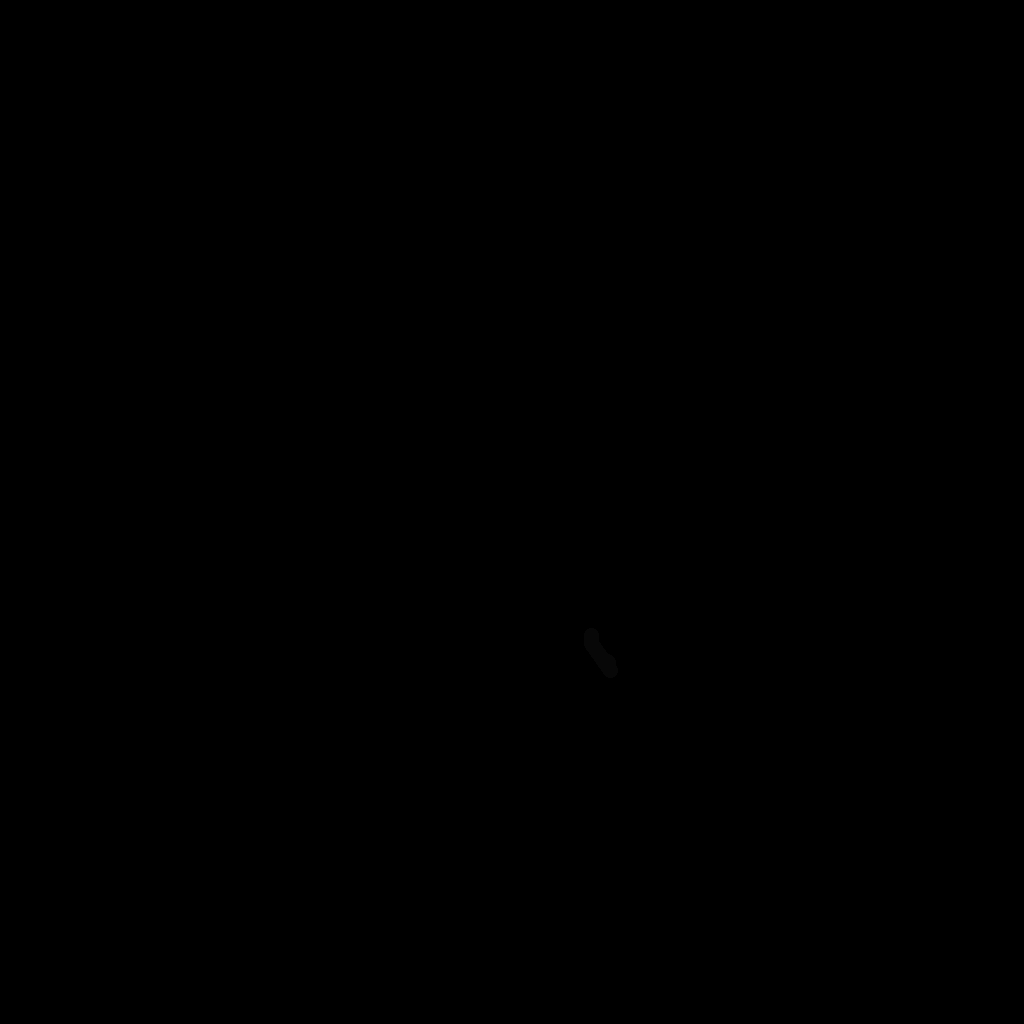

Supplement: Supplemental Information 1 [file peerj-cs-10-2097-s001.zip › IIT-AFF VL/masks/04_00000146.png]

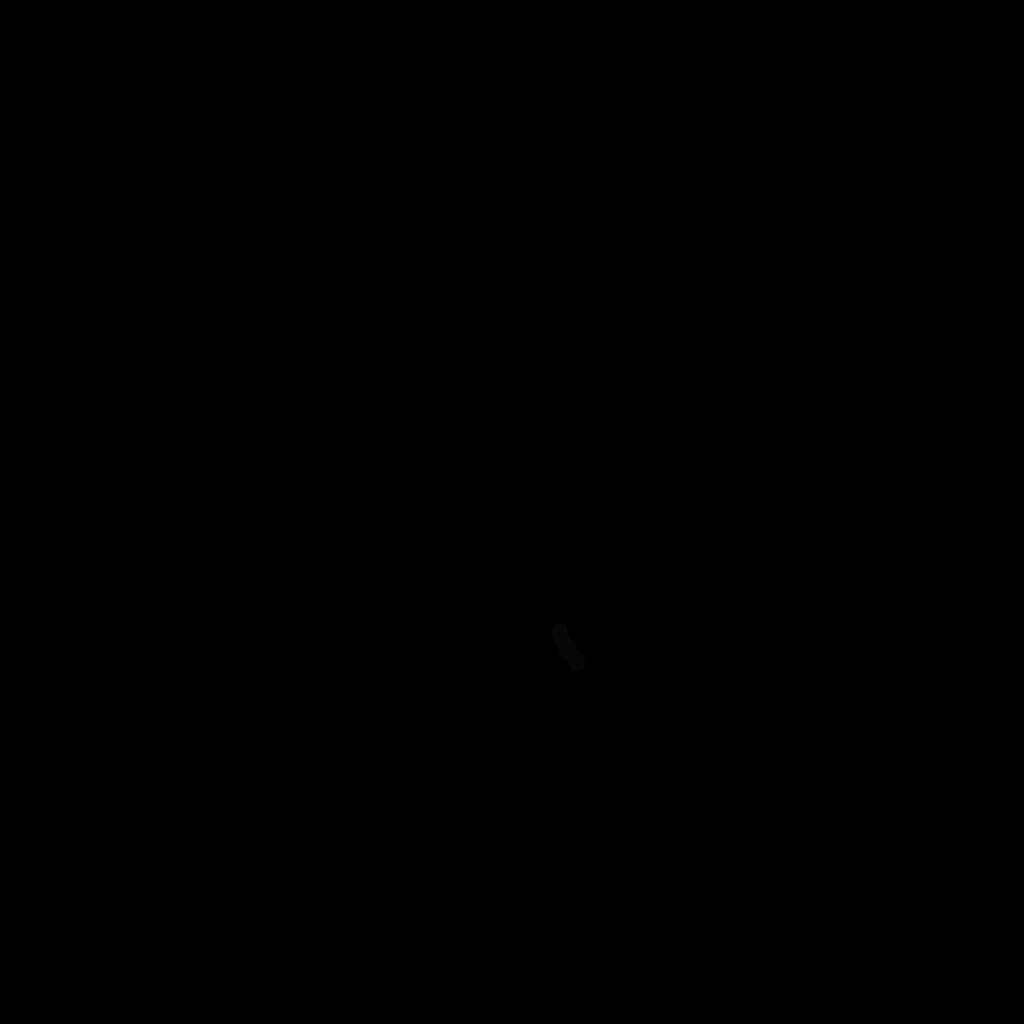

Supplement: Supplemental Information 1 [file peerj-cs-10-2097-s001.zip › IIT-AFF VL/masks/04_00000150.png]

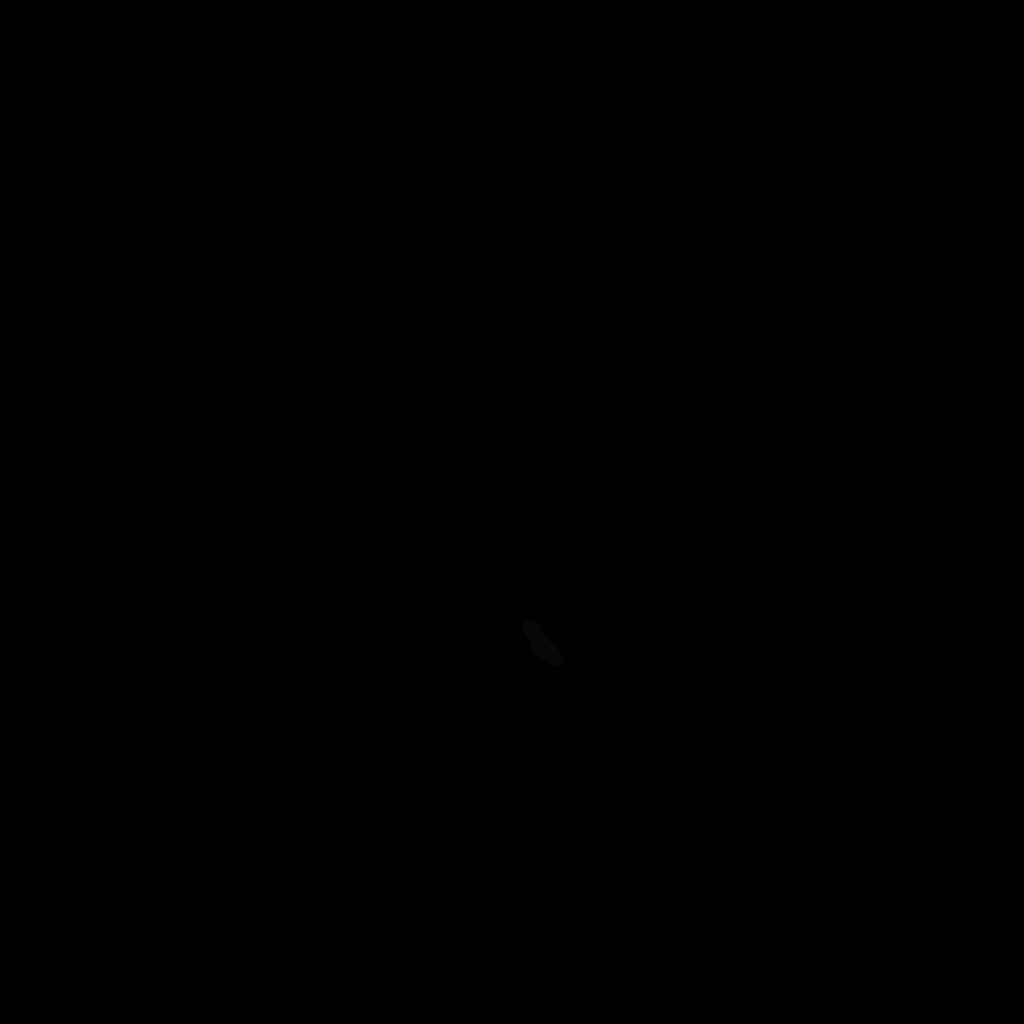

Supplement: Supplemental Information 1 [file peerj-cs-10-2097-s001.zip › IIT-AFF VL/masks/04_00000154.png]

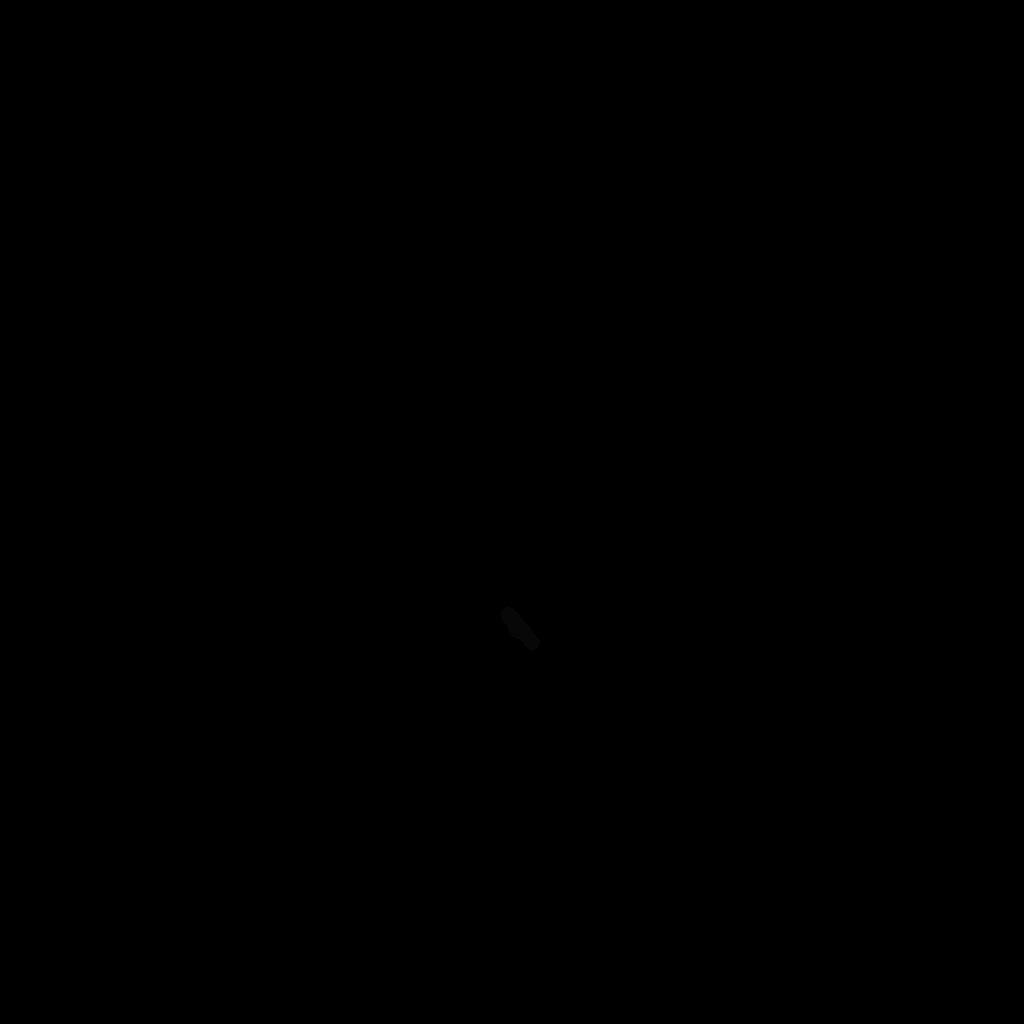

Supplement: Supplemental Information 1 [file peerj-cs-10-2097-s001.zip › IIT-AFF VL/masks/04_00000159.png]

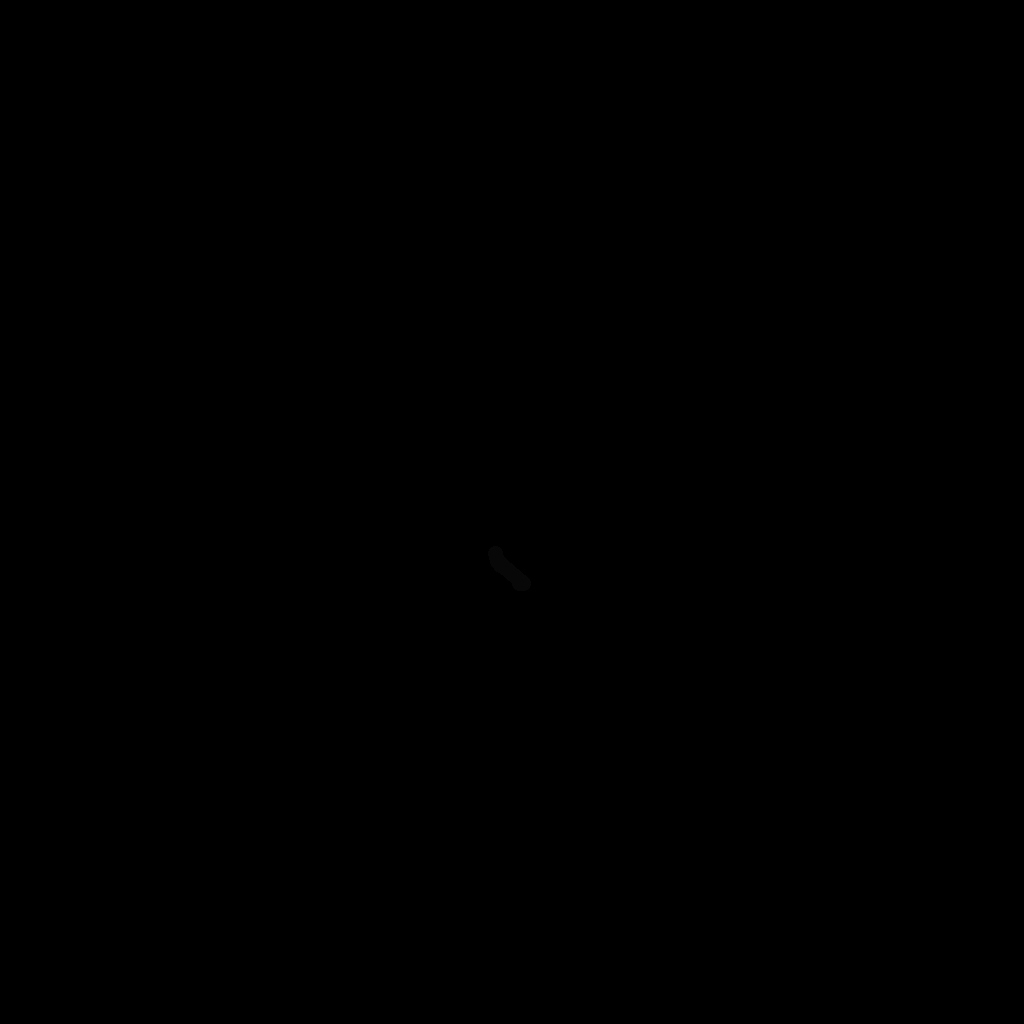

Supplement: Supplemental Information 1 [file peerj-cs-10-2097-s001.zip › IIT-AFF VL/masks/04_00000163.png]

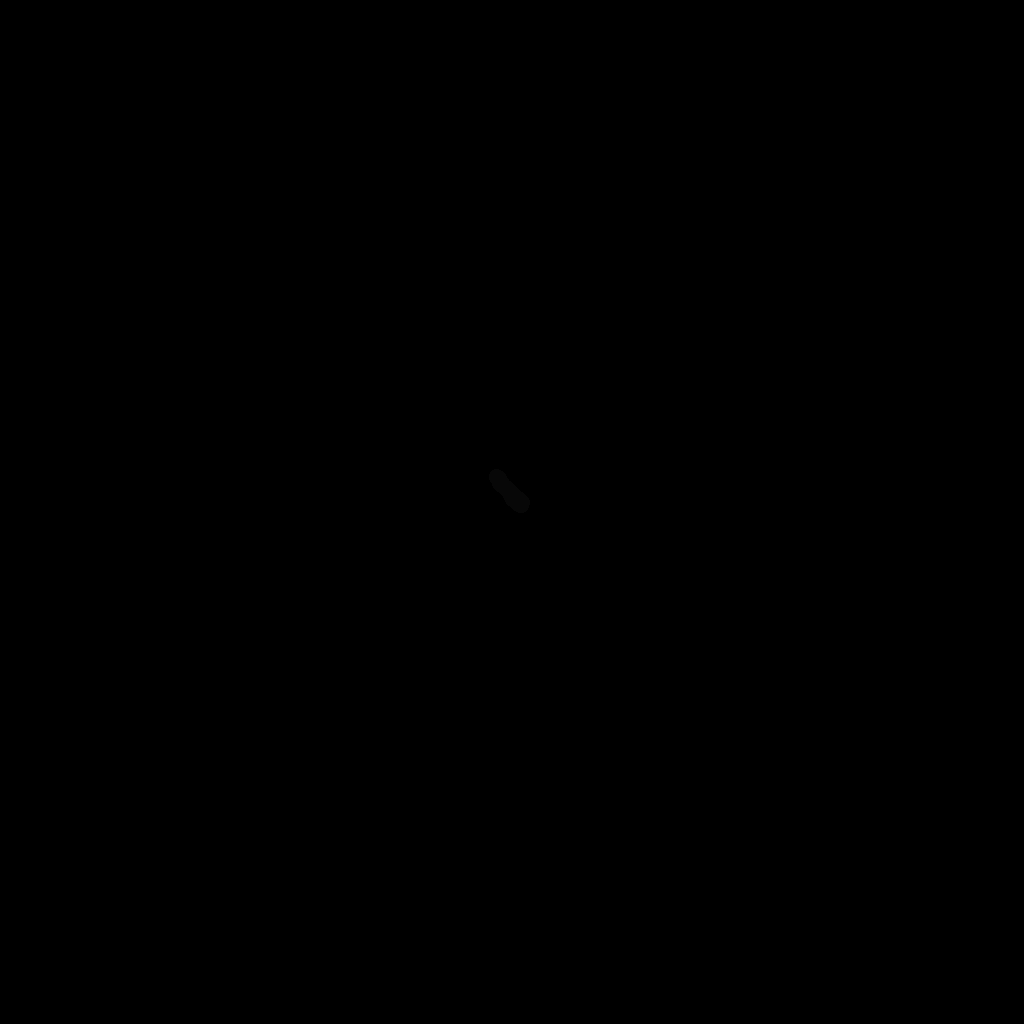

Supplement: Supplemental Information 1 [file peerj-cs-10-2097-s001.zip › IIT-AFF VL/masks/04_00000167.png]

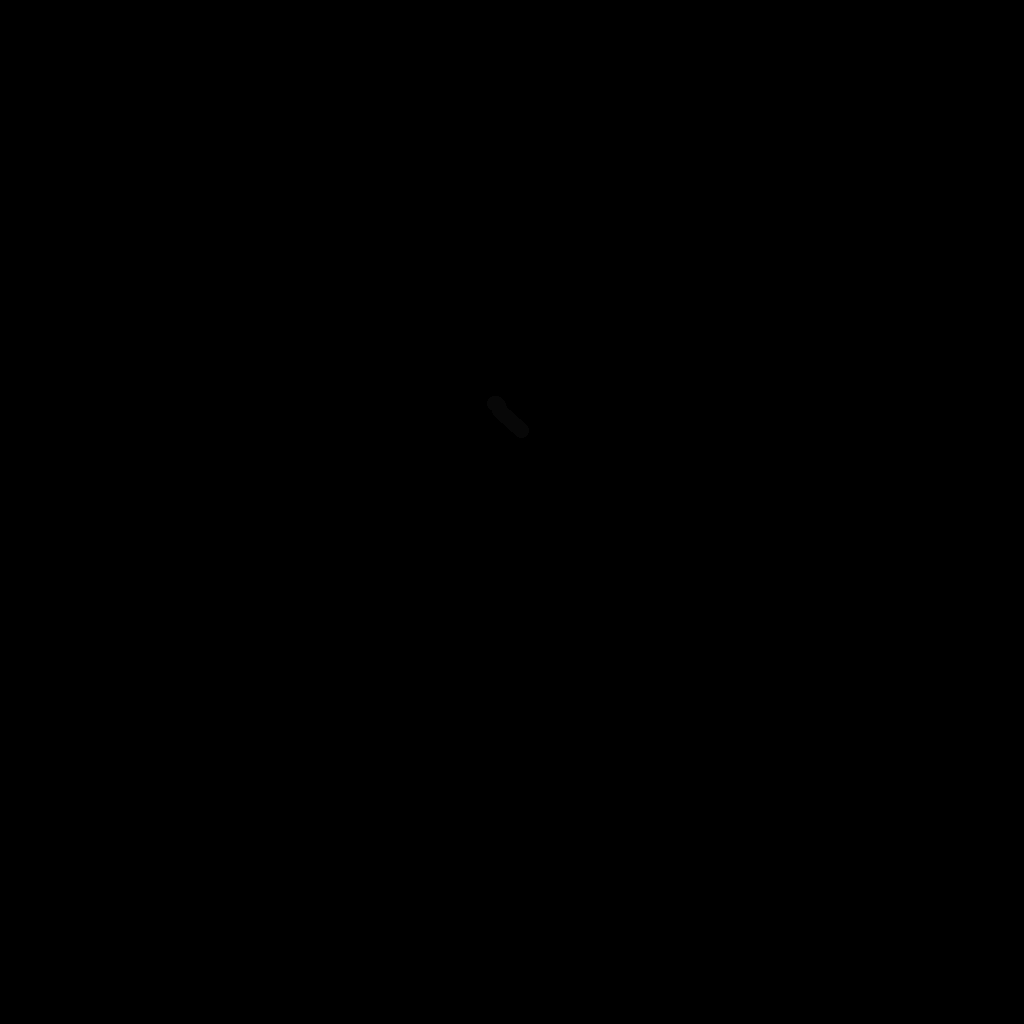

Supplement: Supplemental Information 1 [file peerj-cs-10-2097-s001.zip › IIT-AFF VL/masks/04_00000171.png]

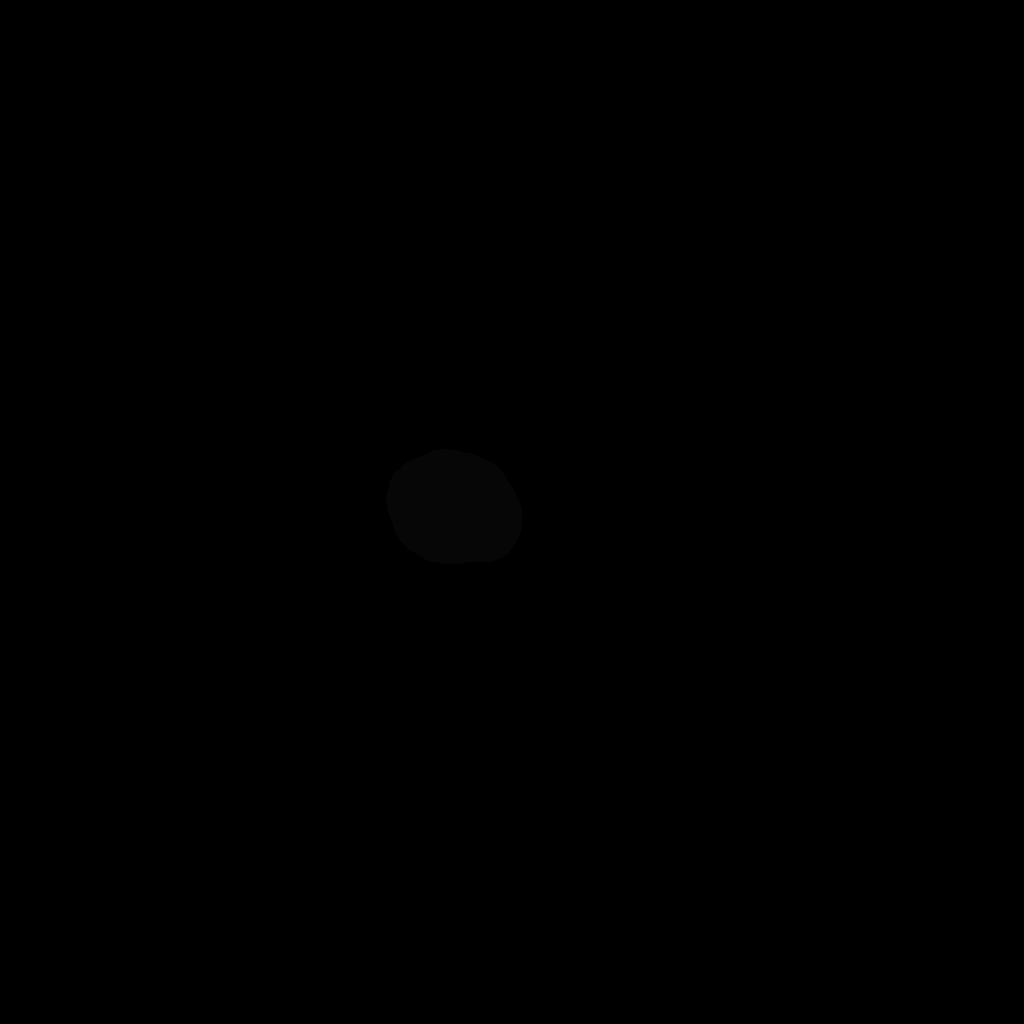

Supplement: Supplemental Information 1 [file peerj-cs-10-2097-s001.zip › IIT-AFF VL/masks/04_00000348.png]

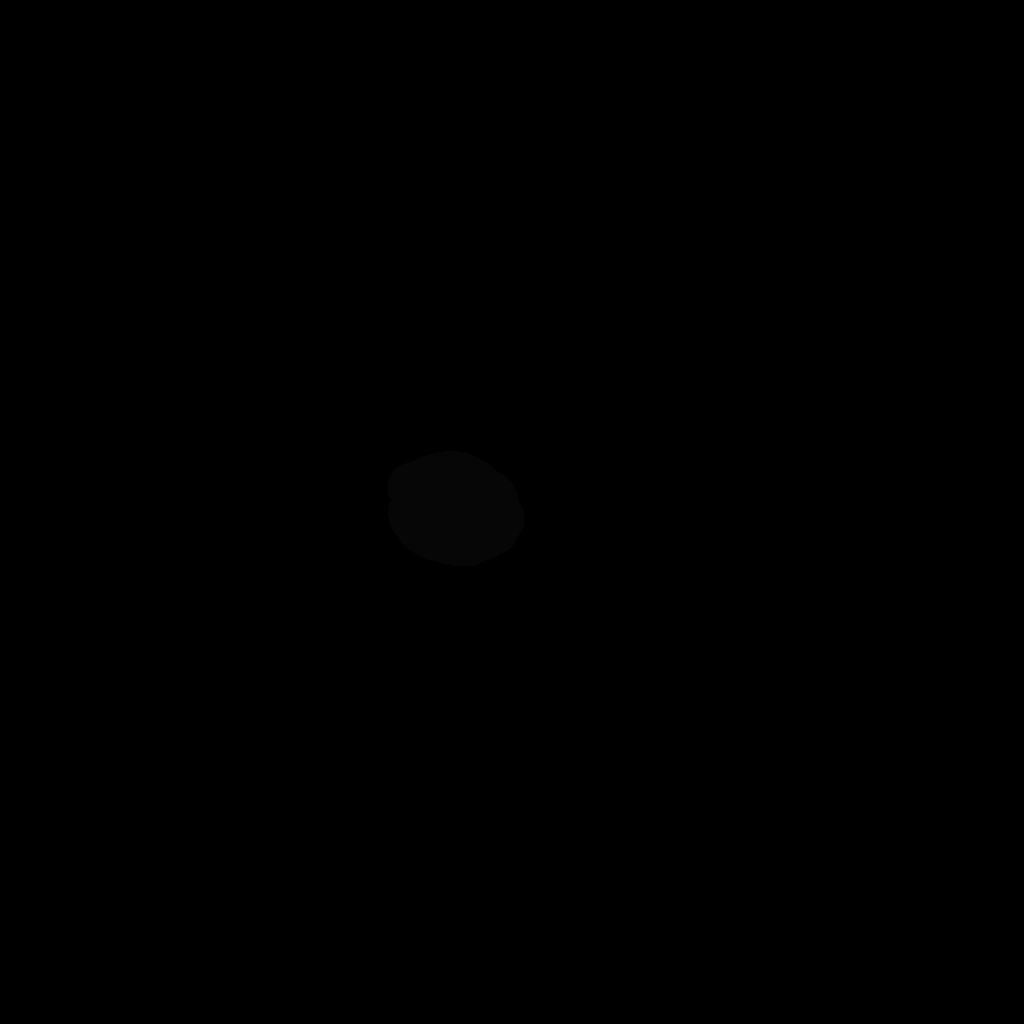

Supplement: Supplemental Information 1 [file peerj-cs-10-2097-s001.zip › IIT-AFF VL/masks/04_00000352.png]

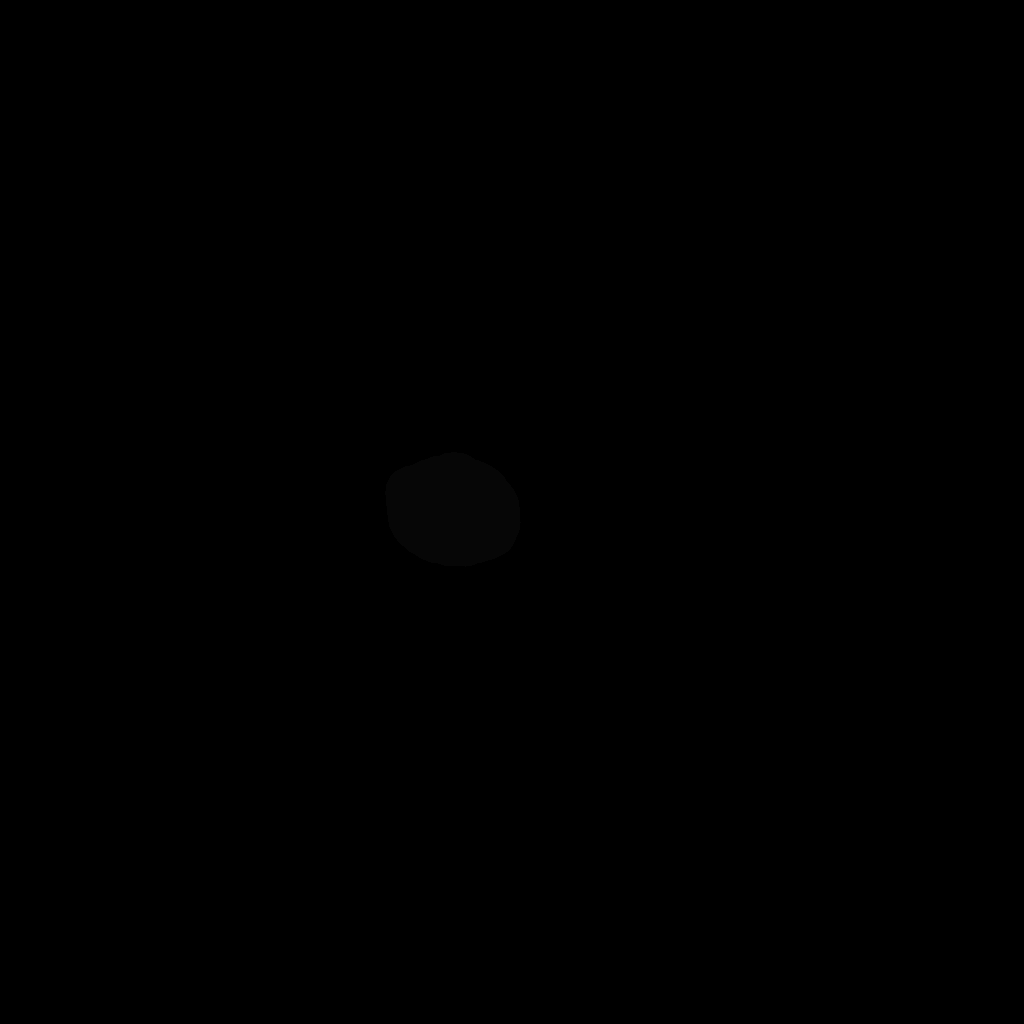

Supplement: Supplemental Information 1 [file peerj-cs-10-2097-s001.zip › IIT-AFF VL/masks/04_00000356.png]

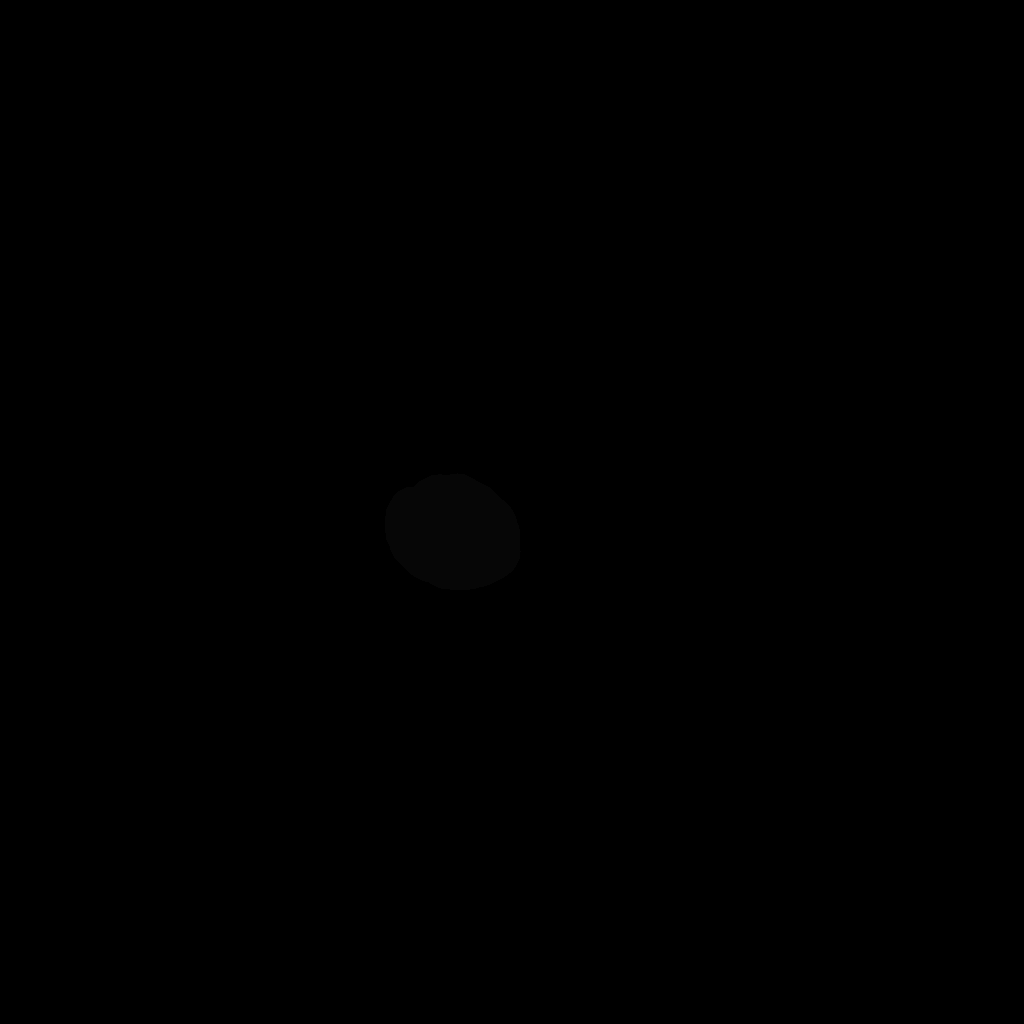

Supplement: Supplemental Information 1 [file peerj-cs-10-2097-s001.zip › IIT-AFF VL/masks/04_00000359.png]

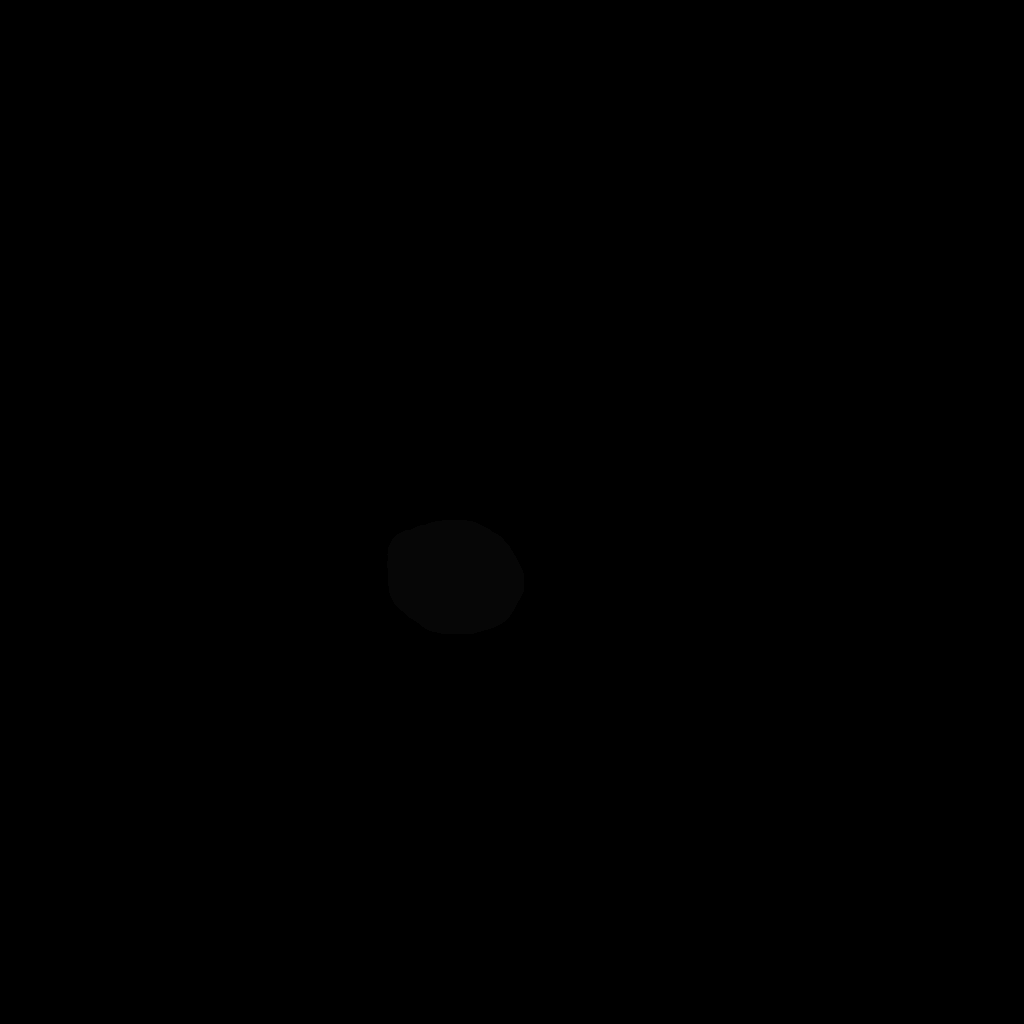

Supplement: Supplemental Information 1 [file peerj-cs-10-2097-s001.zip › IIT-AFF VL/masks/04_00000363.png]

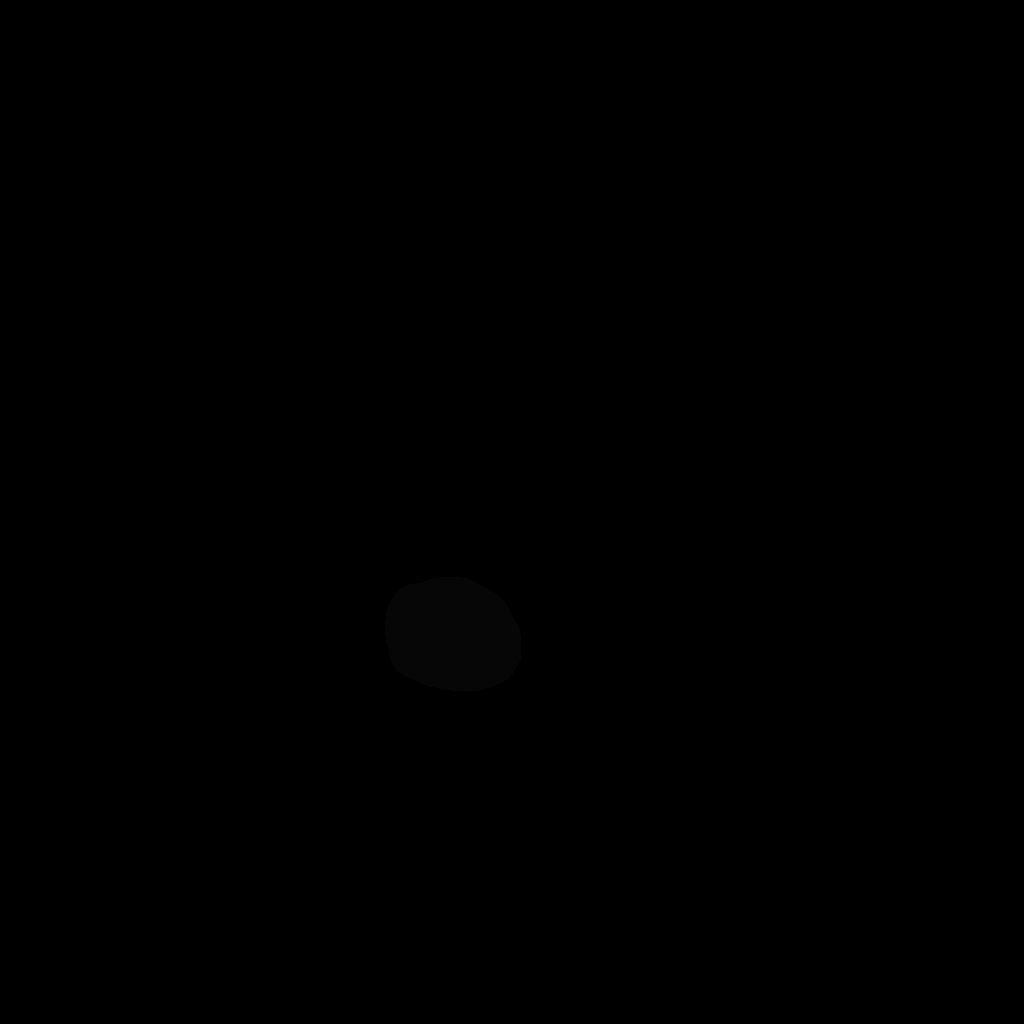

Supplement: Supplemental Information 1 [file peerj-cs-10-2097-s001.zip › IIT-AFF VL/masks/04_00000367.png]

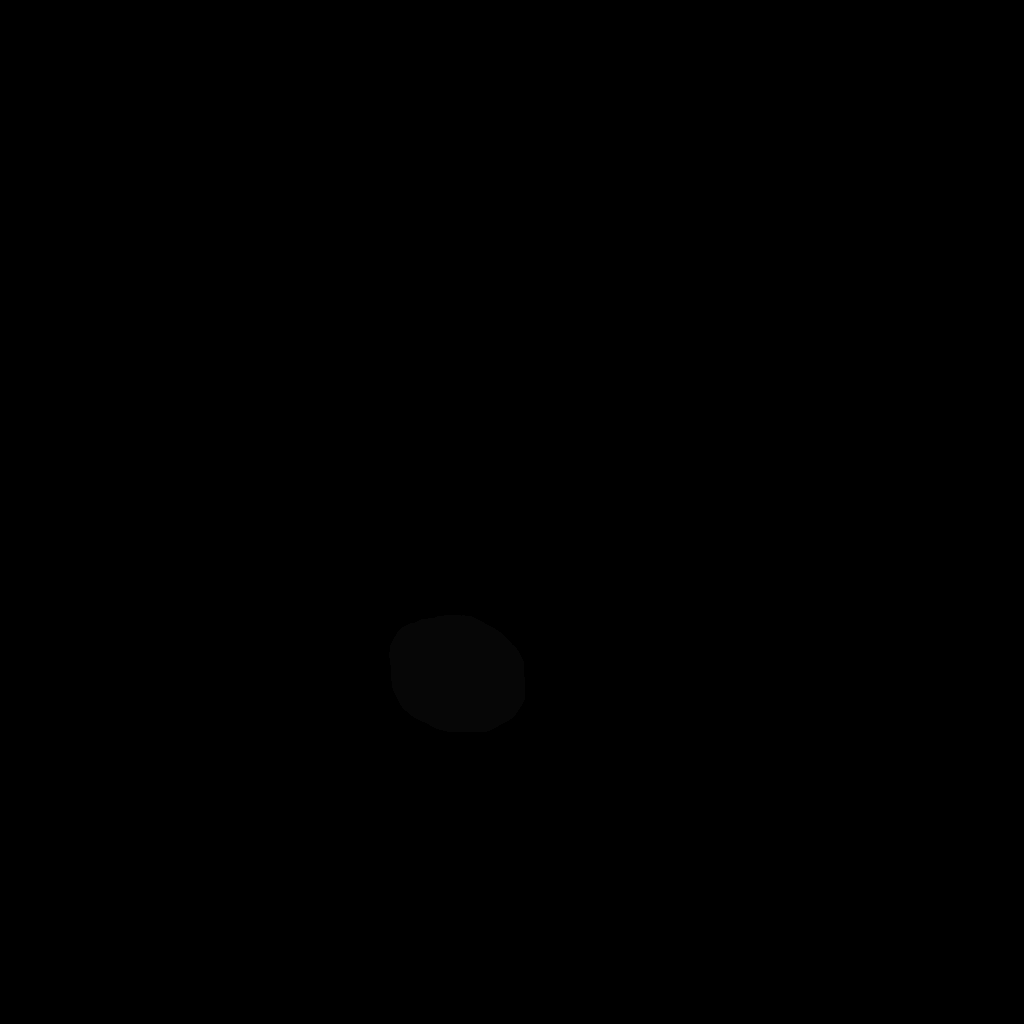

Supplement: Supplemental Information 1 [file peerj-cs-10-2097-s001.zip › IIT-AFF VL/masks/04_00000371.png]

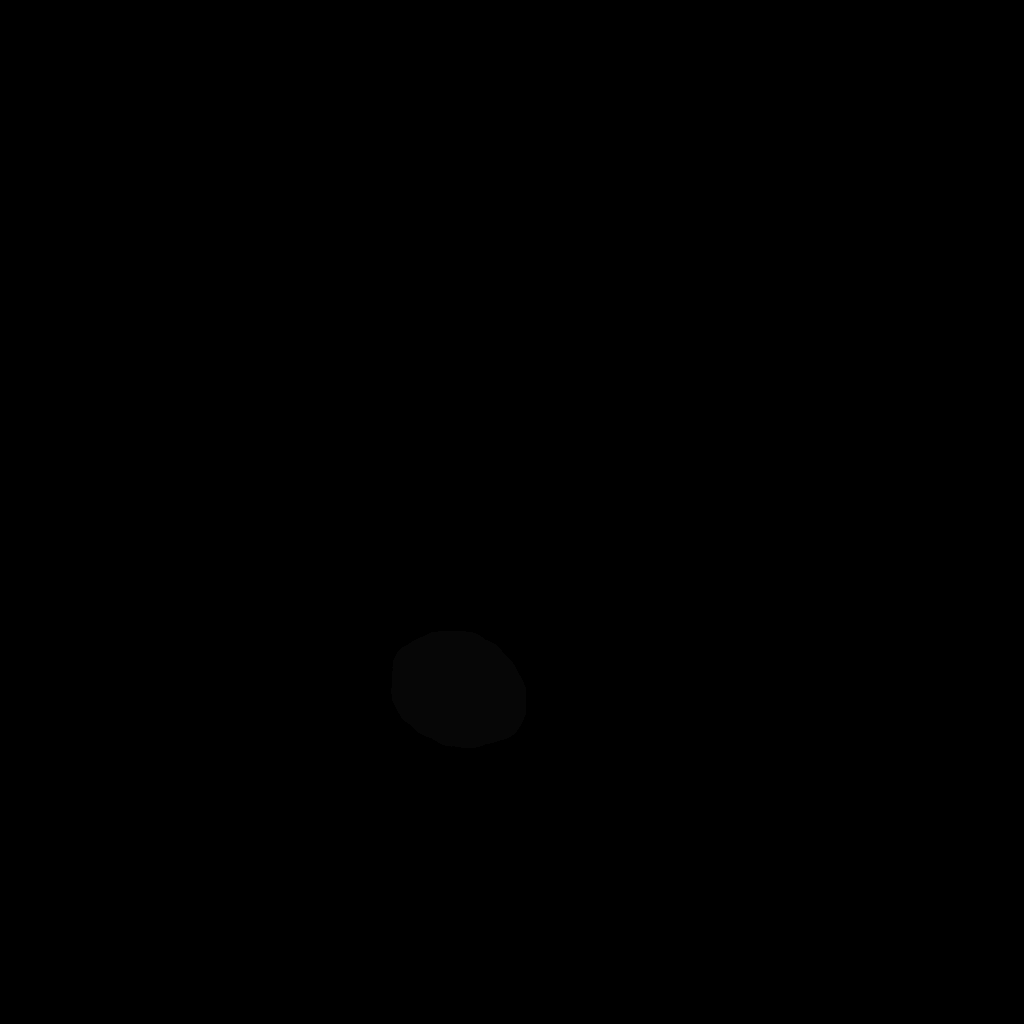

Supplement: Supplemental Information 1 [file peerj-cs-10-2097-s001.zip › IIT-AFF VL/masks/04_00000376.png]

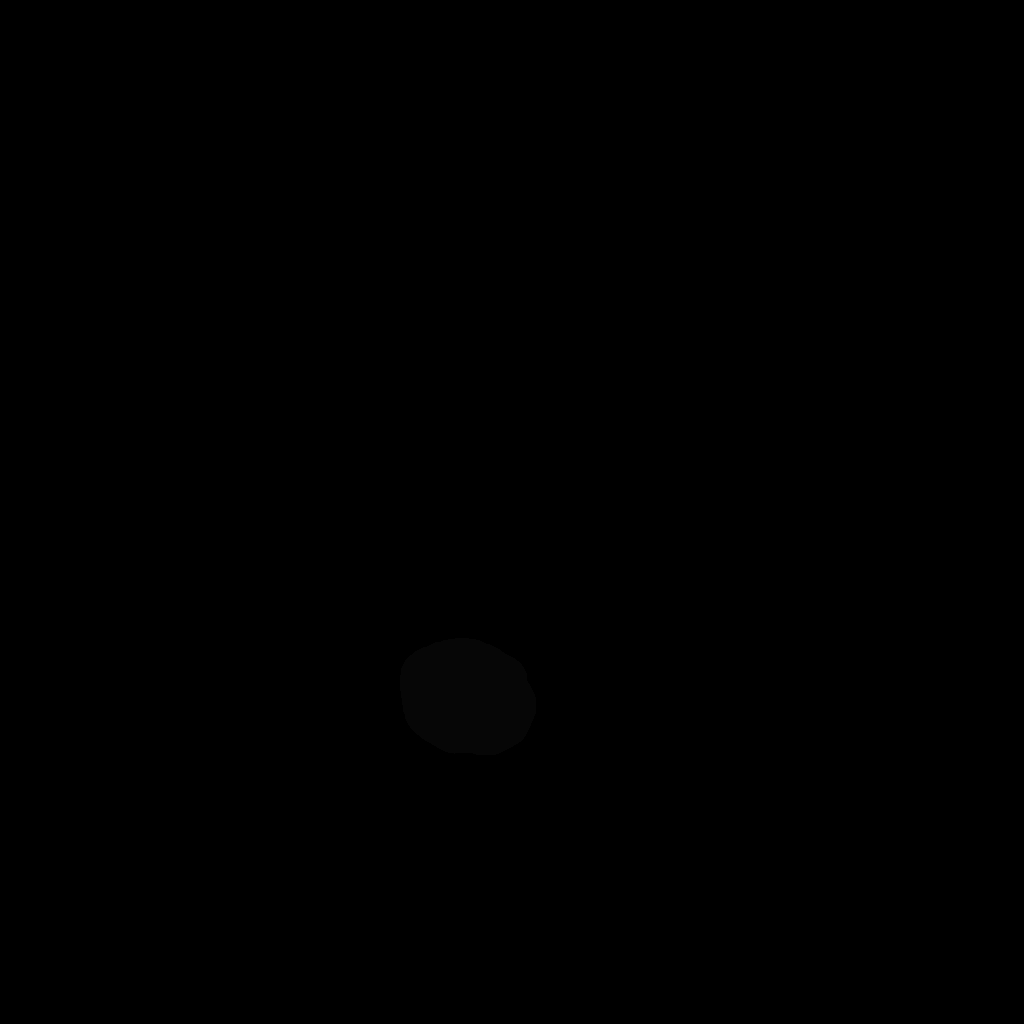

Supplement: Supplemental Information 1 [file peerj-cs-10-2097-s001.zip › IIT-AFF VL/masks/04_00000380.png]

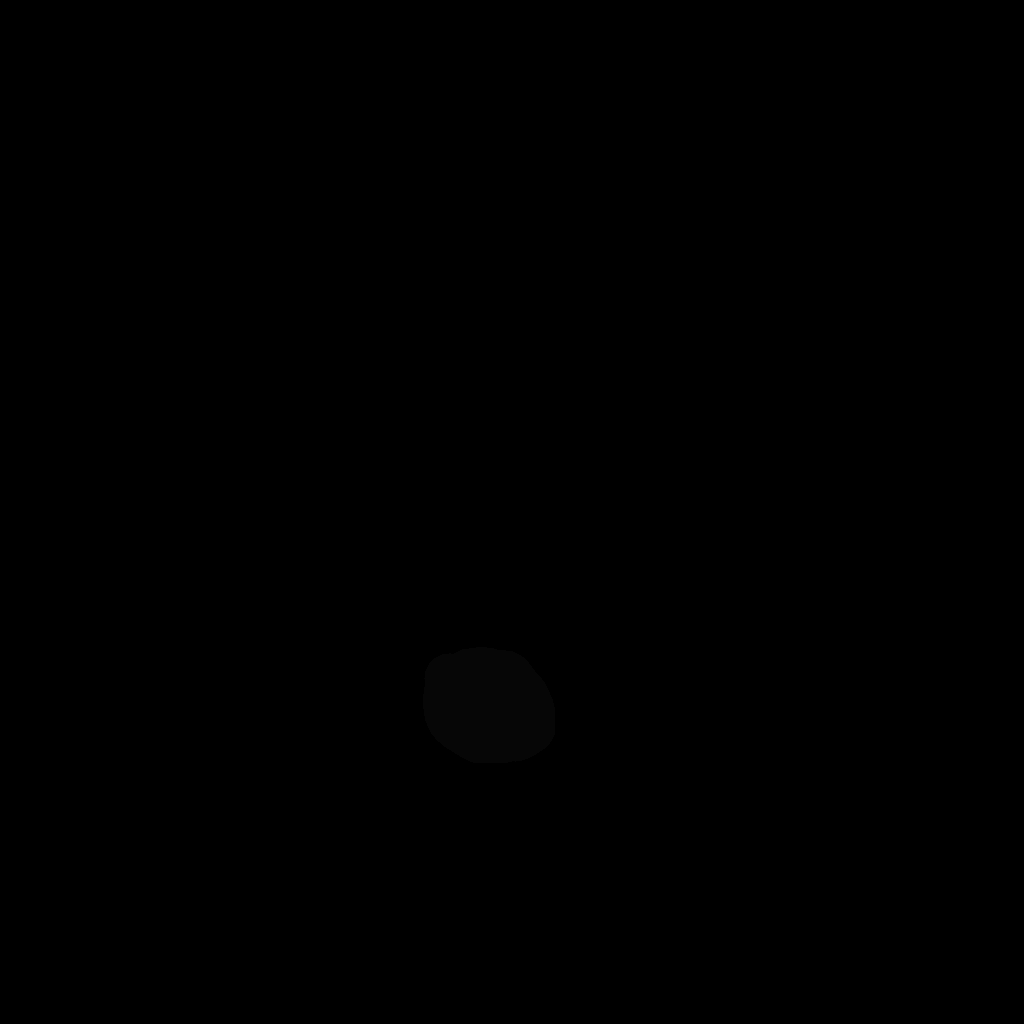

Supplement: Supplemental Information 1 [file peerj-cs-10-2097-s001.zip › IIT-AFF VL/masks/04_00000384.png]

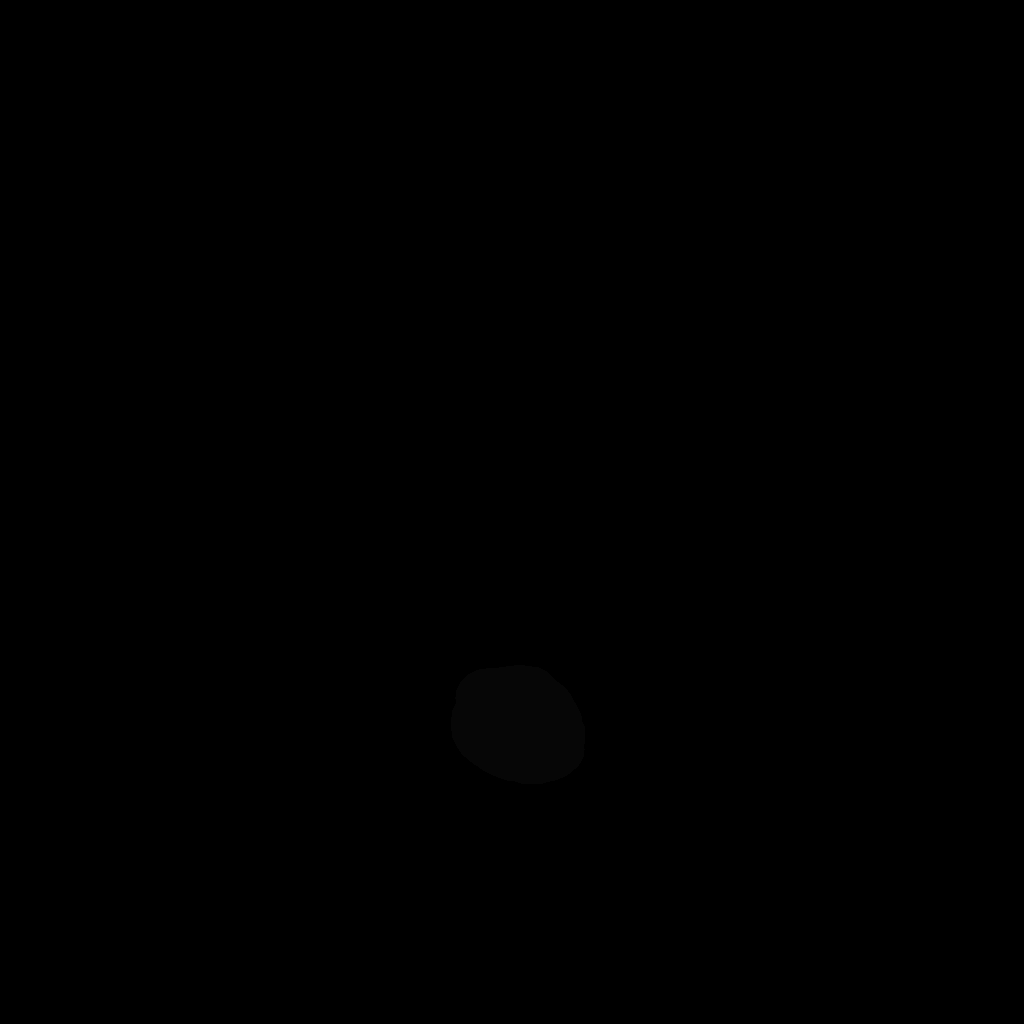

Supplement: Supplemental Information 1 [file peerj-cs-10-2097-s001.zip › IIT-AFF VL/masks/04_00000388.png]

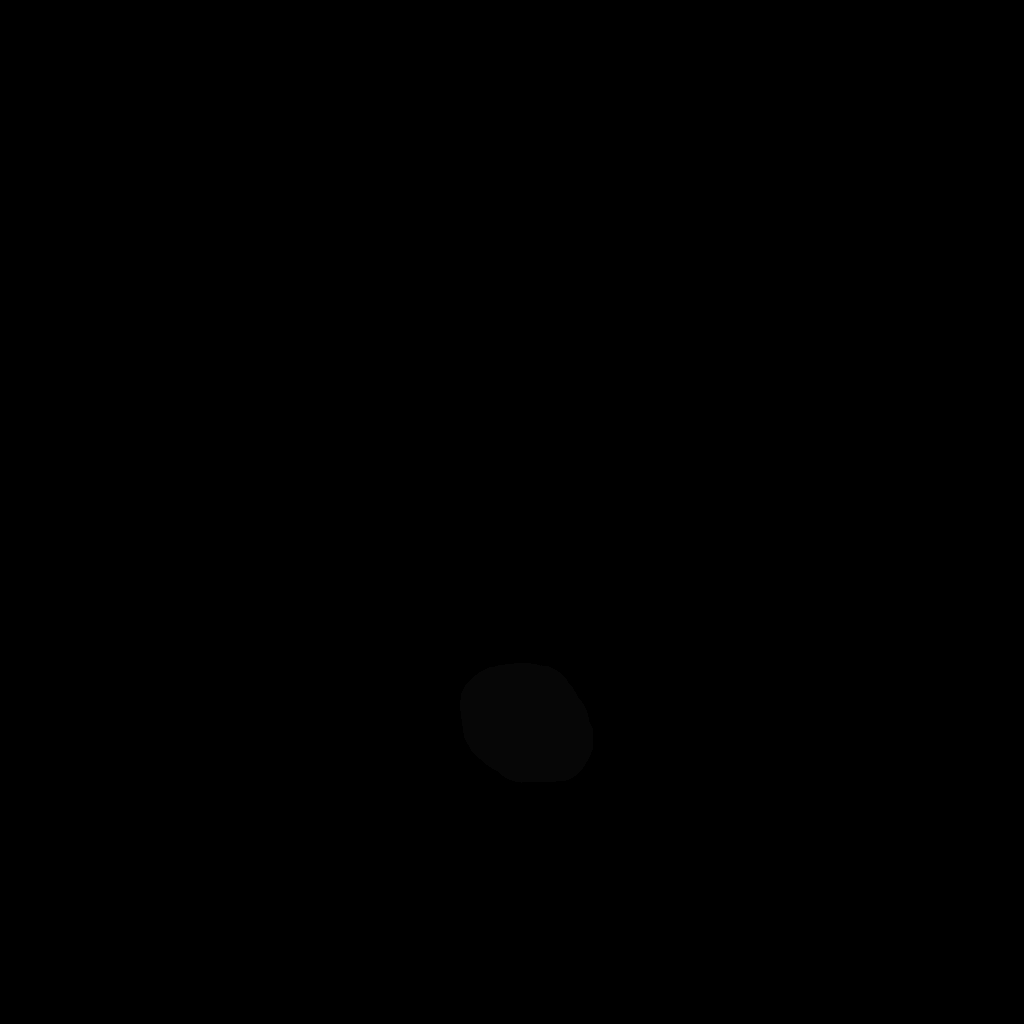

Supplement: Supplemental Information 1 [file peerj-cs-10-2097-s001.zip › IIT-AFF VL/masks/04_00000392.png]

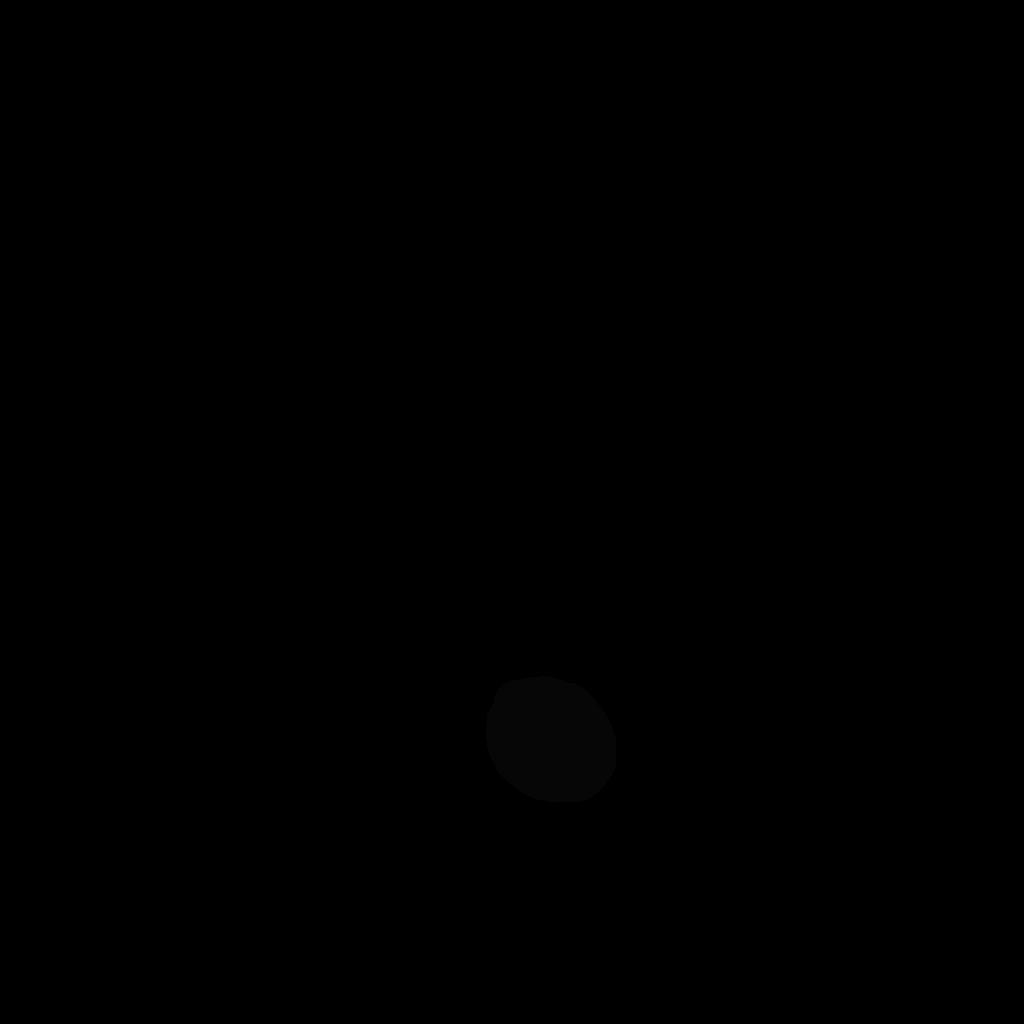

Supplement: Supplemental Information 1 [file peerj-cs-10-2097-s001.zip › IIT-AFF VL/masks/04_00000396.png]

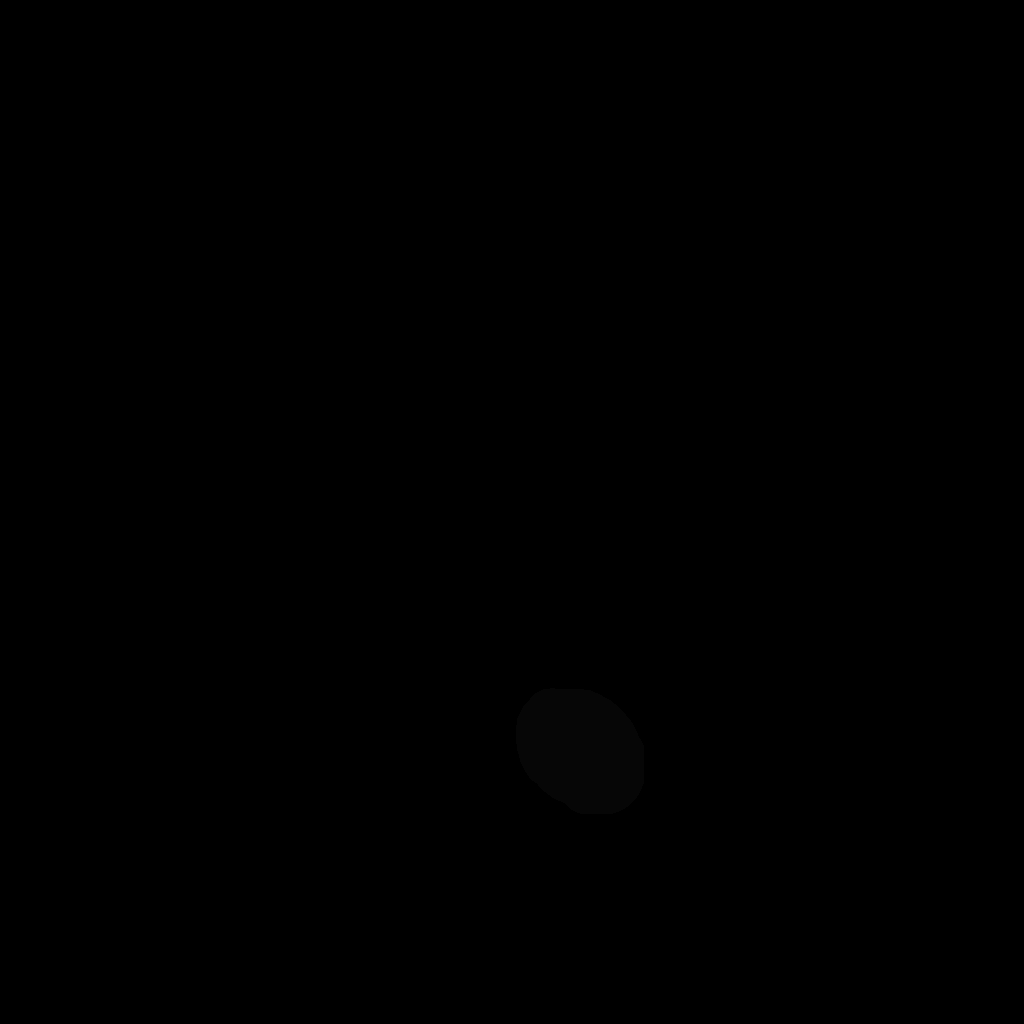

Supplement: Supplemental Information 1 [file peerj-cs-10-2097-s001.zip › IIT-AFF VL/masks/04_00000401.png]

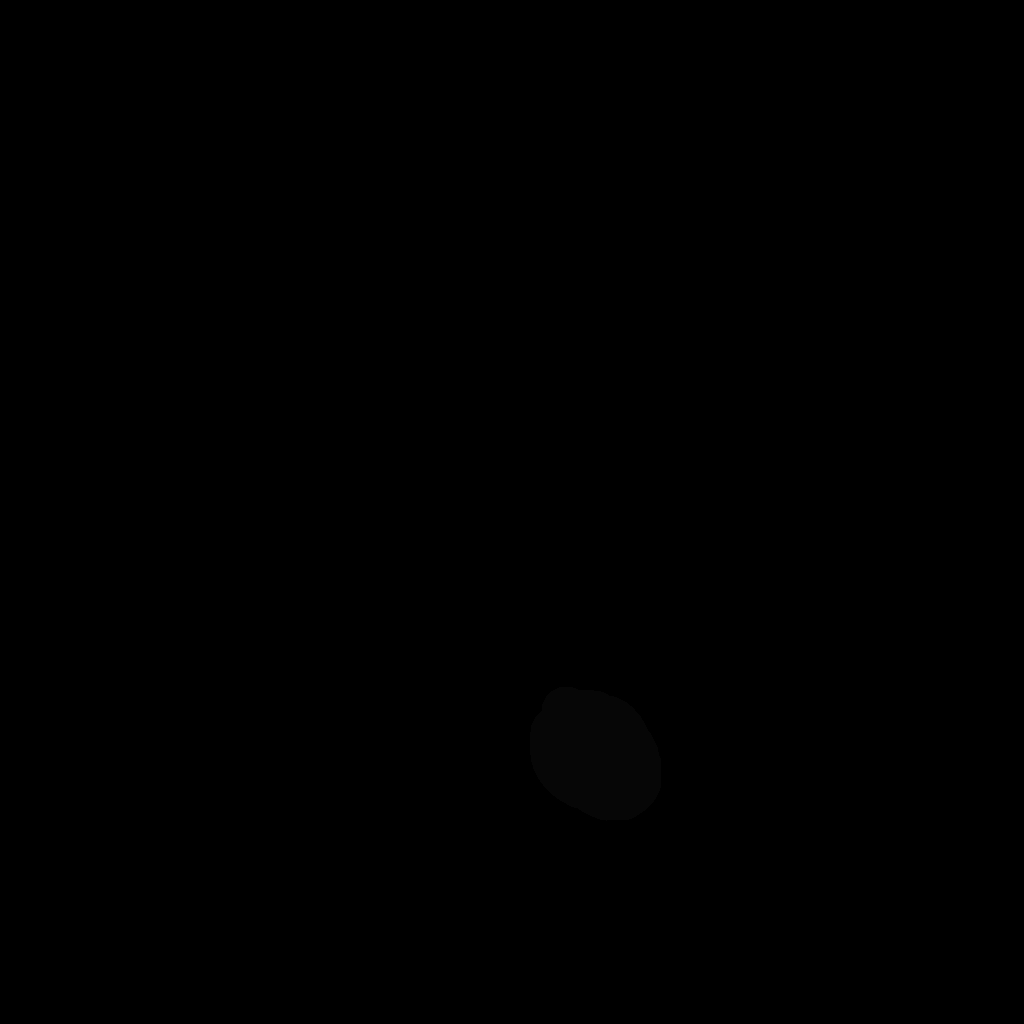

Supplement: Supplemental Information 1 [file peerj-cs-10-2097-s001.zip › IIT-AFF VL/masks/04_00000405.png]

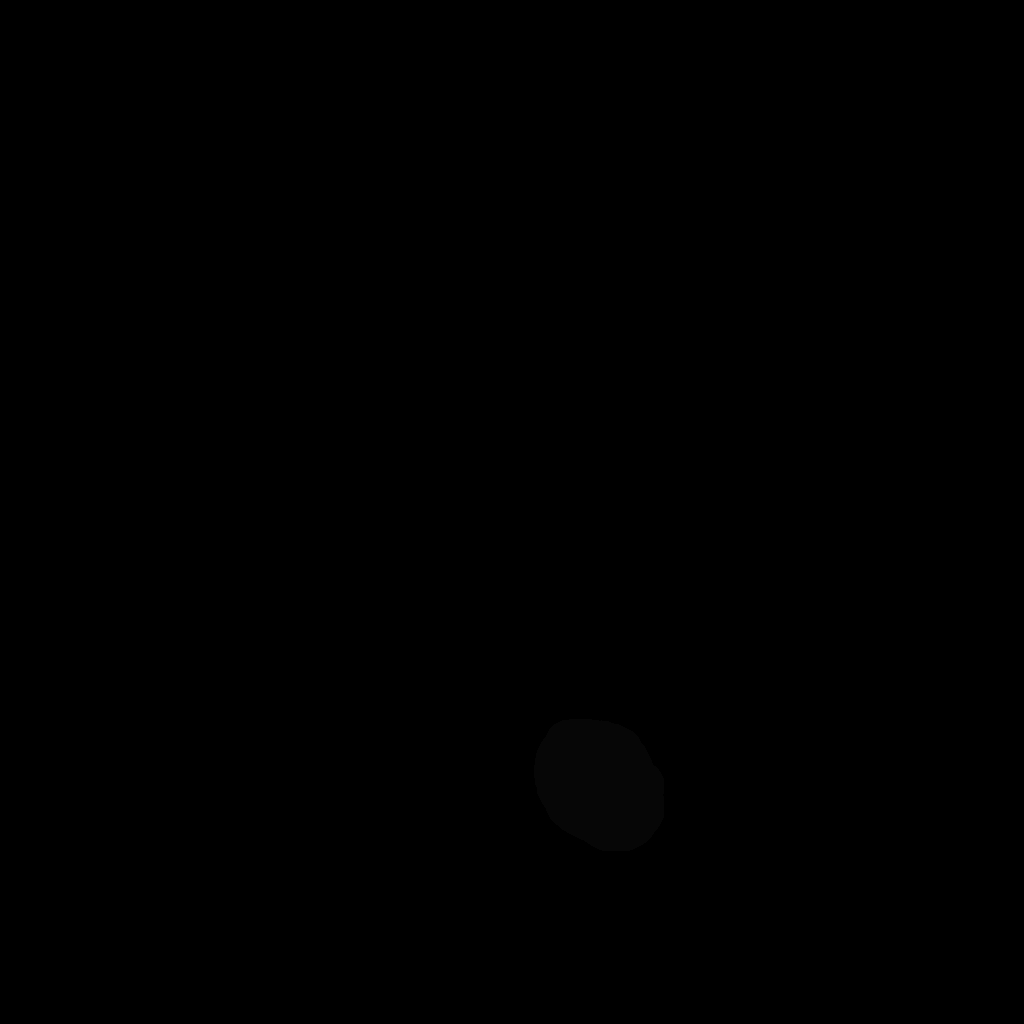

Supplement: Supplemental Information 1 [file peerj-cs-10-2097-s001.zip › IIT-AFF VL/masks/04_00000411.png]

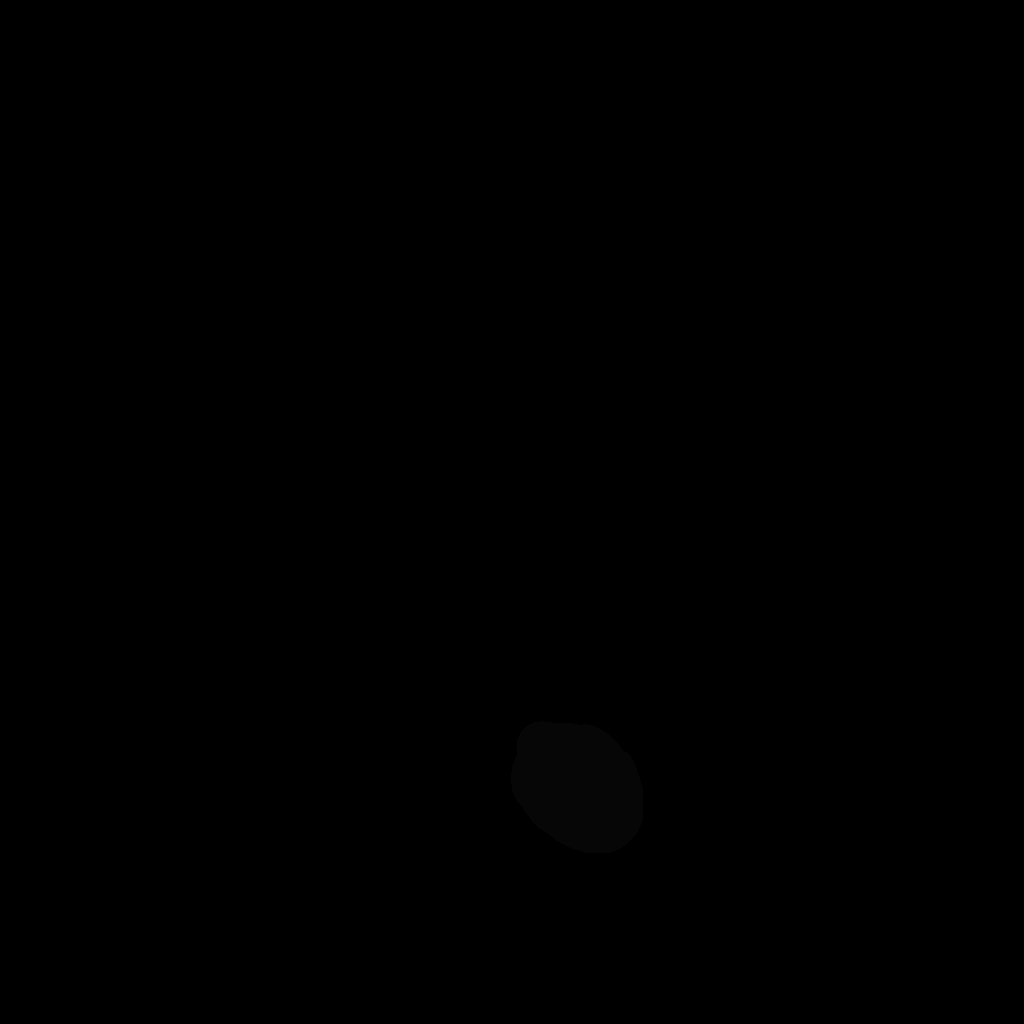

Supplement: Supplemental Information 1 [file peerj-cs-10-2097-s001.zip › IIT-AFF VL/masks/04_00000415.png]

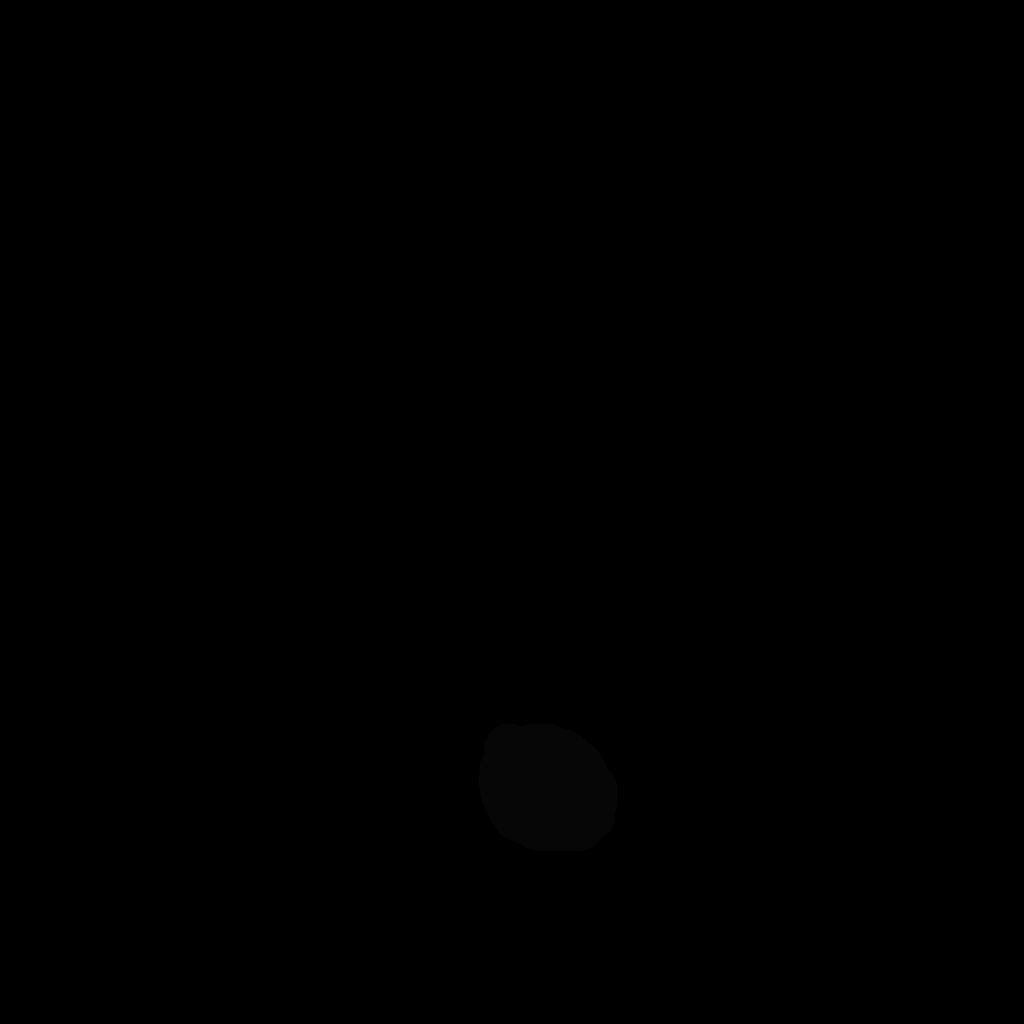

Supplement: Supplemental Information 1 [file peerj-cs-10-2097-s001.zip › IIT-AFF VL/masks/04_00000420.png]

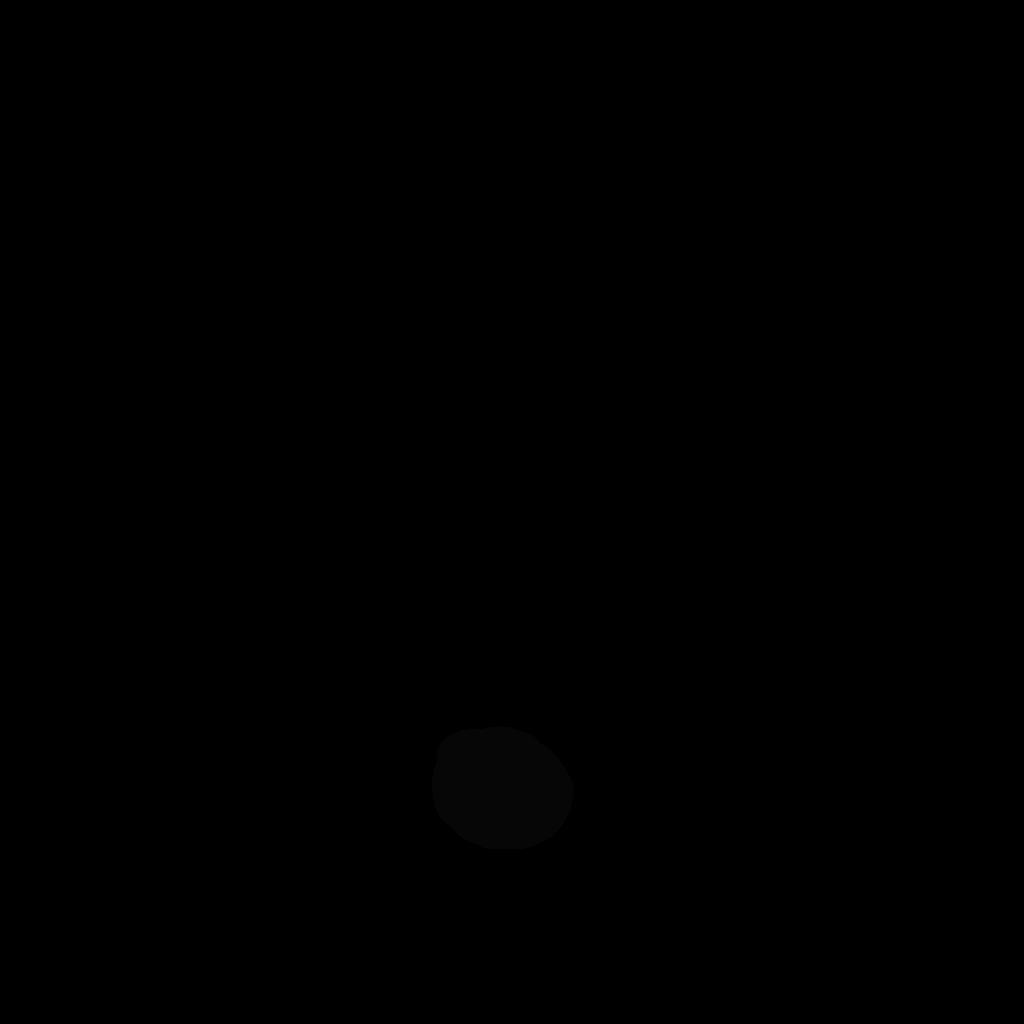

Supplement: Supplemental Information 1 [file peerj-cs-10-2097-s001.zip › IIT-AFF VL/masks/04_00000424.png]

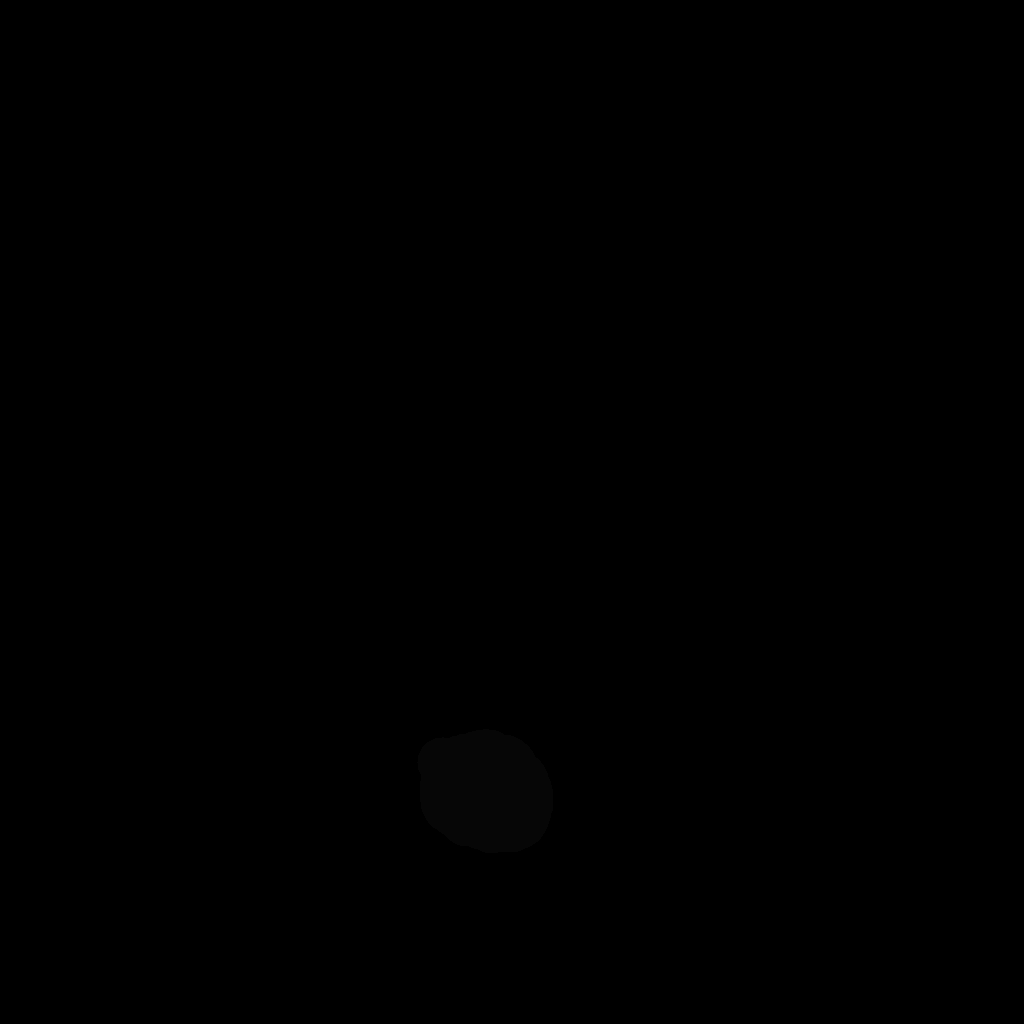

Supplement: Supplemental Information 1 [file peerj-cs-10-2097-s001.zip › IIT-AFF VL/masks/04_00000429.png]

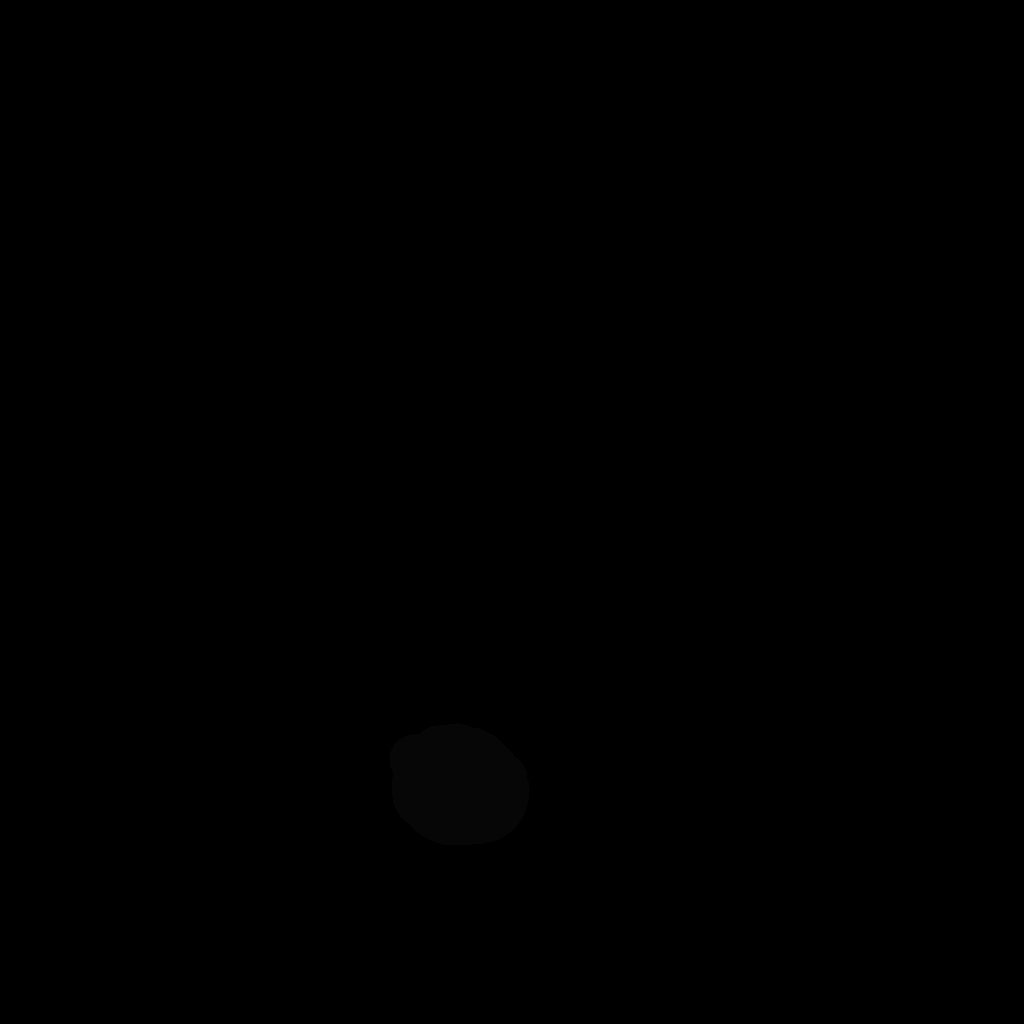

Supplement: Supplemental Information 1 [file peerj-cs-10-2097-s001.zip › IIT-AFF VL/masks/04_00000435.png]

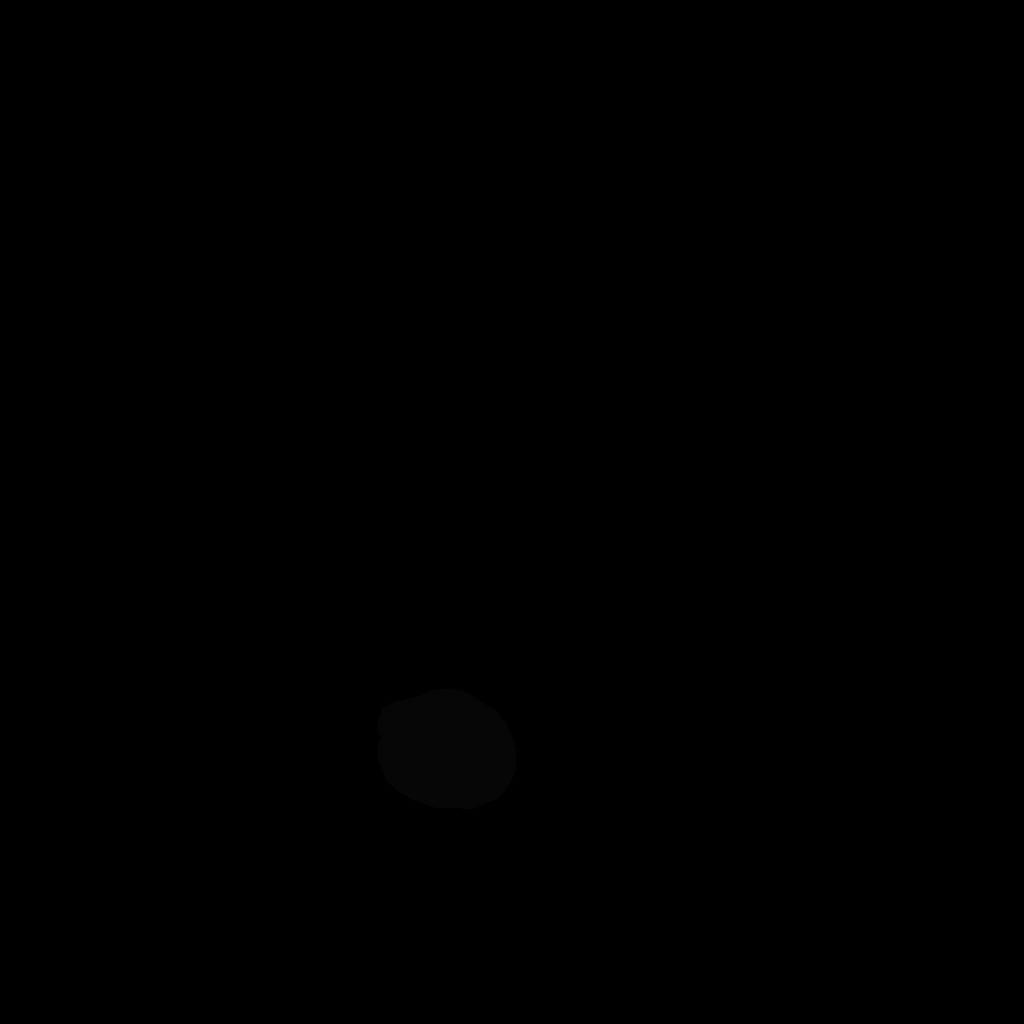

Supplement: Supplemental Information 1 [file peerj-cs-10-2097-s001.zip › IIT-AFF VL/masks/04_00000439.png]

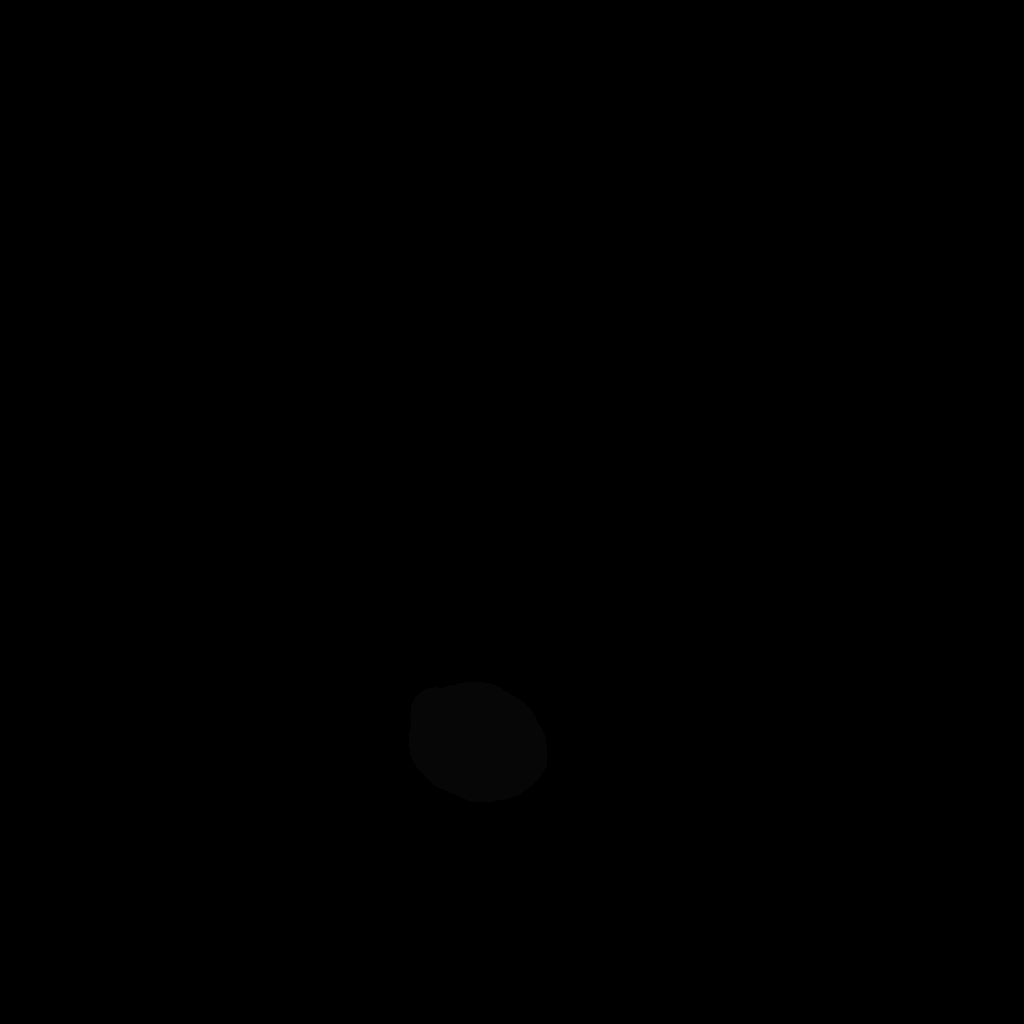

Supplement: Supplemental Information 1 [file peerj-cs-10-2097-s001.zip › IIT-AFF VL/masks/04_00000443.png]

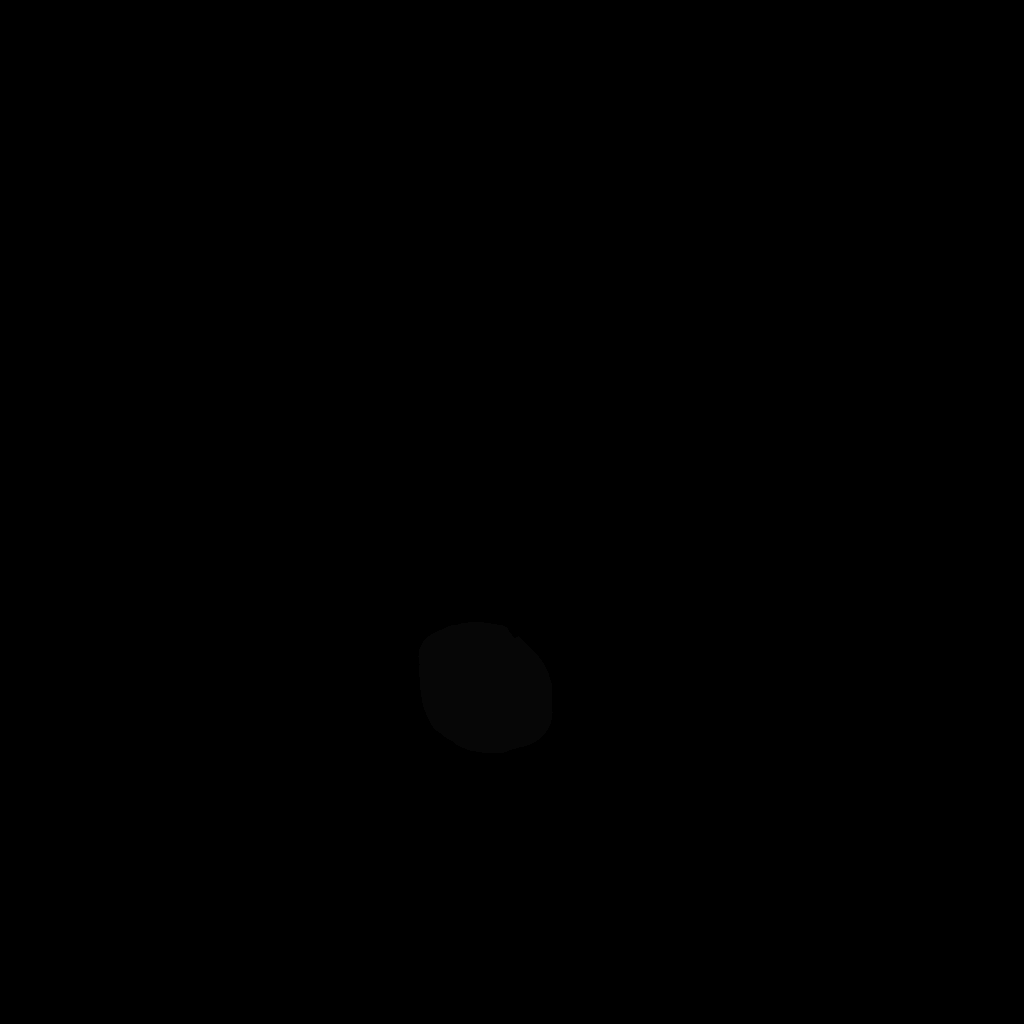

Supplement: Supplemental Information 1 [file peerj-cs-10-2097-s001.zip › IIT-AFF VL/masks/04_00000447.png]

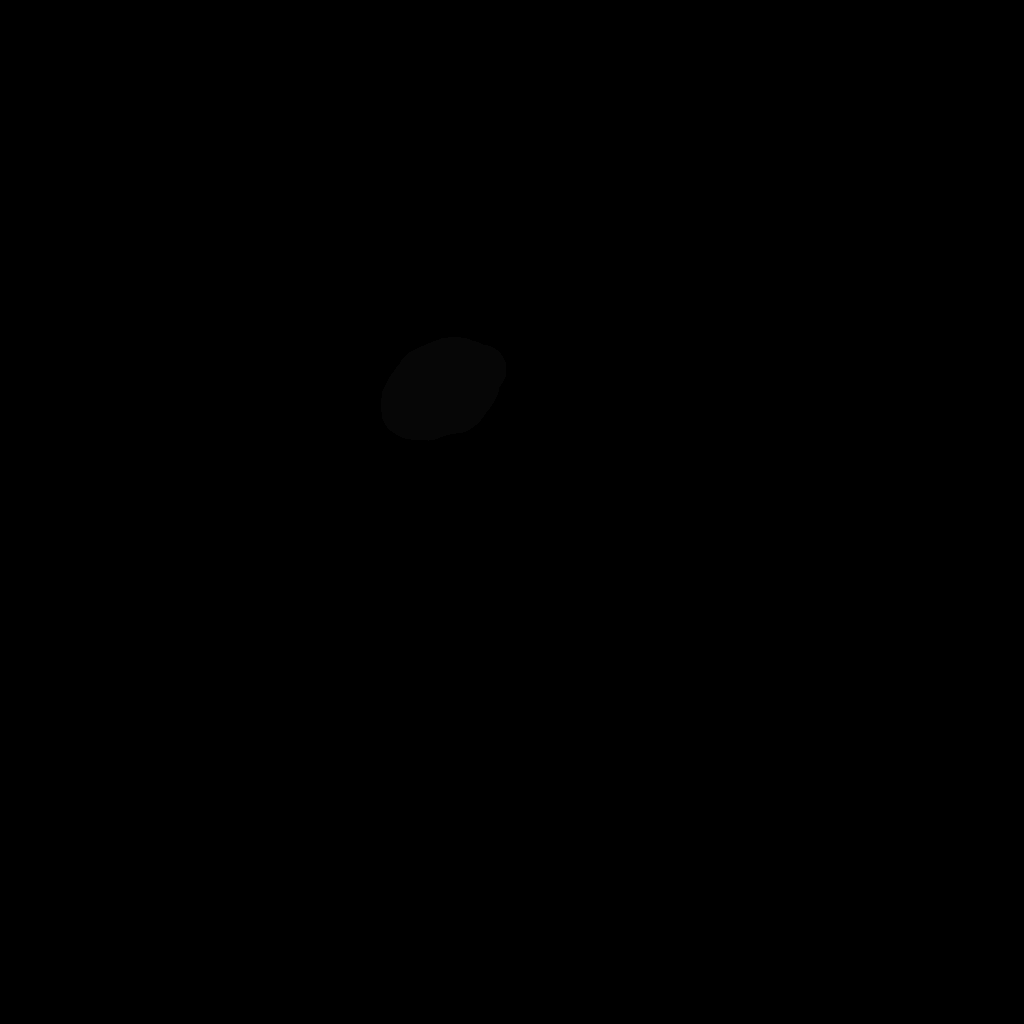

Supplement: Supplemental Information 1 [file peerj-cs-10-2097-s001.zip › IIT-AFF VL/masks/04_00000500.png]

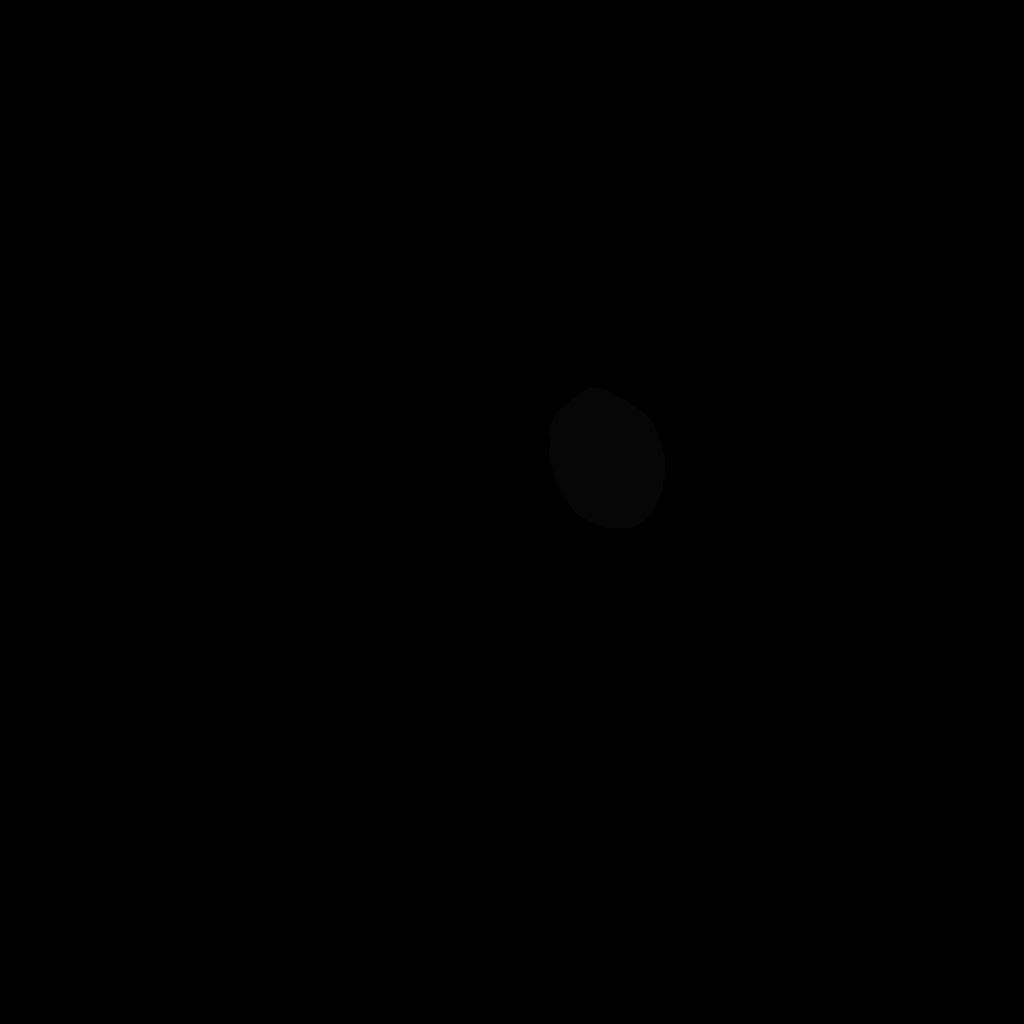

Supplement: Supplemental Information 1 [file peerj-cs-10-2097-s001.zip › IIT-AFF VL/masks/04_00000577.png]

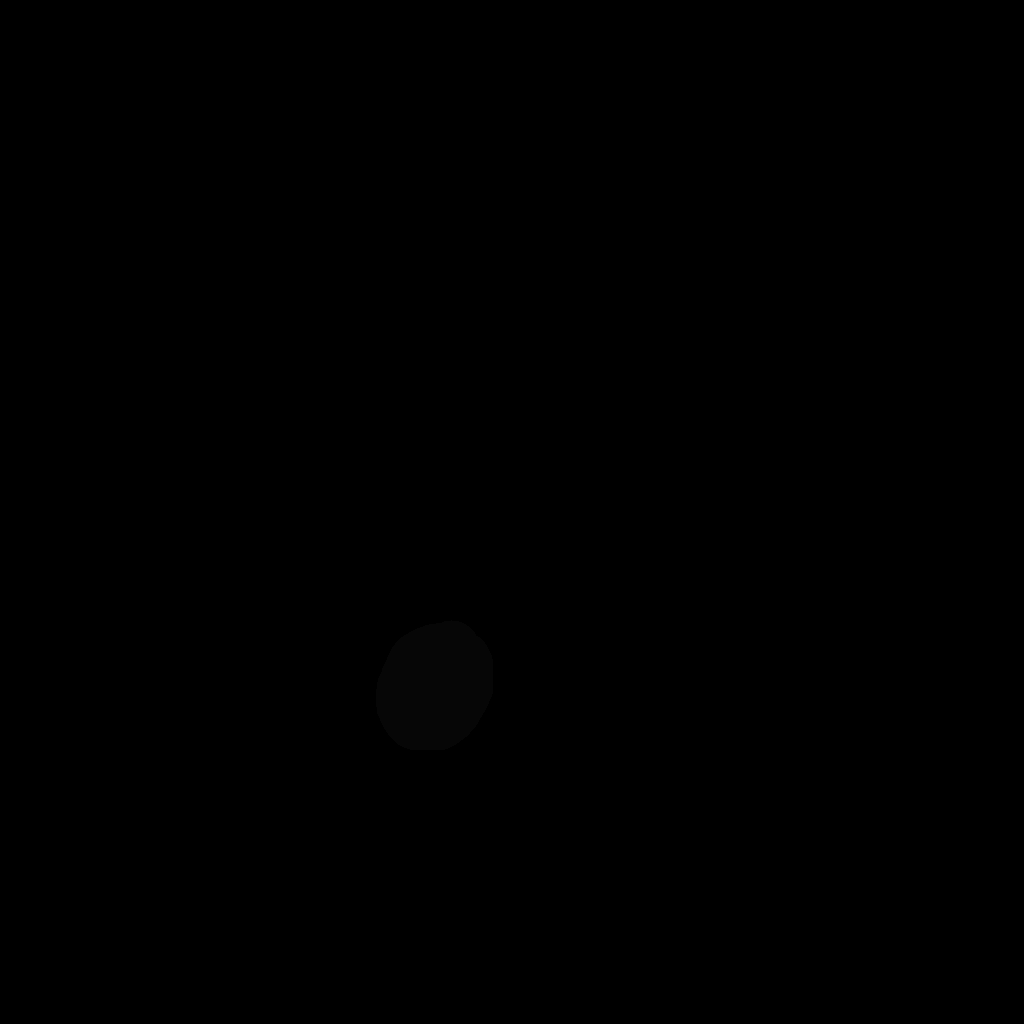

Supplement: Supplemental Information 1 [file peerj-cs-10-2097-s001.zip › IIT-AFF VL/masks/04_00000628.png]

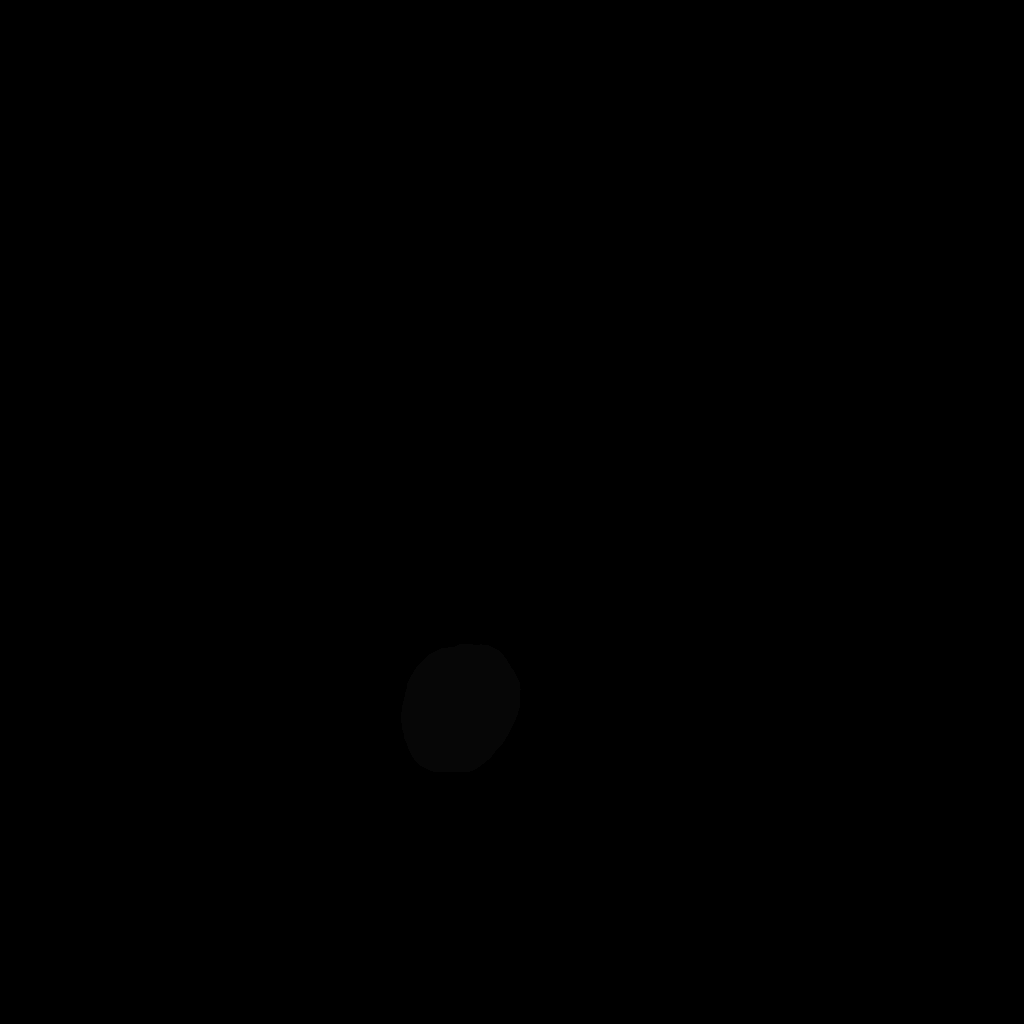

Supplement: Supplemental Information 1 [file peerj-cs-10-2097-s001.zip › IIT-AFF VL/masks/04_00000633.png]

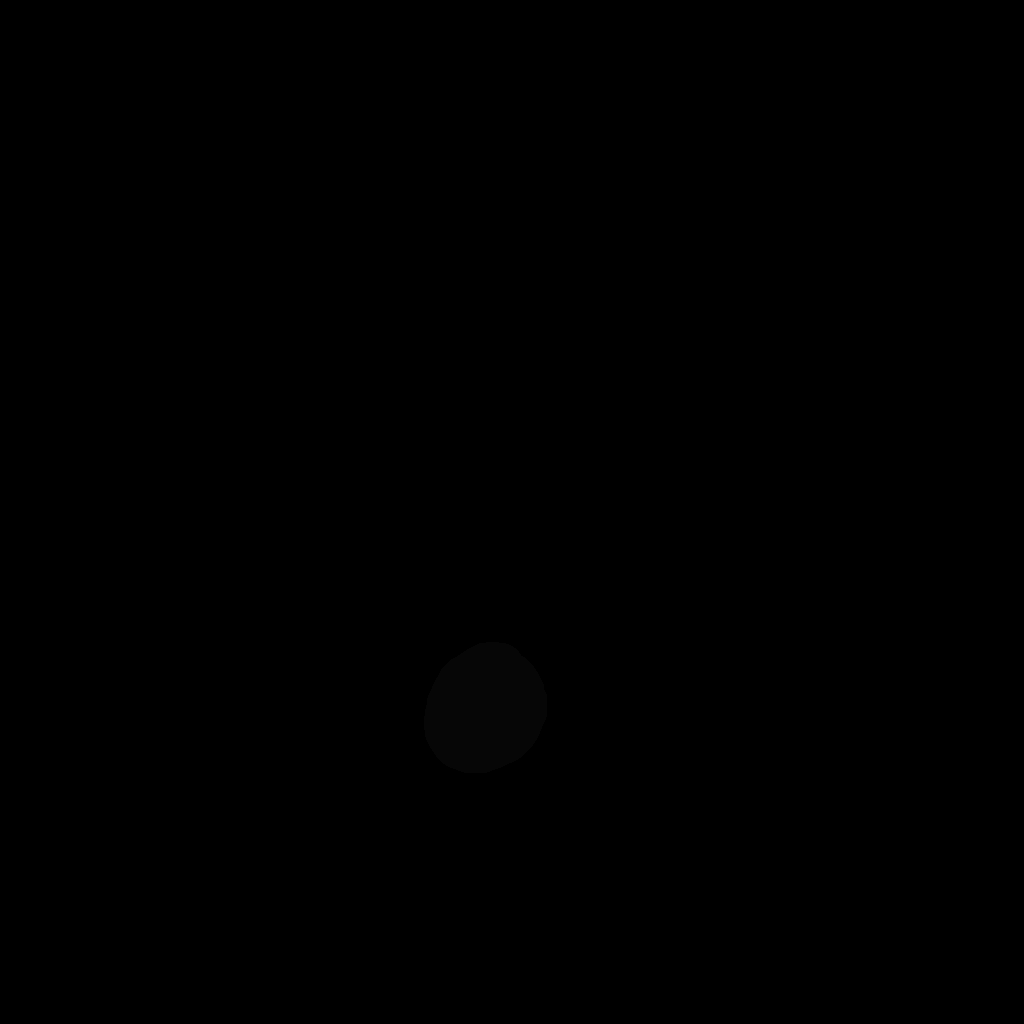

Supplement: Supplemental Information 1 [file peerj-cs-10-2097-s001.zip › IIT-AFF VL/masks/04_00000637.png]

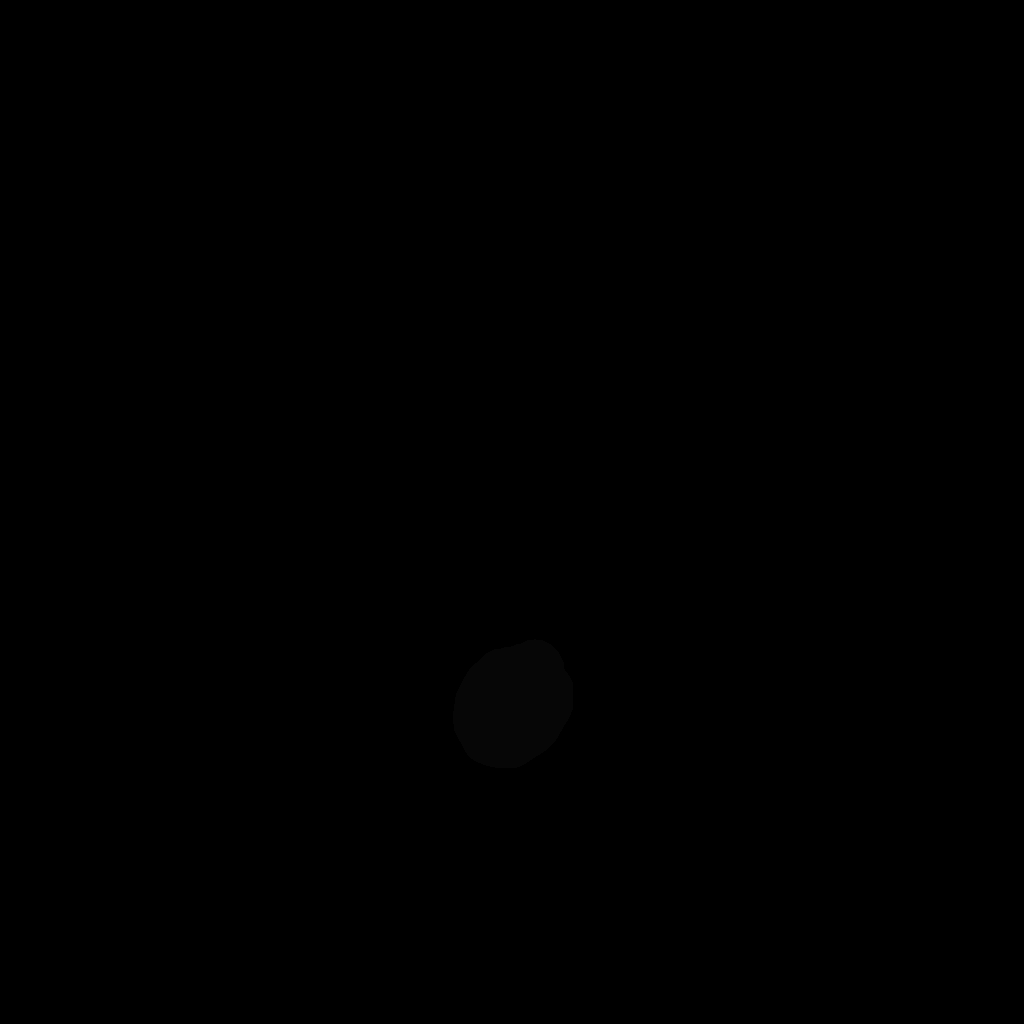

Supplement: Supplemental Information 1 [file peerj-cs-10-2097-s001.zip › IIT-AFF VL/masks/04_00000643.png]

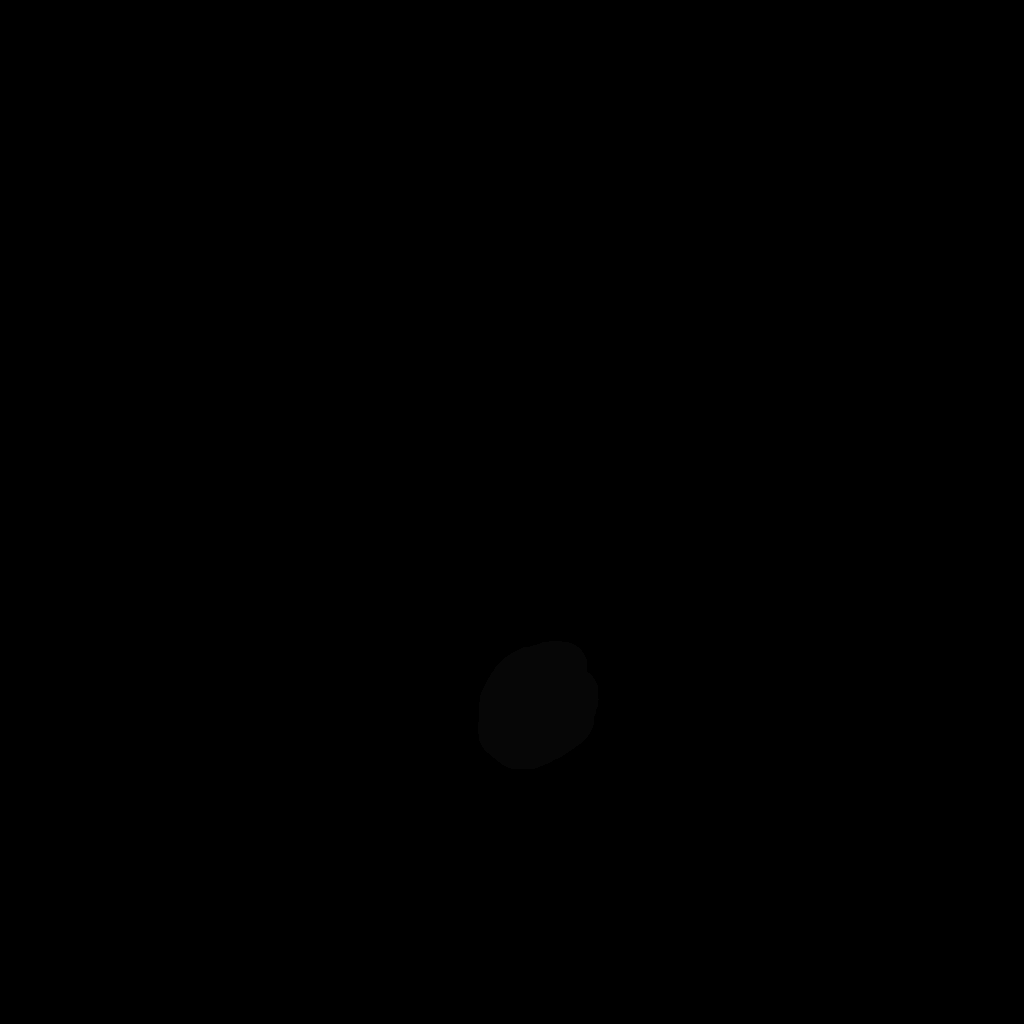

Supplement: Supplemental Information 1 [file peerj-cs-10-2097-s001.zip › IIT-AFF VL/masks/04_00000648.png]

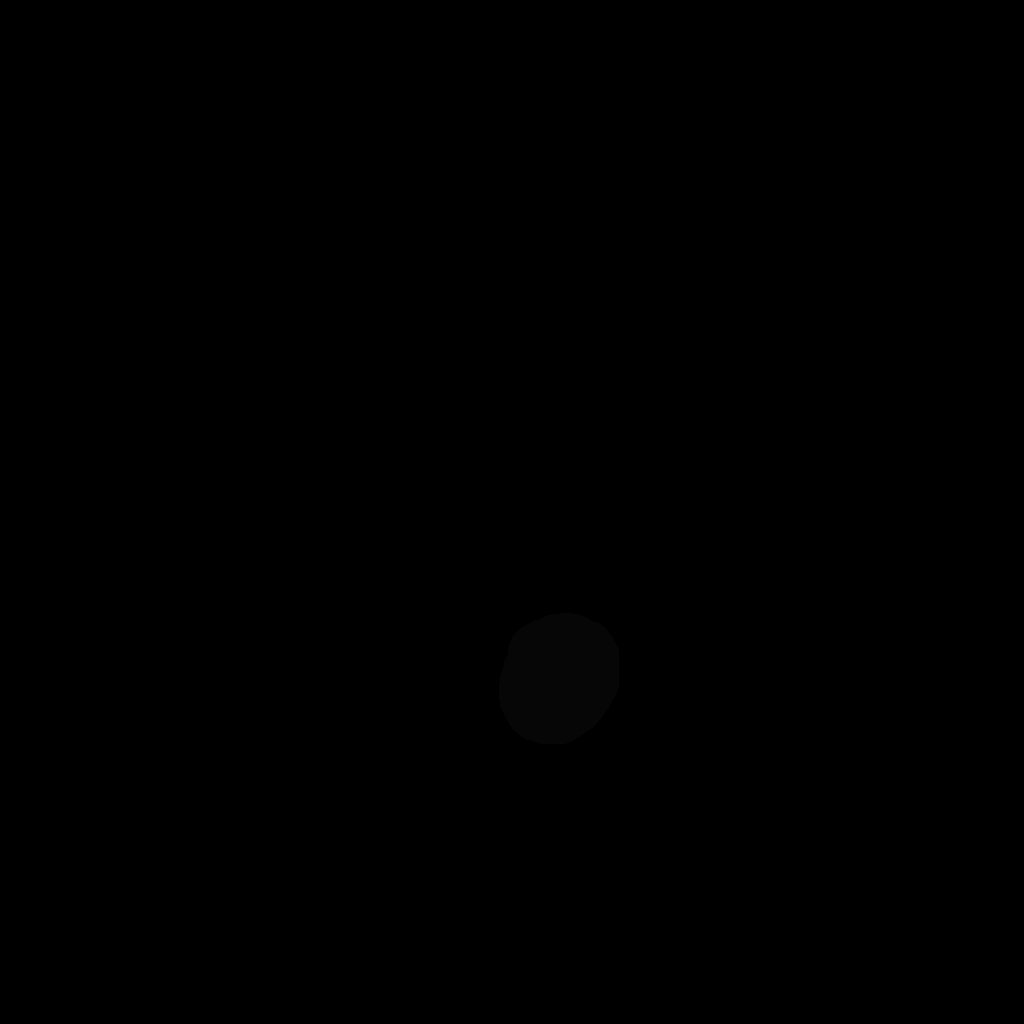

Supplement: Supplemental Information 1 [file peerj-cs-10-2097-s001.zip › IIT-AFF VL/masks/04_00000654.png]

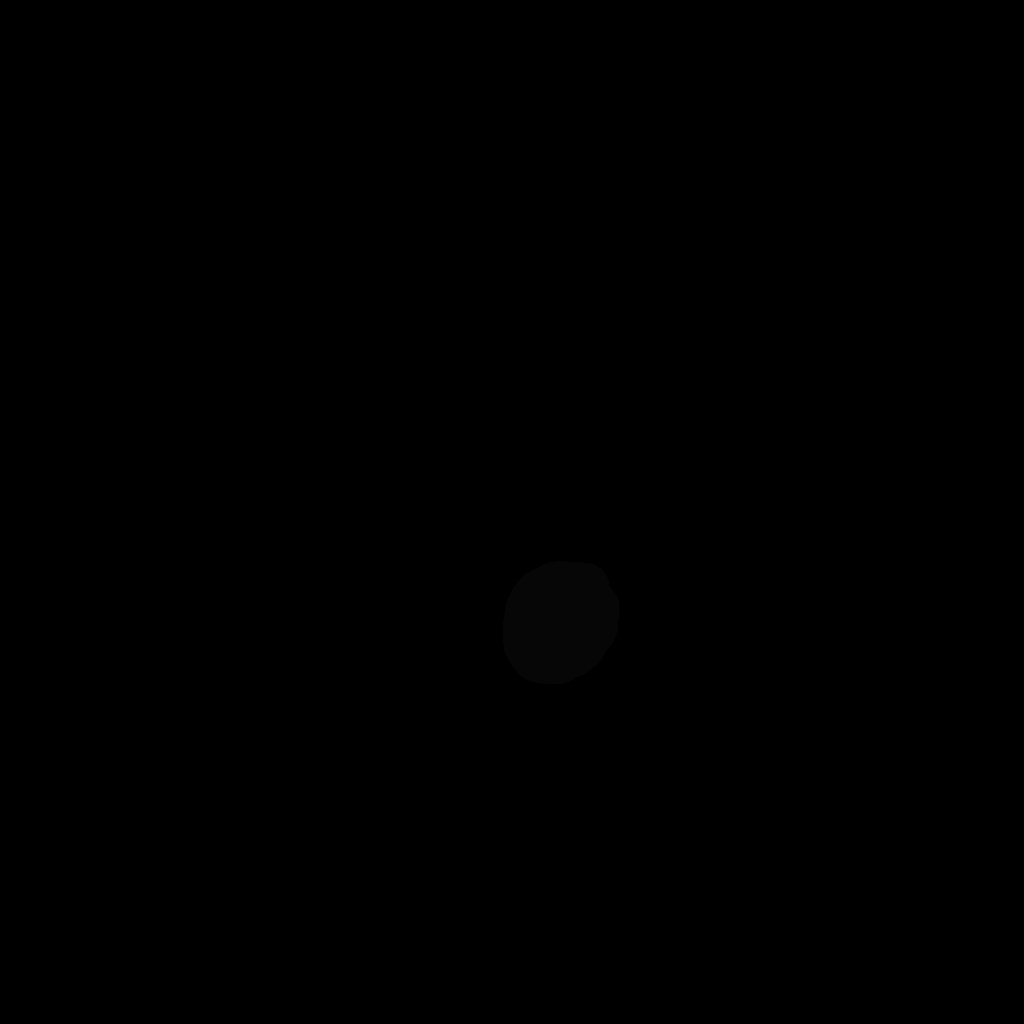

Supplement: Supplemental Information 1 [file peerj-cs-10-2097-s001.zip › IIT-AFF VL/masks/04_00000659.png]

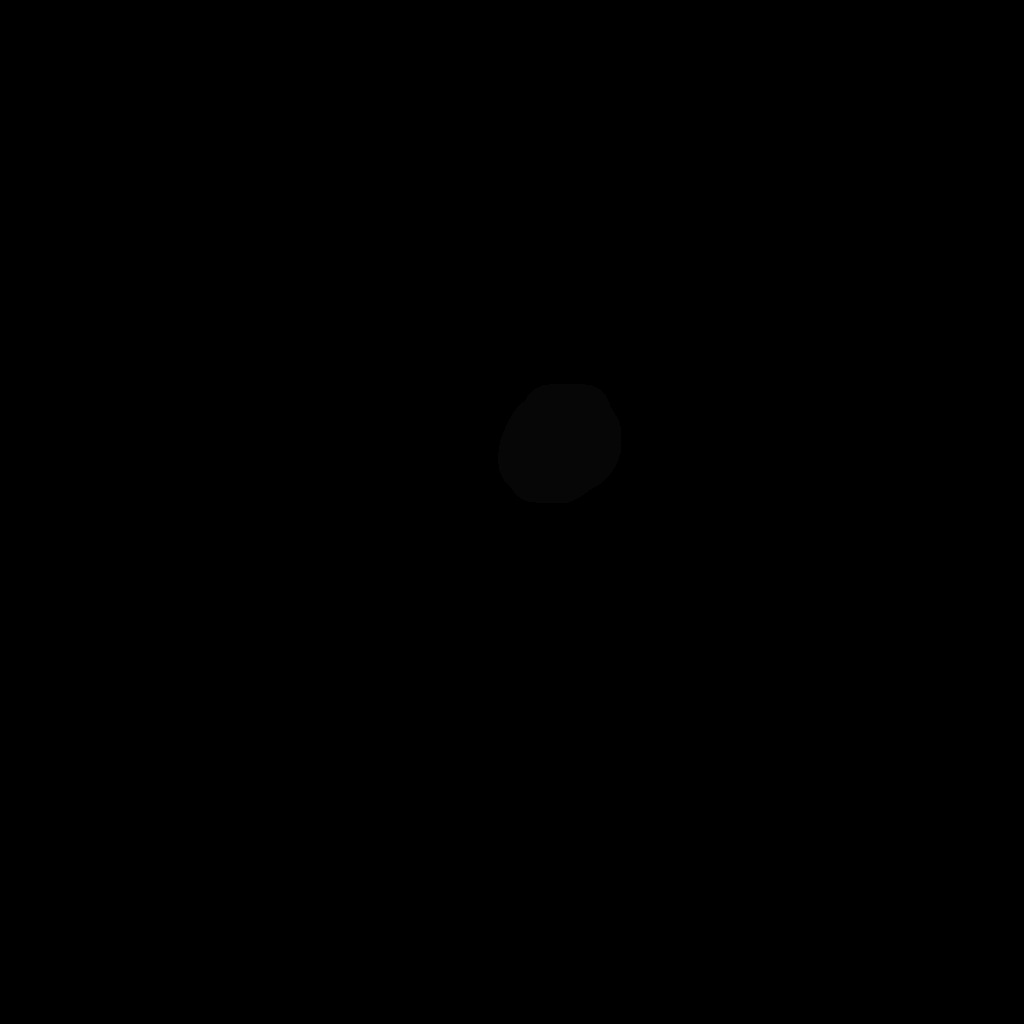

Supplement: Supplemental Information 1 [file peerj-cs-10-2097-s001.zip › IIT-AFF VL/masks/04_00000681.png]

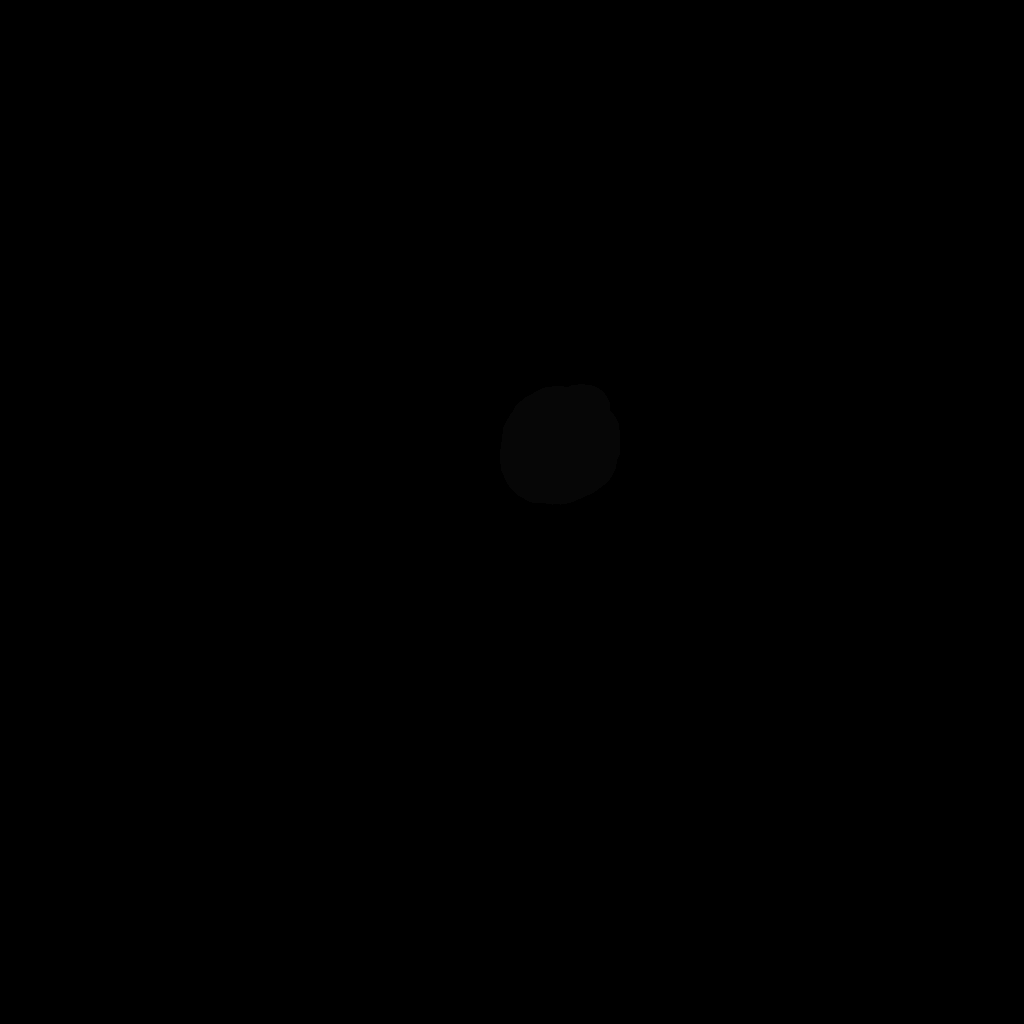

Supplement: Supplemental Information 1 [file peerj-cs-10-2097-s001.zip › IIT-AFF VL/masks/04_00000685.png]

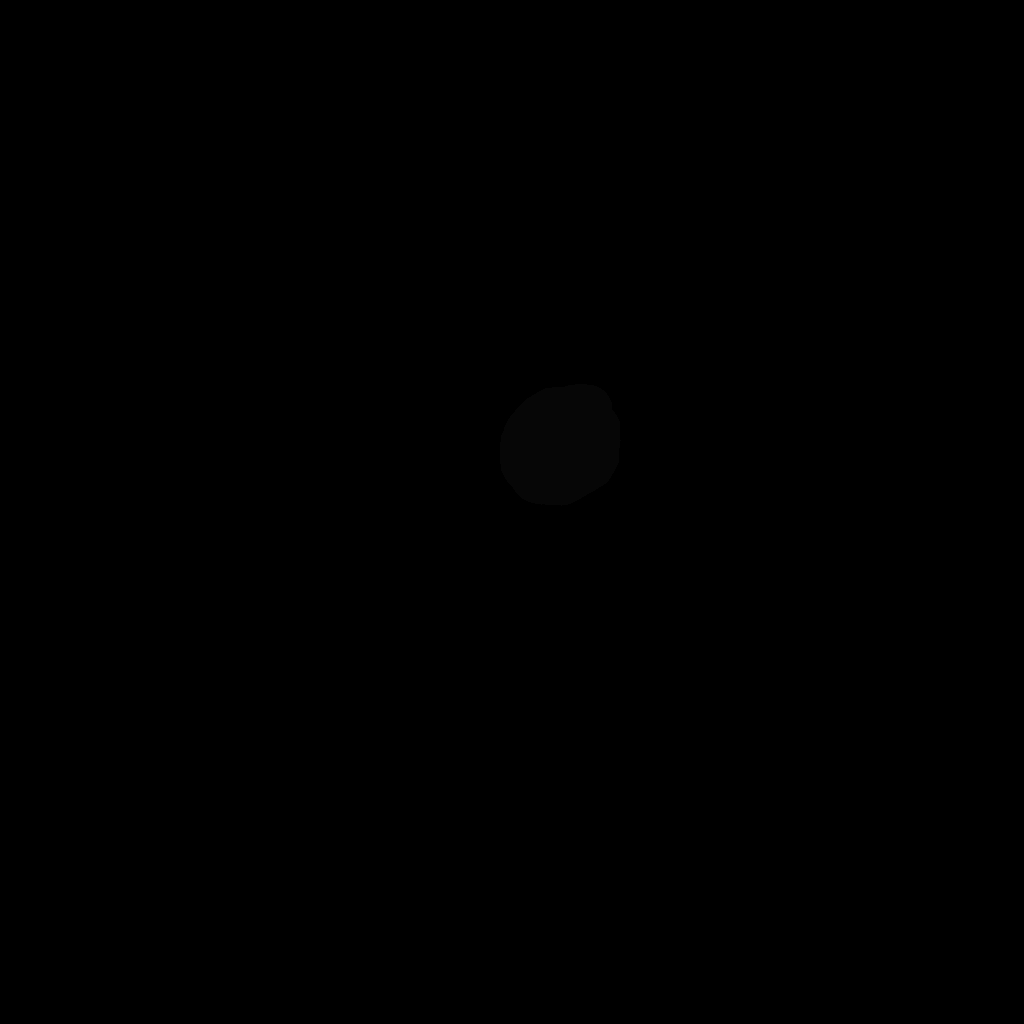

Supplement: Supplemental Information 1 [file peerj-cs-10-2097-s001.zip › IIT-AFF VL/masks/04_00000689.png]

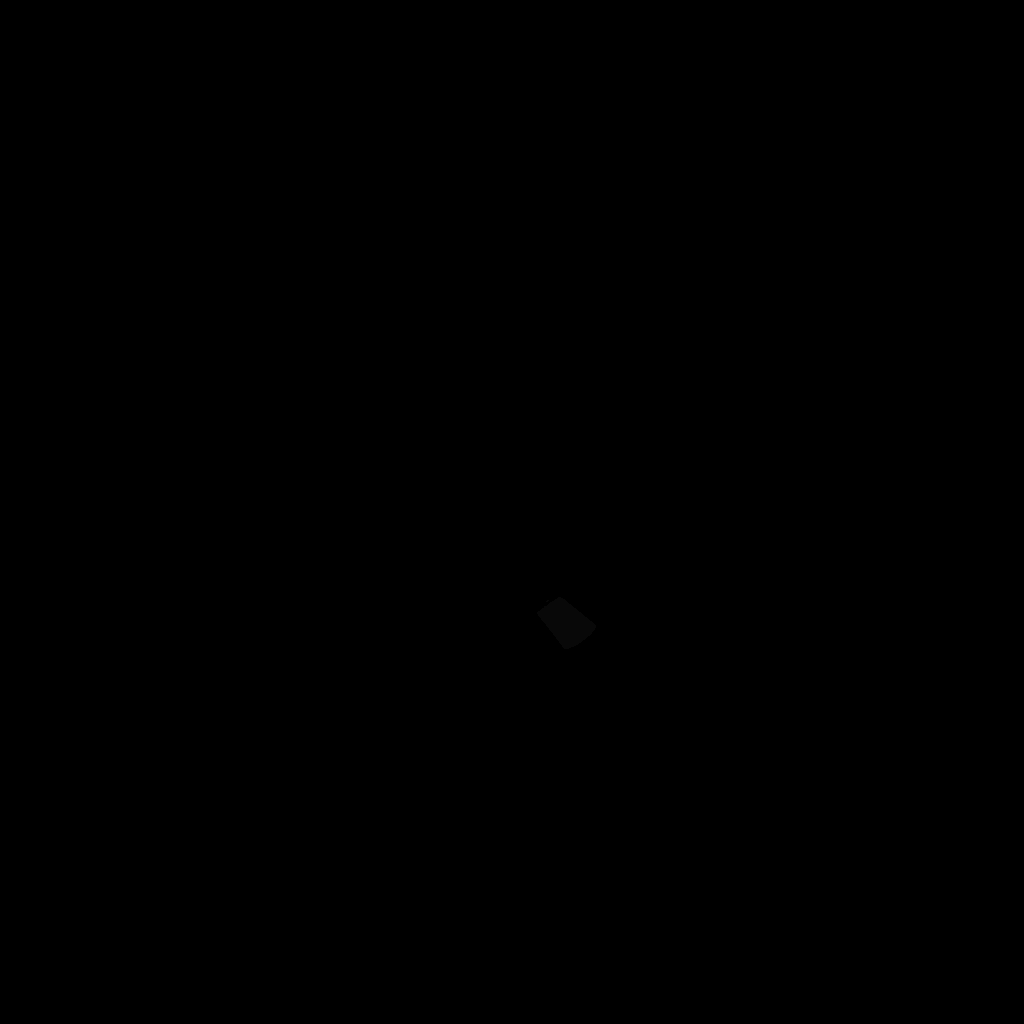

Supplement: Supplemental Information 1 [file peerj-cs-10-2097-s001.zip › IIT-AFF VL/masks/05_00000107.png]

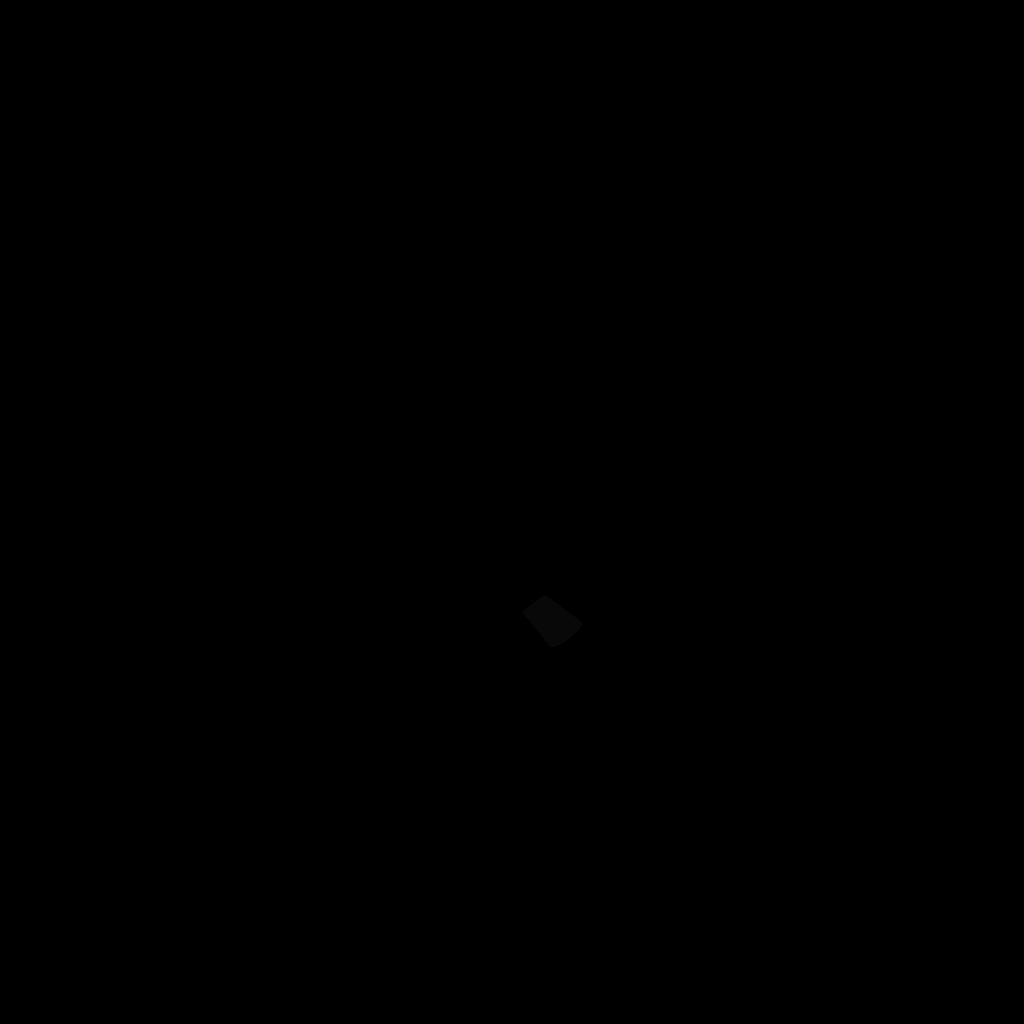

Supplement: Supplemental Information 1 [file peerj-cs-10-2097-s001.zip › IIT-AFF VL/masks/05_00000111.png]

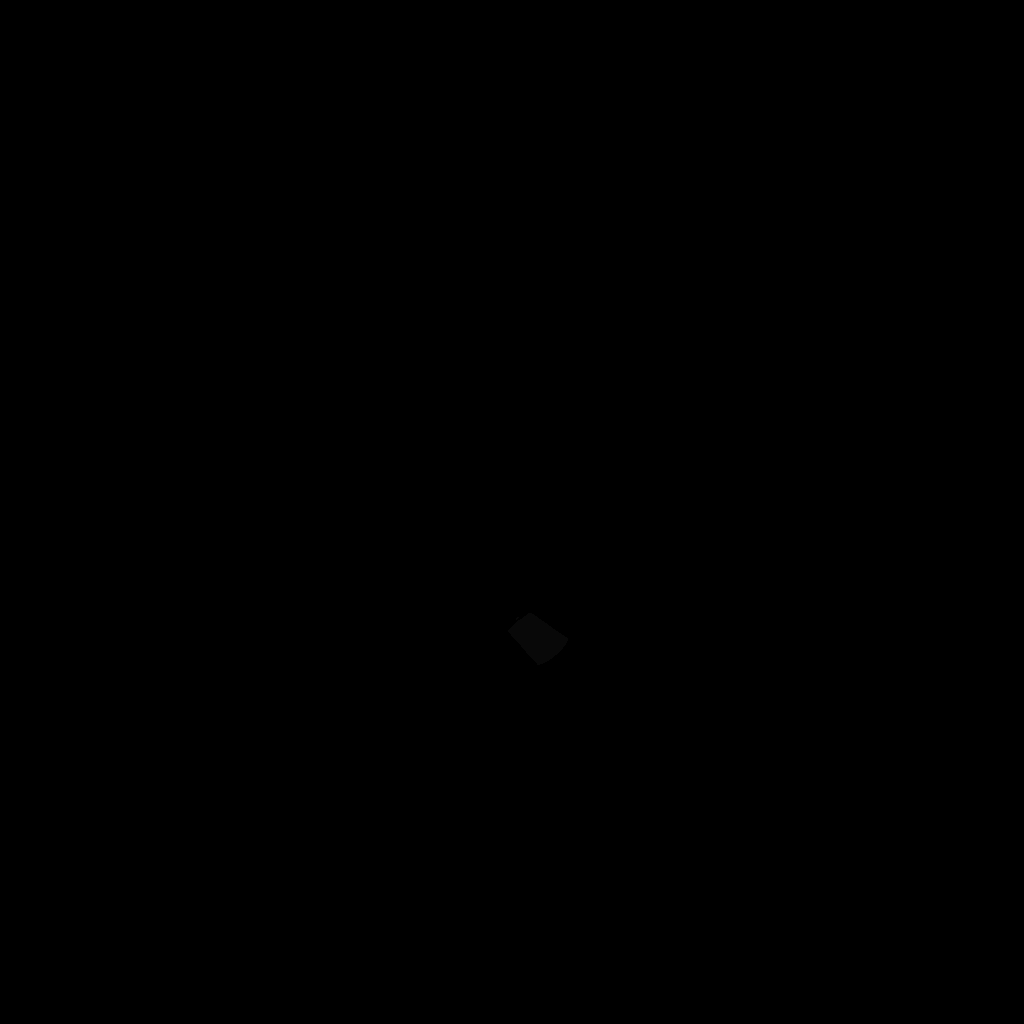

Supplement: Supplemental Information 1 [file peerj-cs-10-2097-s001.zip › IIT-AFF VL/masks/05_00000115.png]

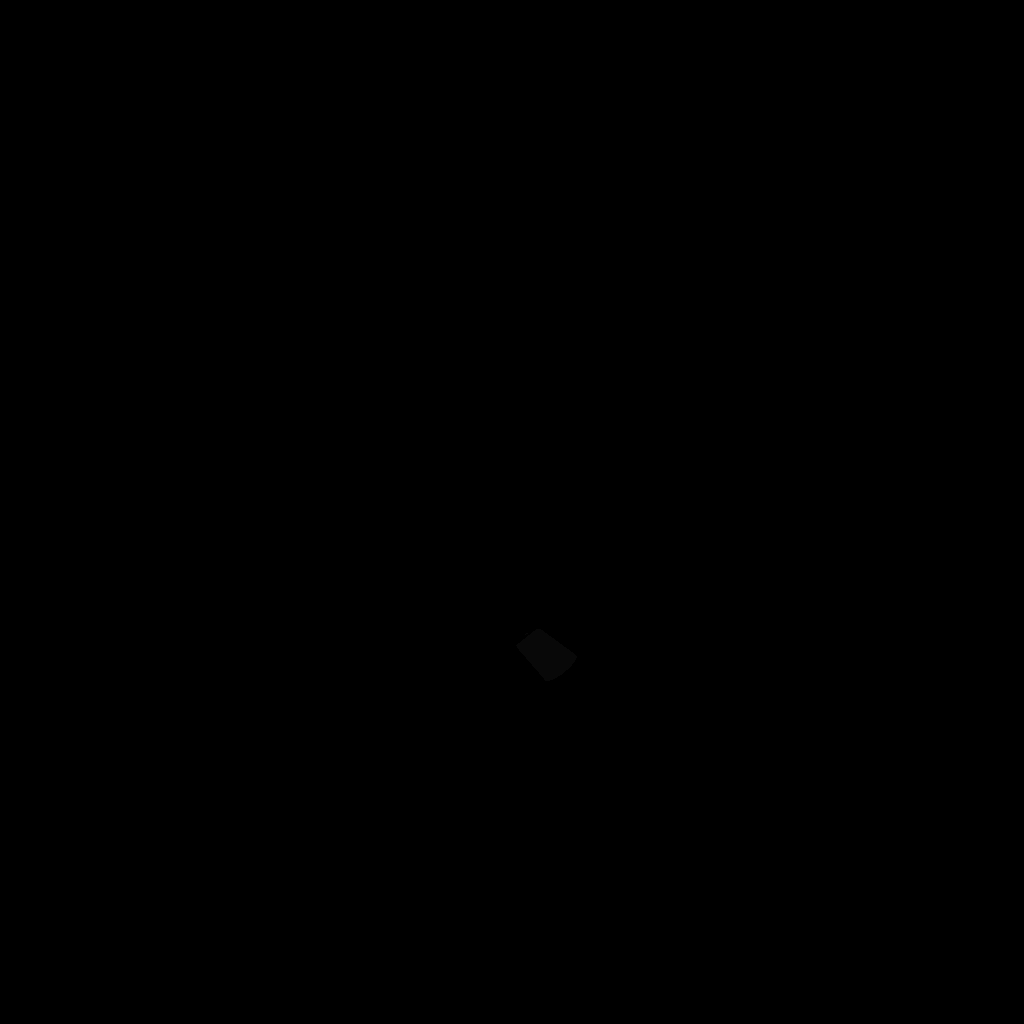

Supplement: Supplemental Information 1 [file peerj-cs-10-2097-s001.zip › IIT-AFF VL/masks/05_00000119.png]

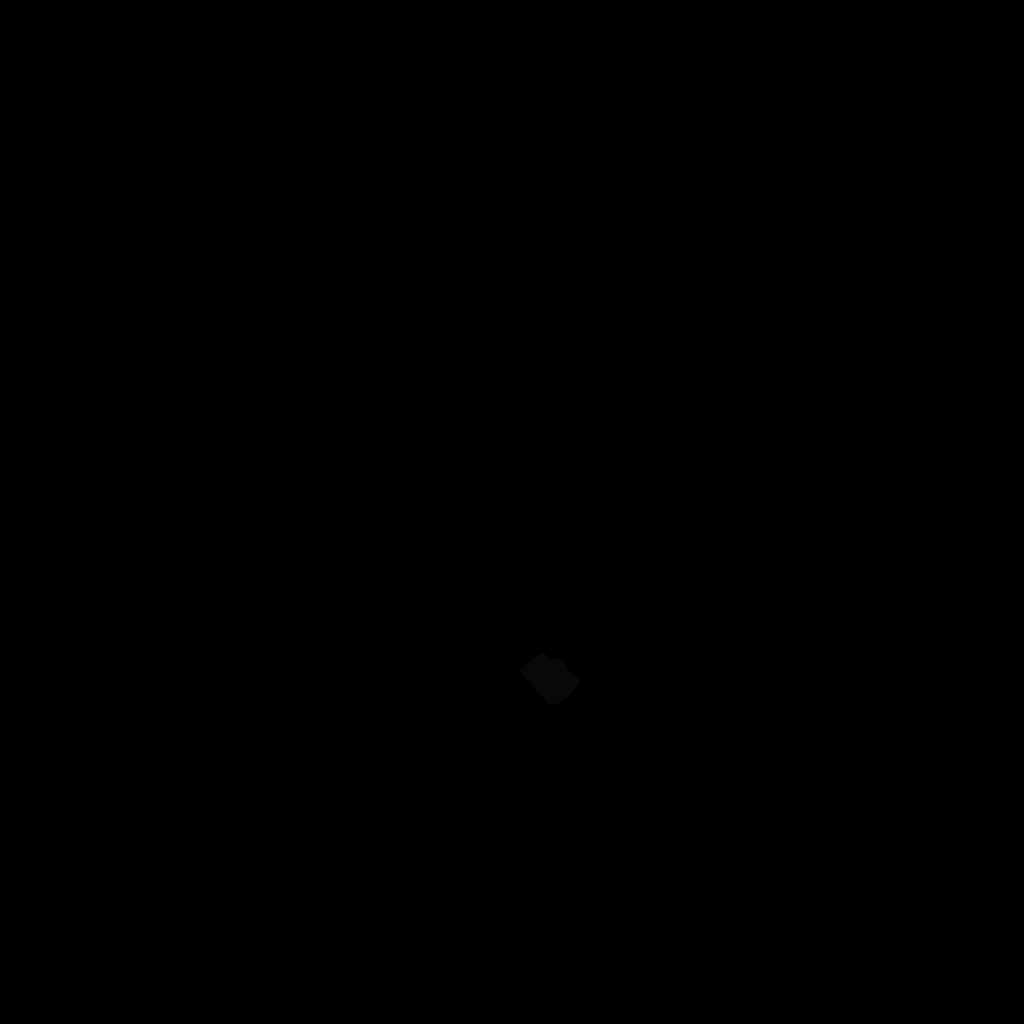

Supplement: Supplemental Information 1 [file peerj-cs-10-2097-s001.zip › IIT-AFF VL/masks/05_00000123.png]

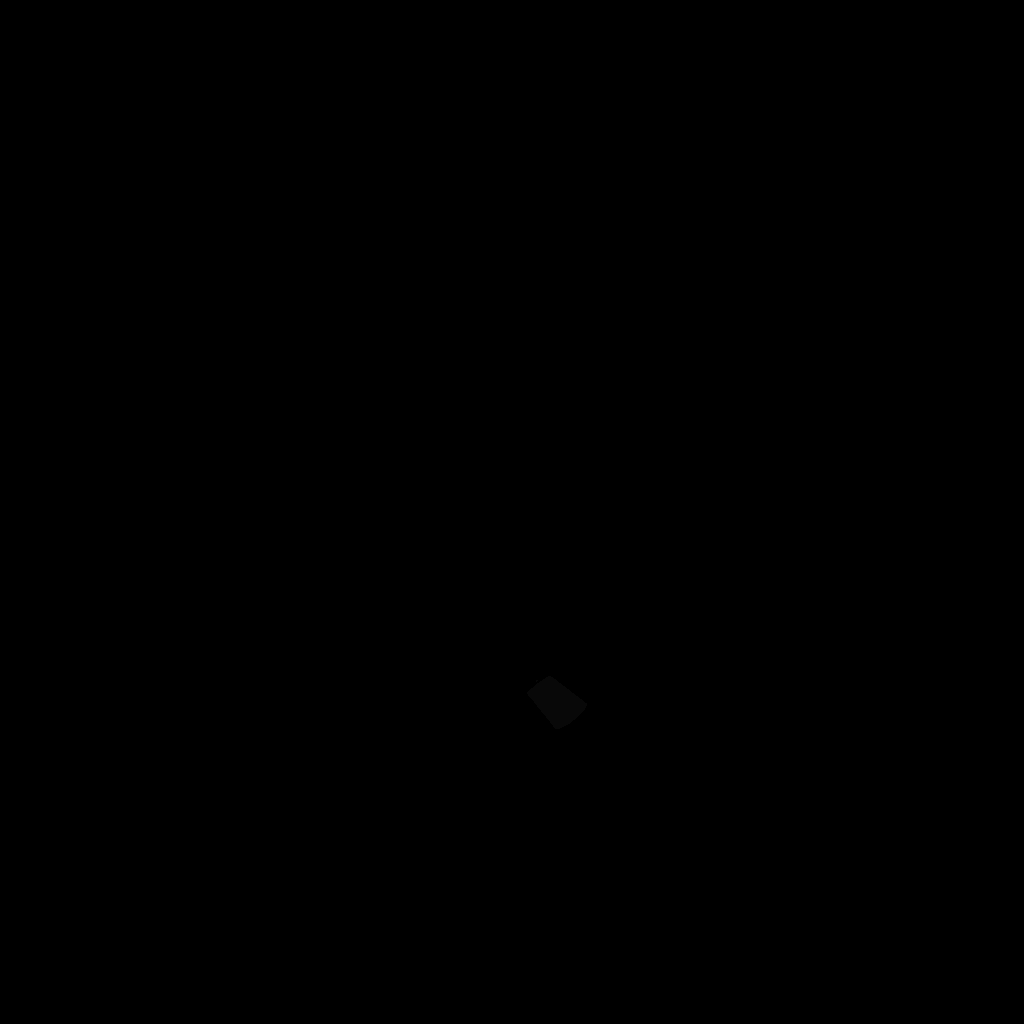

Supplement: Supplemental Information 1 [file peerj-cs-10-2097-s001.zip › IIT-AFF VL/masks/05_00000127.png]

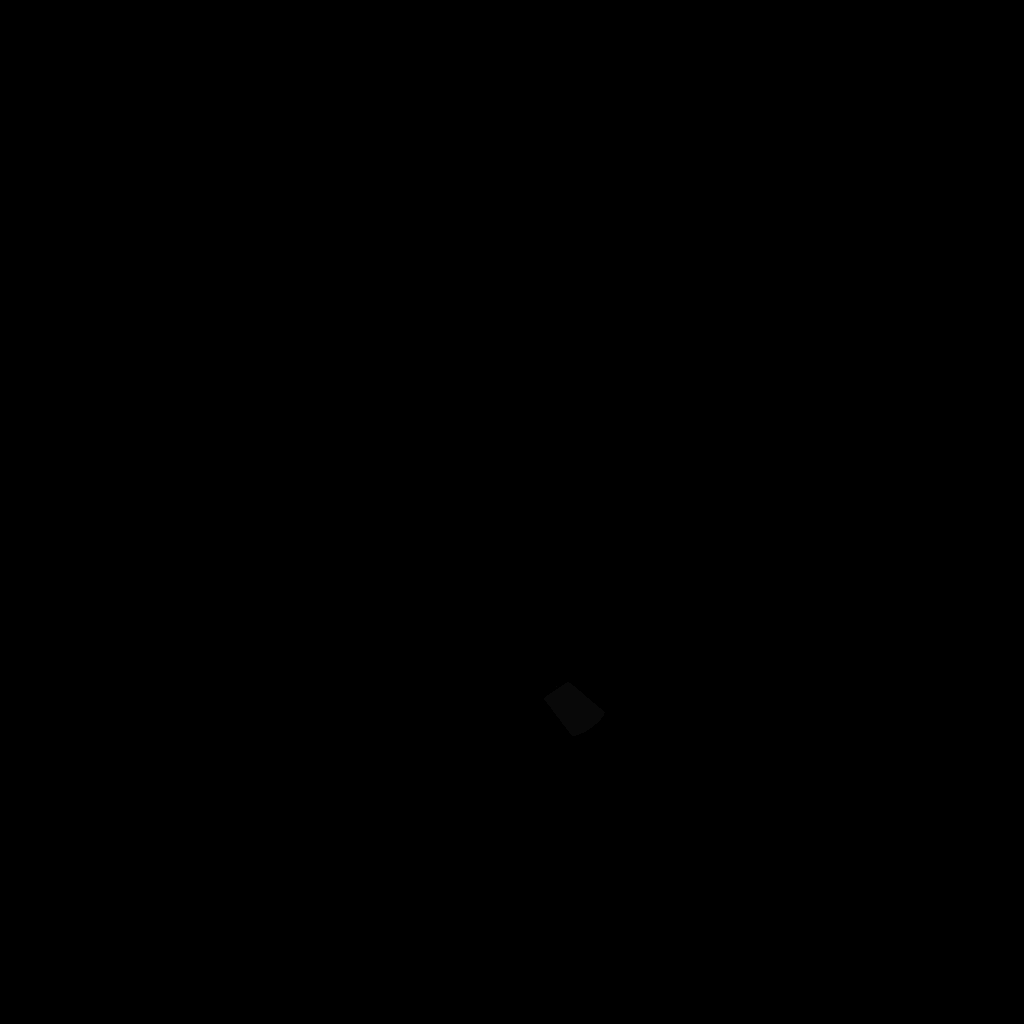

Supplement: Supplemental Information 1 [file peerj-cs-10-2097-s001.zip › IIT-AFF VL/masks/05_00000131.png]

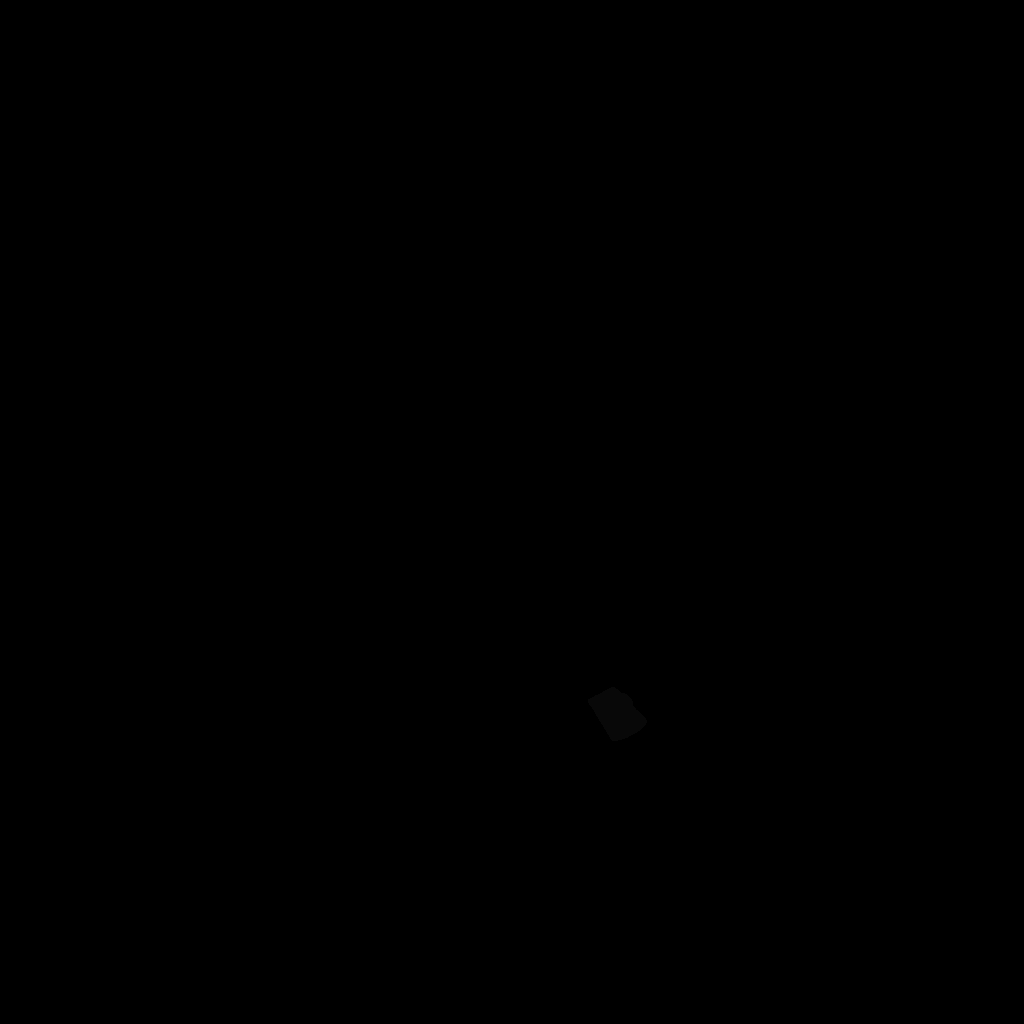

Supplement: Supplemental Information 1 [file peerj-cs-10-2097-s001.zip › IIT-AFF VL/masks/05_00000137.png]

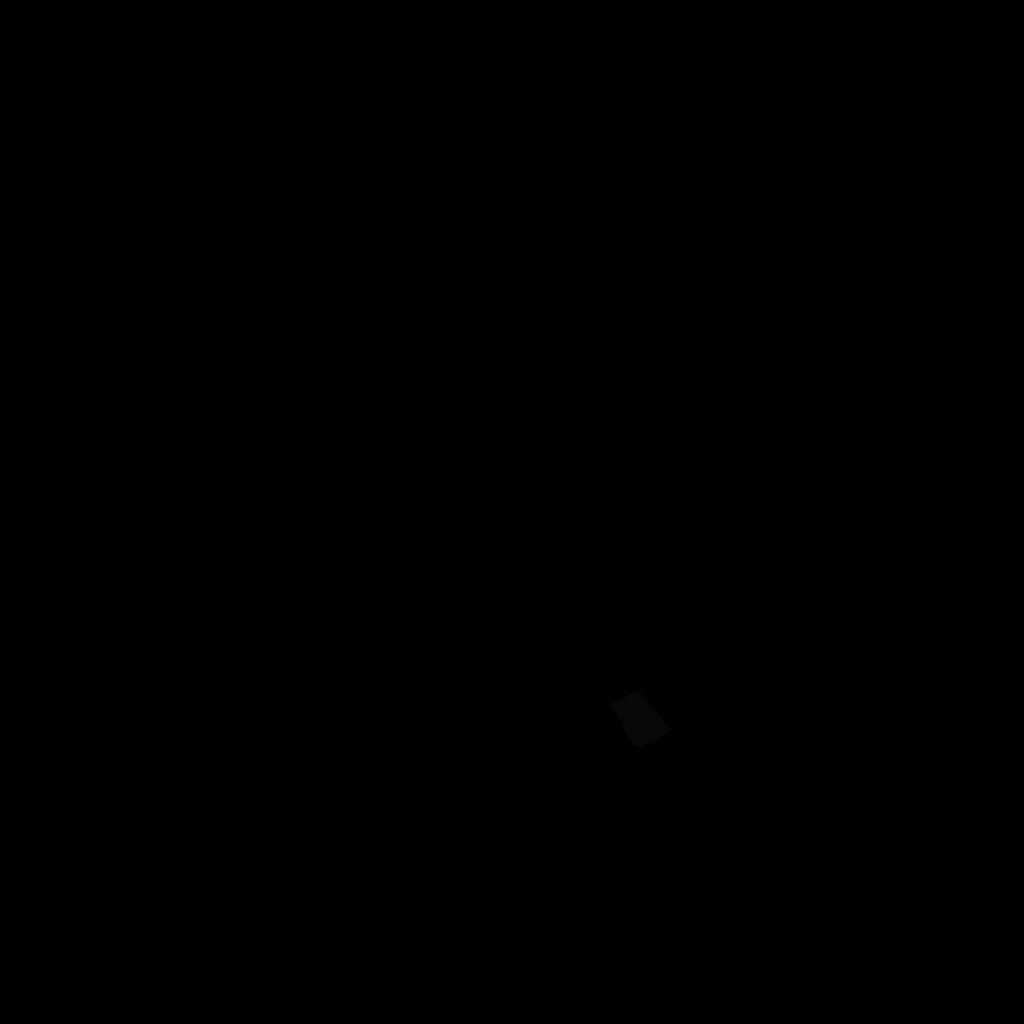

Supplement: Supplemental Information 1 [file peerj-cs-10-2097-s001.zip › IIT-AFF VL/masks/05_00000141.png]
