# Supplementary figures and images for: Knowledge enhanced bottom-up affordance grounding for robotic interaction (part 1 of 2)
Source: PeerJ Comput Sci. 2024 Jul 5;10:e2097. doi: 10.7717/peerj-cs.2097 (PMC11232630; doi:10.7717/peerj-cs.2097)

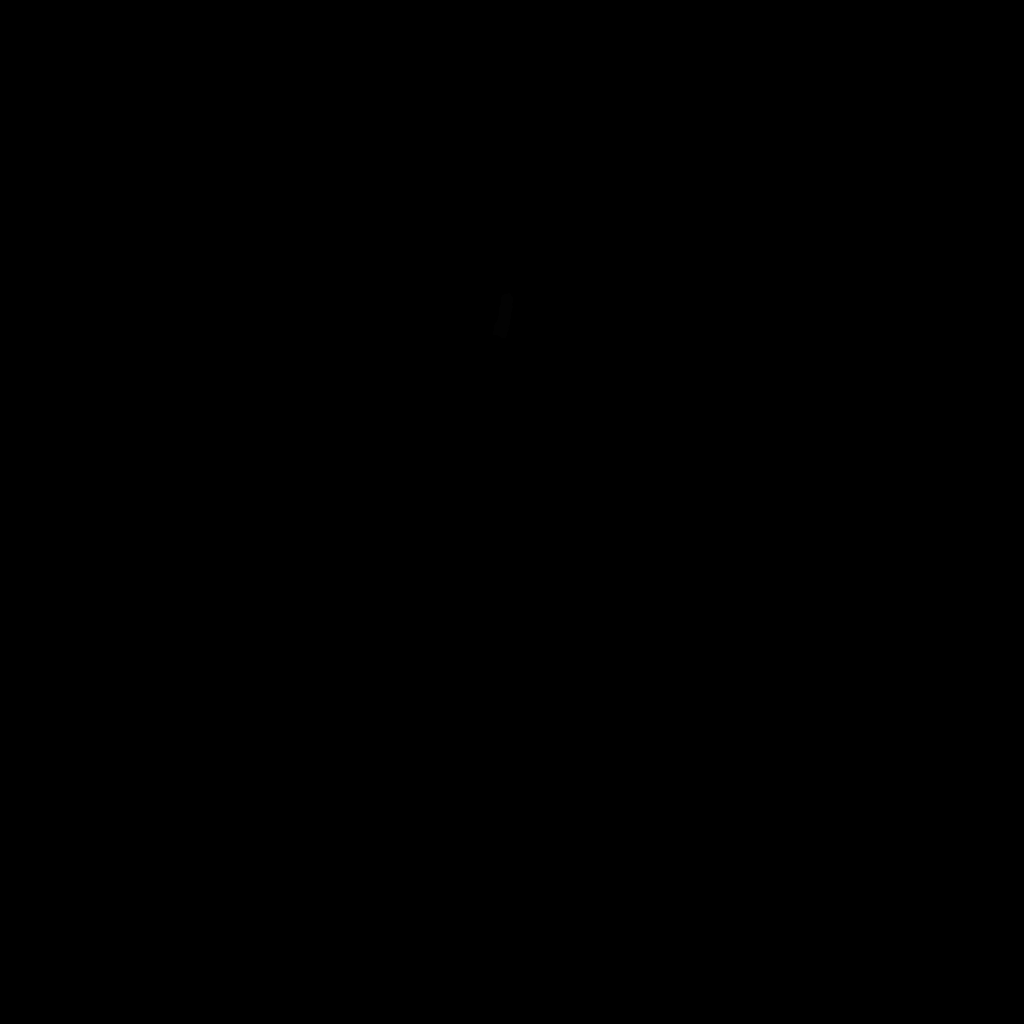

Supplement: Supplemental Information 1 [file peerj-cs-10-2097-s001.zip › IIT-AFF VL/masks/00_00000090.png]

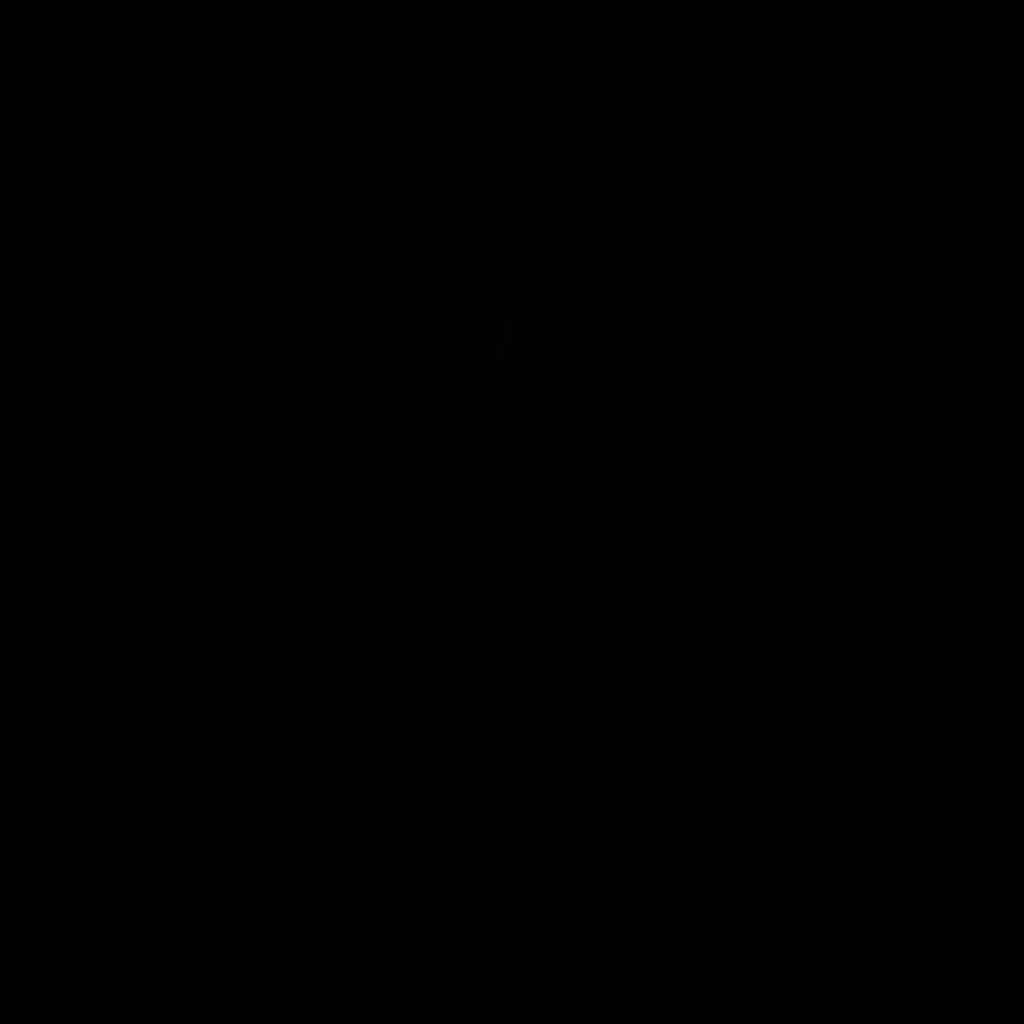

Supplement: Supplemental Information 1 [file peerj-cs-10-2097-s001.zip › IIT-AFF VL/masks/00_00000097.png]

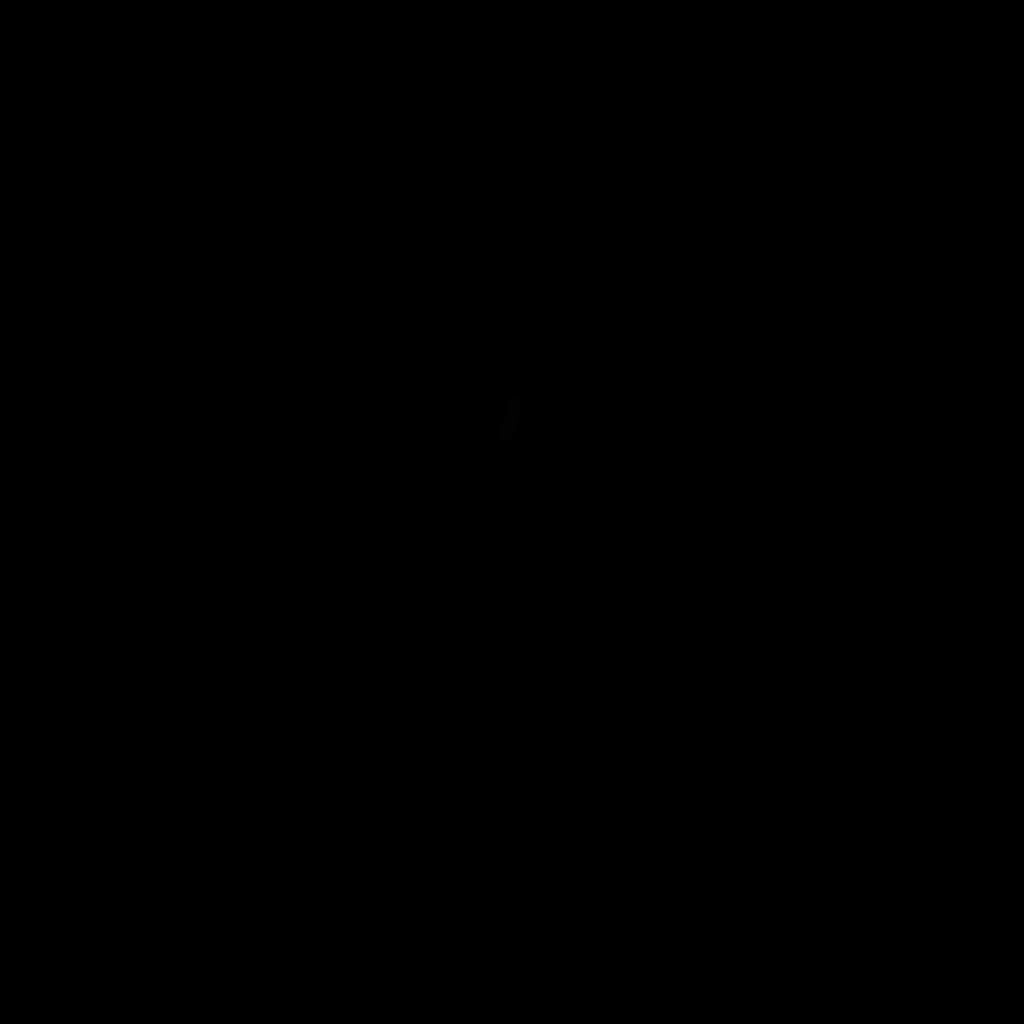

Supplement: Supplemental Information 1 [file peerj-cs-10-2097-s001.zip › IIT-AFF VL/masks/00_00000116.png]

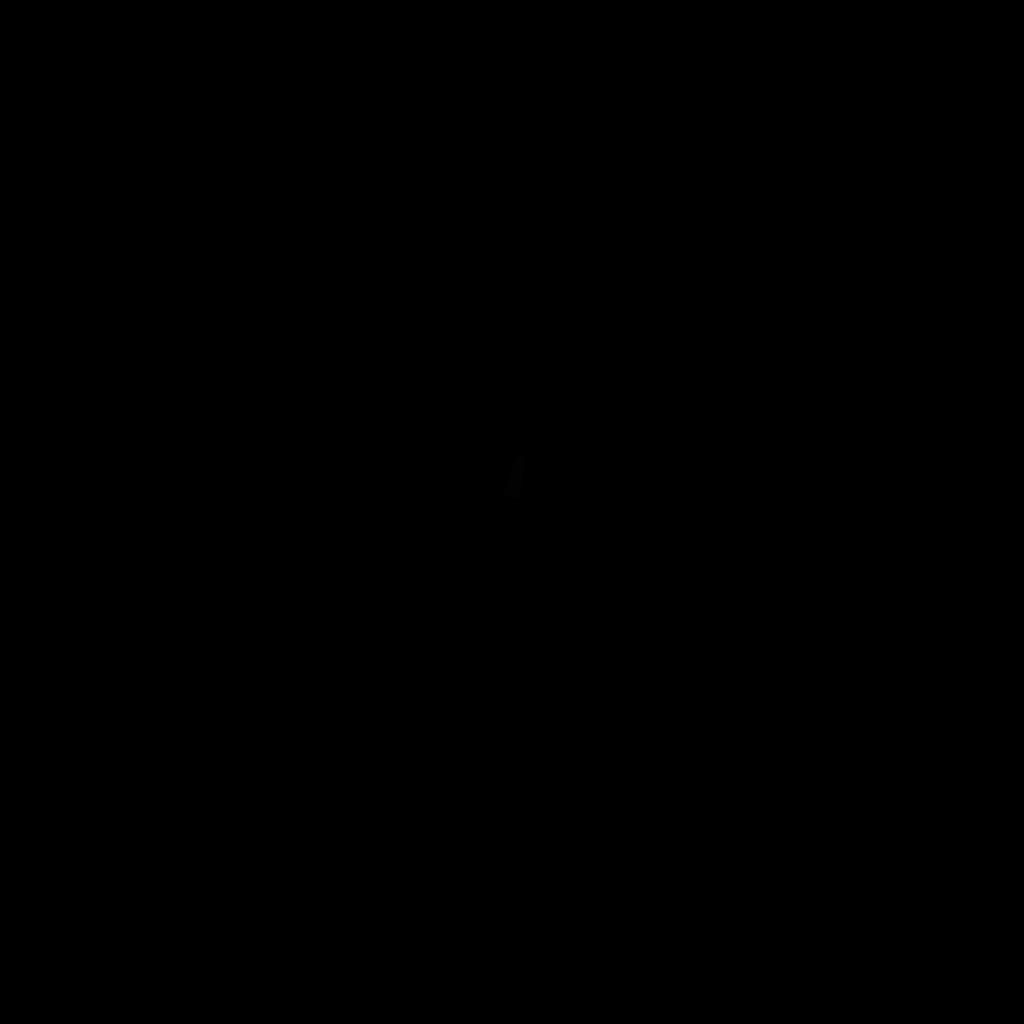

Supplement: Supplemental Information 1 [file peerj-cs-10-2097-s001.zip › IIT-AFF VL/masks/00_00000127.png]

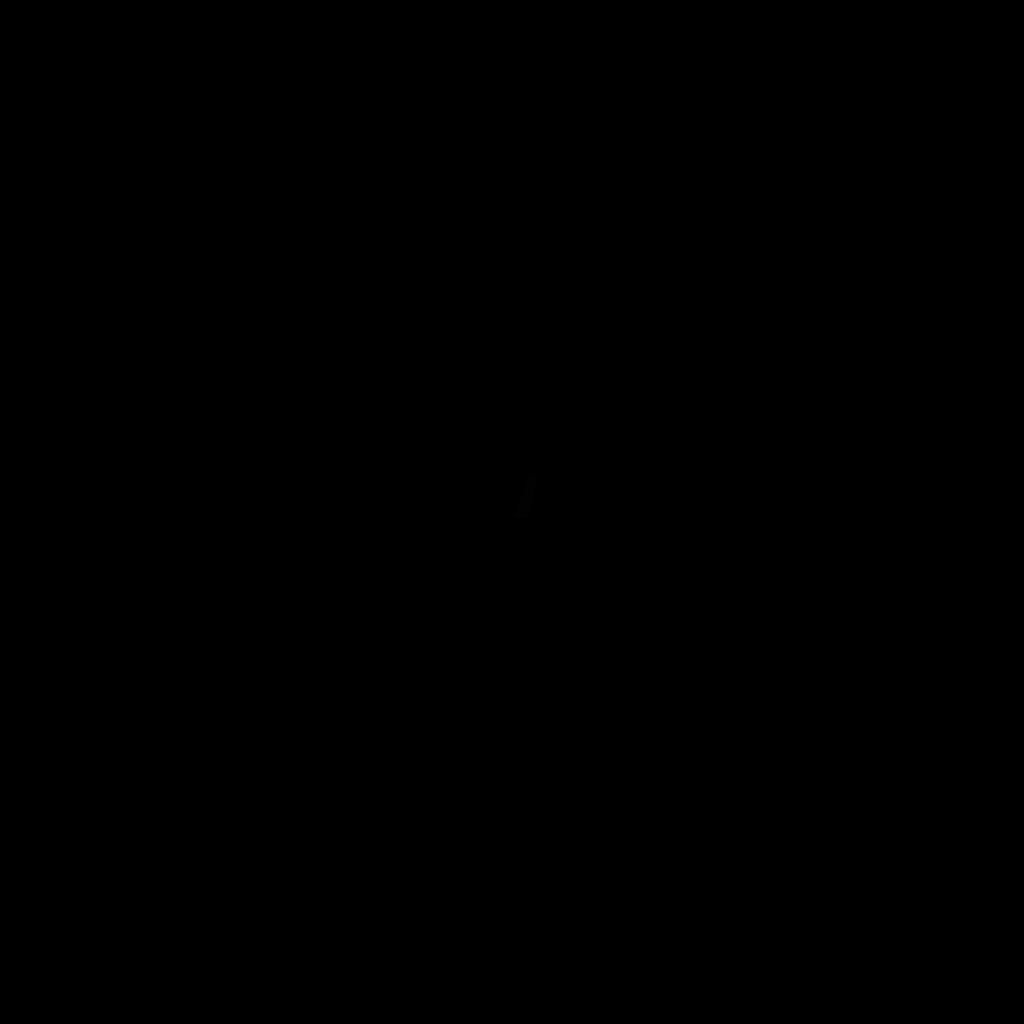

Supplement: Supplemental Information 1 [file peerj-cs-10-2097-s001.zip › IIT-AFF VL/masks/00_00000133.png]

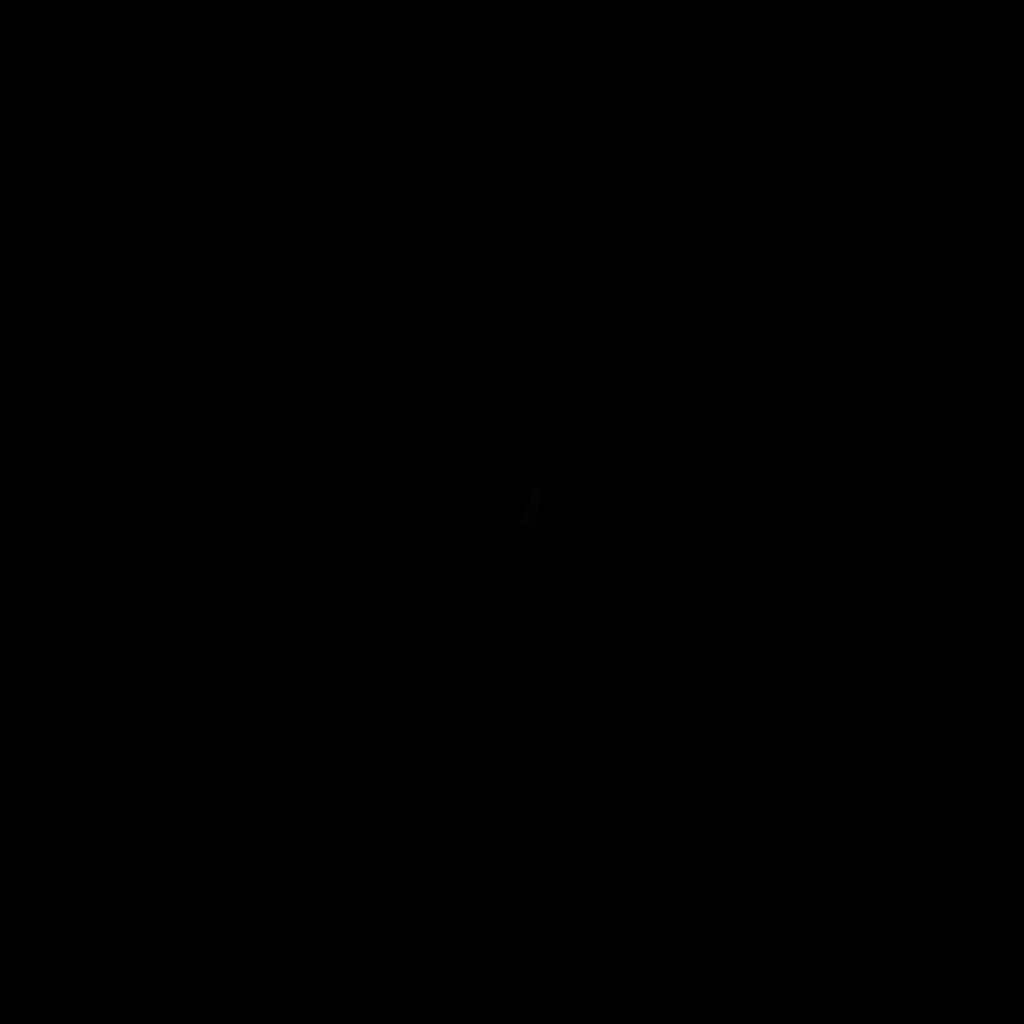

Supplement: Supplemental Information 1 [file peerj-cs-10-2097-s001.zip › IIT-AFF VL/masks/00_00000137.png]

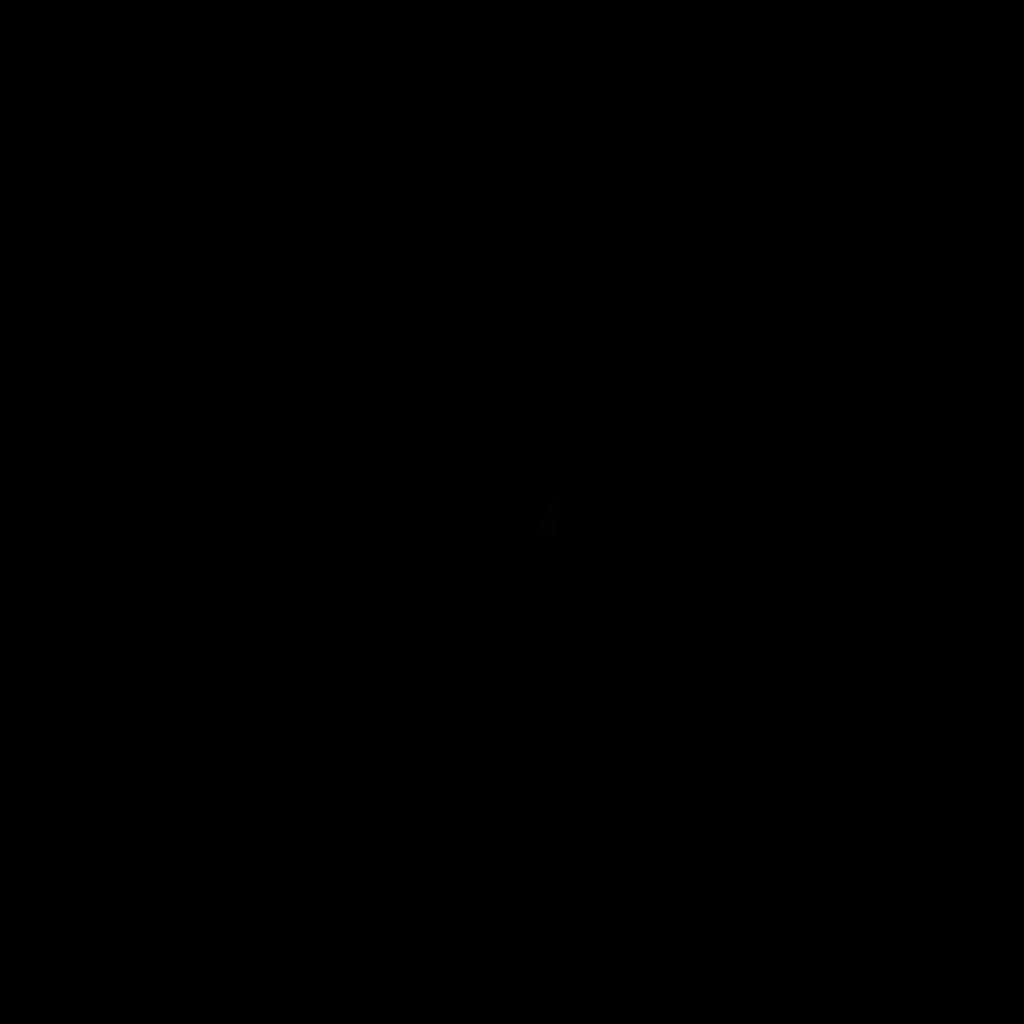

Supplement: Supplemental Information 1 [file peerj-cs-10-2097-s001.zip › IIT-AFF VL/masks/00_00000141.png]

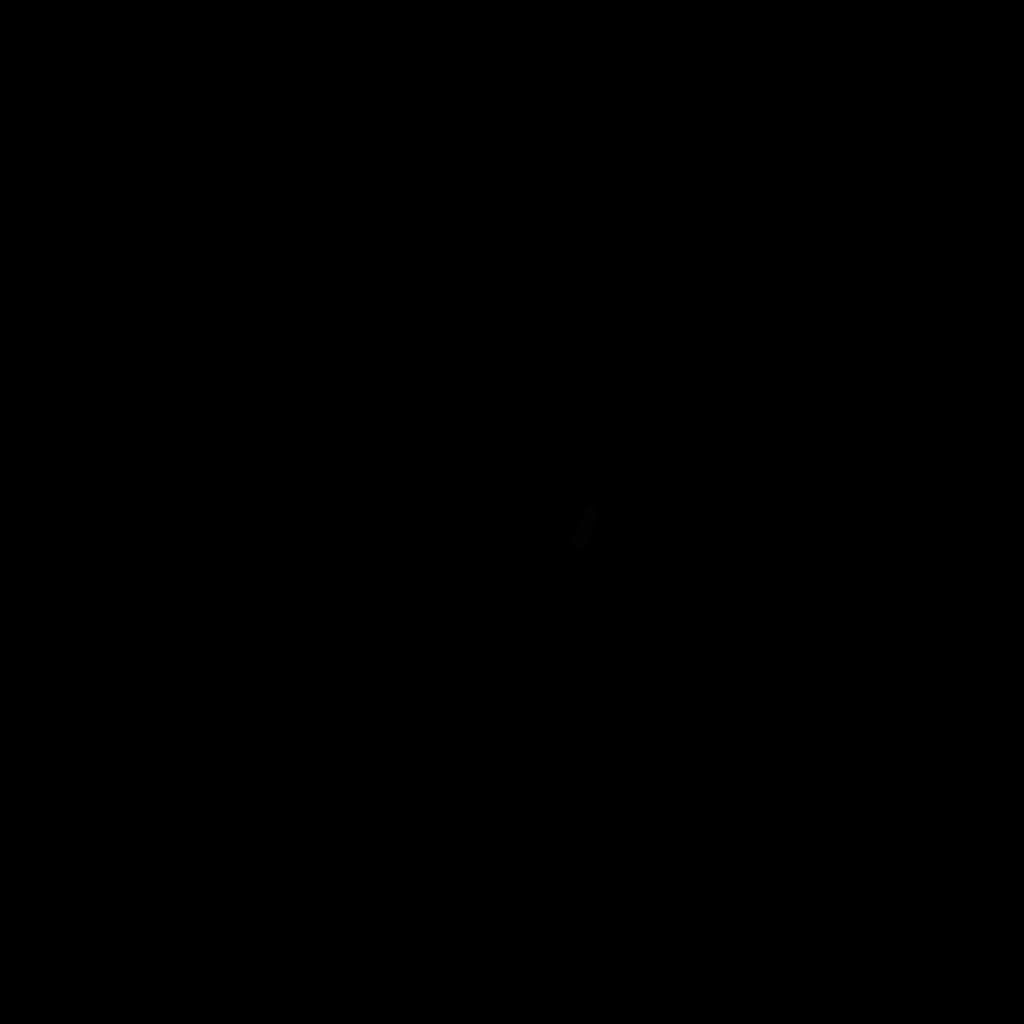

Supplement: Supplemental Information 1 [file peerj-cs-10-2097-s001.zip › IIT-AFF VL/masks/00_00000148.png]

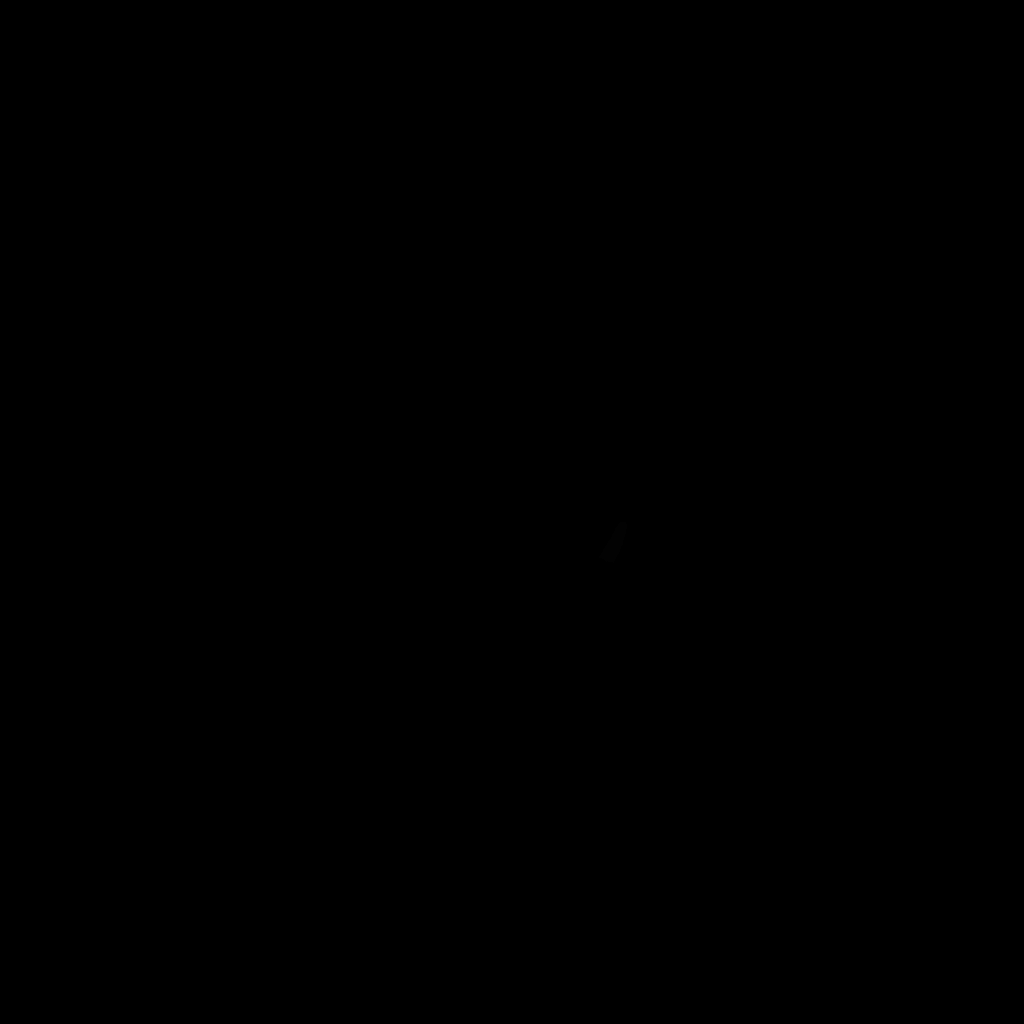

Supplement: Supplemental Information 1 [file peerj-cs-10-2097-s001.zip › IIT-AFF VL/masks/00_00000154.png]

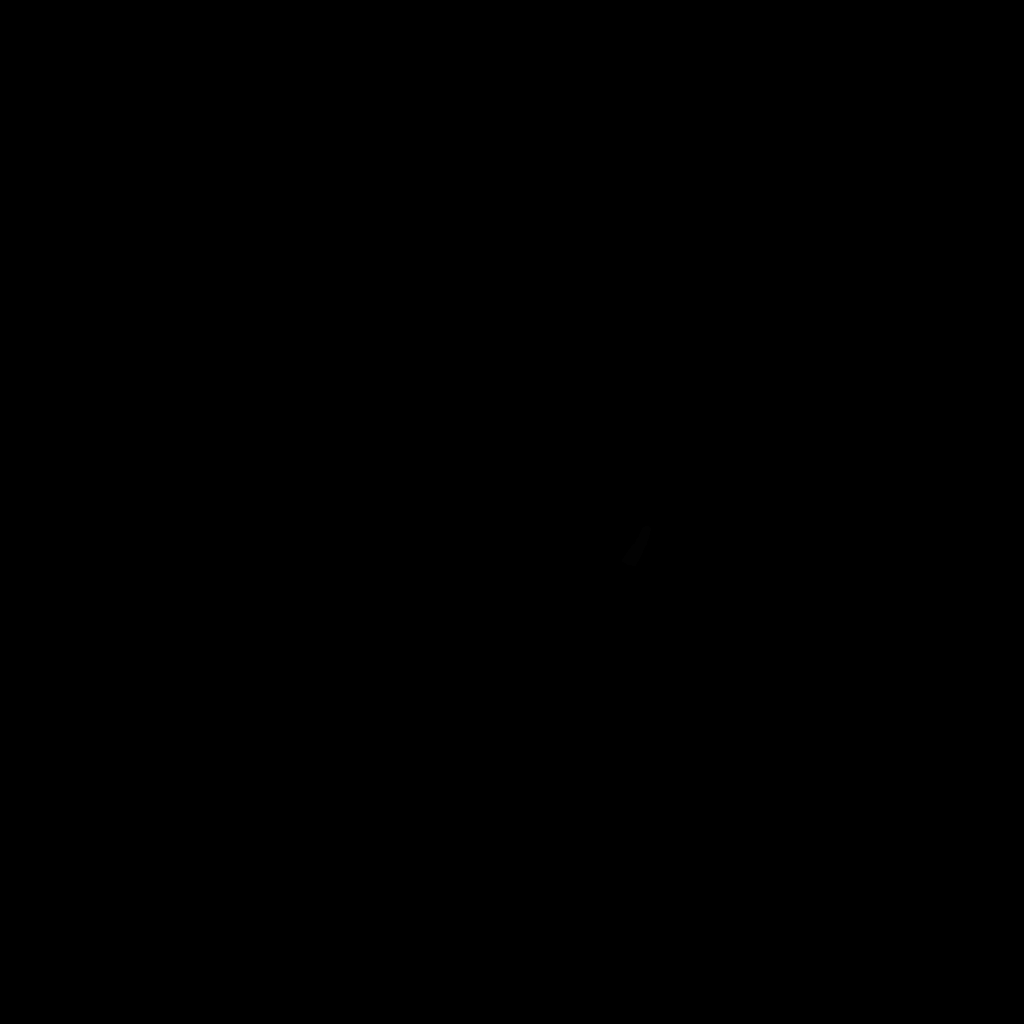

Supplement: Supplemental Information 1 [file peerj-cs-10-2097-s001.zip › IIT-AFF VL/masks/00_00000158.png]

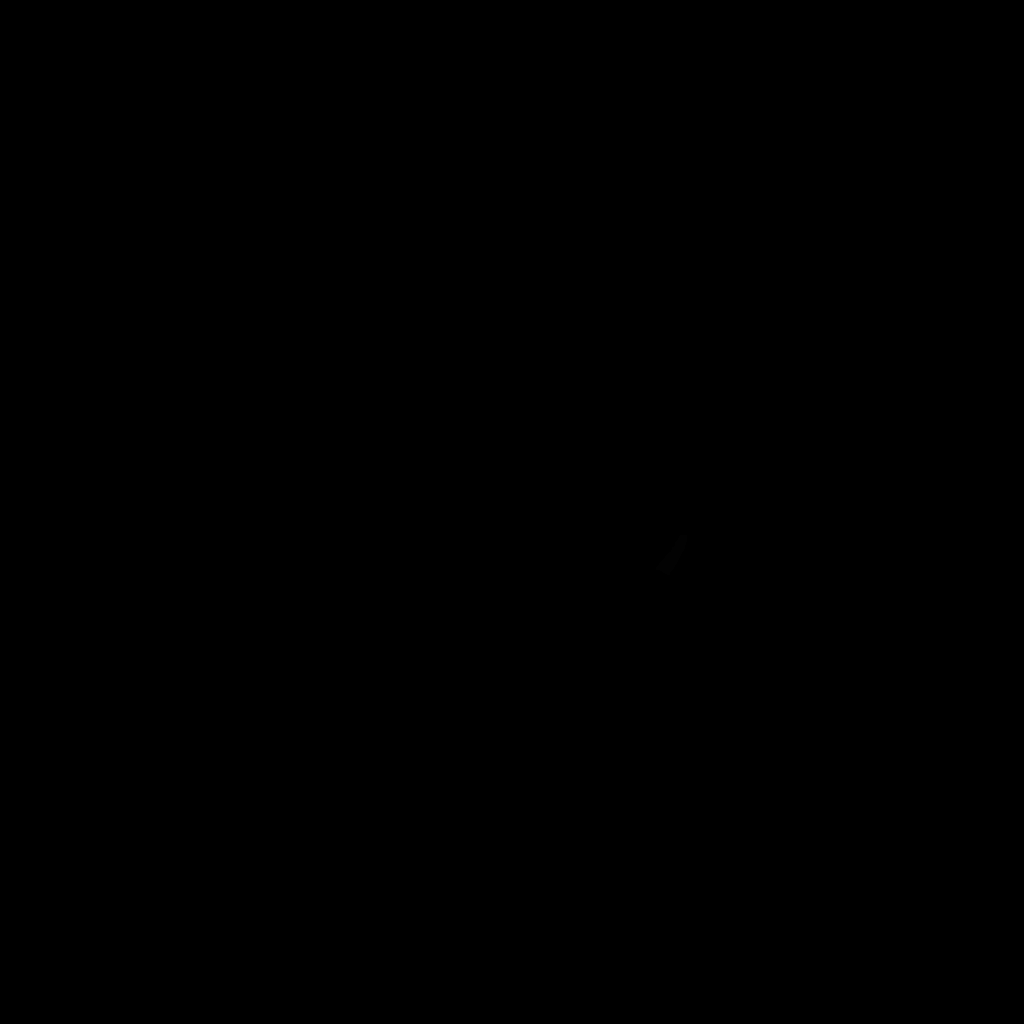

Supplement: Supplemental Information 1 [file peerj-cs-10-2097-s001.zip › IIT-AFF VL/masks/00_00000162.png]

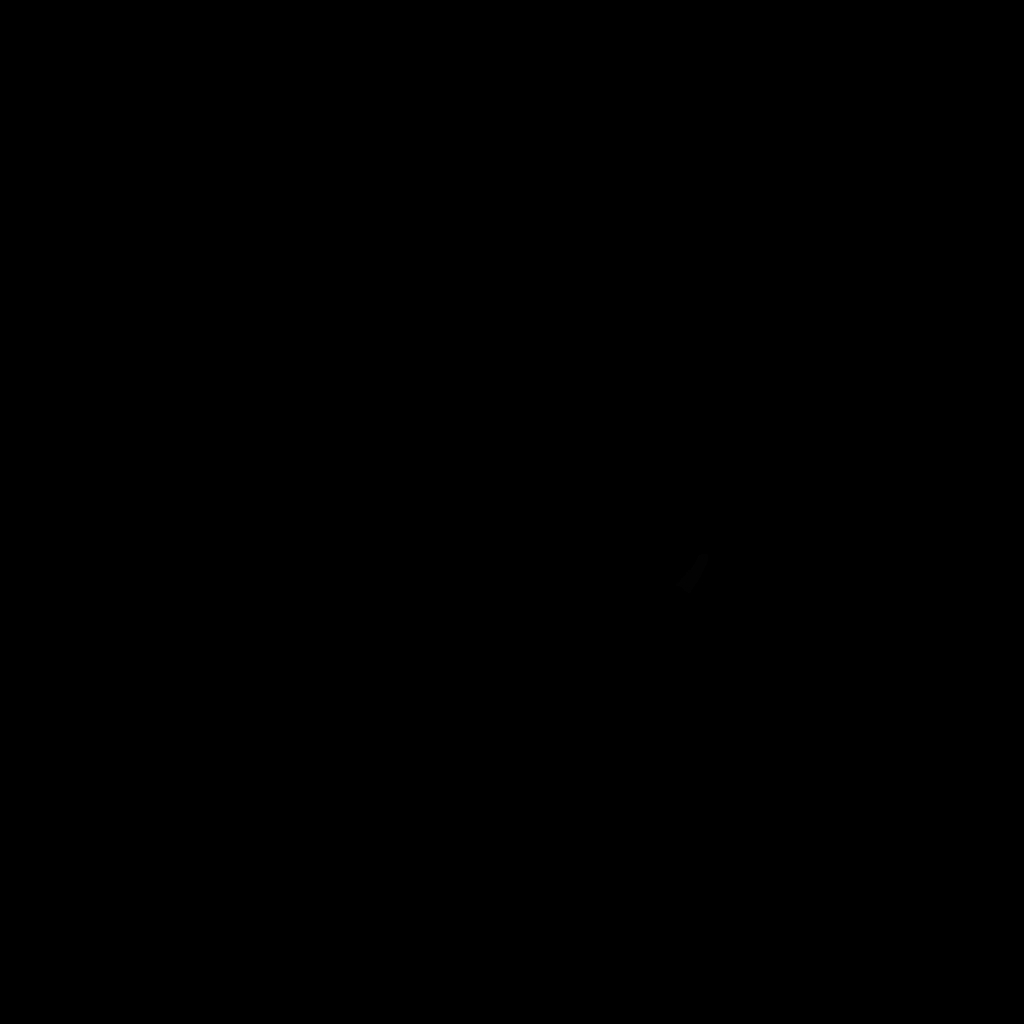

Supplement: Supplemental Information 1 [file peerj-cs-10-2097-s001.zip › IIT-AFF VL/masks/00_00000166.png]

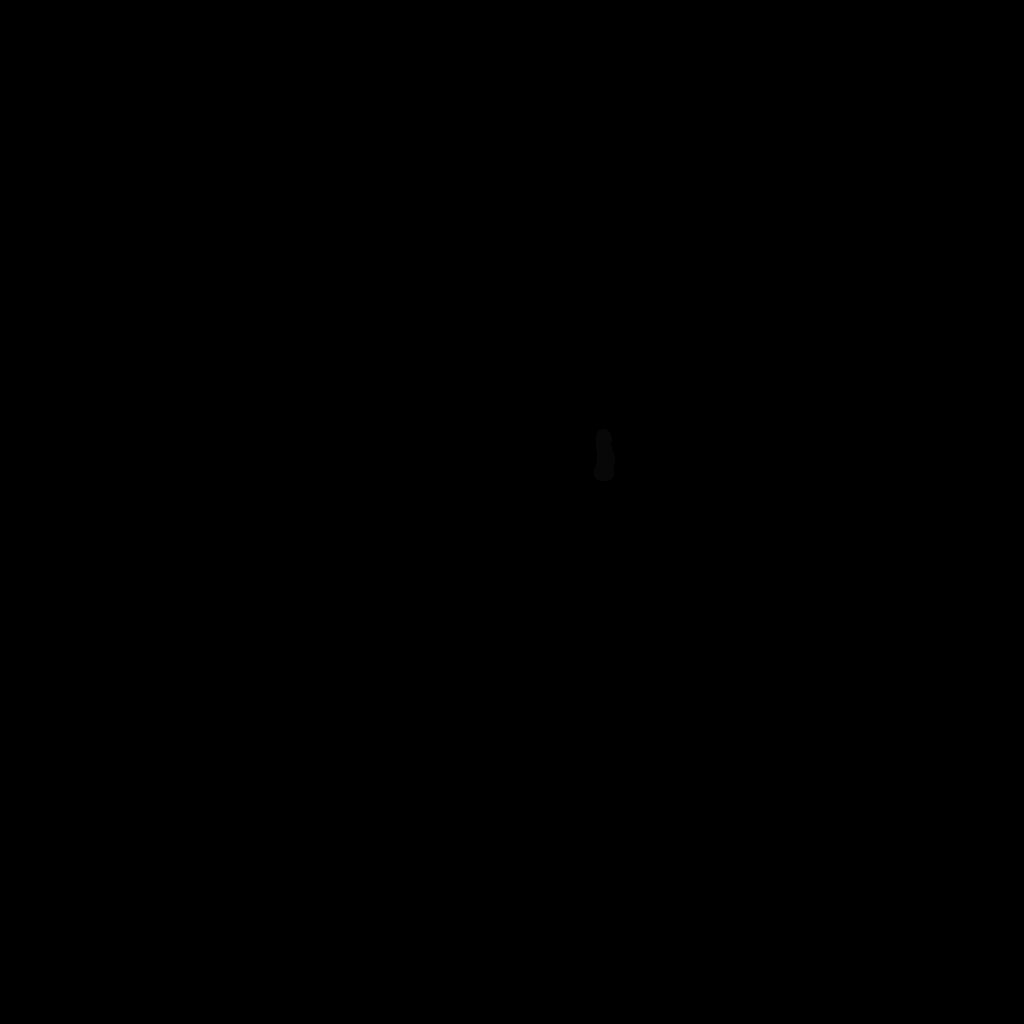

Supplement: Supplemental Information 1 [file peerj-cs-10-2097-s001.zip › IIT-AFF VL/masks/01_00000089.png]

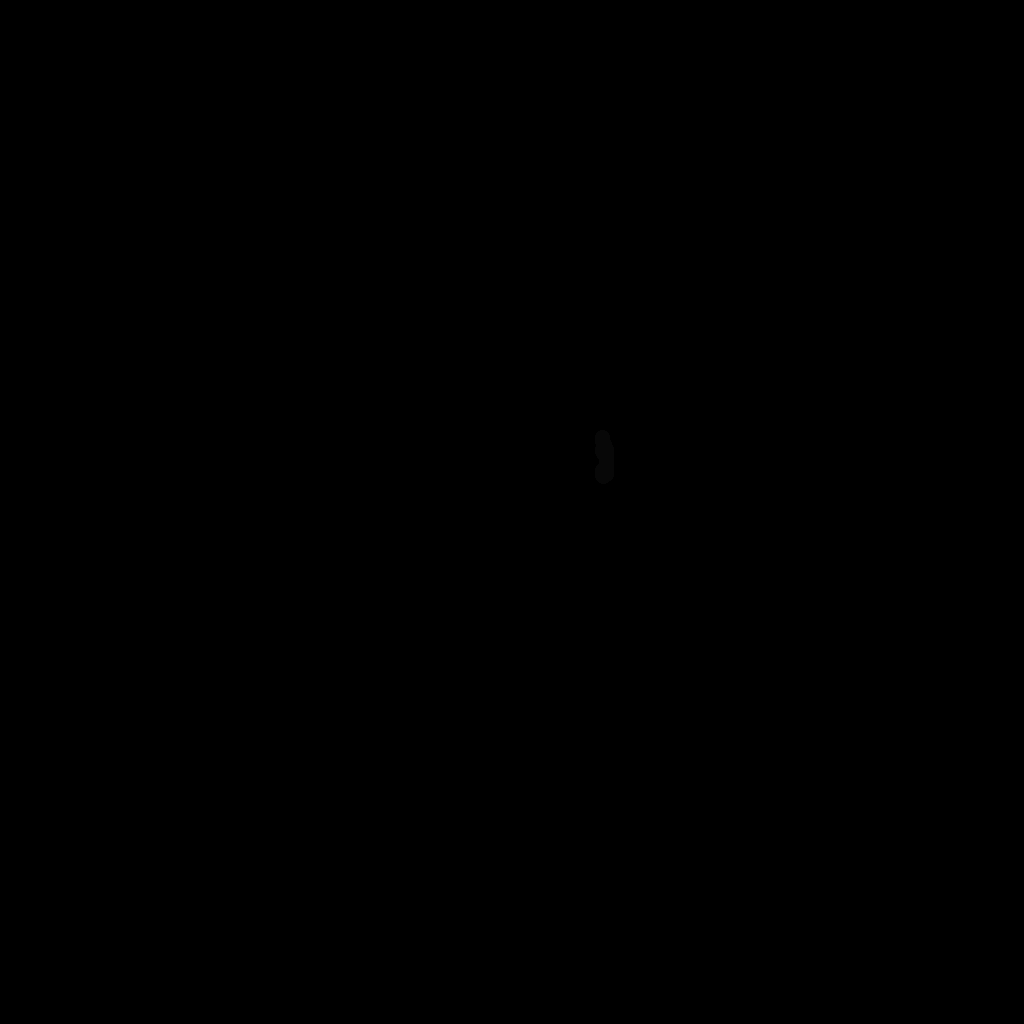

Supplement: Supplemental Information 1 [file peerj-cs-10-2097-s001.zip › IIT-AFF VL/masks/01_00000093.png]

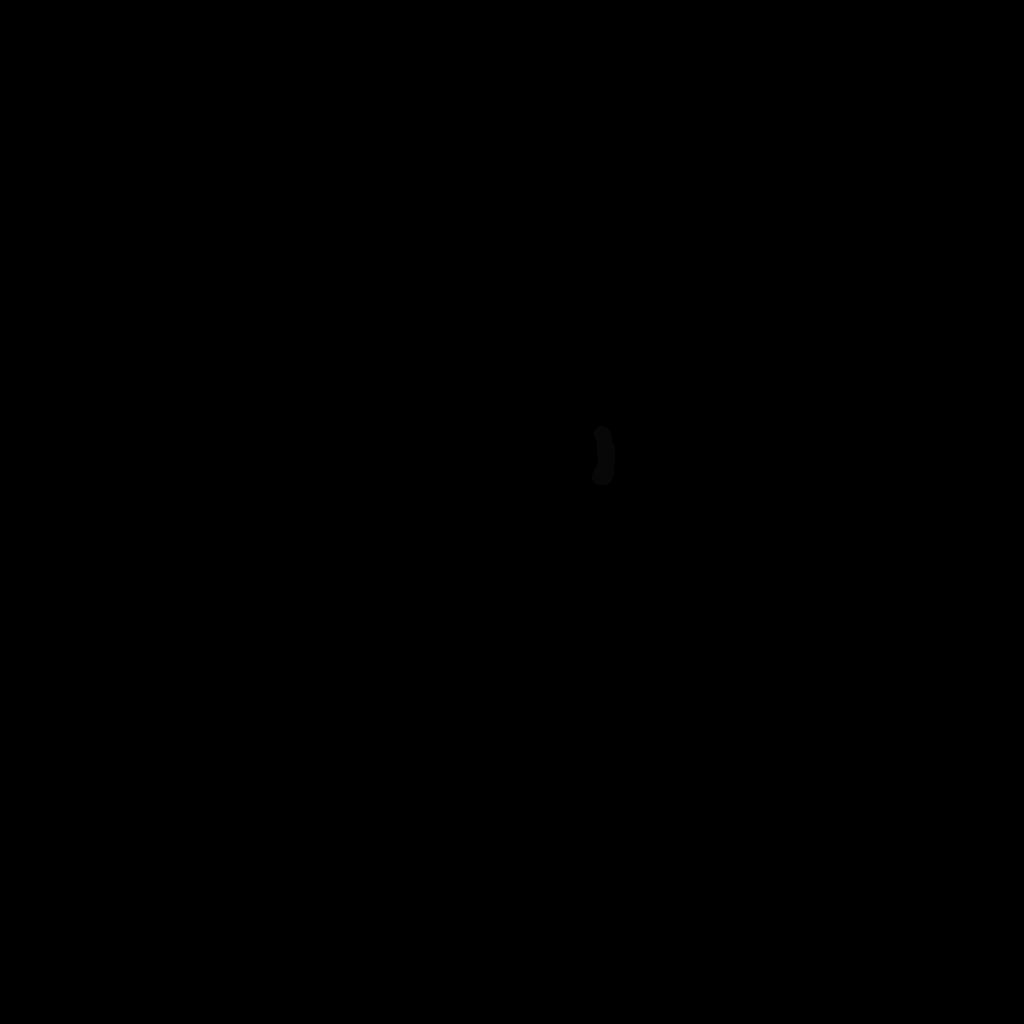

Supplement: Supplemental Information 1 [file peerj-cs-10-2097-s001.zip › IIT-AFF VL/masks/01_00000097.png]

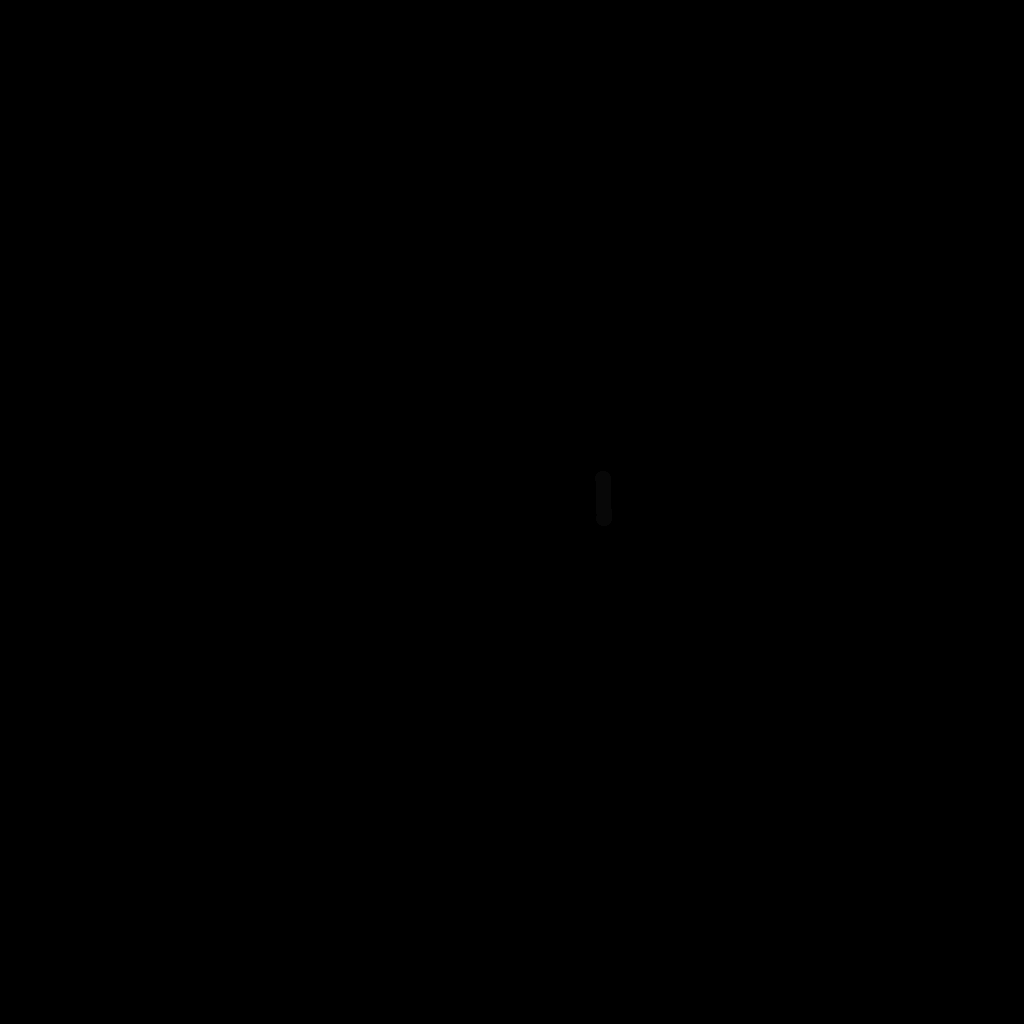

Supplement: Supplemental Information 1 [file peerj-cs-10-2097-s001.zip › IIT-AFF VL/masks/01_00000105.png]

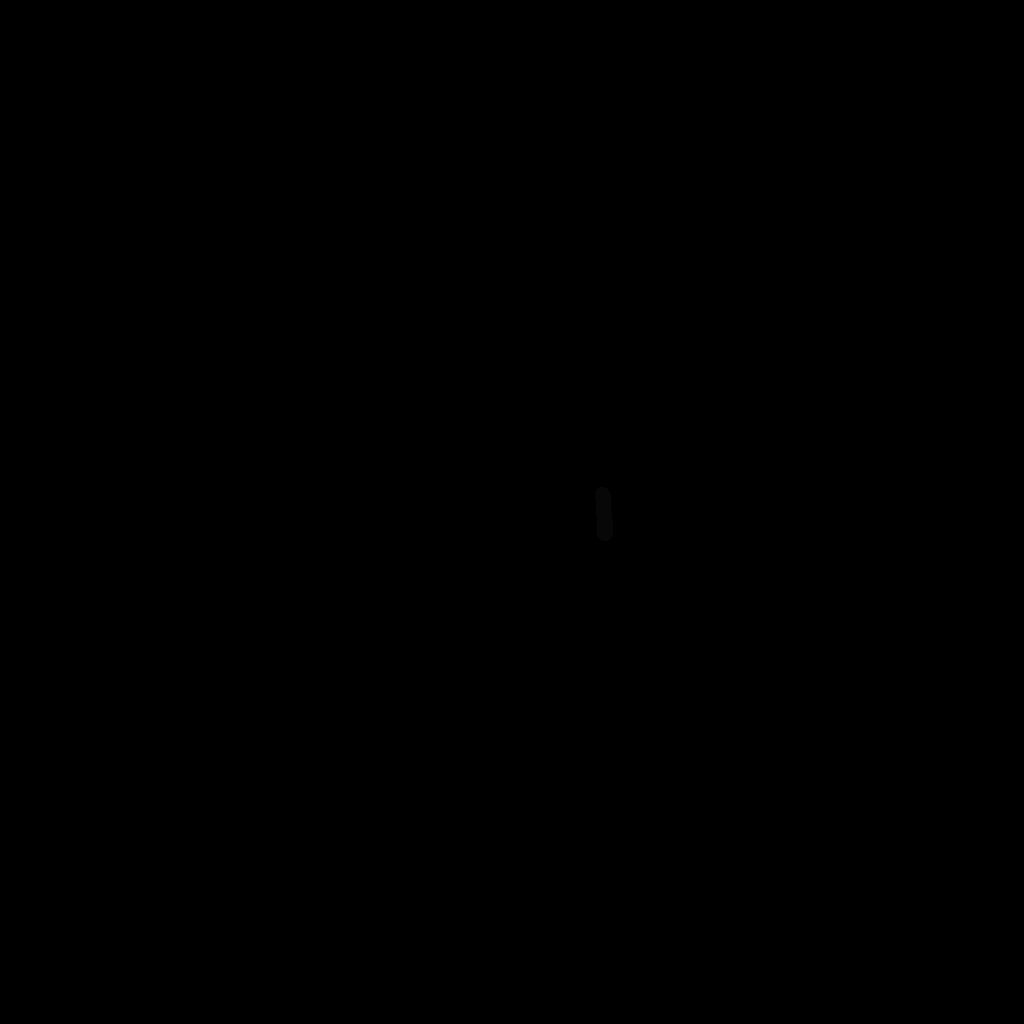

Supplement: Supplemental Information 1 [file peerj-cs-10-2097-s001.zip › IIT-AFF VL/masks/01_00000109.png]

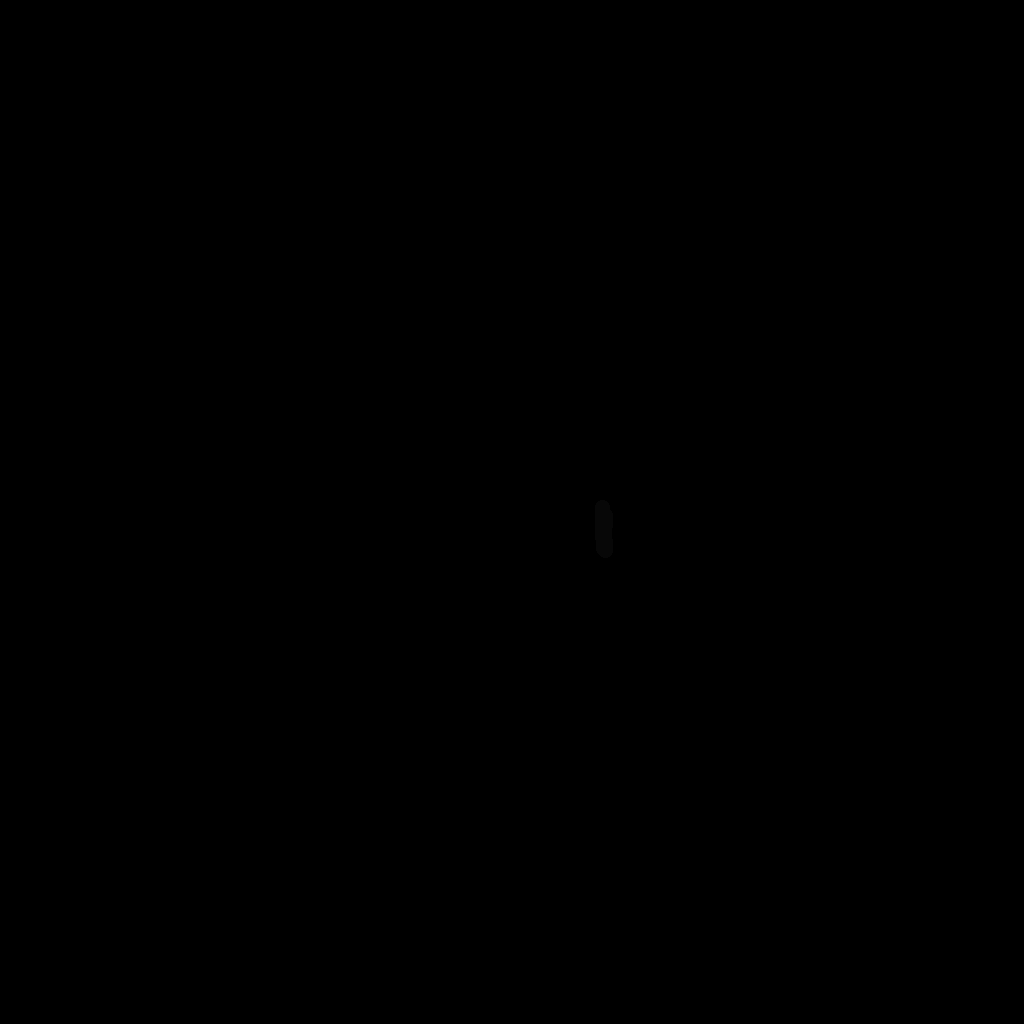

Supplement: Supplemental Information 1 [file peerj-cs-10-2097-s001.zip › IIT-AFF VL/masks/01_00000113.png]

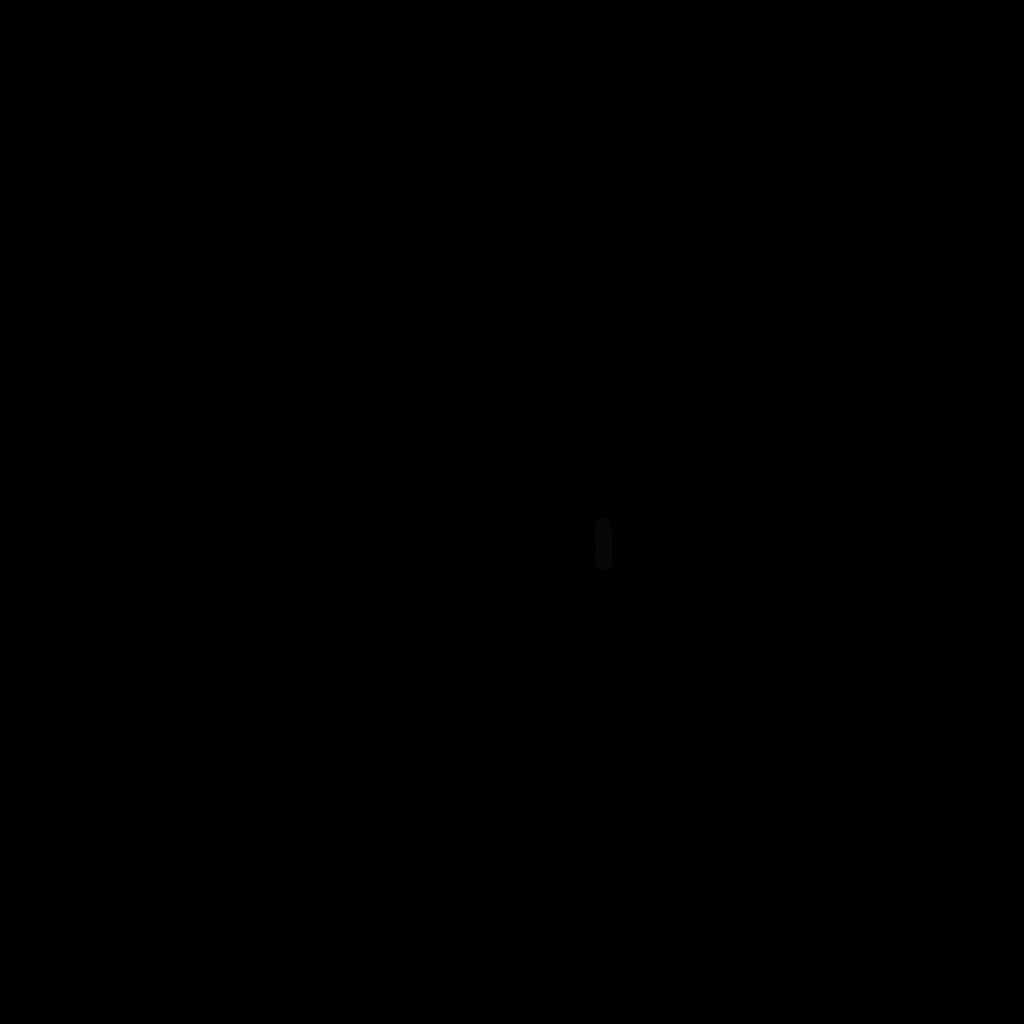

Supplement: Supplemental Information 1 [file peerj-cs-10-2097-s001.zip › IIT-AFF VL/masks/01_00000117.png]

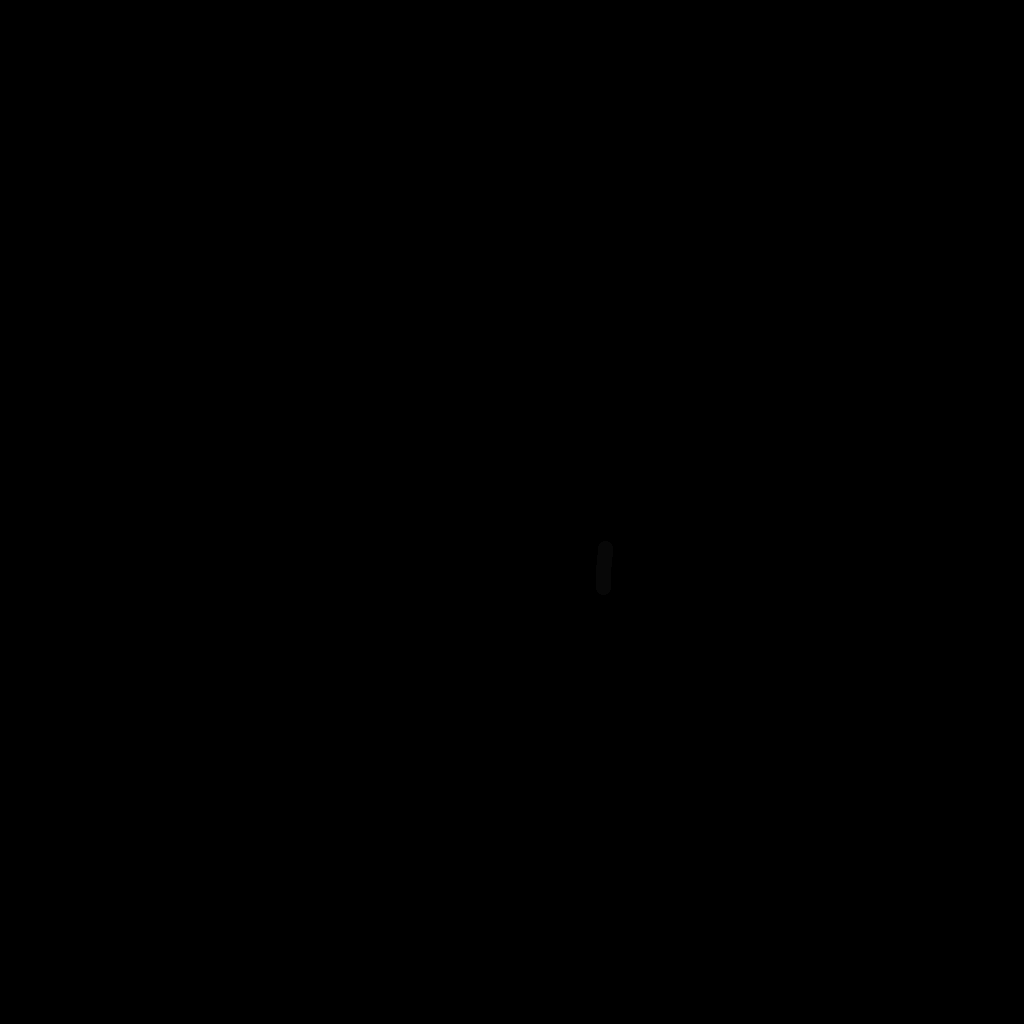

Supplement: Supplemental Information 1 [file peerj-cs-10-2097-s001.zip › IIT-AFF VL/masks/01_00000121.png]

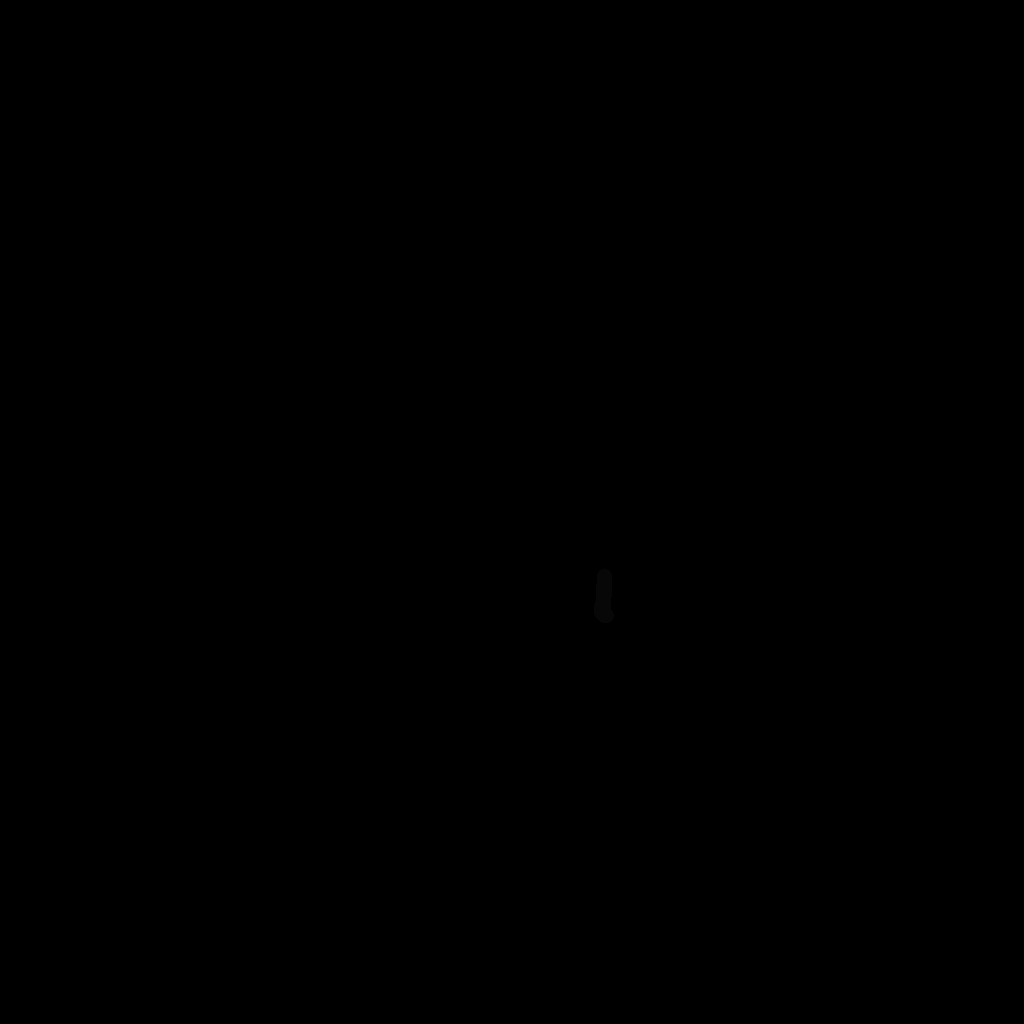

Supplement: Supplemental Information 1 [file peerj-cs-10-2097-s001.zip › IIT-AFF VL/masks/01_00000125.png]

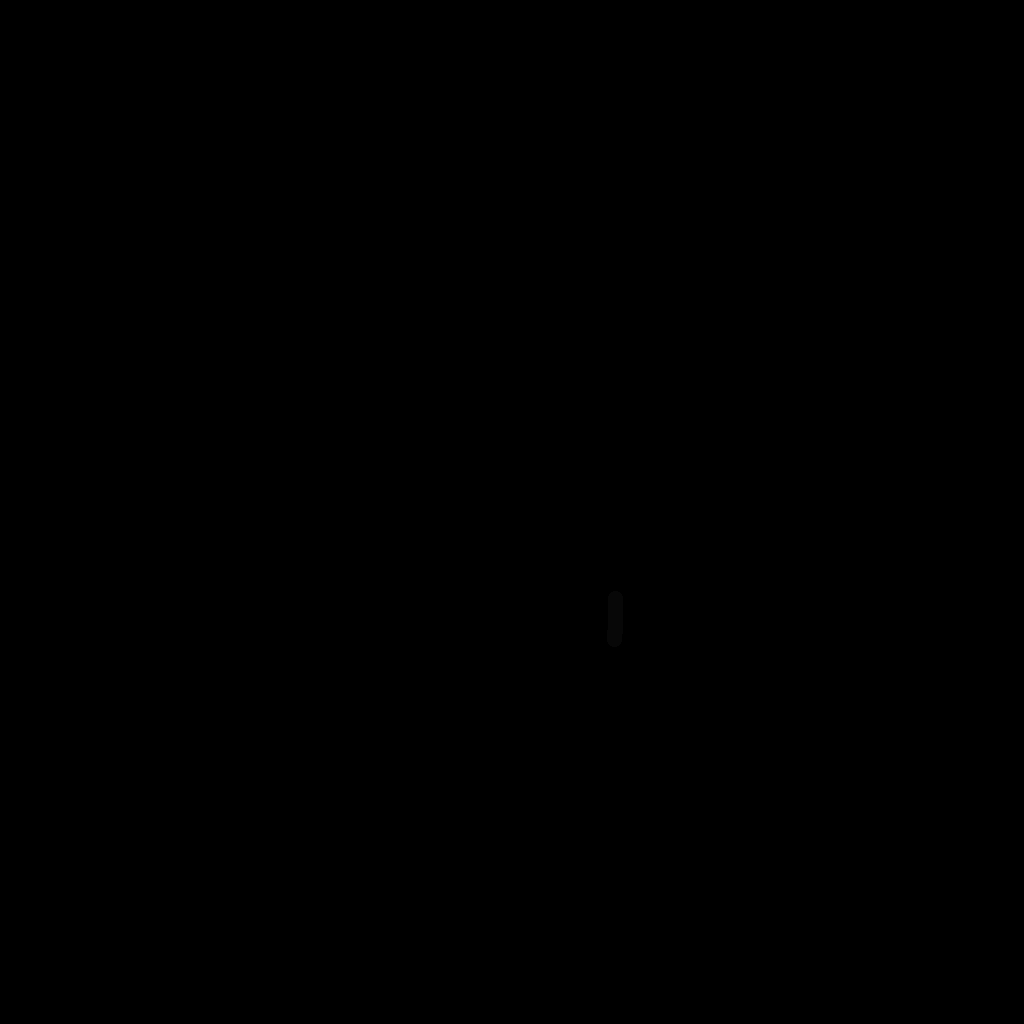

Supplement: Supplemental Information 1 [file peerj-cs-10-2097-s001.zip › IIT-AFF VL/masks/01_00000129.png]

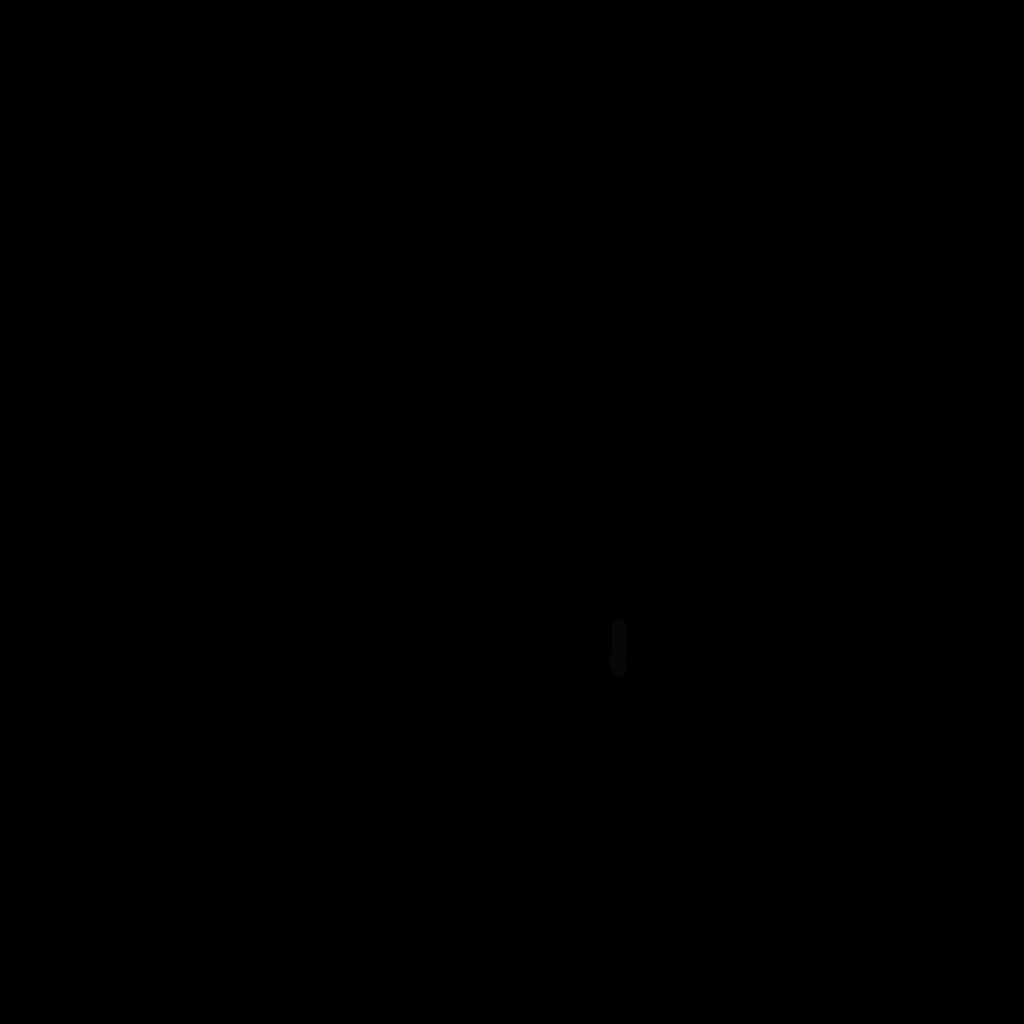

Supplement: Supplemental Information 1 [file peerj-cs-10-2097-s001.zip › IIT-AFF VL/masks/01_00000133.png]

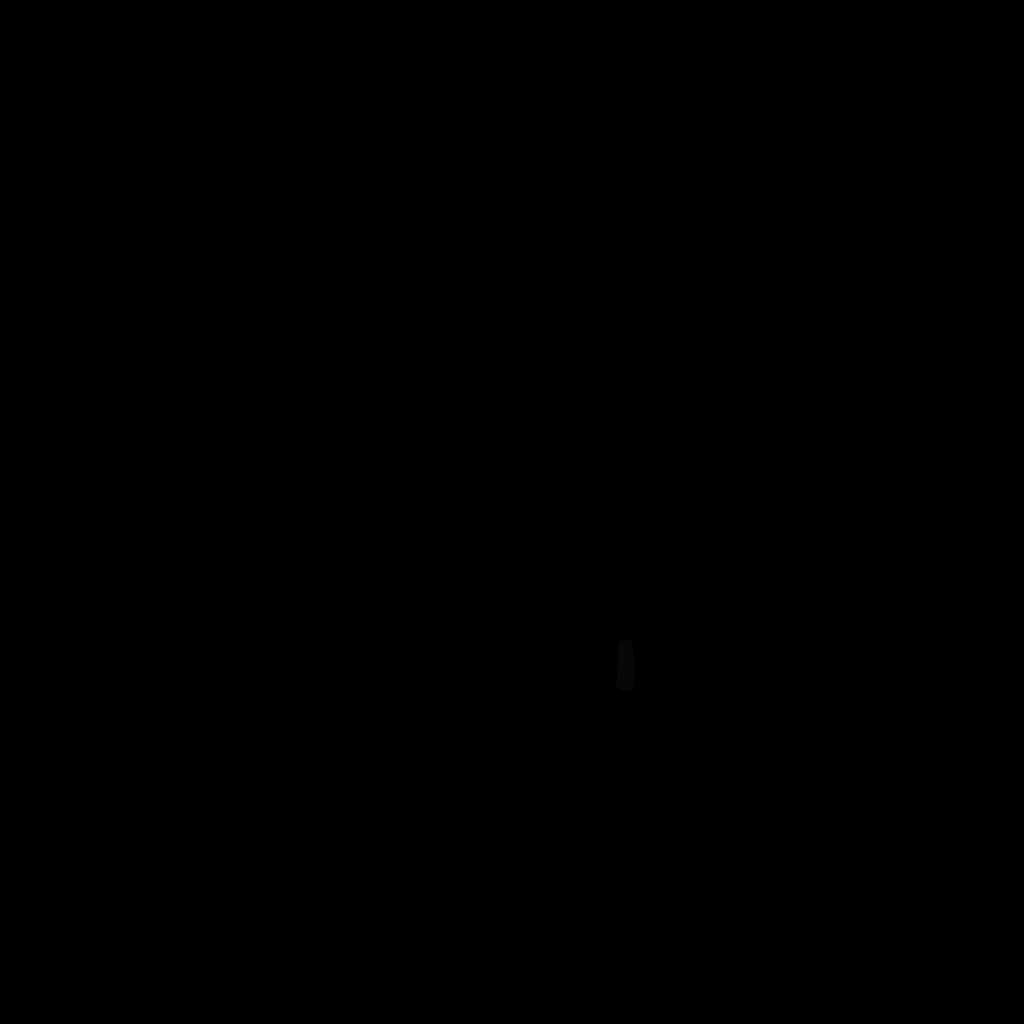

Supplement: Supplemental Information 1 [file peerj-cs-10-2097-s001.zip › IIT-AFF VL/masks/01_00000137.png]

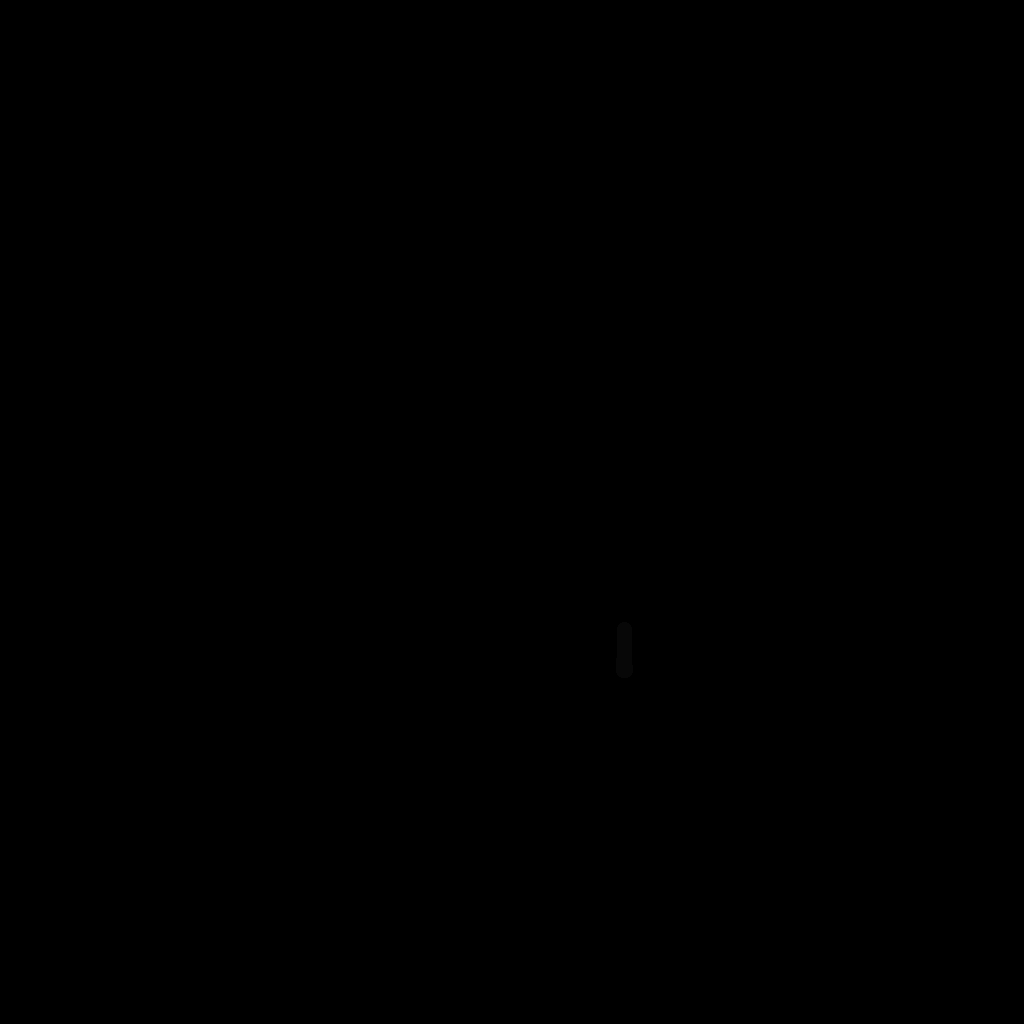

Supplement: Supplemental Information 1 [file peerj-cs-10-2097-s001.zip › IIT-AFF VL/masks/01_00000141.png]

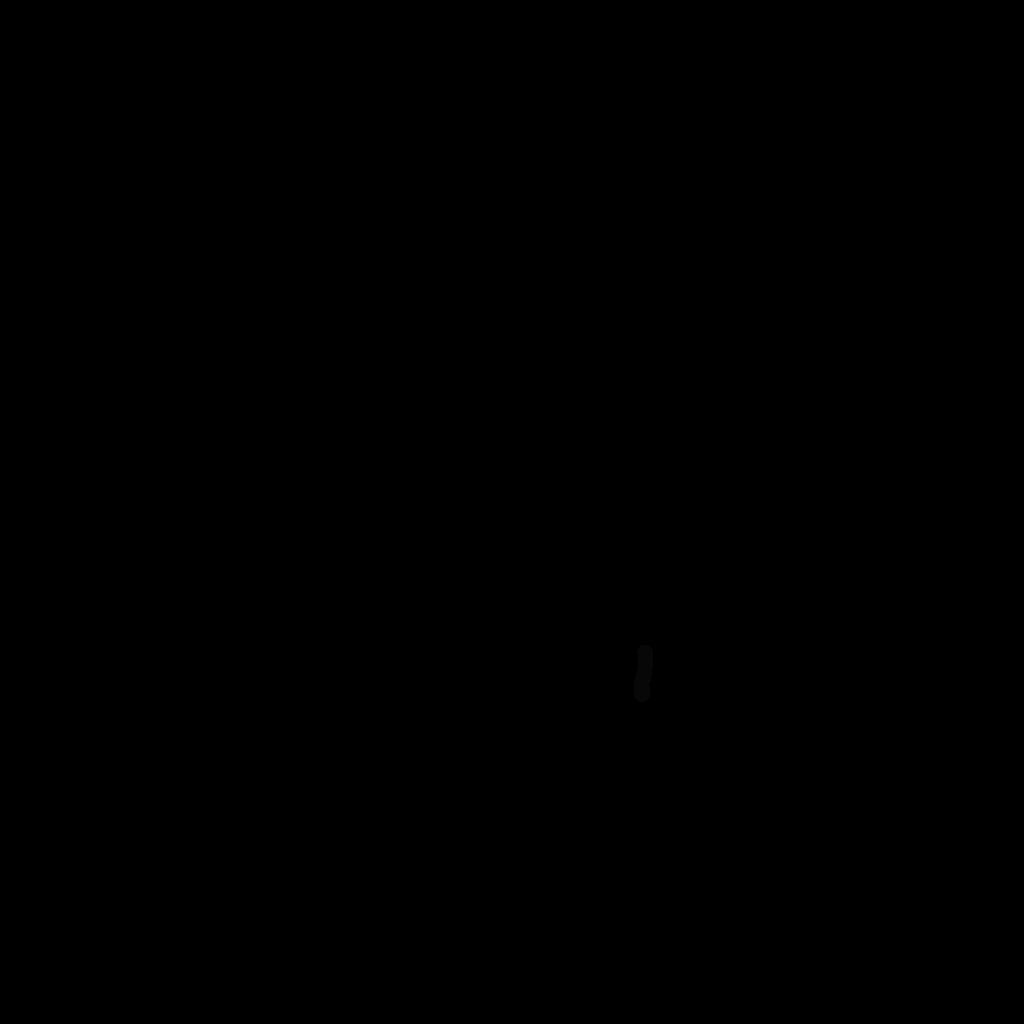

Supplement: Supplemental Information 1 [file peerj-cs-10-2097-s001.zip › IIT-AFF VL/masks/01_00000145.png]

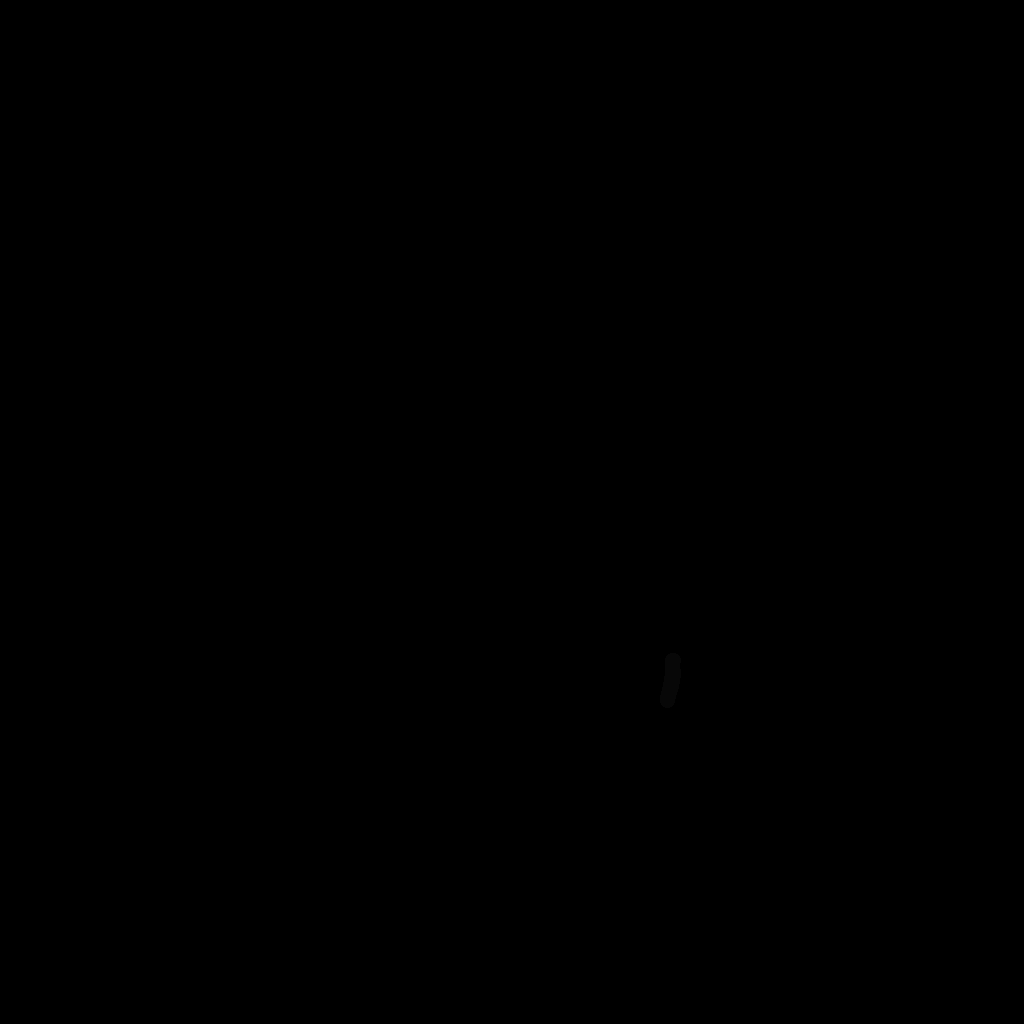

Supplement: Supplemental Information 1 [file peerj-cs-10-2097-s001.zip › IIT-AFF VL/masks/01_00000149.png]

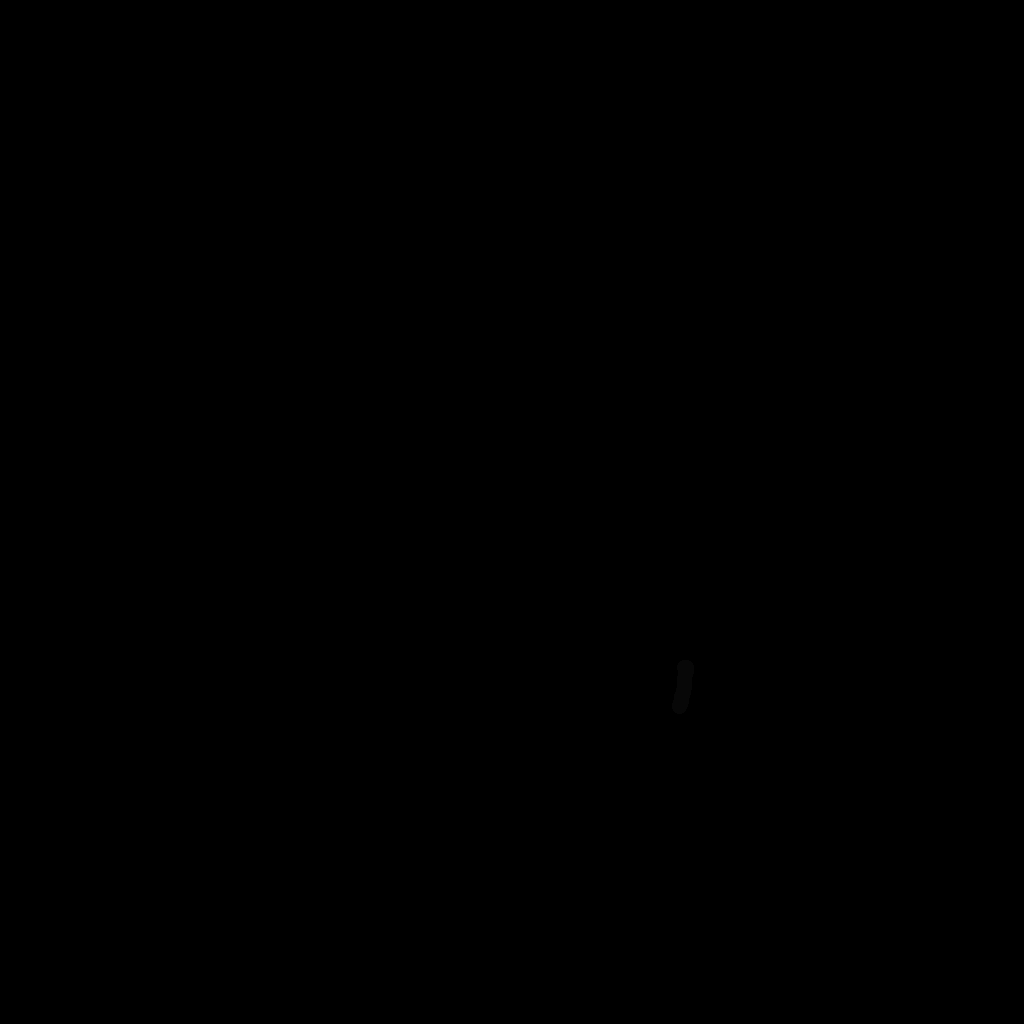

Supplement: Supplemental Information 1 [file peerj-cs-10-2097-s001.zip › IIT-AFF VL/masks/01_00000153.png]

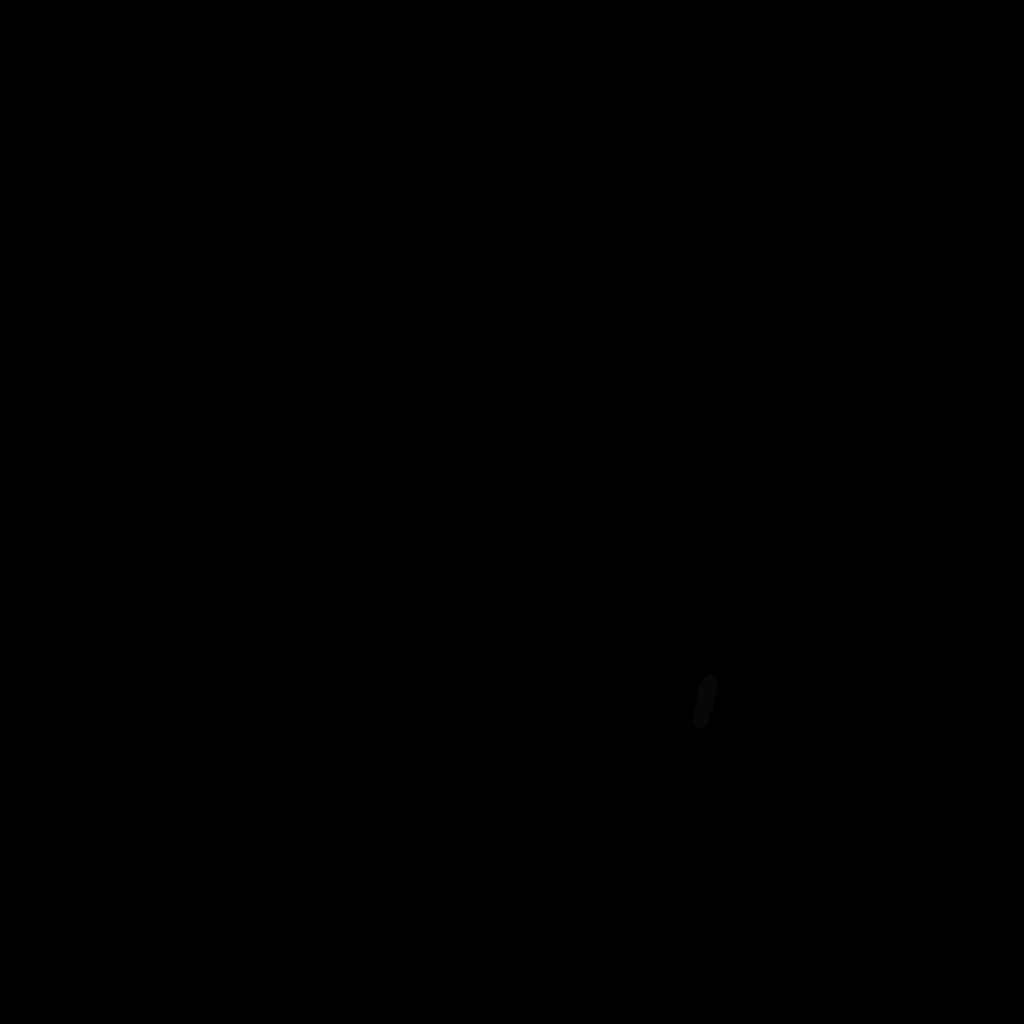

Supplement: Supplemental Information 1 [file peerj-cs-10-2097-s001.zip › IIT-AFF VL/masks/01_00000157.png]

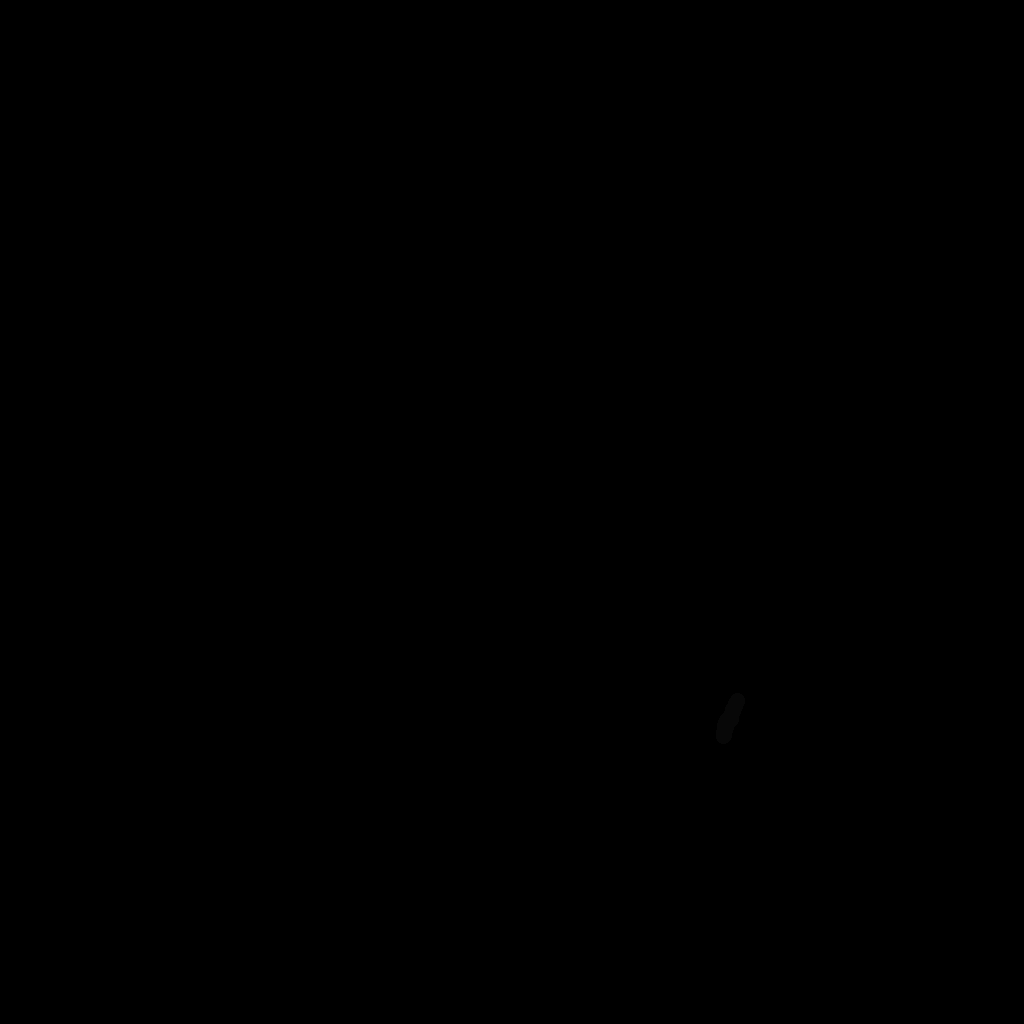

Supplement: Supplemental Information 1 [file peerj-cs-10-2097-s001.zip › IIT-AFF VL/masks/01_00000161.png]

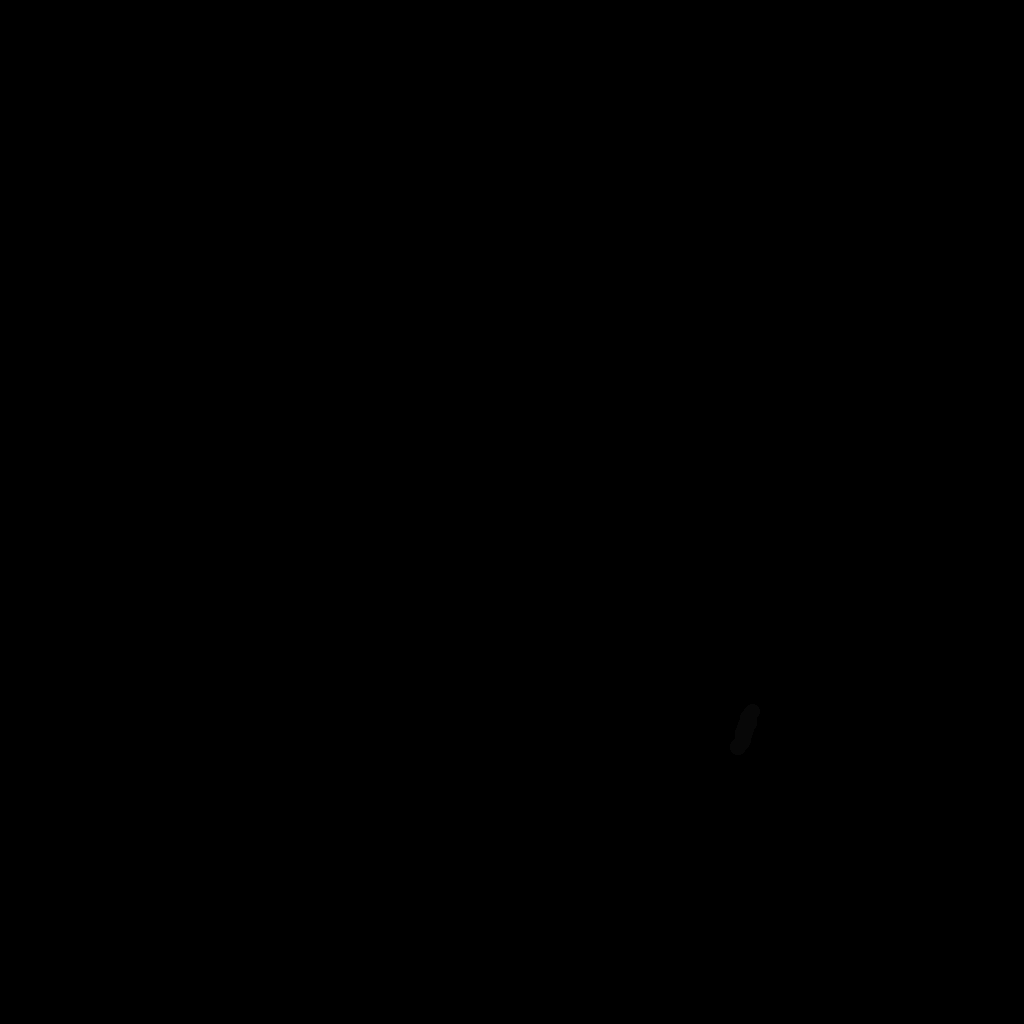

Supplement: Supplemental Information 1 [file peerj-cs-10-2097-s001.zip › IIT-AFF VL/masks/01_00000165.png]

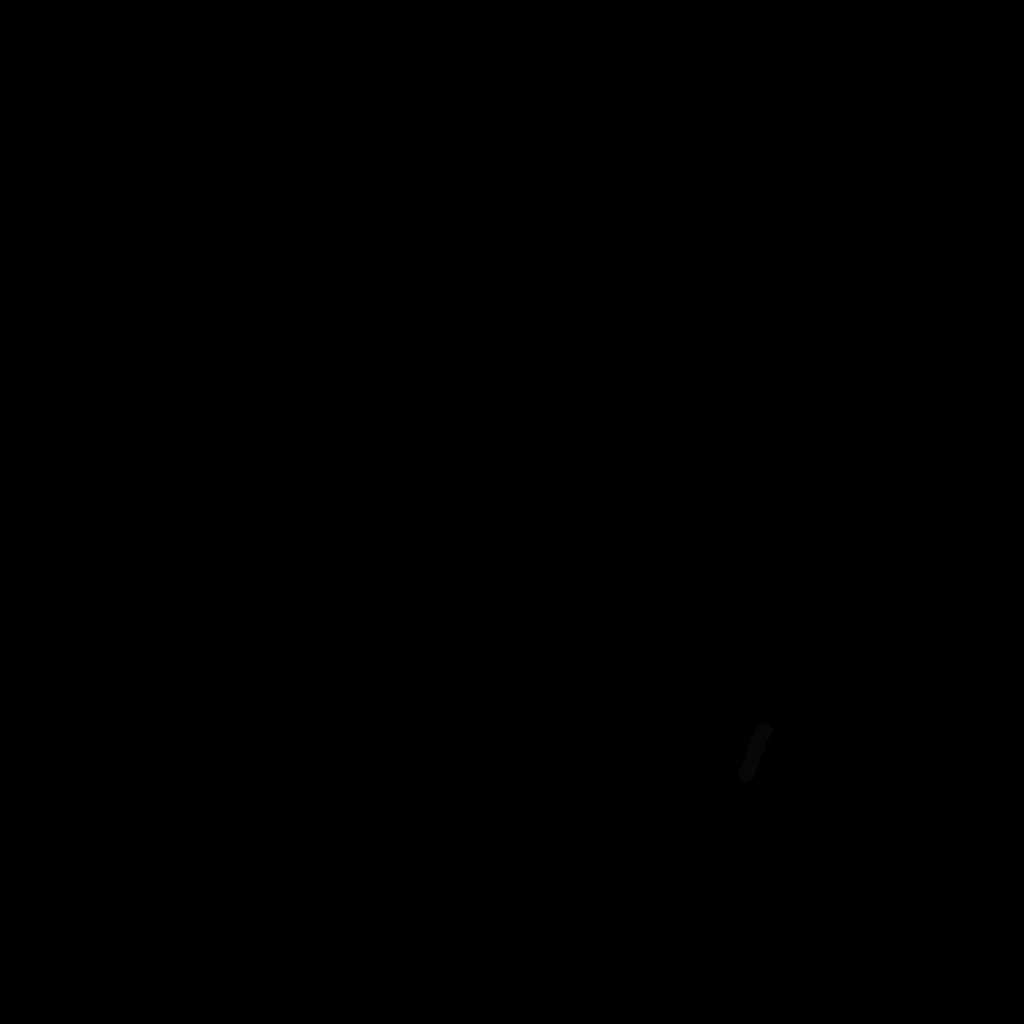

Supplement: Supplemental Information 1 [file peerj-cs-10-2097-s001.zip › IIT-AFF VL/masks/01_00000169.png]

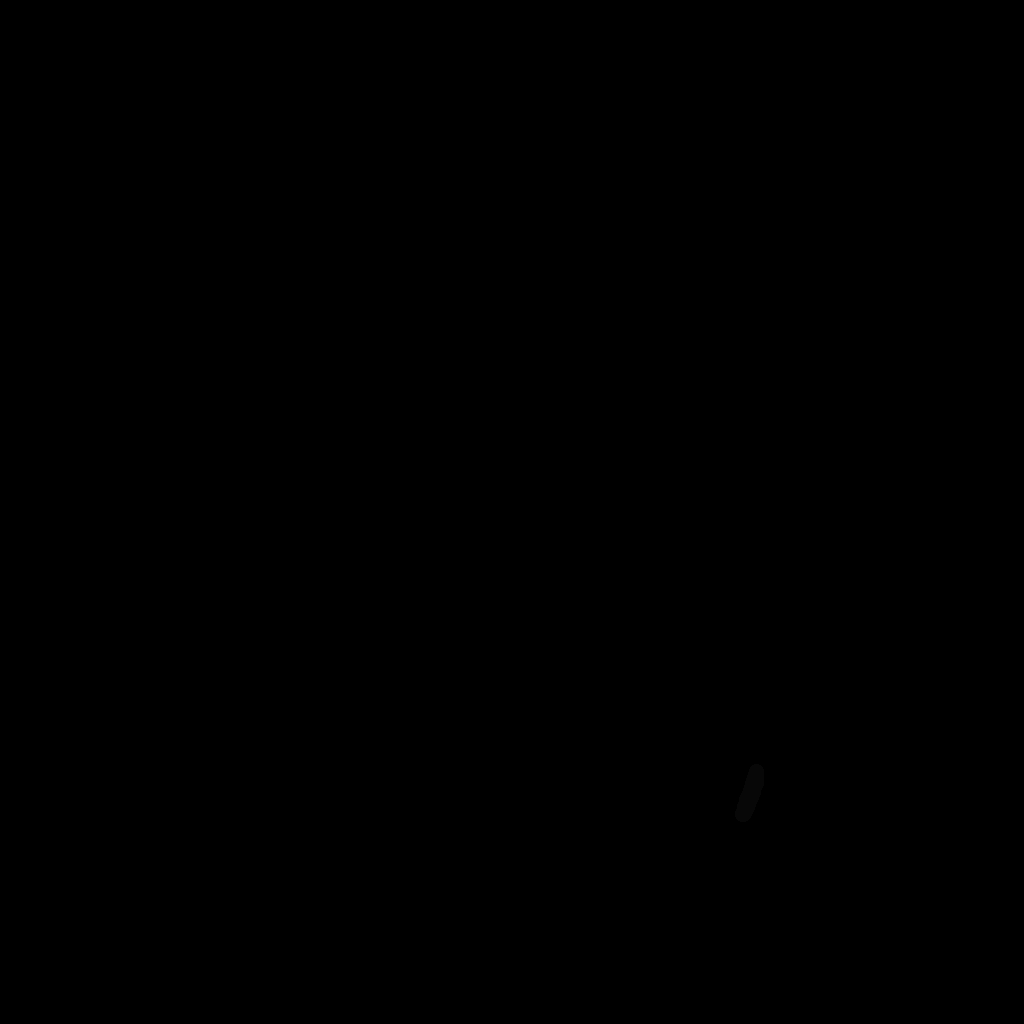

Supplement: Supplemental Information 1 [file peerj-cs-10-2097-s001.zip › IIT-AFF VL/masks/01_00000173.png]

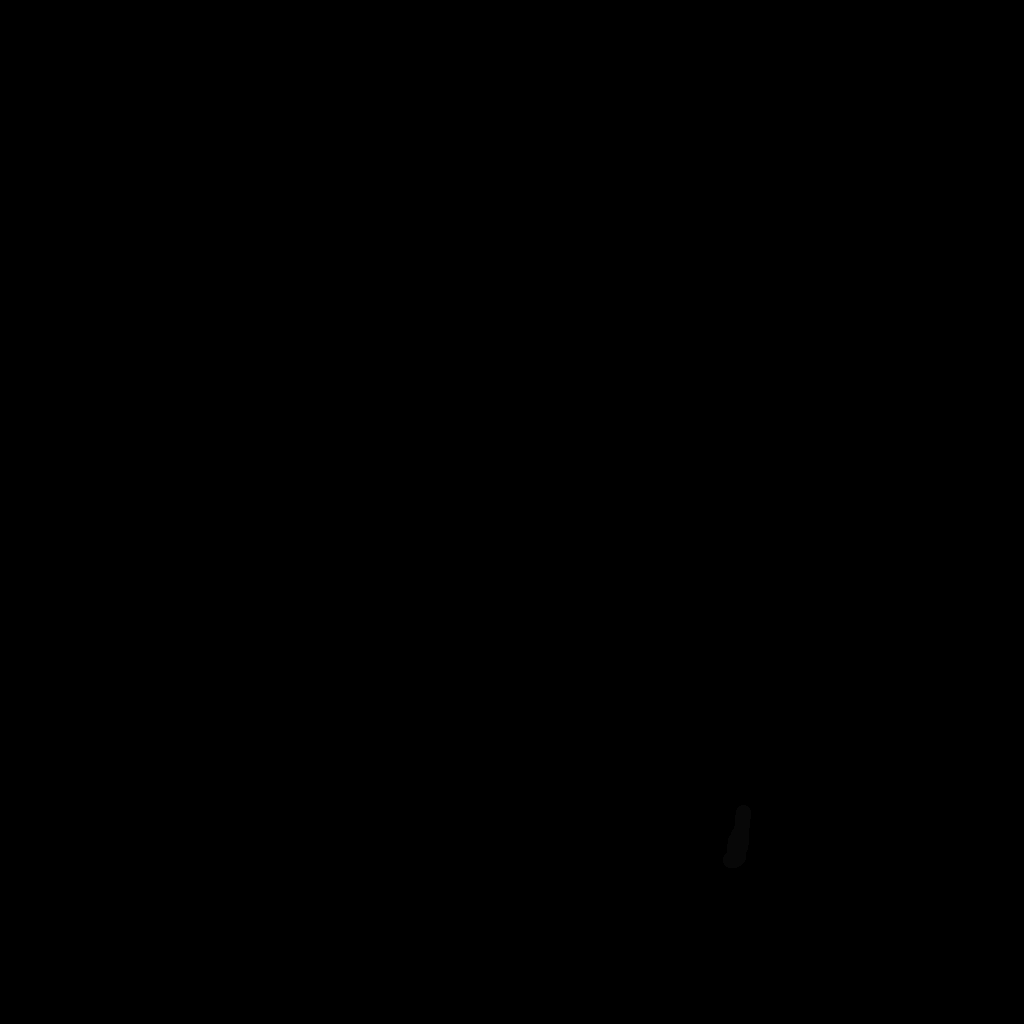

Supplement: Supplemental Information 1 [file peerj-cs-10-2097-s001.zip › IIT-AFF VL/masks/01_00000177.png]

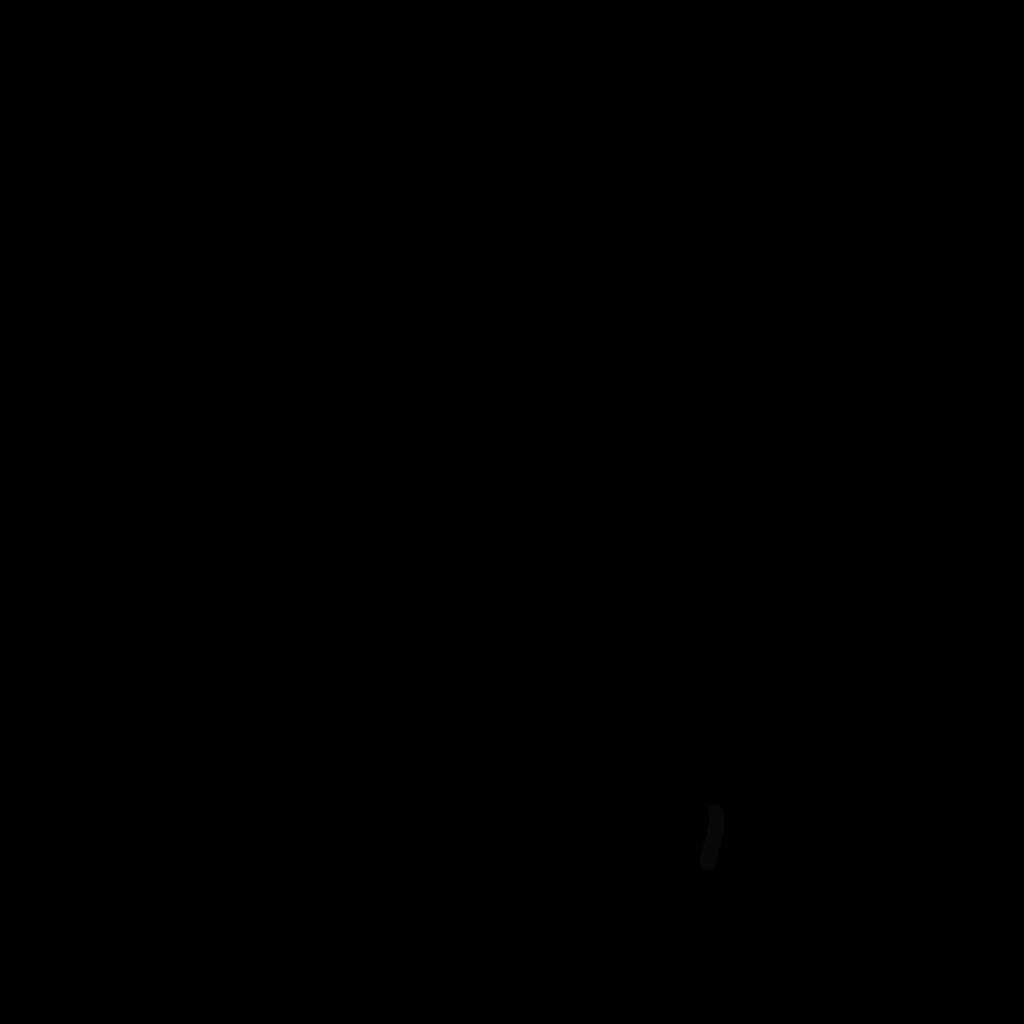

Supplement: Supplemental Information 1 [file peerj-cs-10-2097-s001.zip › IIT-AFF VL/masks/01_00000181.png]

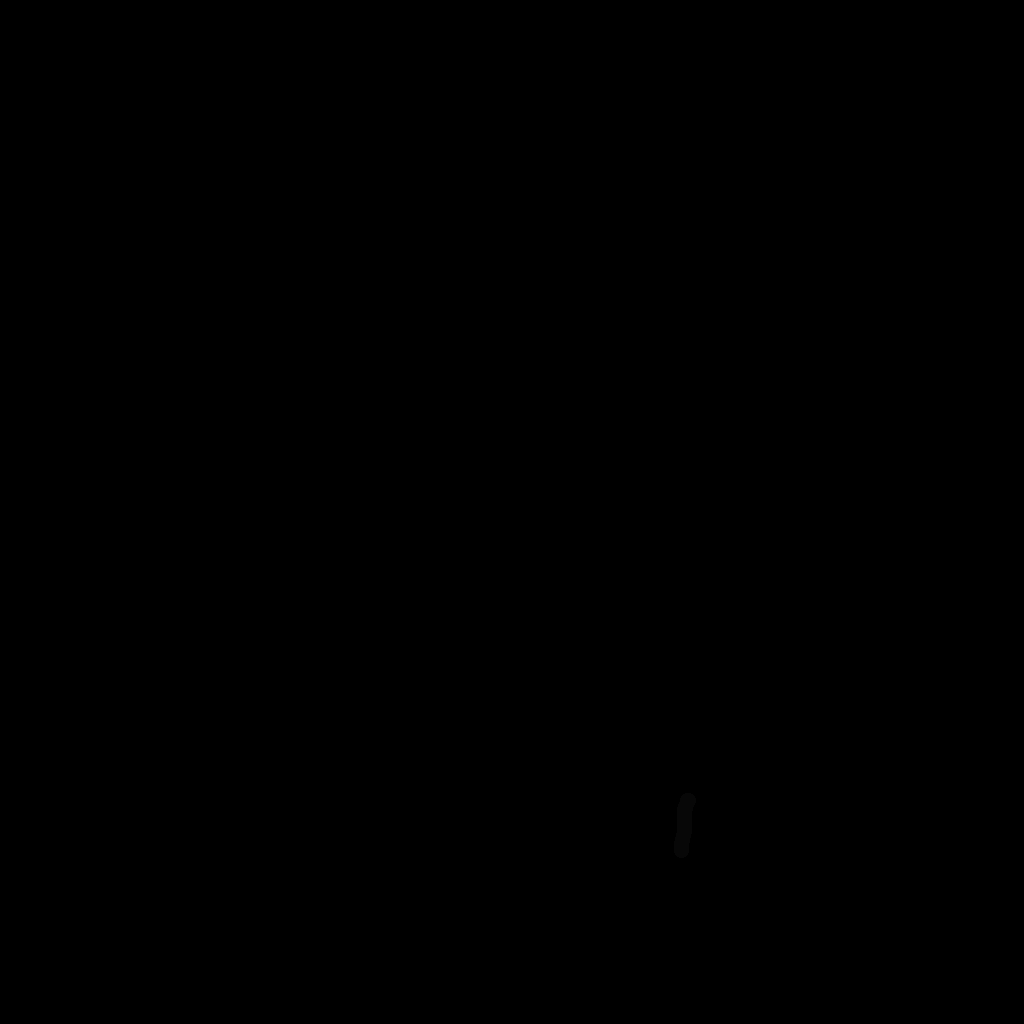

Supplement: Supplemental Information 1 [file peerj-cs-10-2097-s001.zip › IIT-AFF VL/masks/01_00000185.png]

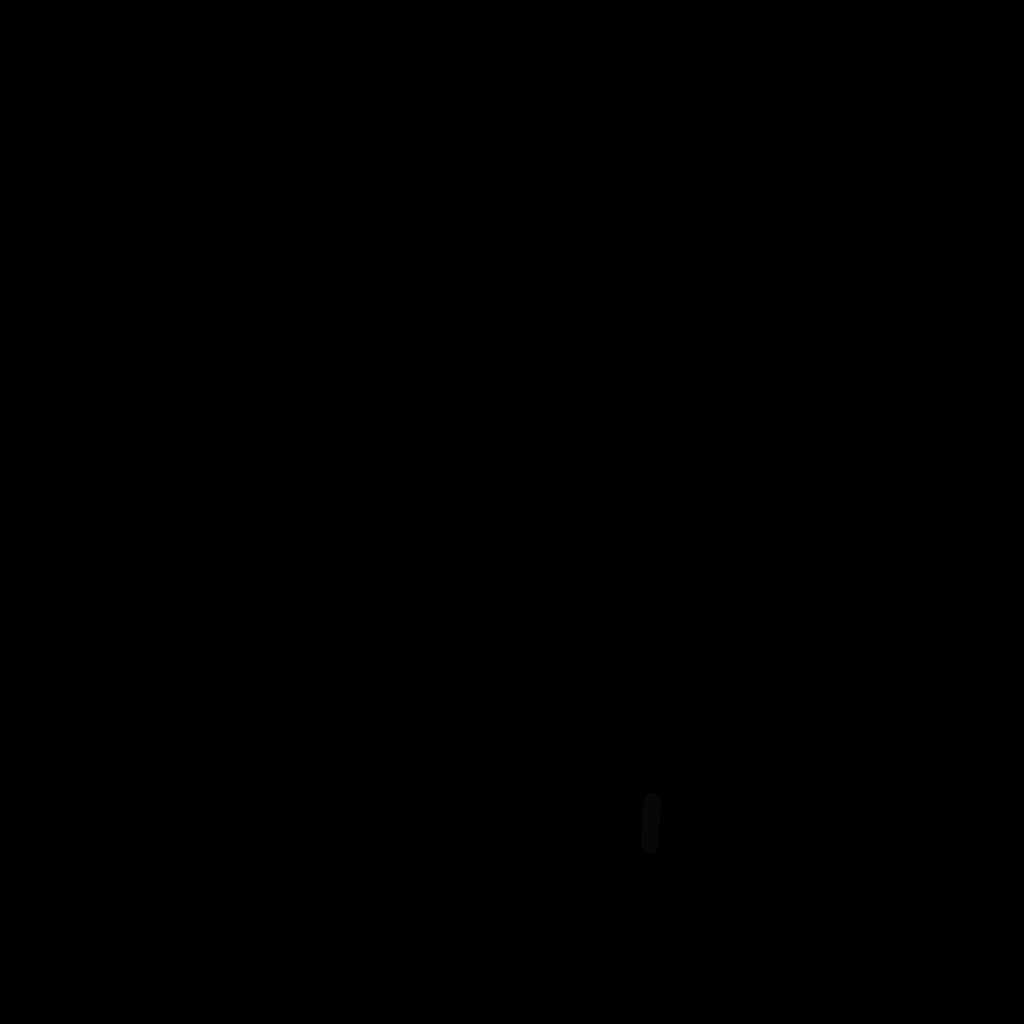

Supplement: Supplemental Information 1 [file peerj-cs-10-2097-s001.zip › IIT-AFF VL/masks/01_00000189.png]

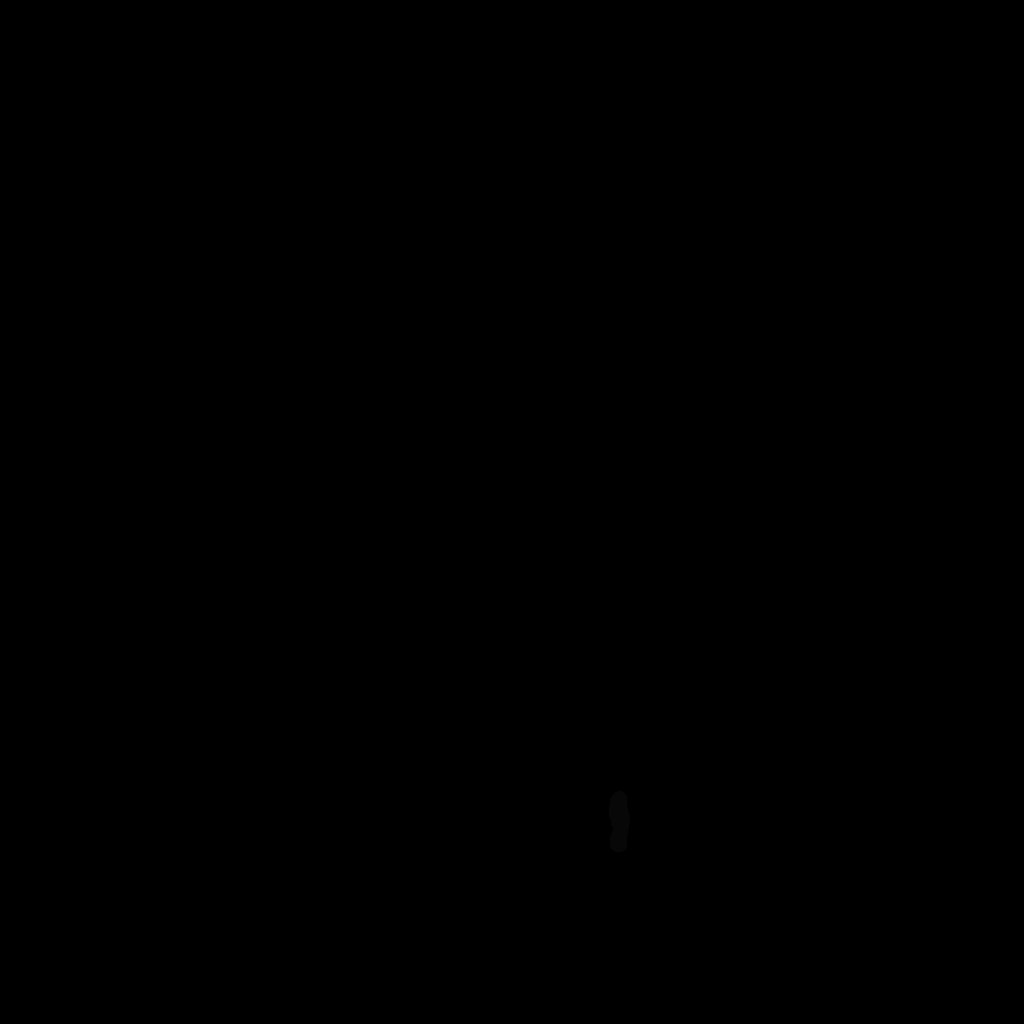

Supplement: Supplemental Information 1 [file peerj-cs-10-2097-s001.zip › IIT-AFF VL/masks/01_00000193.png]

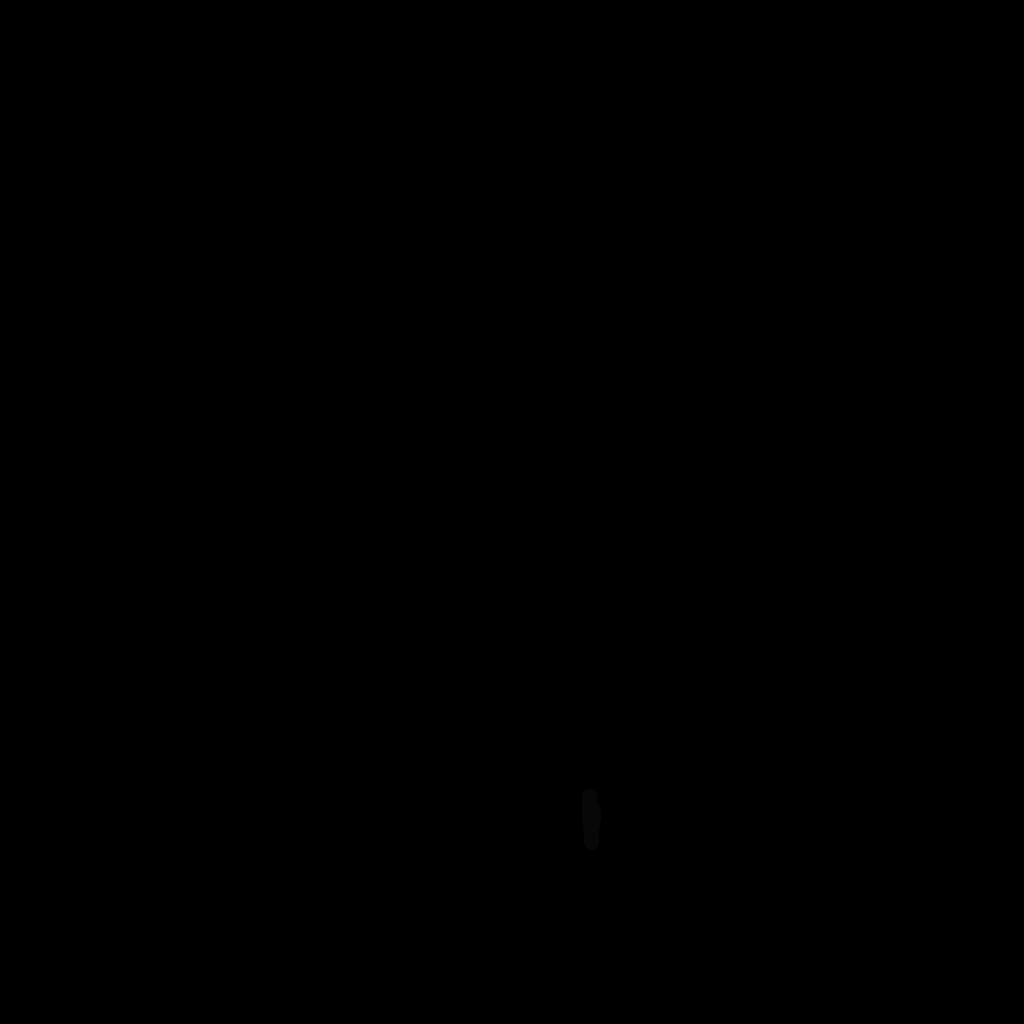

Supplement: Supplemental Information 1 [file peerj-cs-10-2097-s001.zip › IIT-AFF VL/masks/01_00000197.png]

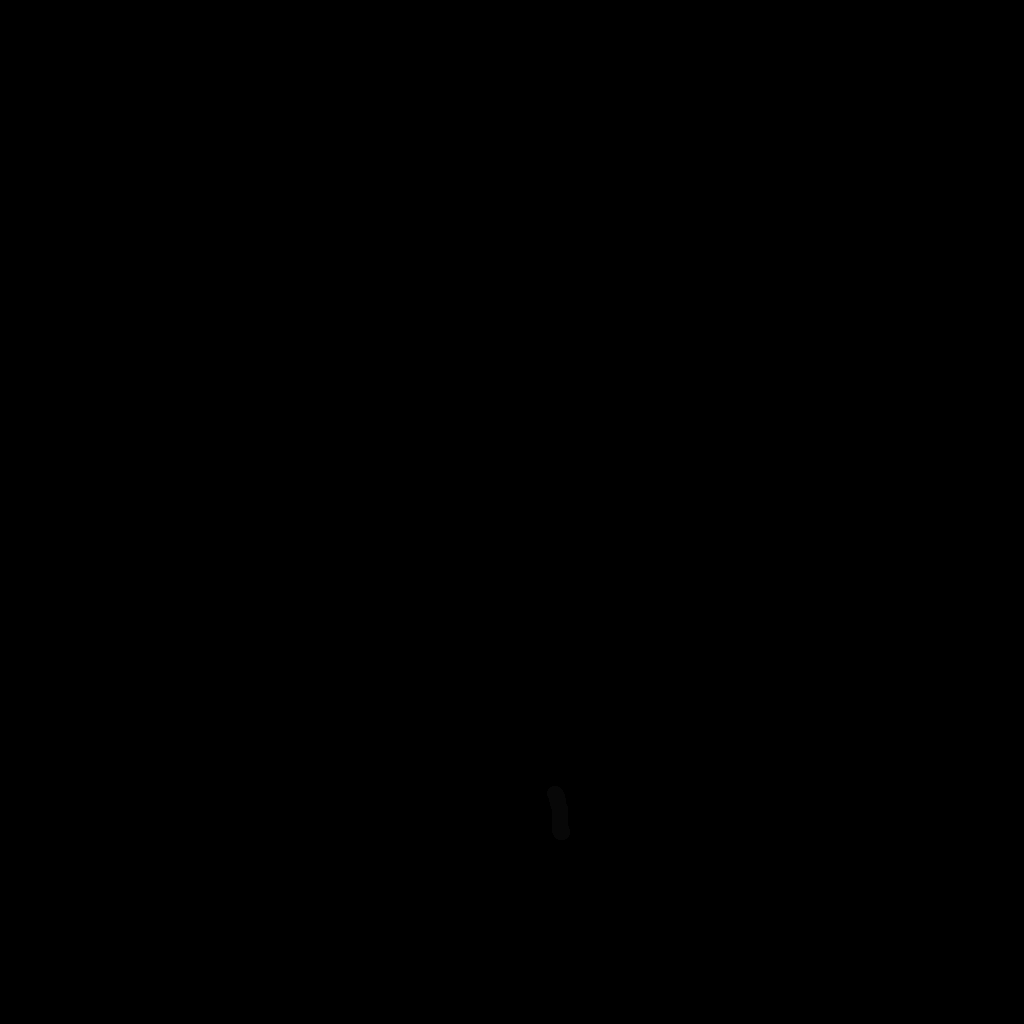

Supplement: Supplemental Information 1 [file peerj-cs-10-2097-s001.zip › IIT-AFF VL/masks/01_00000201.png]

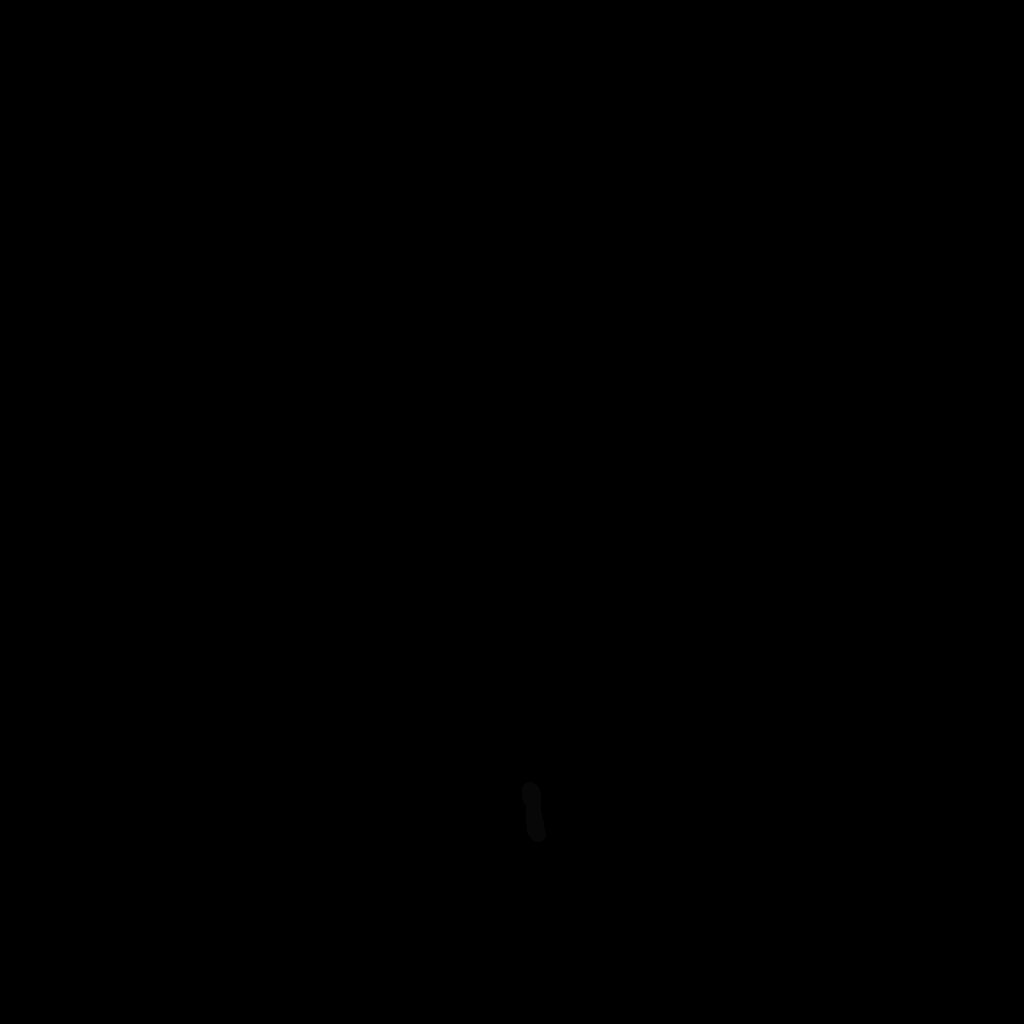

Supplement: Supplemental Information 1 [file peerj-cs-10-2097-s001.zip › IIT-AFF VL/masks/01_00000205.png]

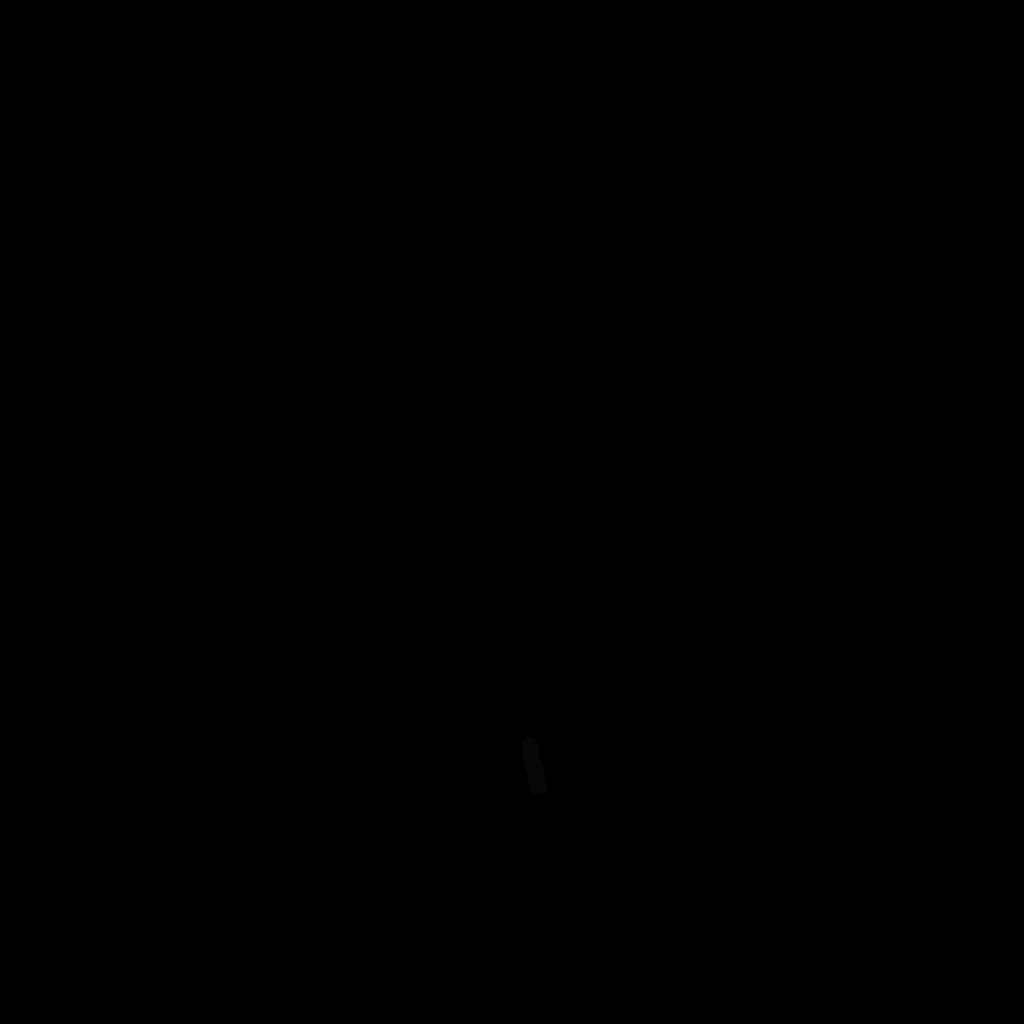

Supplement: Supplemental Information 1 [file peerj-cs-10-2097-s001.zip › IIT-AFF VL/masks/01_00000209.png]

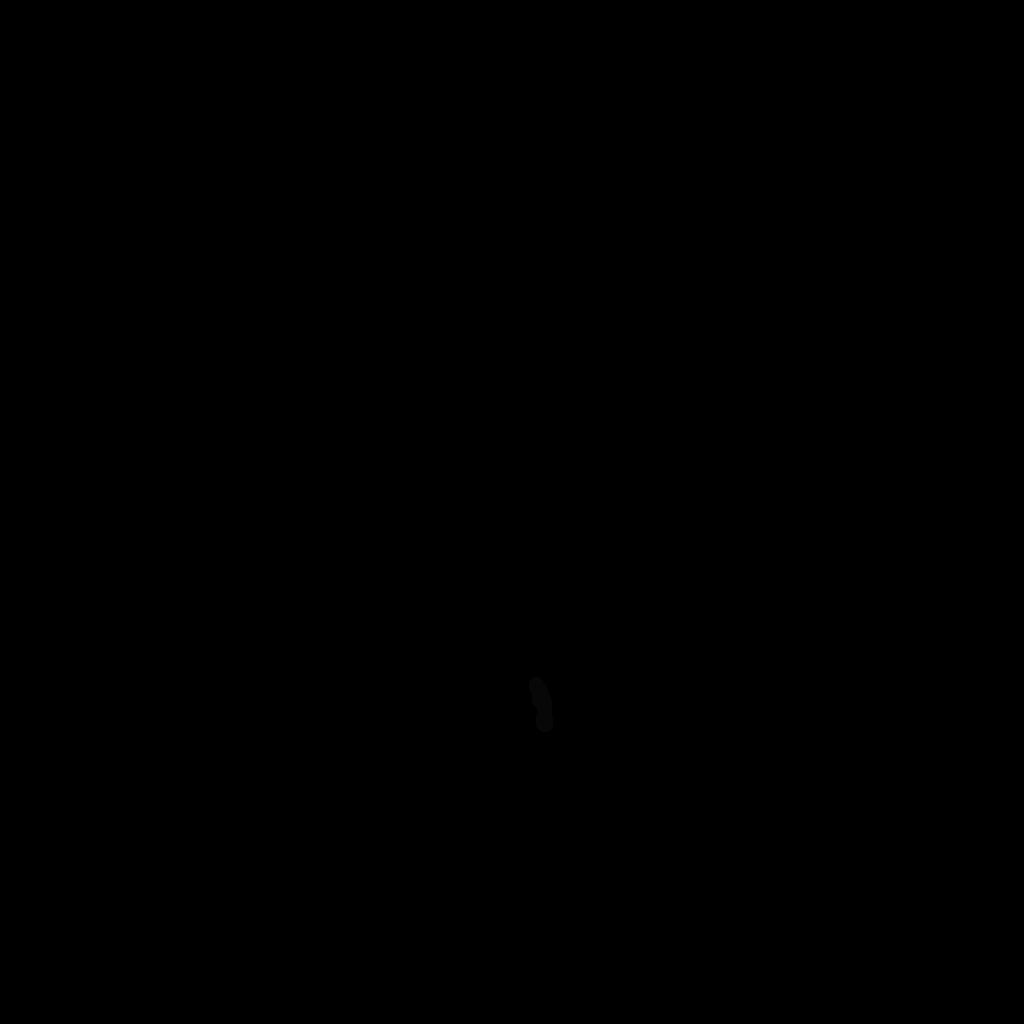

Supplement: Supplemental Information 1 [file peerj-cs-10-2097-s001.zip › IIT-AFF VL/masks/01_00000213.png]

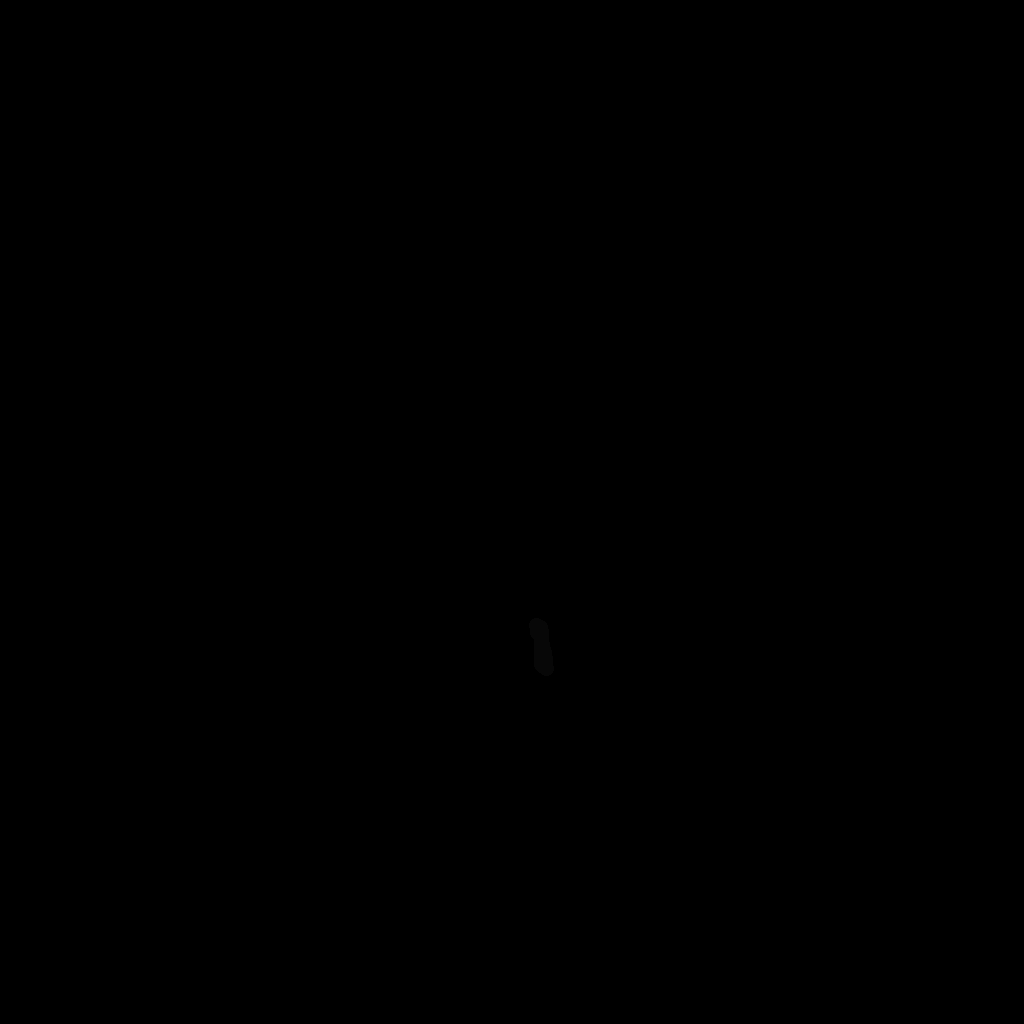

Supplement: Supplemental Information 1 [file peerj-cs-10-2097-s001.zip › IIT-AFF VL/masks/01_00000217.png]

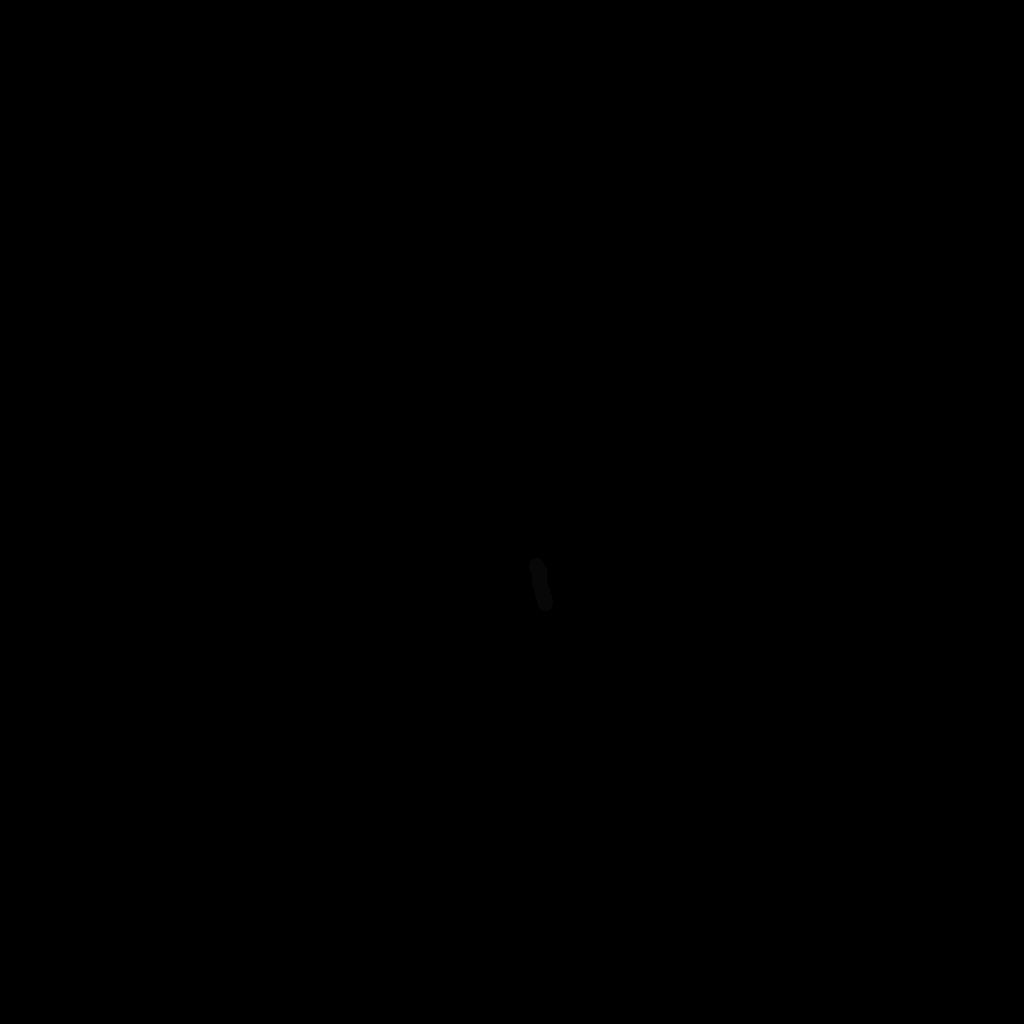

Supplement: Supplemental Information 1 [file peerj-cs-10-2097-s001.zip › IIT-AFF VL/masks/01_00000221.png]

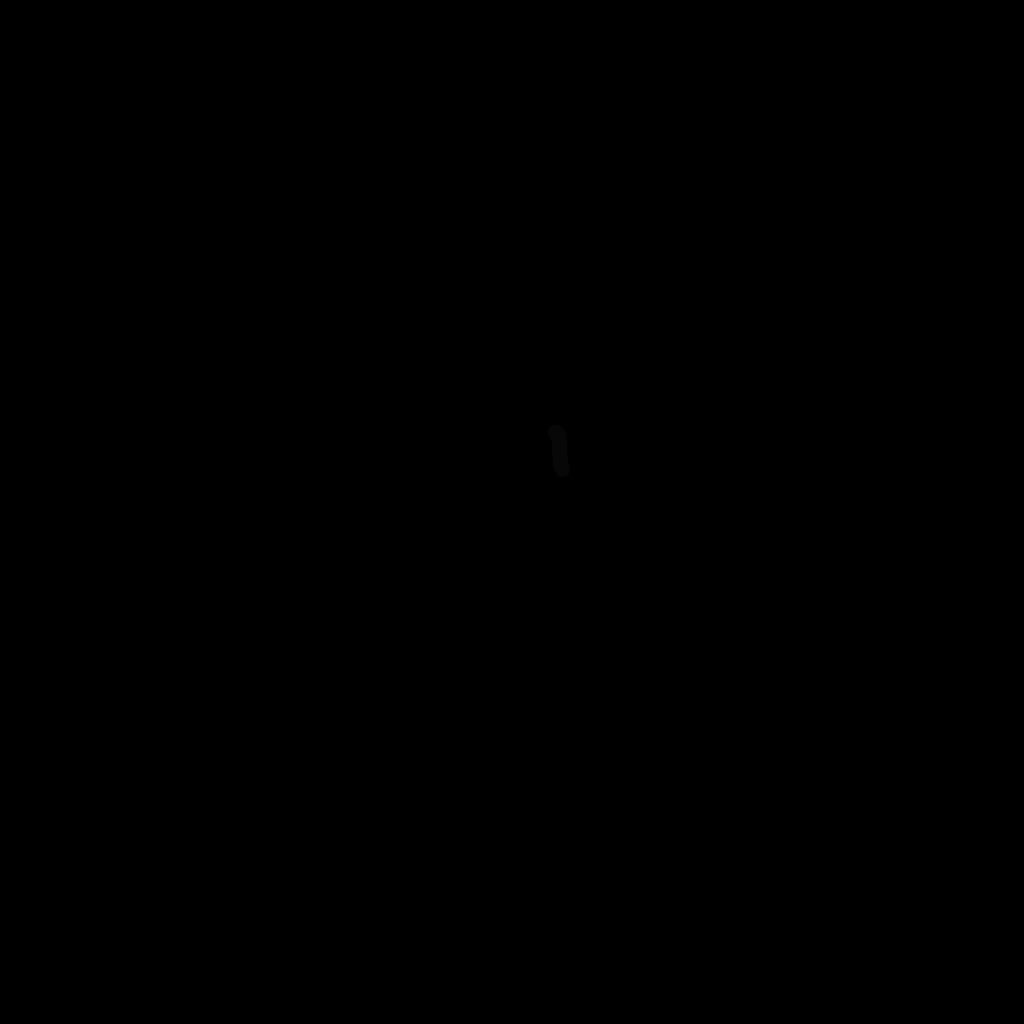

Supplement: Supplemental Information 1 [file peerj-cs-10-2097-s001.zip › IIT-AFF VL/masks/01_00000228.png]

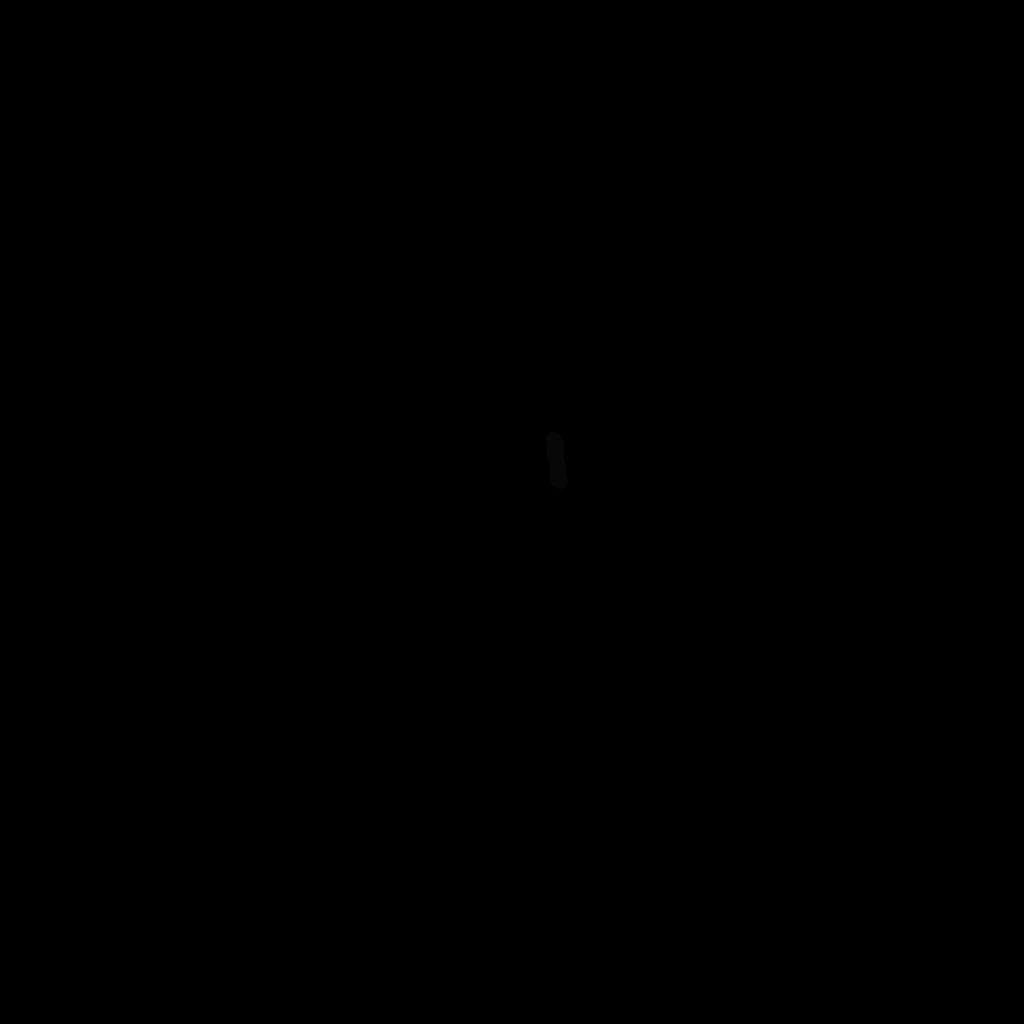

Supplement: Supplemental Information 1 [file peerj-cs-10-2097-s001.zip › IIT-AFF VL/masks/01_00000232.png]

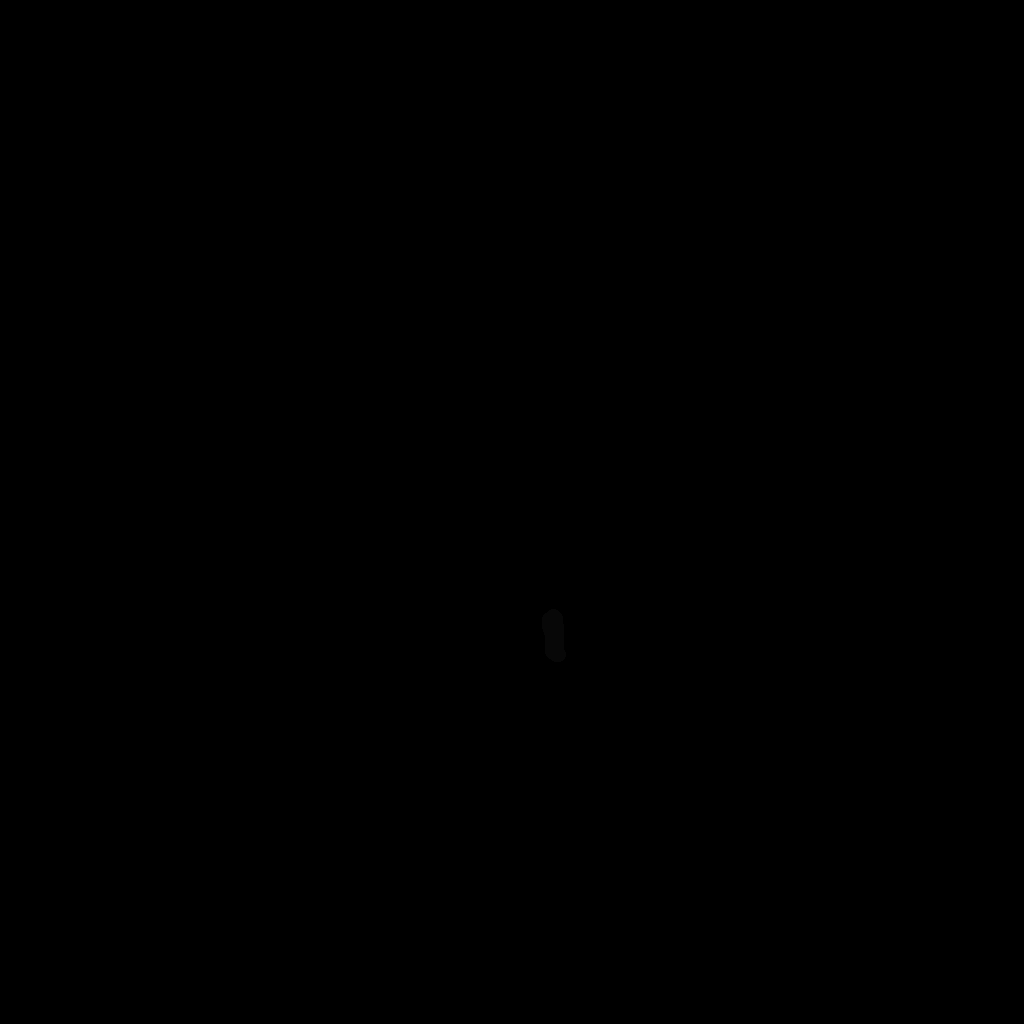

Supplement: Supplemental Information 1 [file peerj-cs-10-2097-s001.zip › IIT-AFF VL/masks/01_00000236.png]

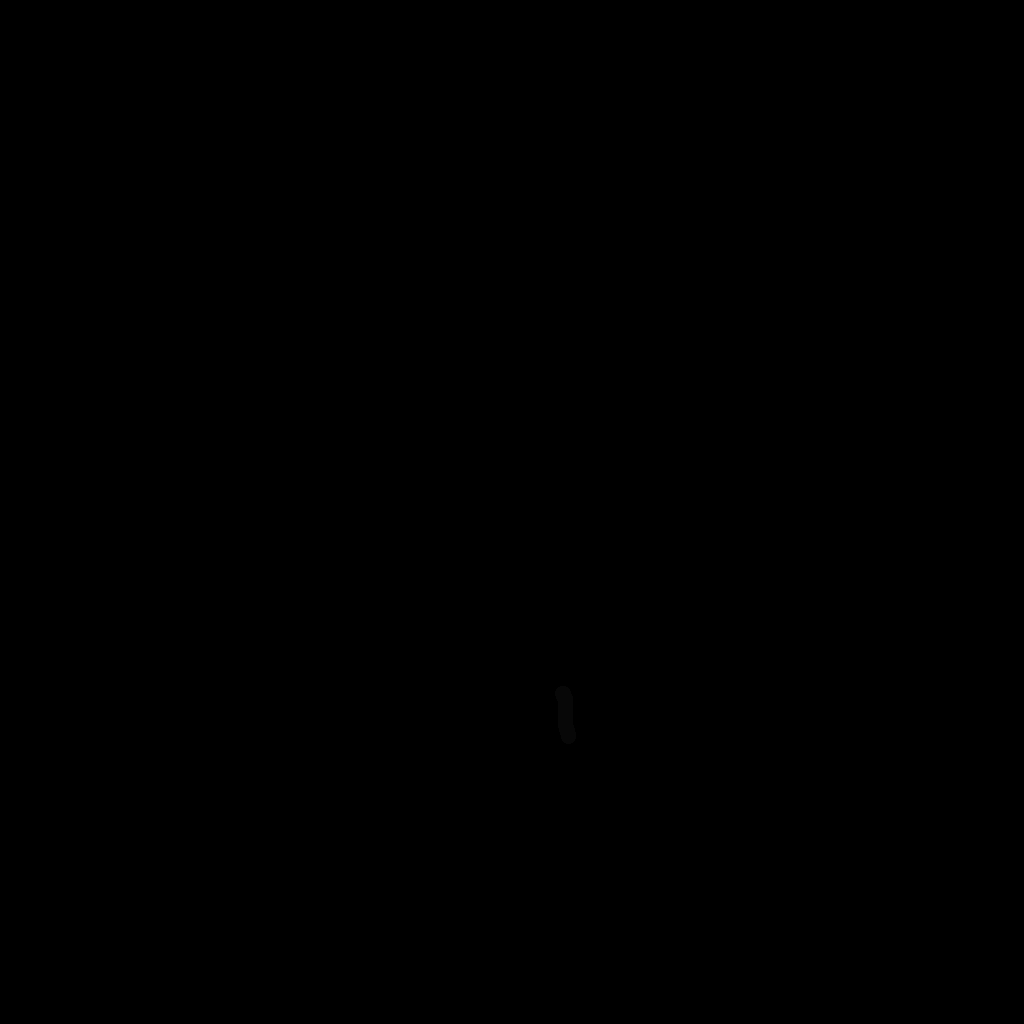

Supplement: Supplemental Information 1 [file peerj-cs-10-2097-s001.zip › IIT-AFF VL/masks/01_00000241.png]

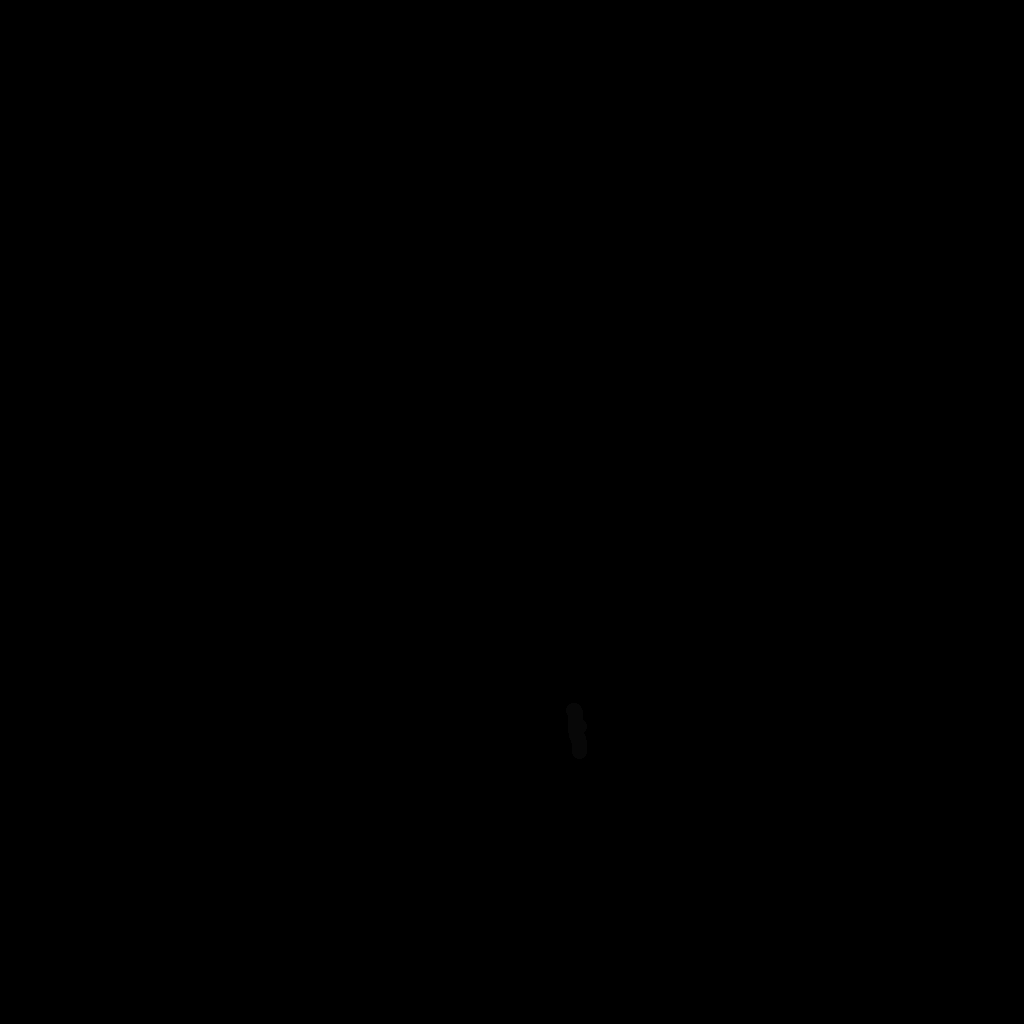

Supplement: Supplemental Information 1 [file peerj-cs-10-2097-s001.zip › IIT-AFF VL/masks/01_00000246.png]

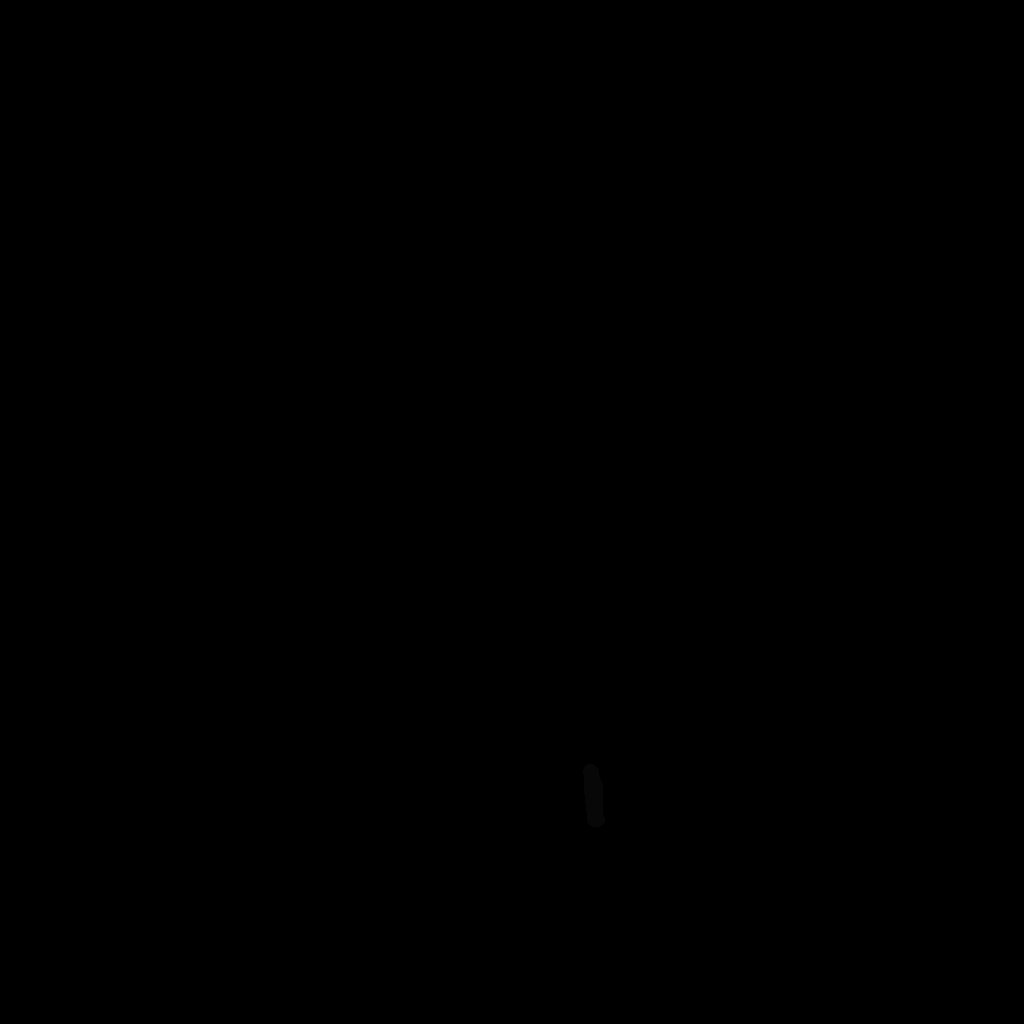

Supplement: Supplemental Information 1 [file peerj-cs-10-2097-s001.zip › IIT-AFF VL/masks/01_00000250.png]

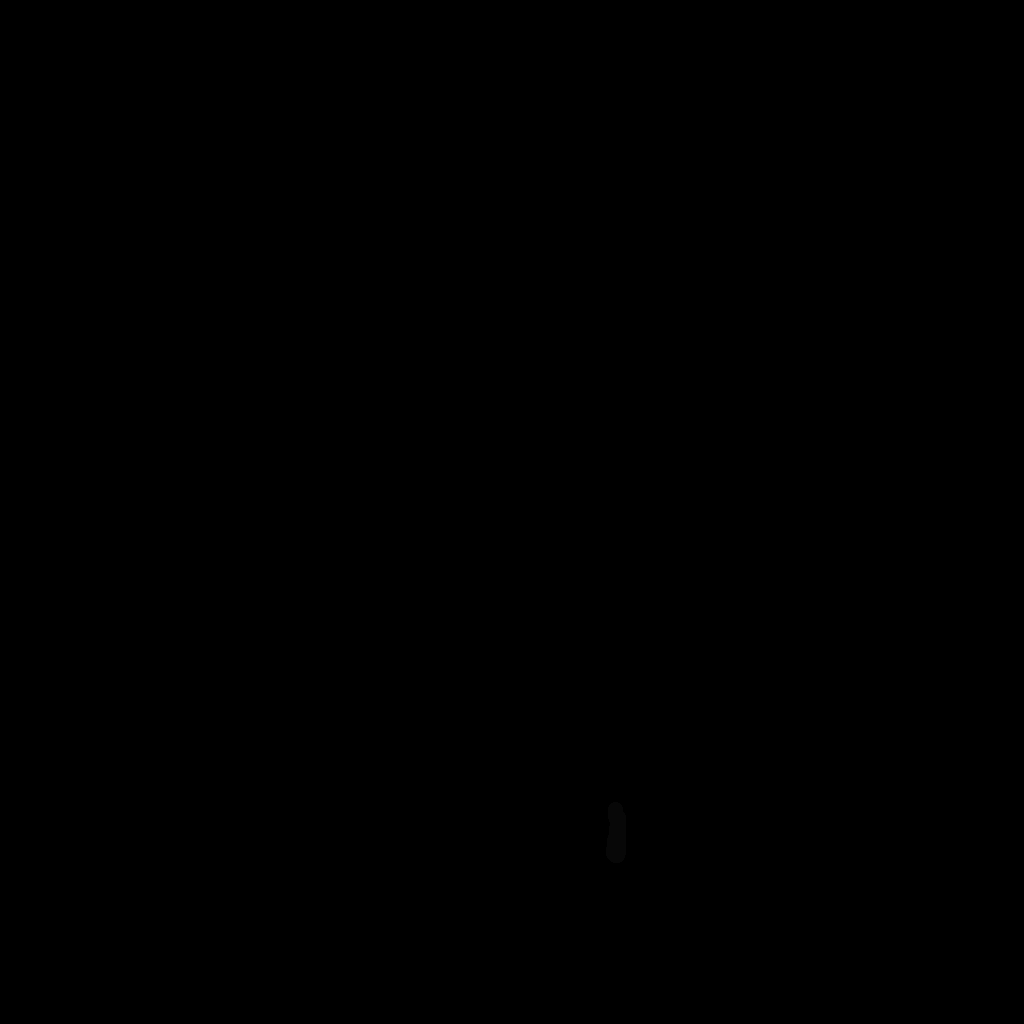

Supplement: Supplemental Information 1 [file peerj-cs-10-2097-s001.zip › IIT-AFF VL/masks/01_00000255.png]

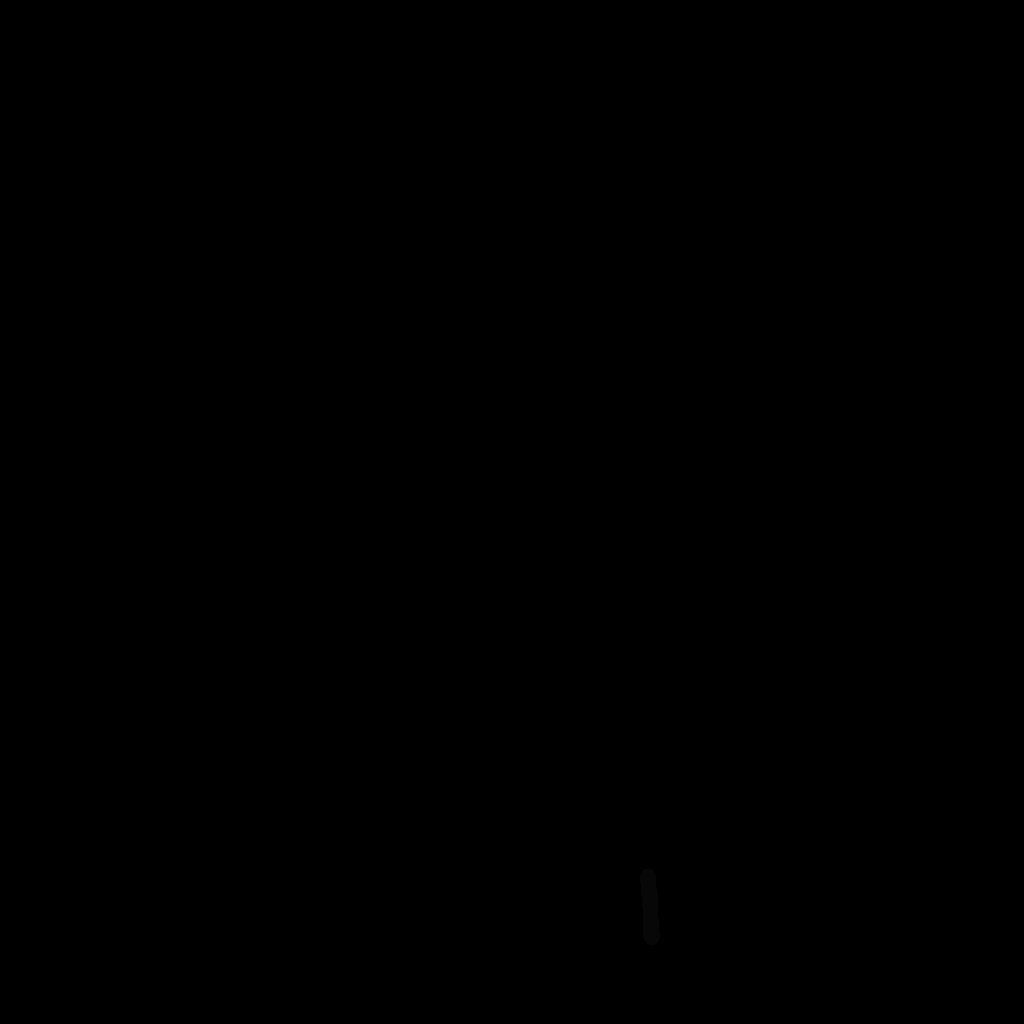

Supplement: Supplemental Information 1 [file peerj-cs-10-2097-s001.zip › IIT-AFF VL/masks/01_00000259.png]

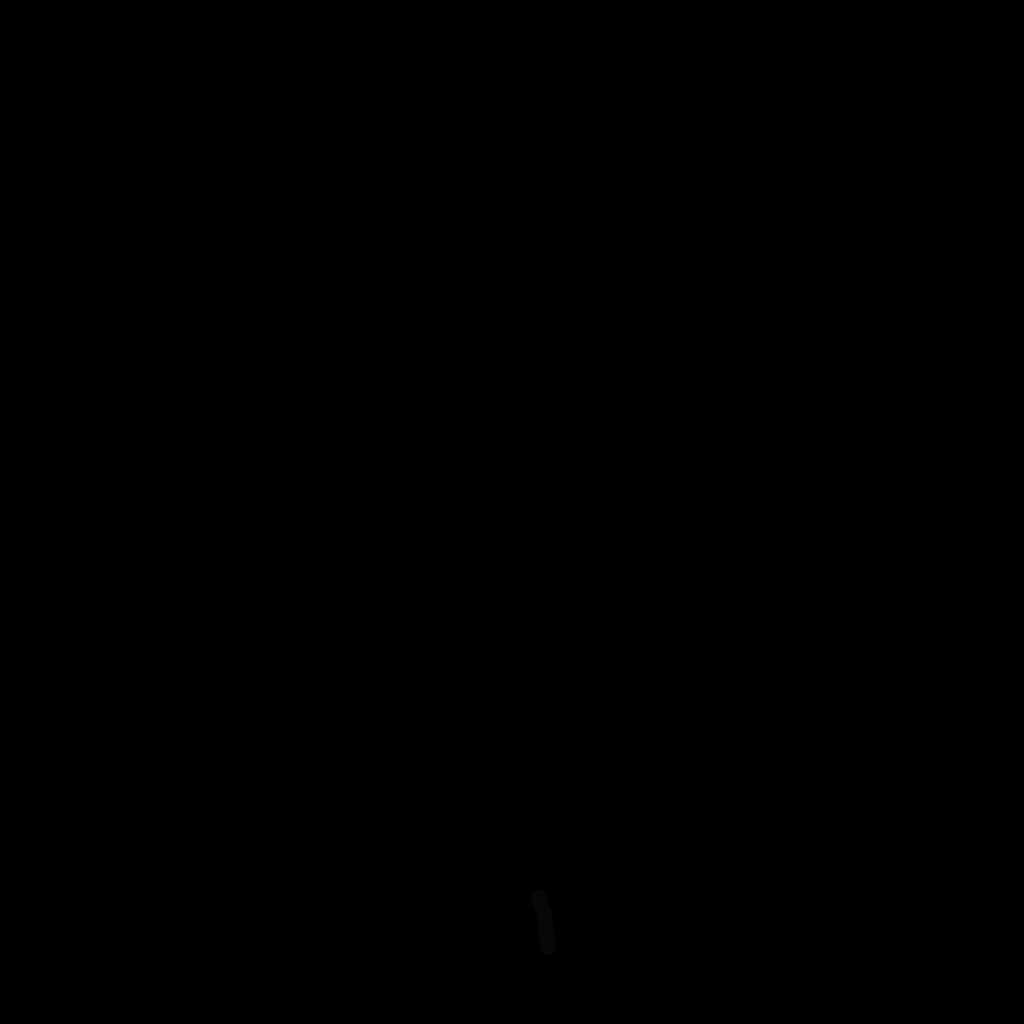

Supplement: Supplemental Information 1 [file peerj-cs-10-2097-s001.zip › IIT-AFF VL/masks/01_00000321.png]

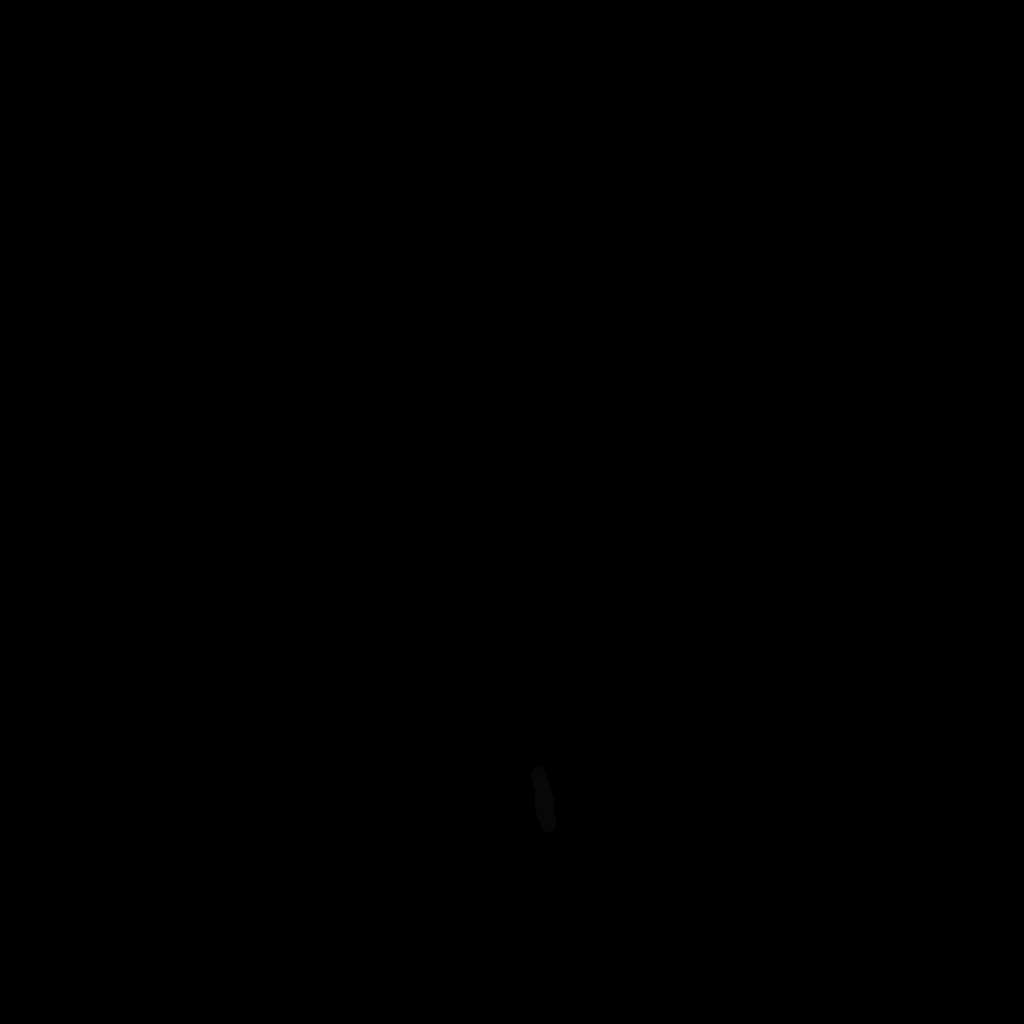

Supplement: Supplemental Information 1 [file peerj-cs-10-2097-s001.zip › IIT-AFF VL/masks/01_00000325.png]

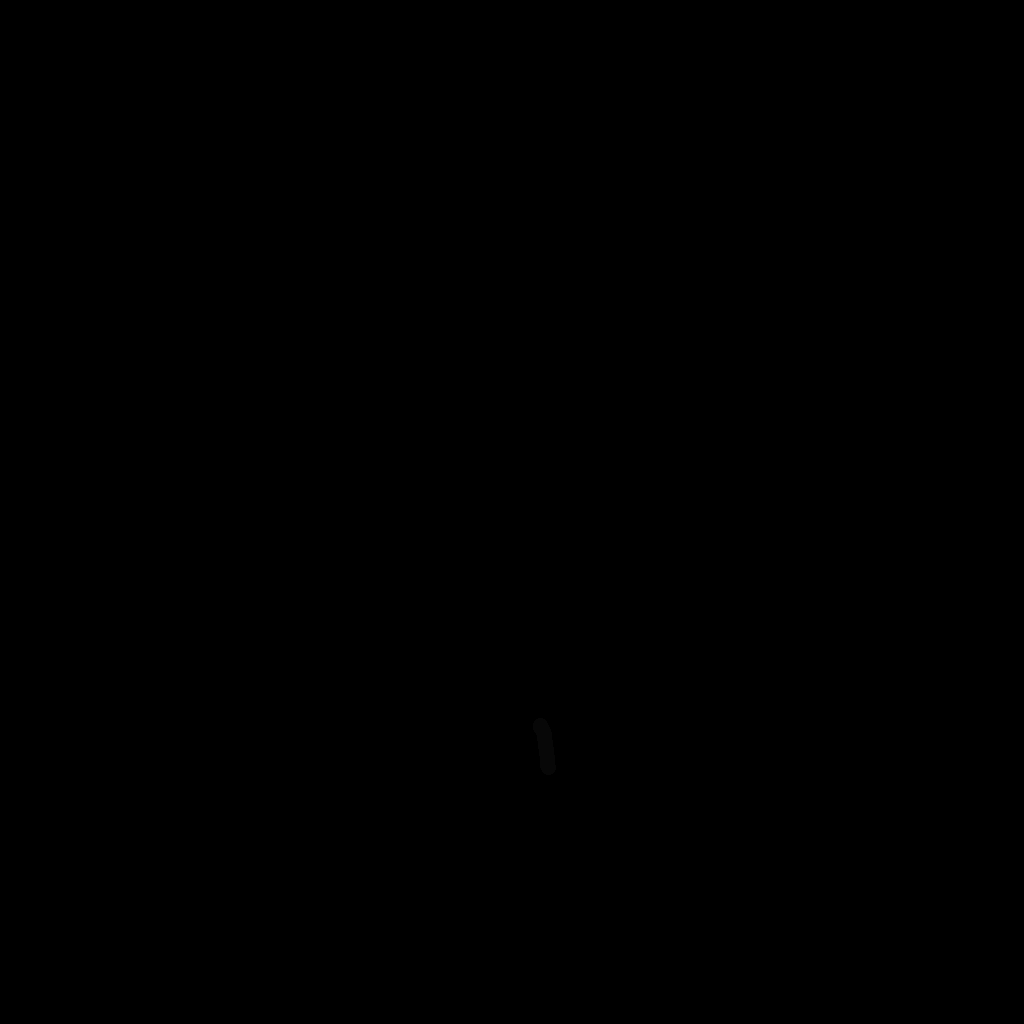

Supplement: Supplemental Information 1 [file peerj-cs-10-2097-s001.zip › IIT-AFF VL/masks/01_00000330.png]

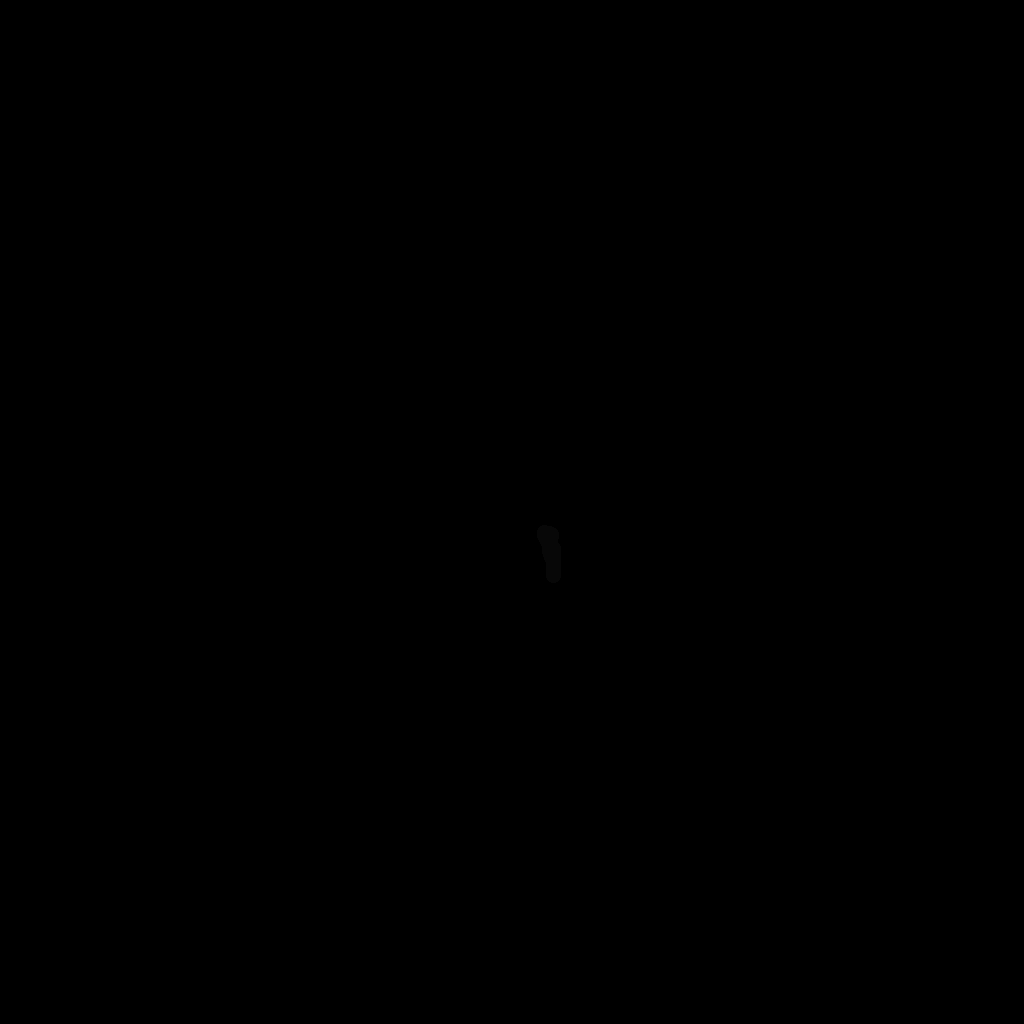

Supplement: Supplemental Information 1 [file peerj-cs-10-2097-s001.zip › IIT-AFF VL/masks/01_00000335.png]

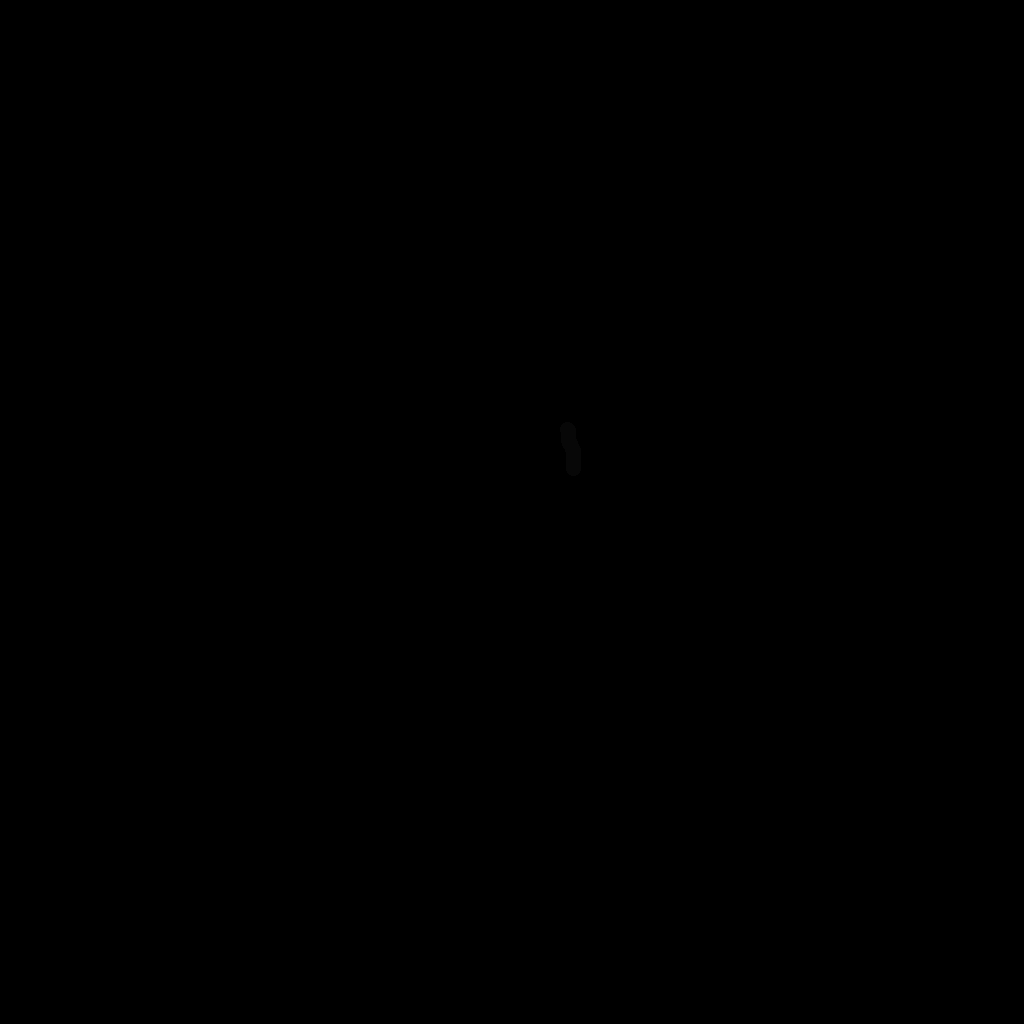

Supplement: Supplemental Information 1 [file peerj-cs-10-2097-s001.zip › IIT-AFF VL/masks/01_00000342.png]

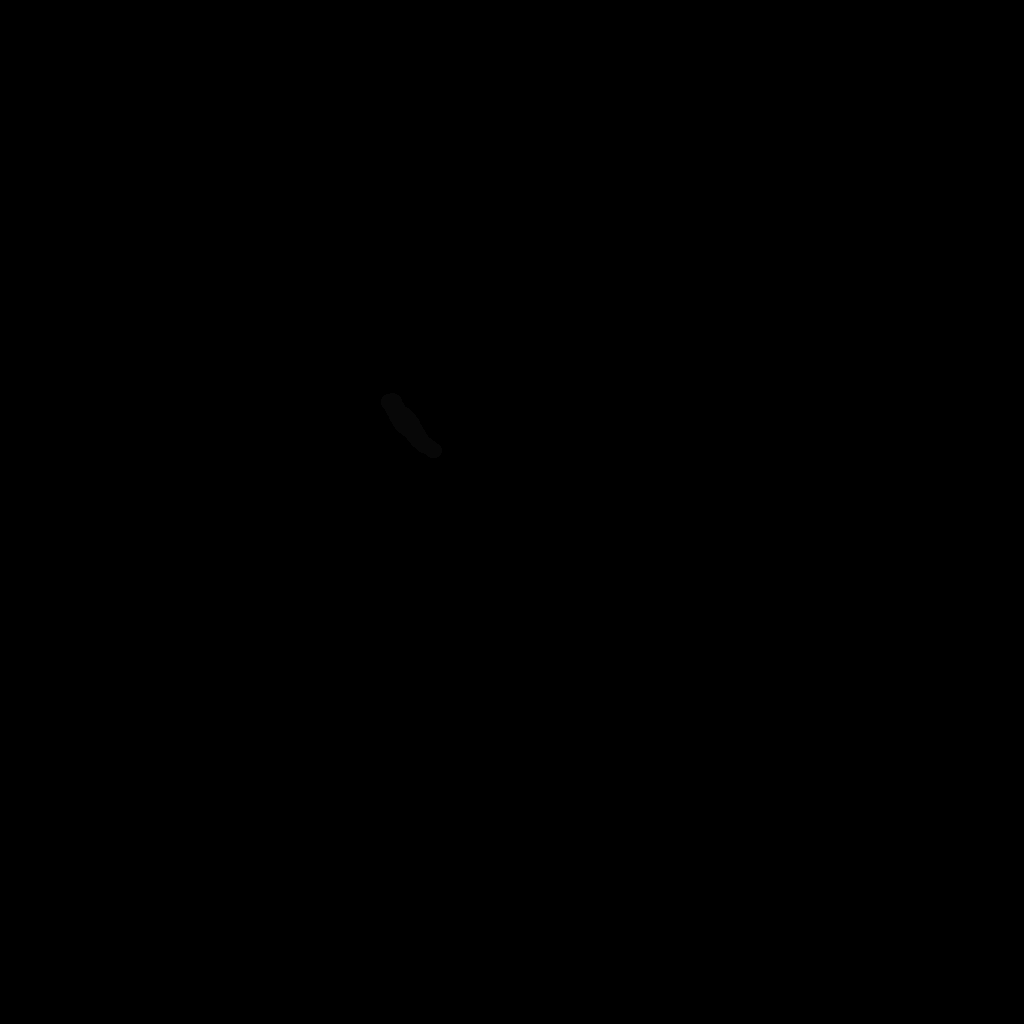

Supplement: Supplemental Information 1 [file peerj-cs-10-2097-s001.zip › IIT-AFF VL/masks/02_00000045.png]

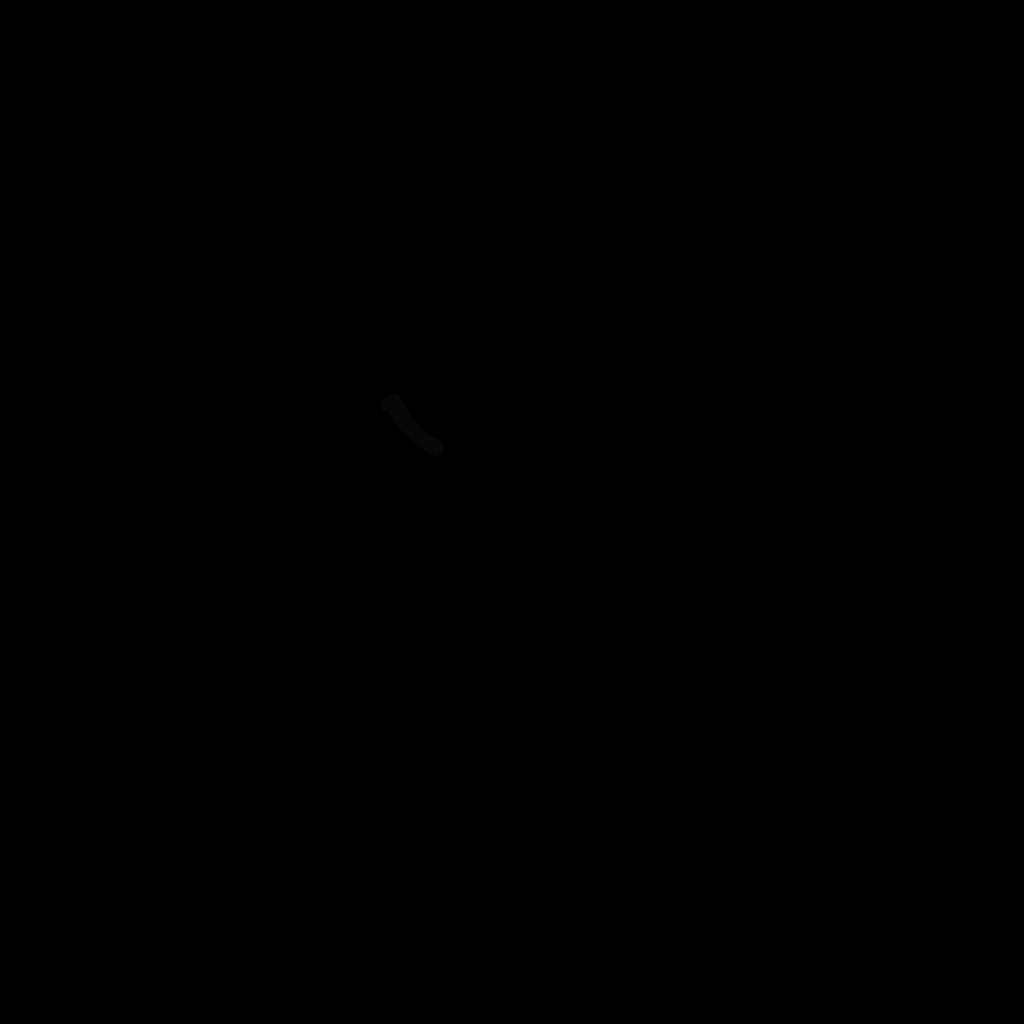

Supplement: Supplemental Information 1 [file peerj-cs-10-2097-s001.zip › IIT-AFF VL/masks/02_00000050.png]

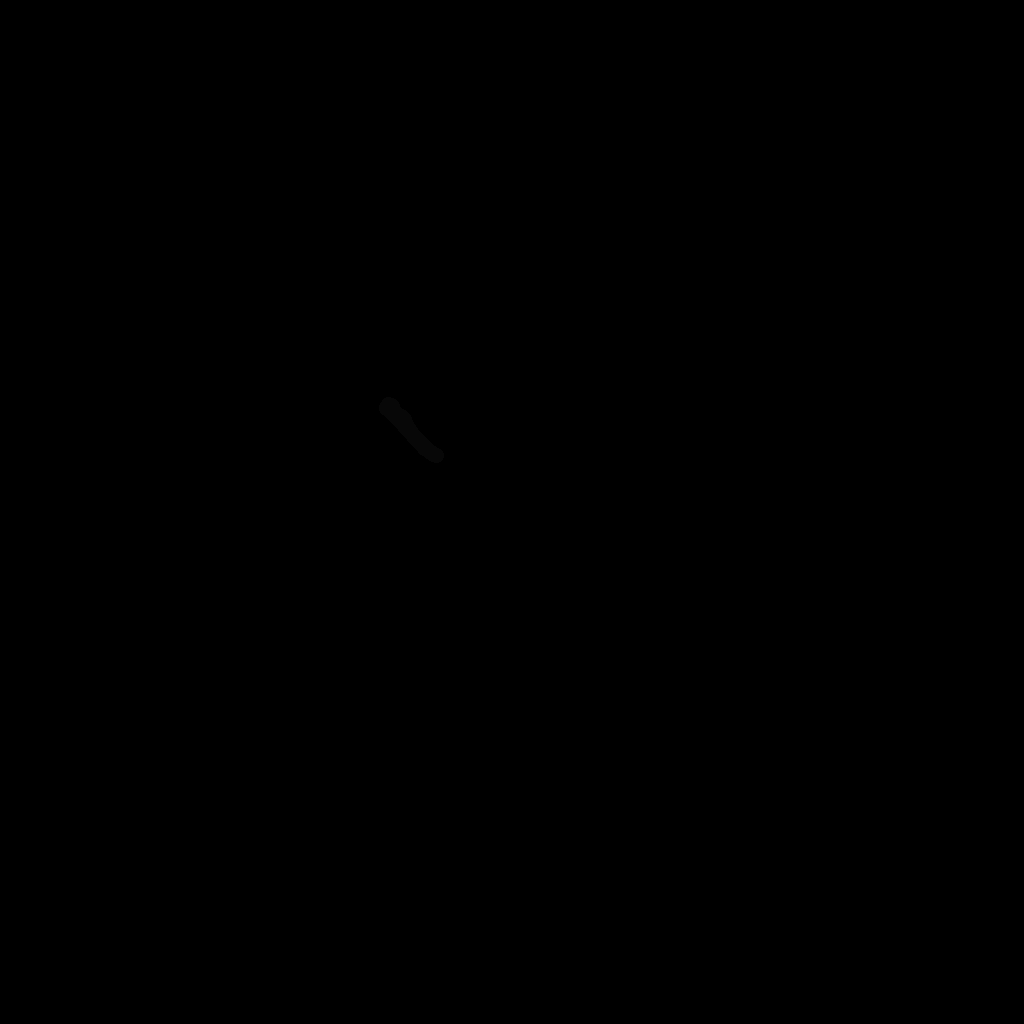

Supplement: Supplemental Information 1 [file peerj-cs-10-2097-s001.zip › IIT-AFF VL/masks/02_00000056.png]

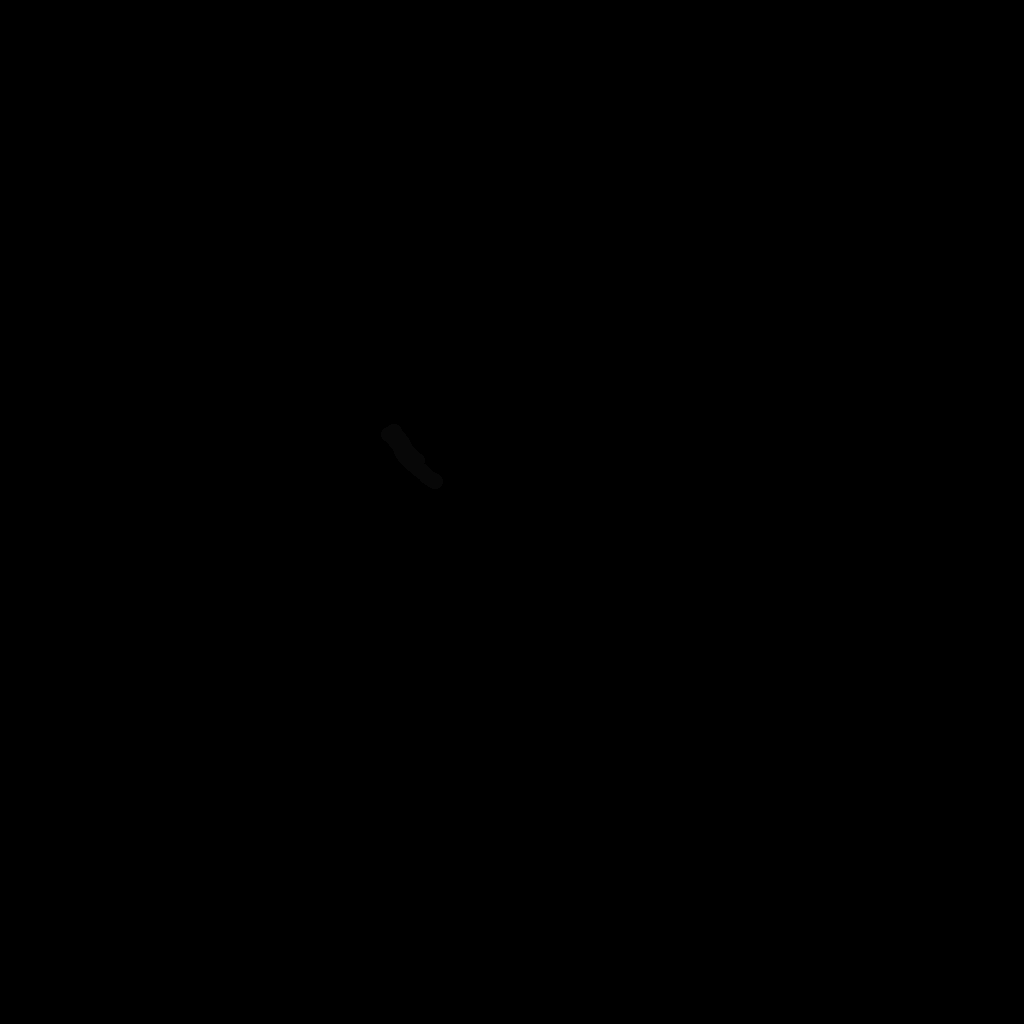

Supplement: Supplemental Information 1 [file peerj-cs-10-2097-s001.zip › IIT-AFF VL/masks/02_00000060.png]

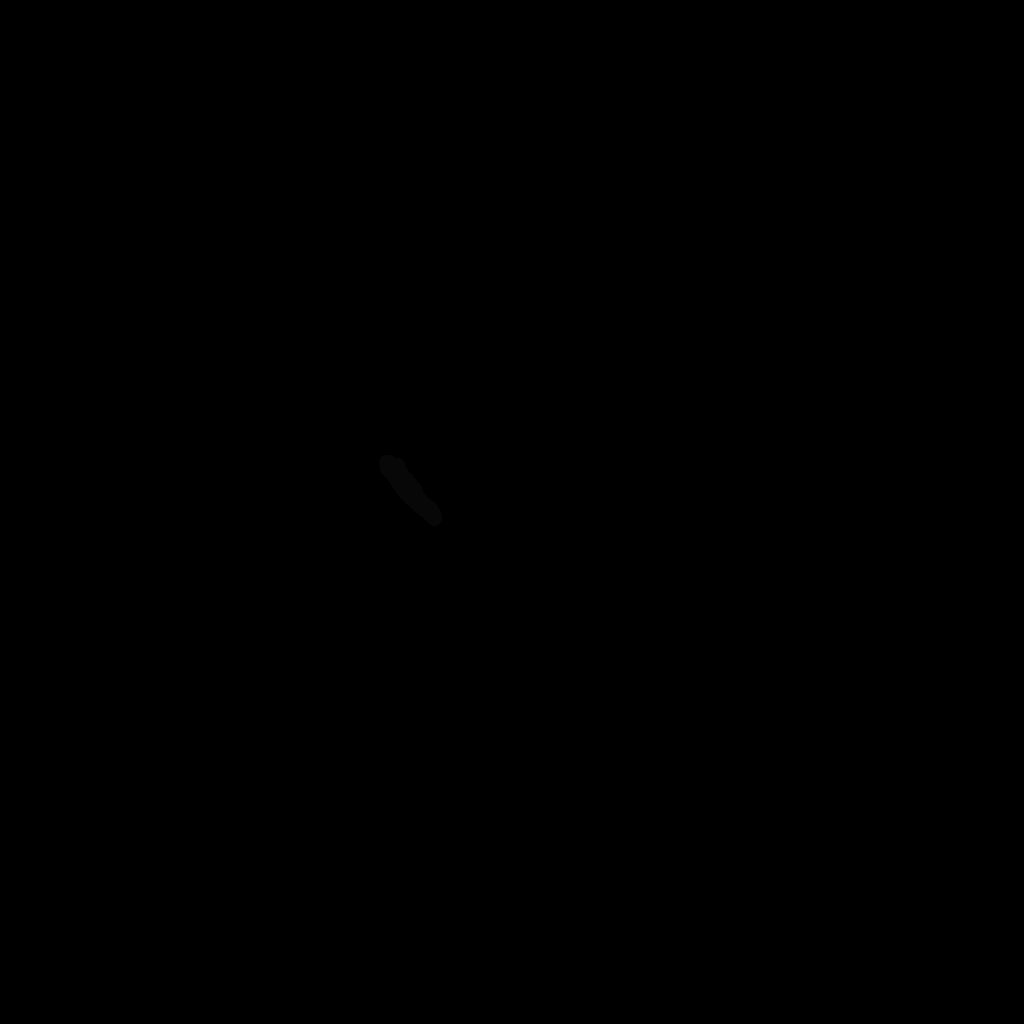

Supplement: Supplemental Information 1 [file peerj-cs-10-2097-s001.zip › IIT-AFF VL/masks/02_00000064.png]

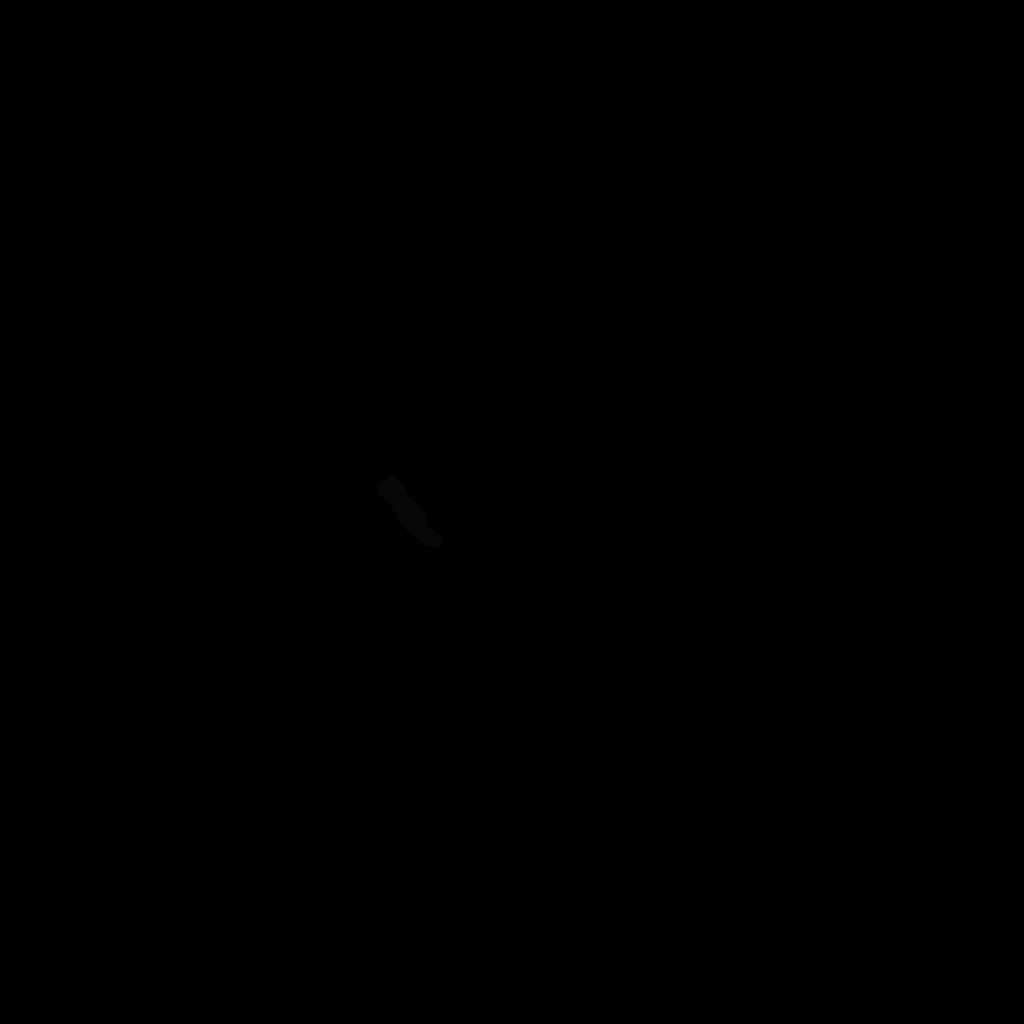

Supplement: Supplemental Information 1 [file peerj-cs-10-2097-s001.zip › IIT-AFF VL/masks/02_00000068.png]

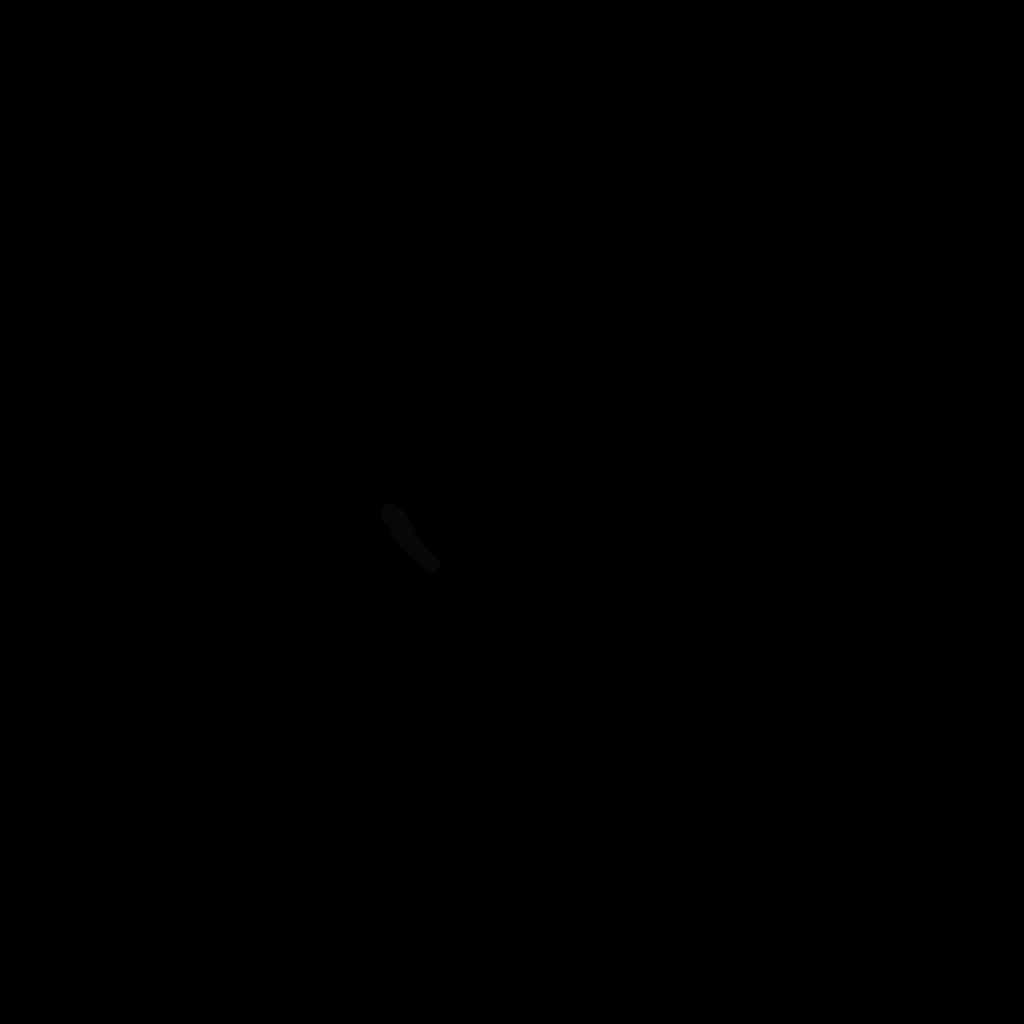

Supplement: Supplemental Information 1 [file peerj-cs-10-2097-s001.zip › IIT-AFF VL/masks/02_00000073.png]

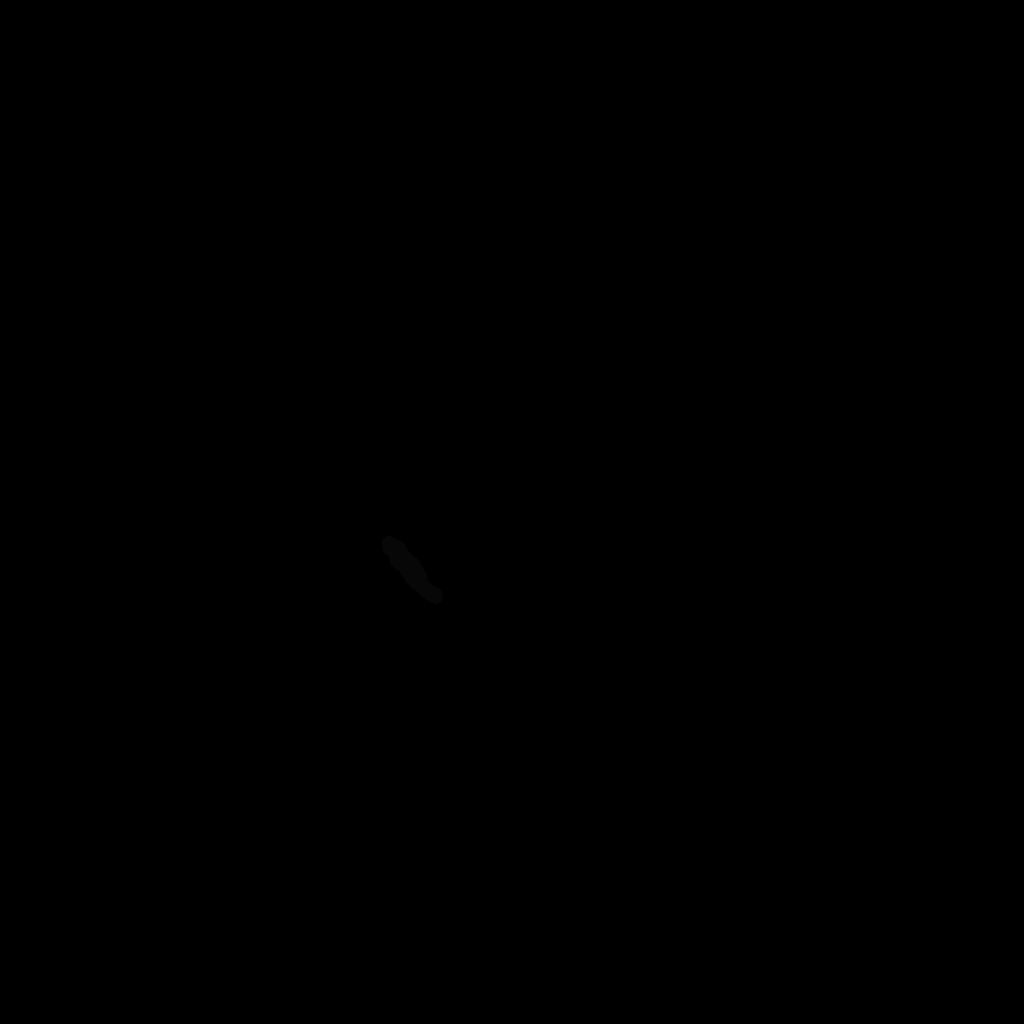

Supplement: Supplemental Information 1 [file peerj-cs-10-2097-s001.zip › IIT-AFF VL/masks/02_00000077.png]

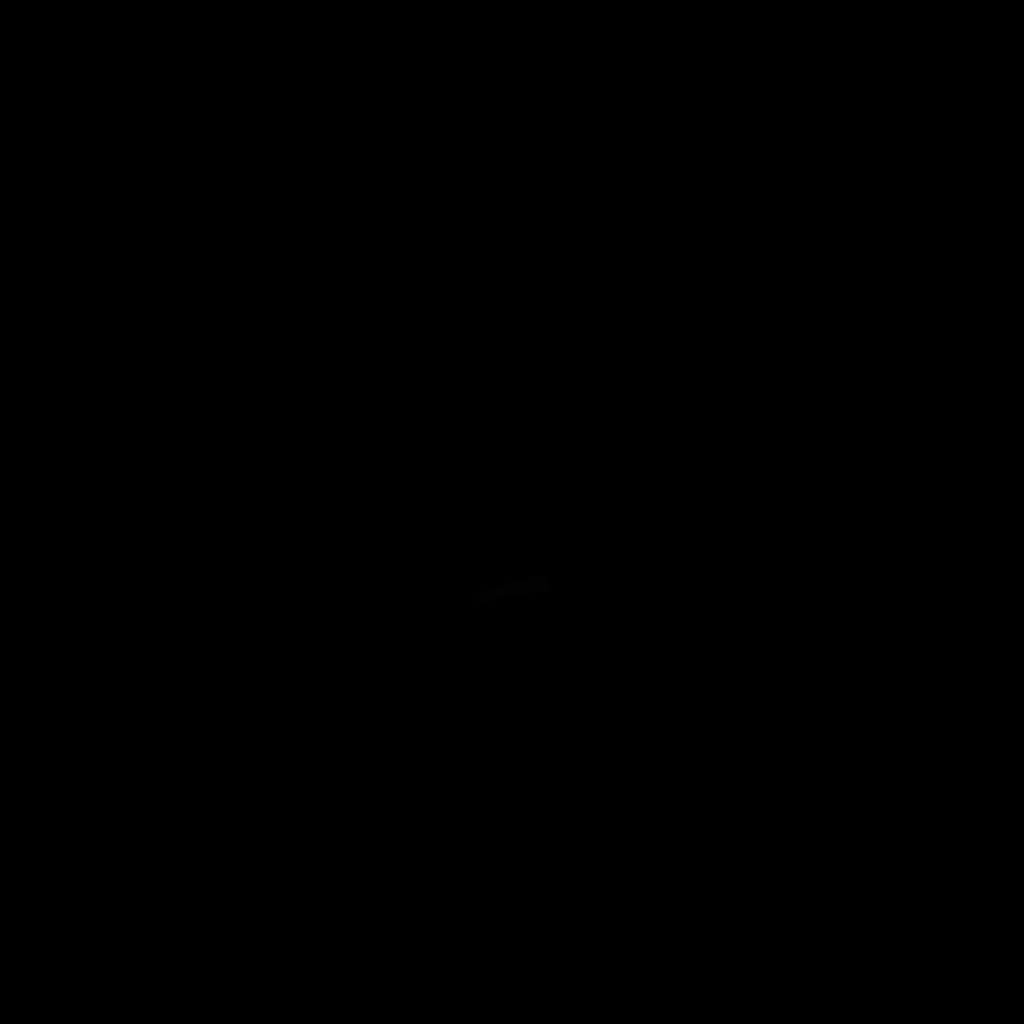

Supplement: Supplemental Information 1 [file peerj-cs-10-2097-s001.zip › IIT-AFF VL/masks/02_00000081.png]

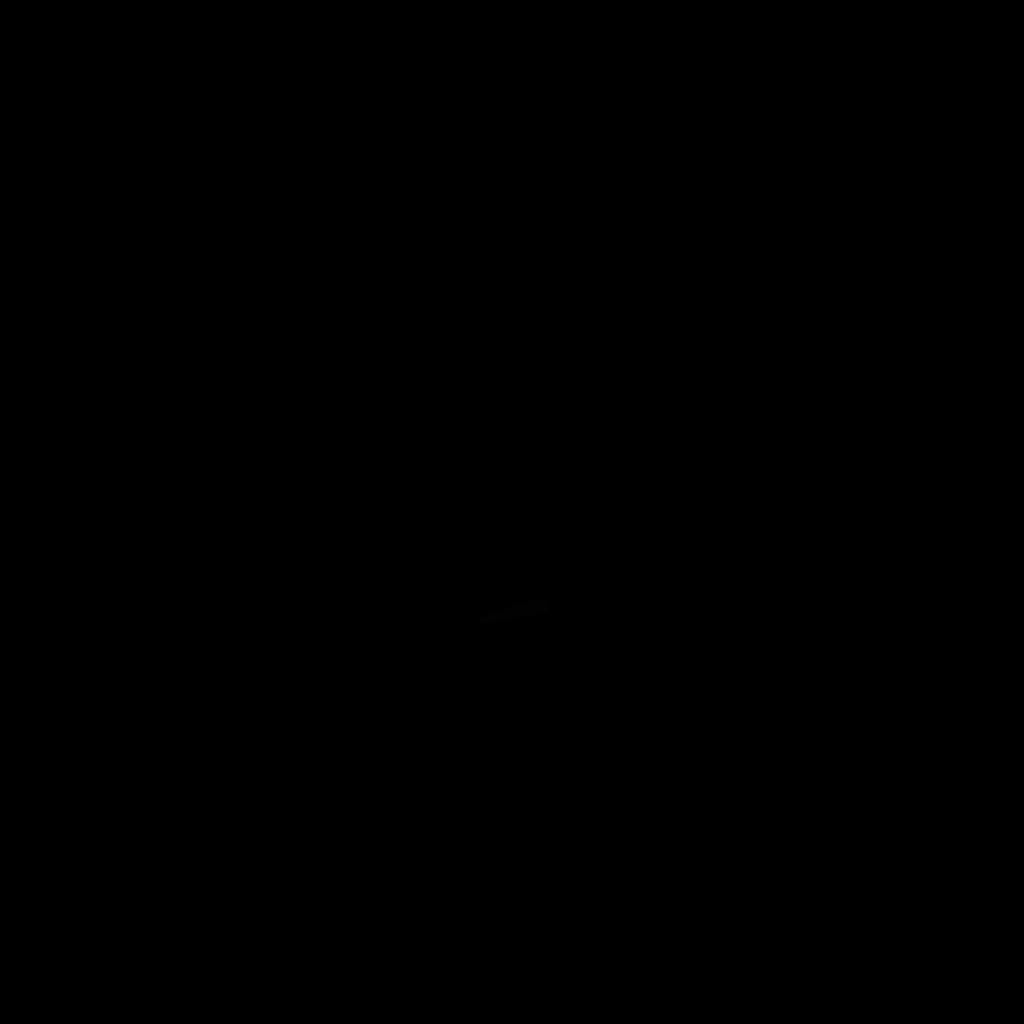

Supplement: Supplemental Information 1 [file peerj-cs-10-2097-s001.zip › IIT-AFF VL/masks/02_00000085.png]

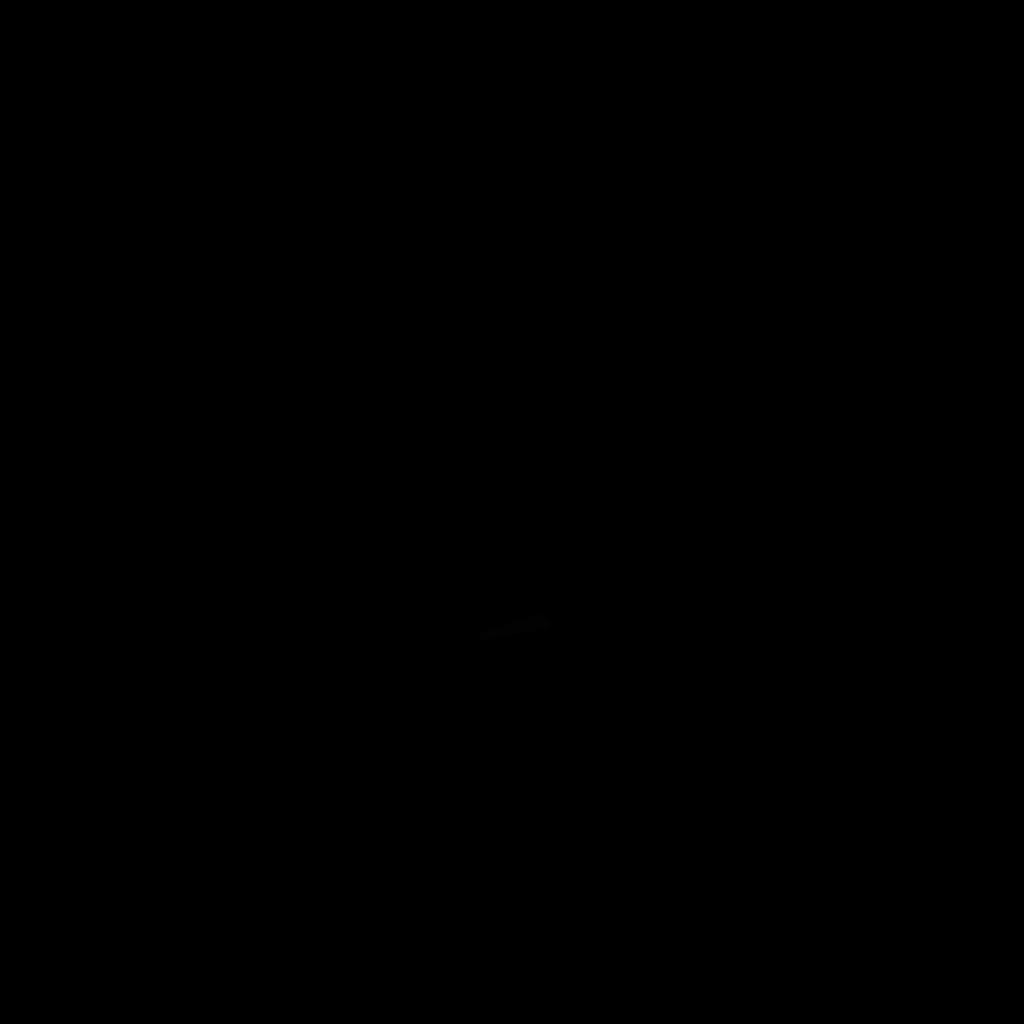

Supplement: Supplemental Information 1 [file peerj-cs-10-2097-s001.zip › IIT-AFF VL/masks/02_00000089.png]

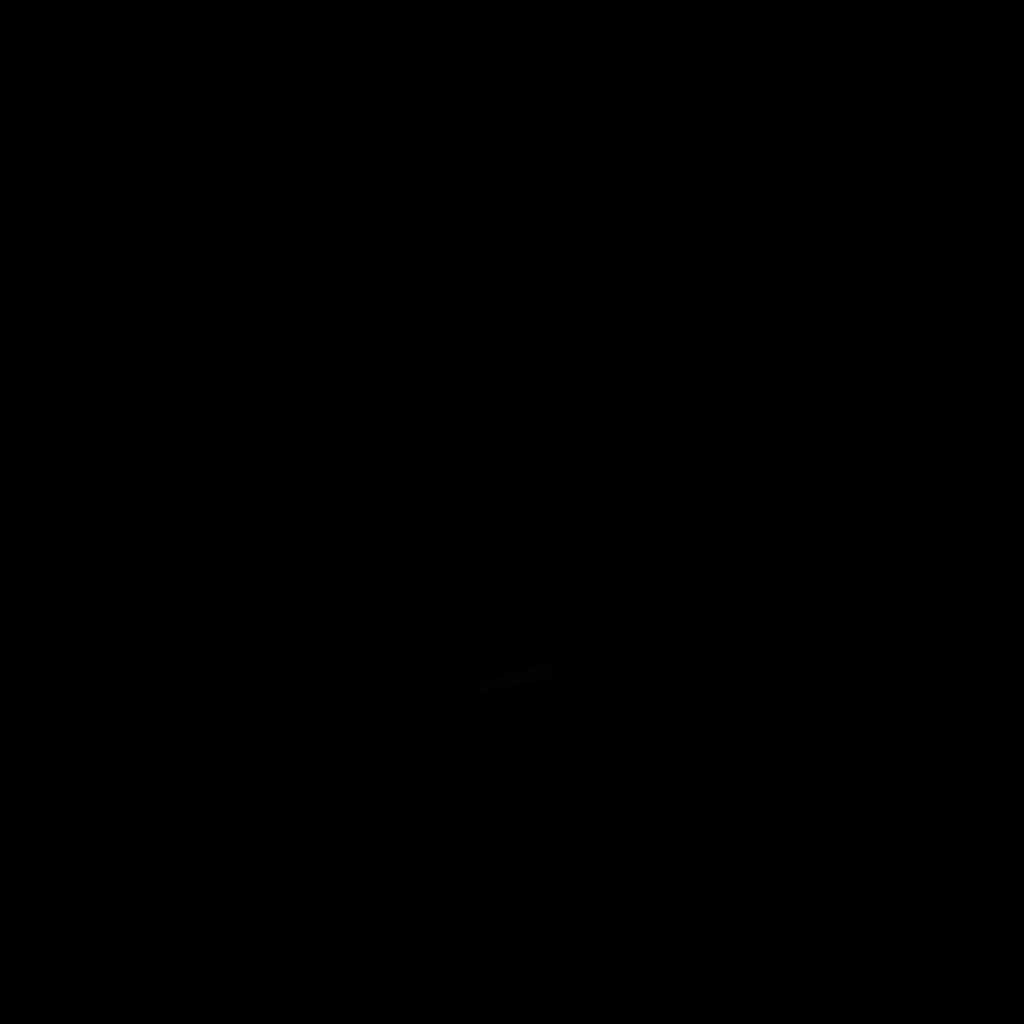

Supplement: Supplemental Information 1 [file peerj-cs-10-2097-s001.zip › IIT-AFF VL/masks/02_00000093.png]

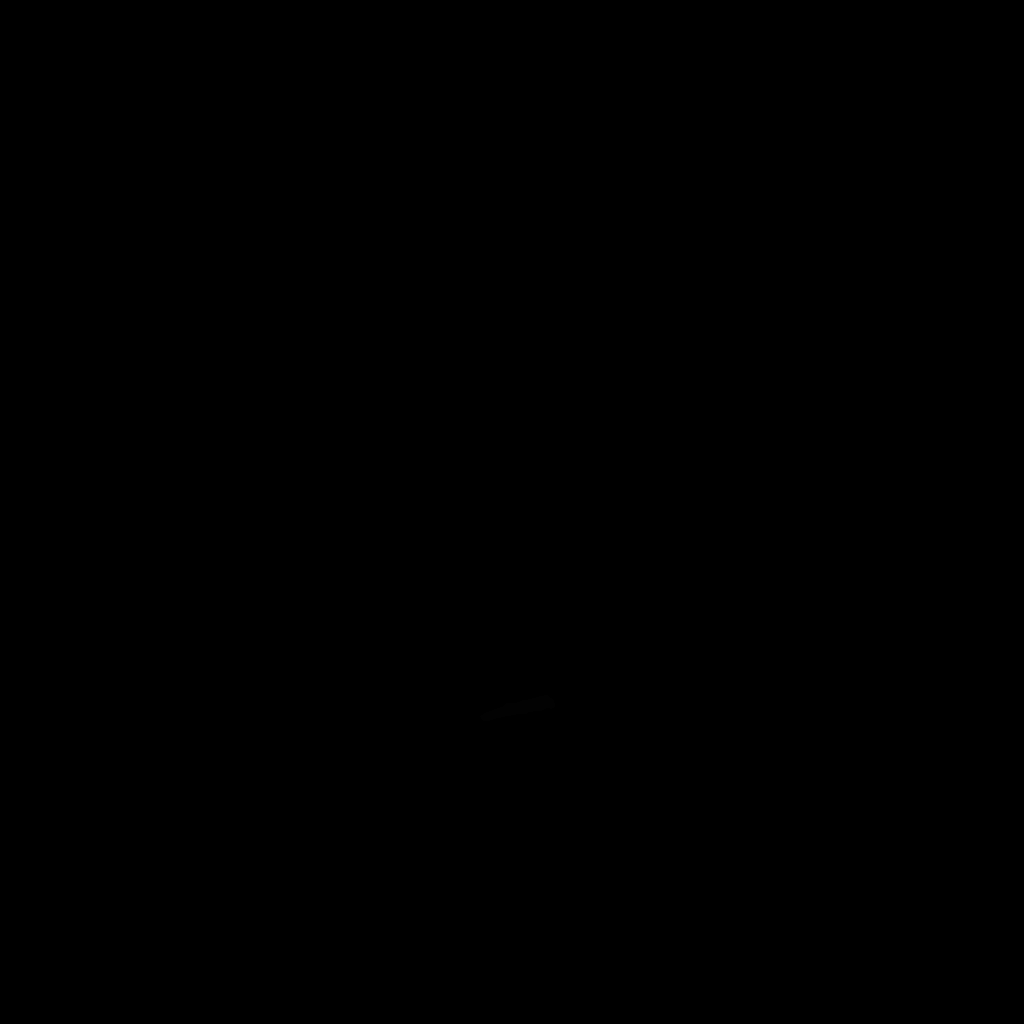

Supplement: Supplemental Information 1 [file peerj-cs-10-2097-s001.zip › IIT-AFF VL/masks/02_00000098.png]

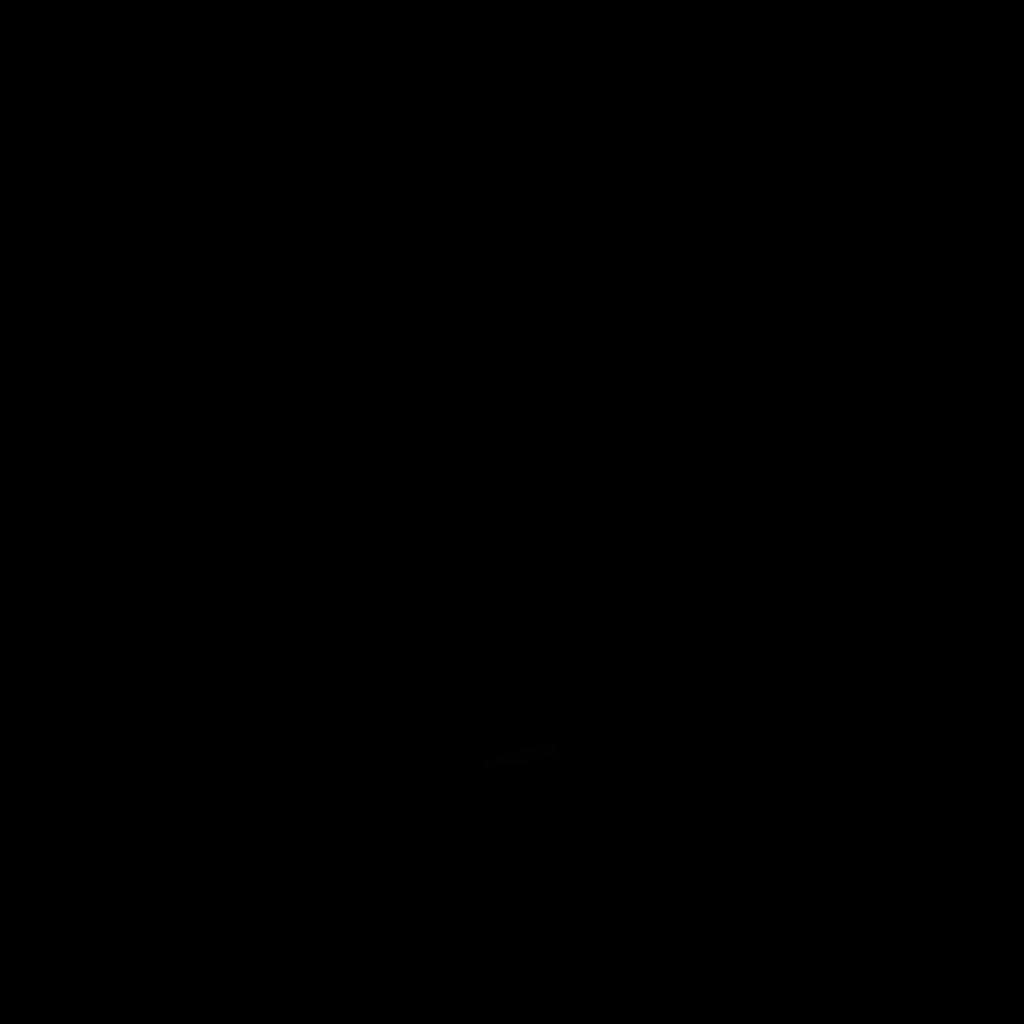

Supplement: Supplemental Information 1 [file peerj-cs-10-2097-s001.zip › IIT-AFF VL/masks/02_00000102.png]

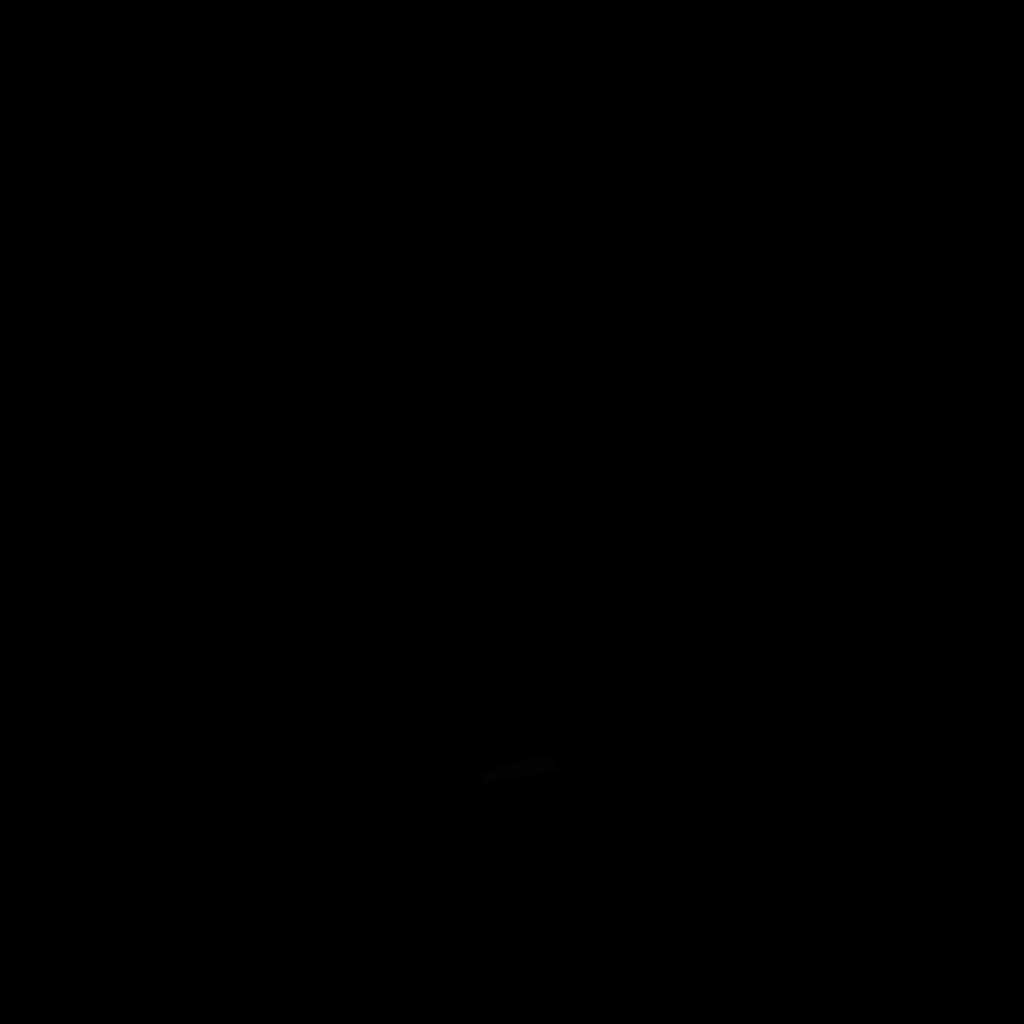

Supplement: Supplemental Information 1 [file peerj-cs-10-2097-s001.zip › IIT-AFF VL/masks/02_00000106.png]

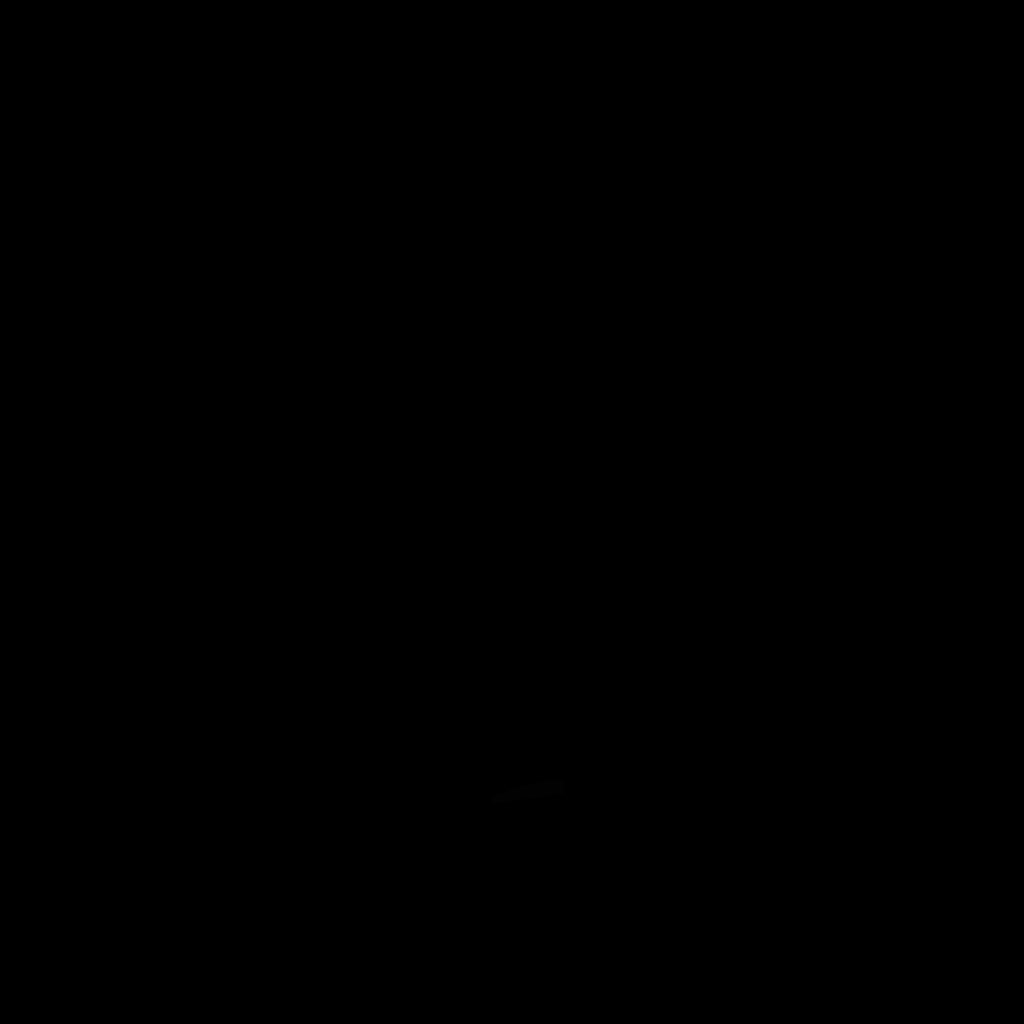

Supplement: Supplemental Information 1 [file peerj-cs-10-2097-s001.zip › IIT-AFF VL/masks/02_00000110.png]

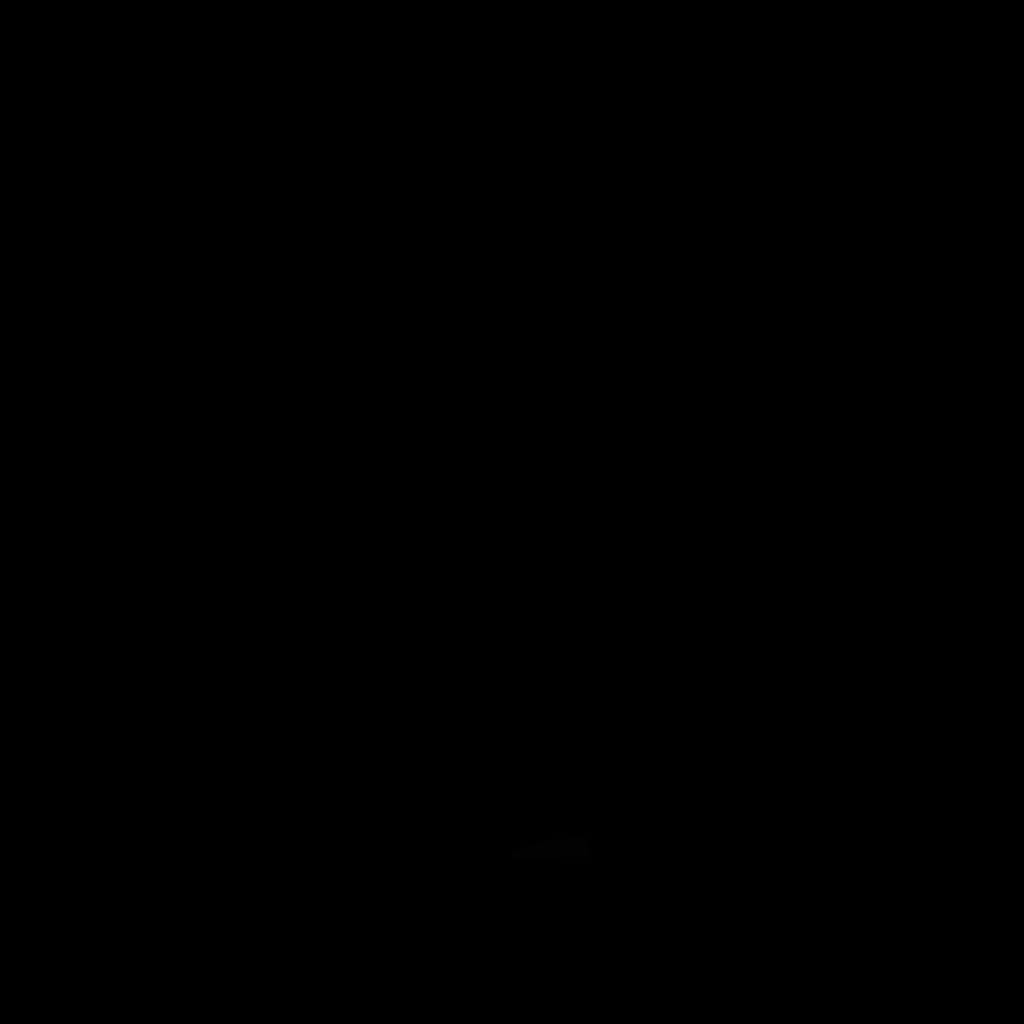

Supplement: Supplemental Information 1 [file peerj-cs-10-2097-s001.zip › IIT-AFF VL/masks/02_00000116.png]

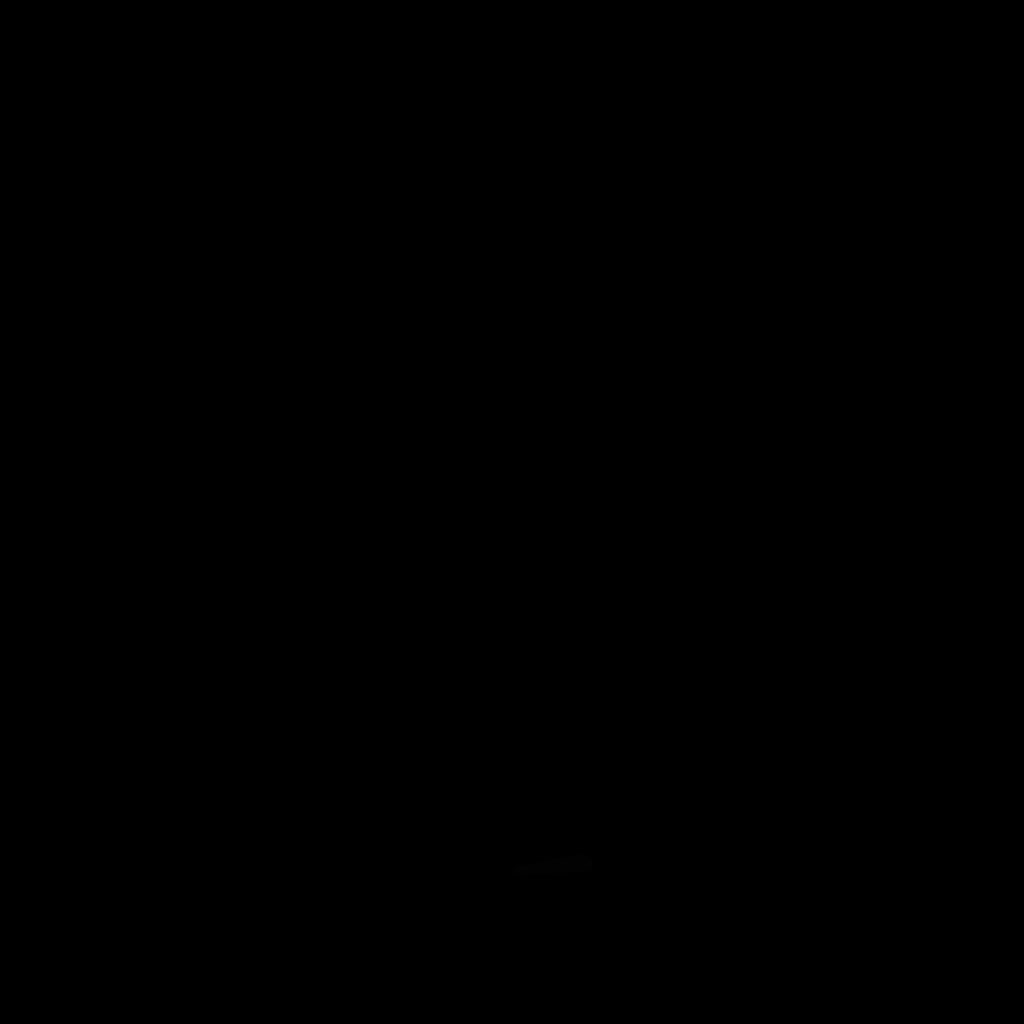

Supplement: Supplemental Information 1 [file peerj-cs-10-2097-s001.zip › IIT-AFF VL/masks/02_00000120.png]

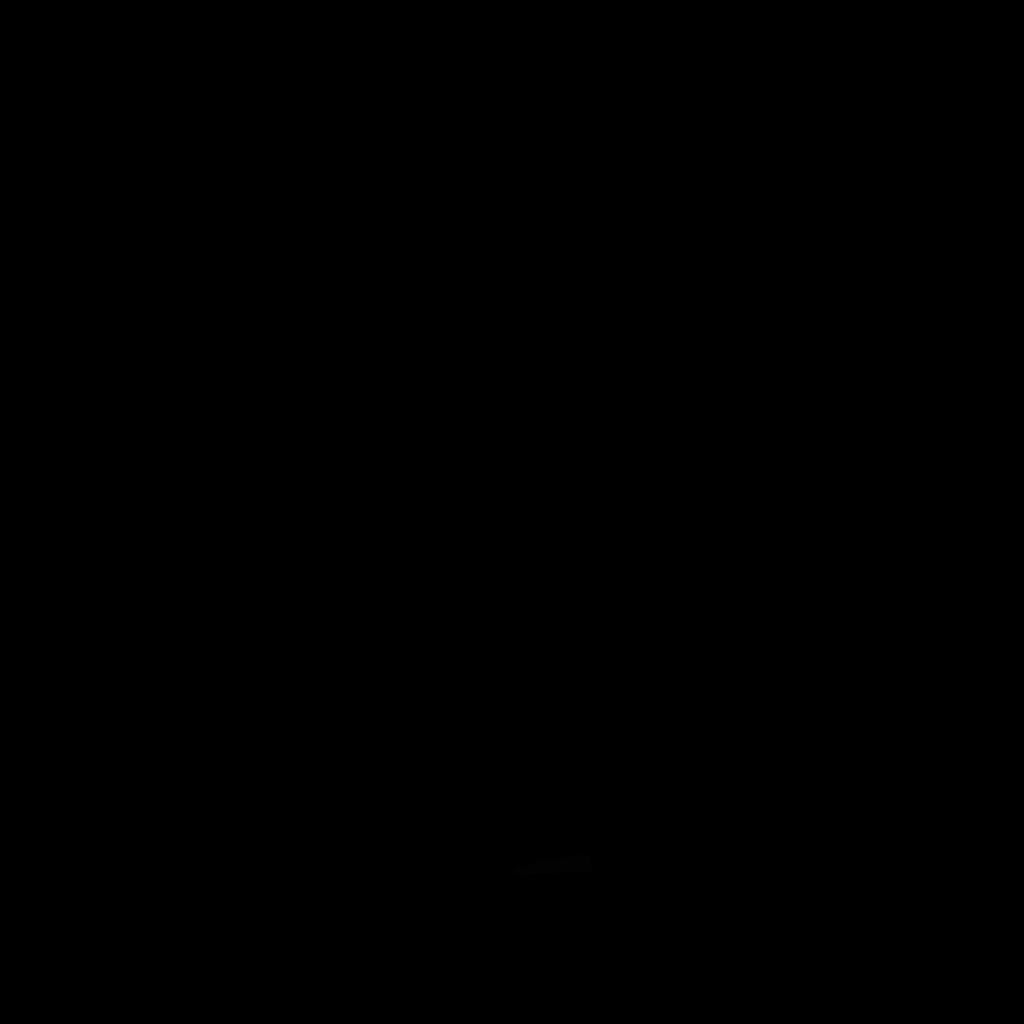

Supplement: Supplemental Information 1 [file peerj-cs-10-2097-s001.zip › IIT-AFF VL/masks/02_00000124.png]

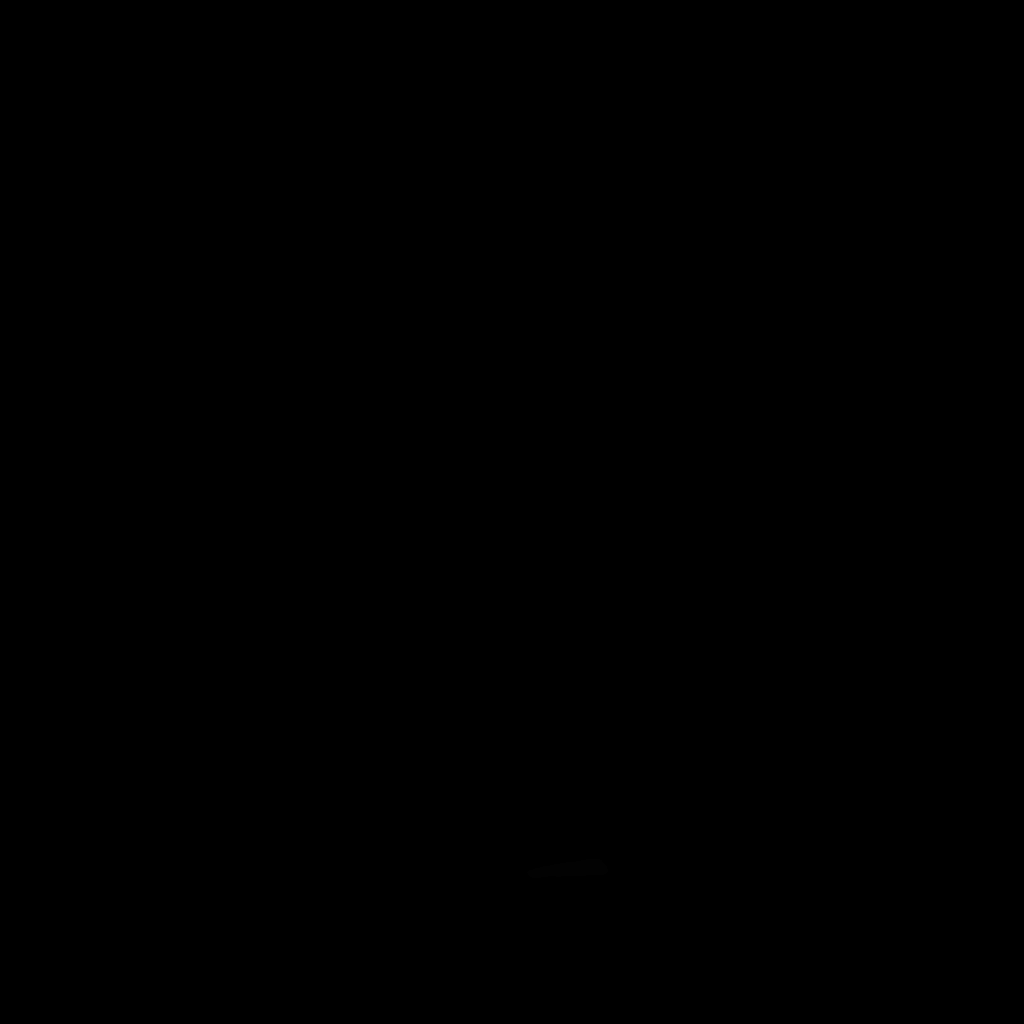

Supplement: Supplemental Information 1 [file peerj-cs-10-2097-s001.zip › IIT-AFF VL/masks/02_00000128.png]

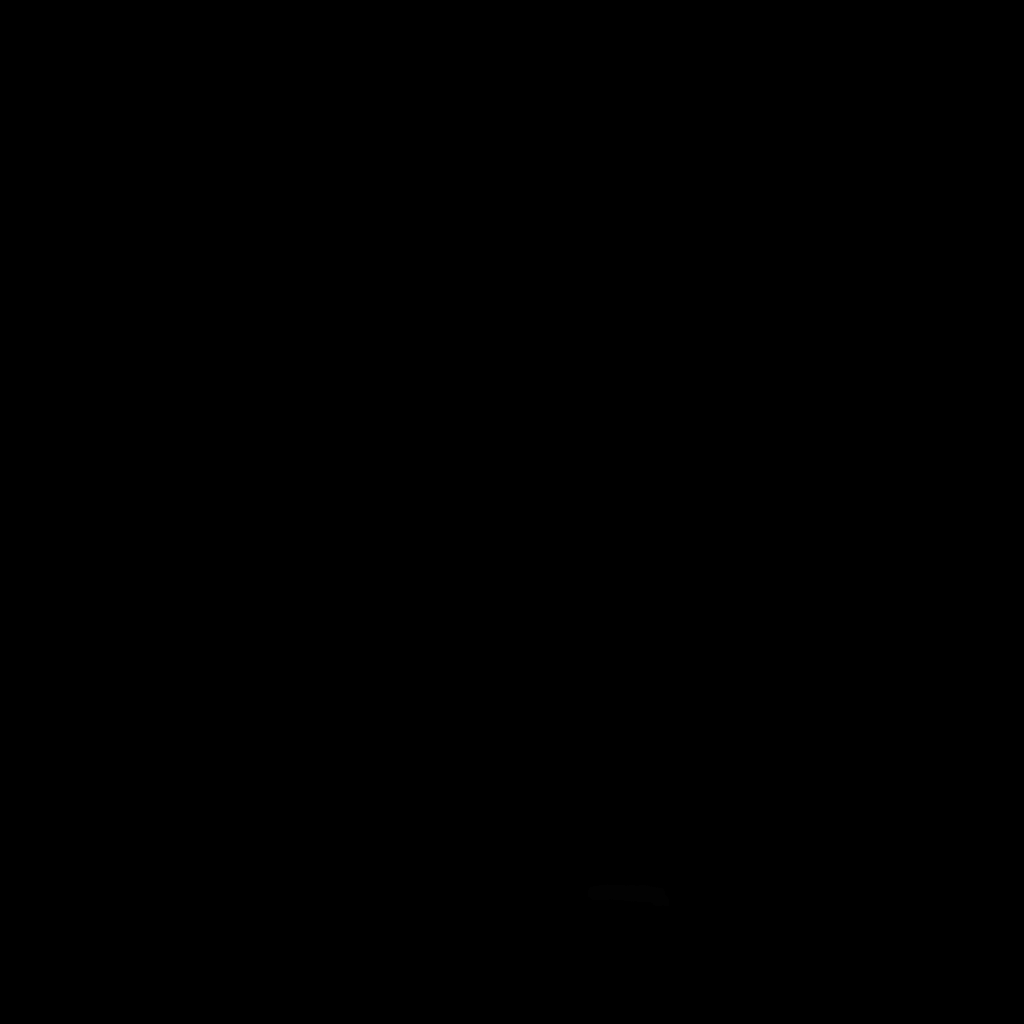

Supplement: Supplemental Information 1 [file peerj-cs-10-2097-s001.zip › IIT-AFF VL/masks/02_00000301.png]

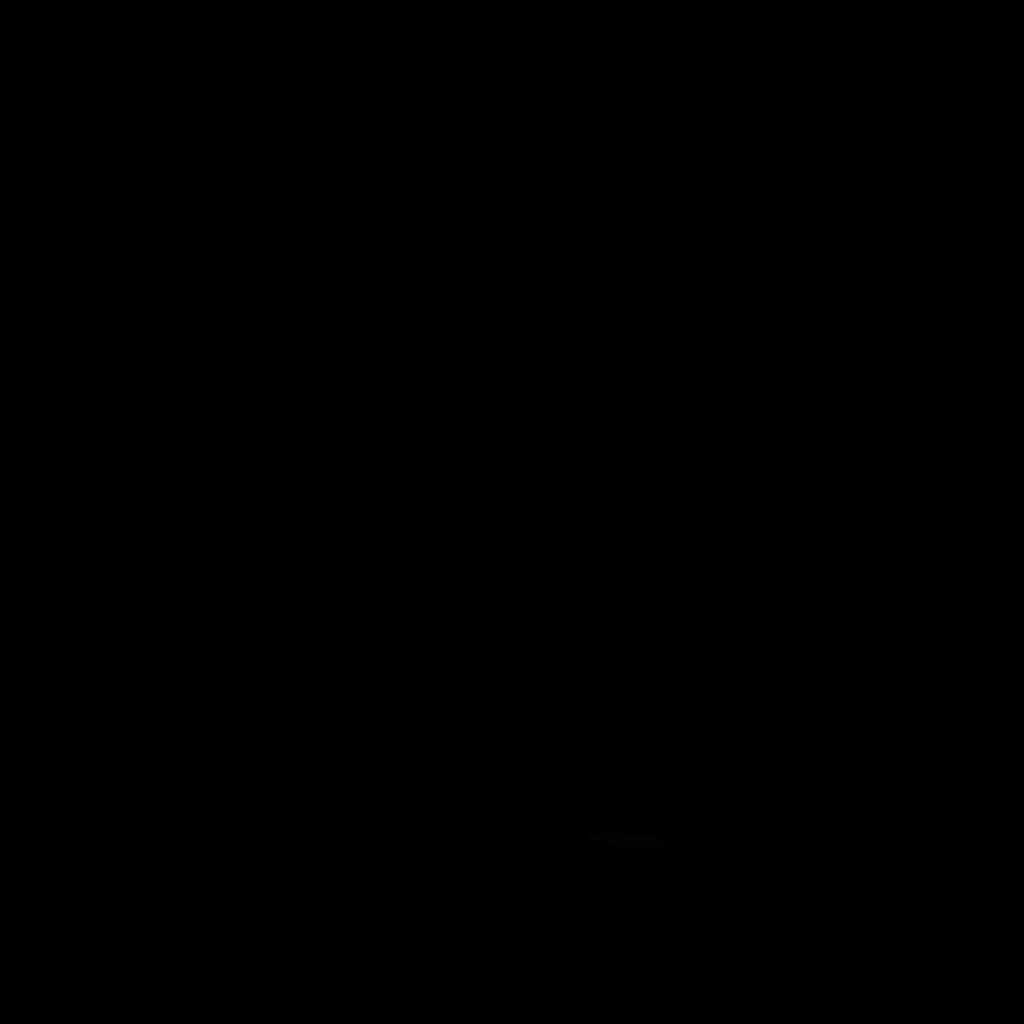

Supplement: Supplemental Information 1 [file peerj-cs-10-2097-s001.zip › IIT-AFF VL/masks/02_00000305.png]

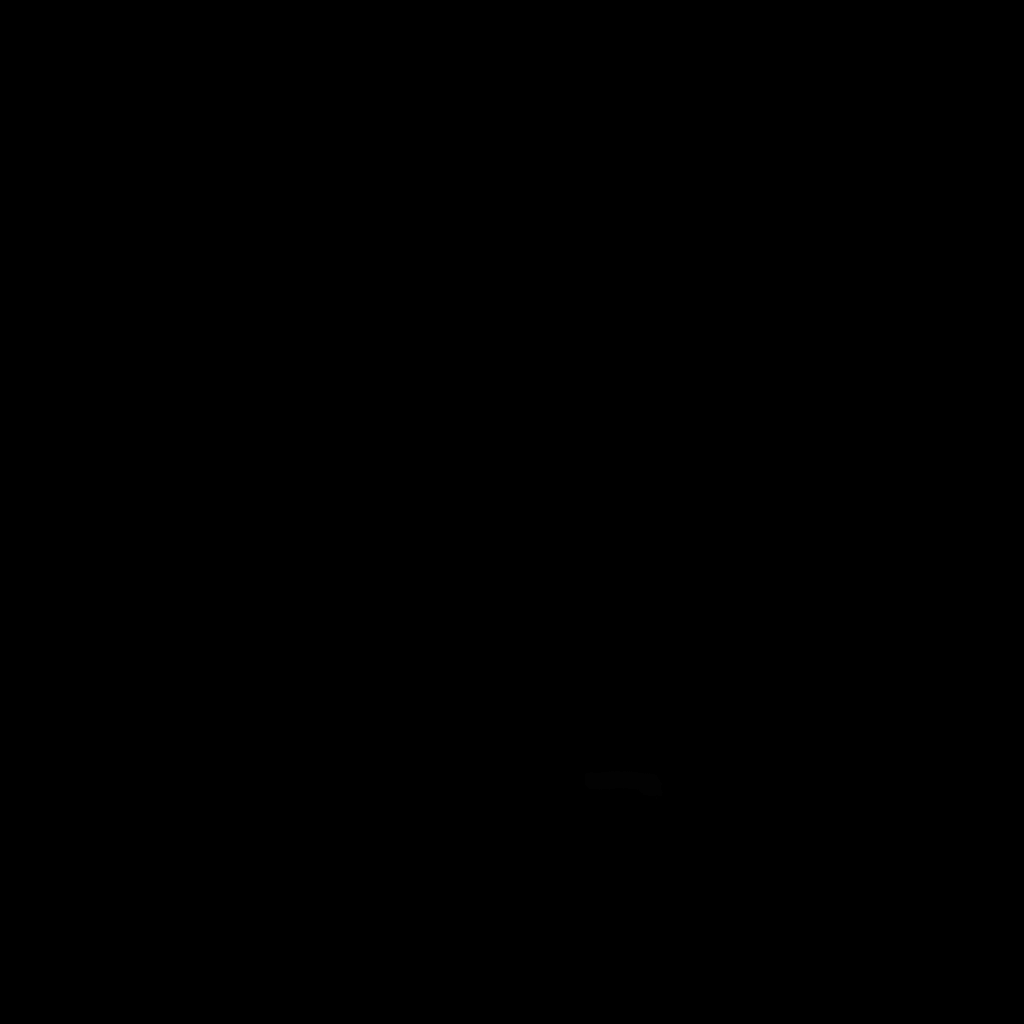

Supplement: Supplemental Information 1 [file peerj-cs-10-2097-s001.zip › IIT-AFF VL/masks/02_00000309.png]

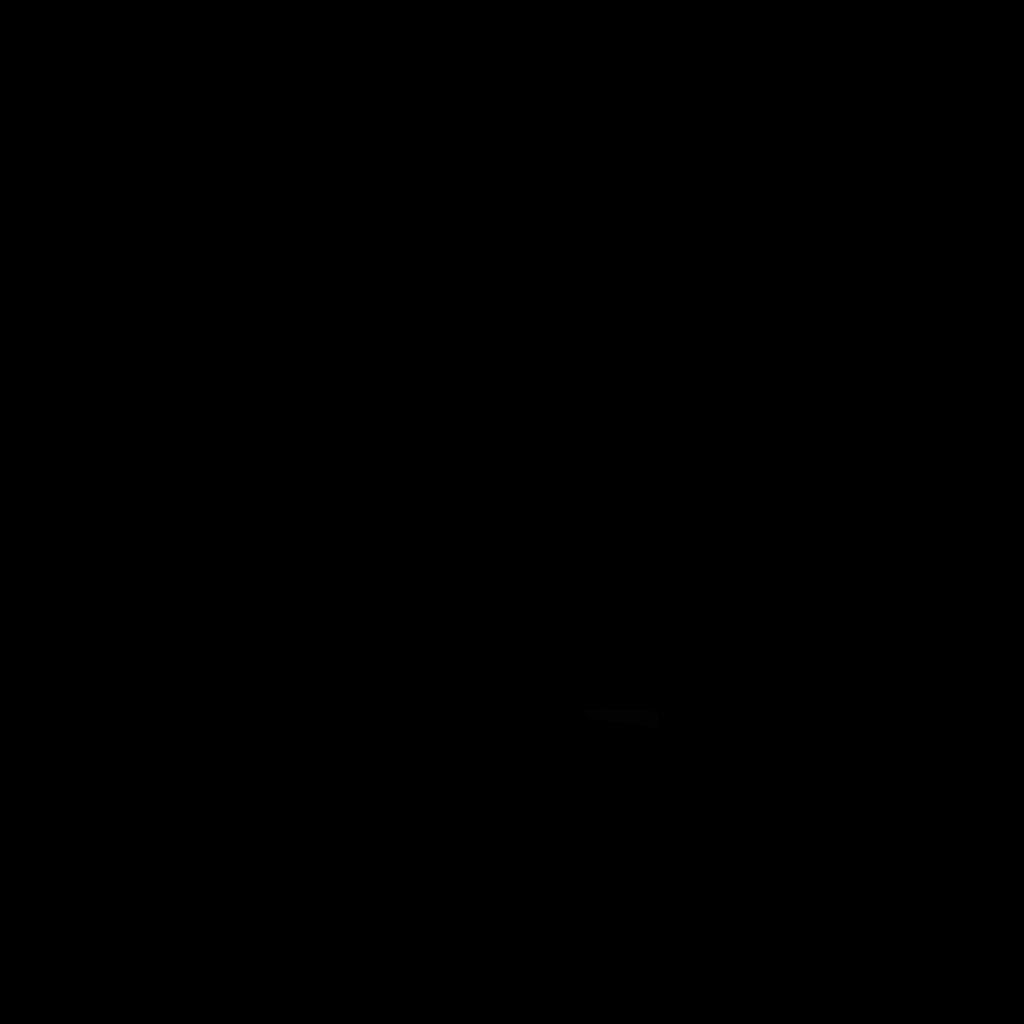

Supplement: Supplemental Information 1 [file peerj-cs-10-2097-s001.zip › IIT-AFF VL/masks/02_00000313.png]

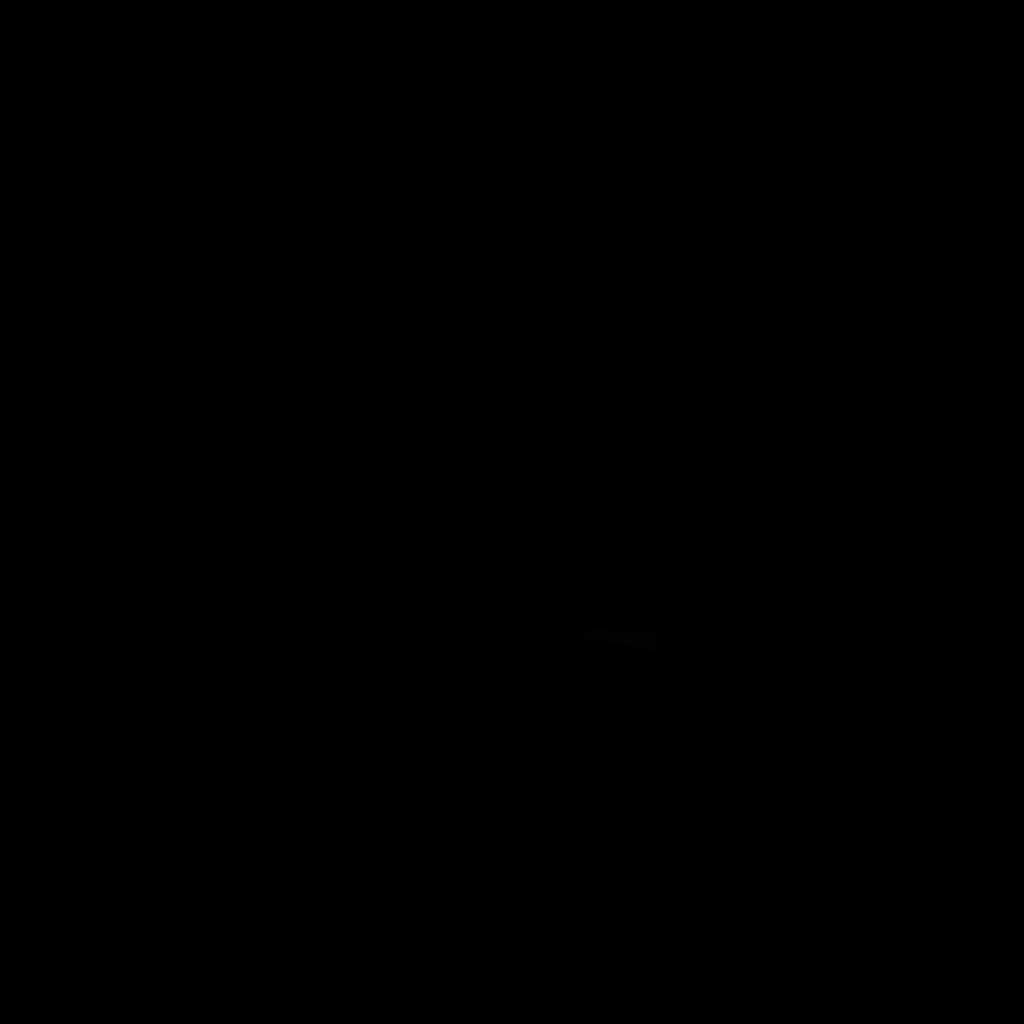

Supplement: Supplemental Information 1 [file peerj-cs-10-2097-s001.zip › IIT-AFF VL/masks/02_00000317.png]

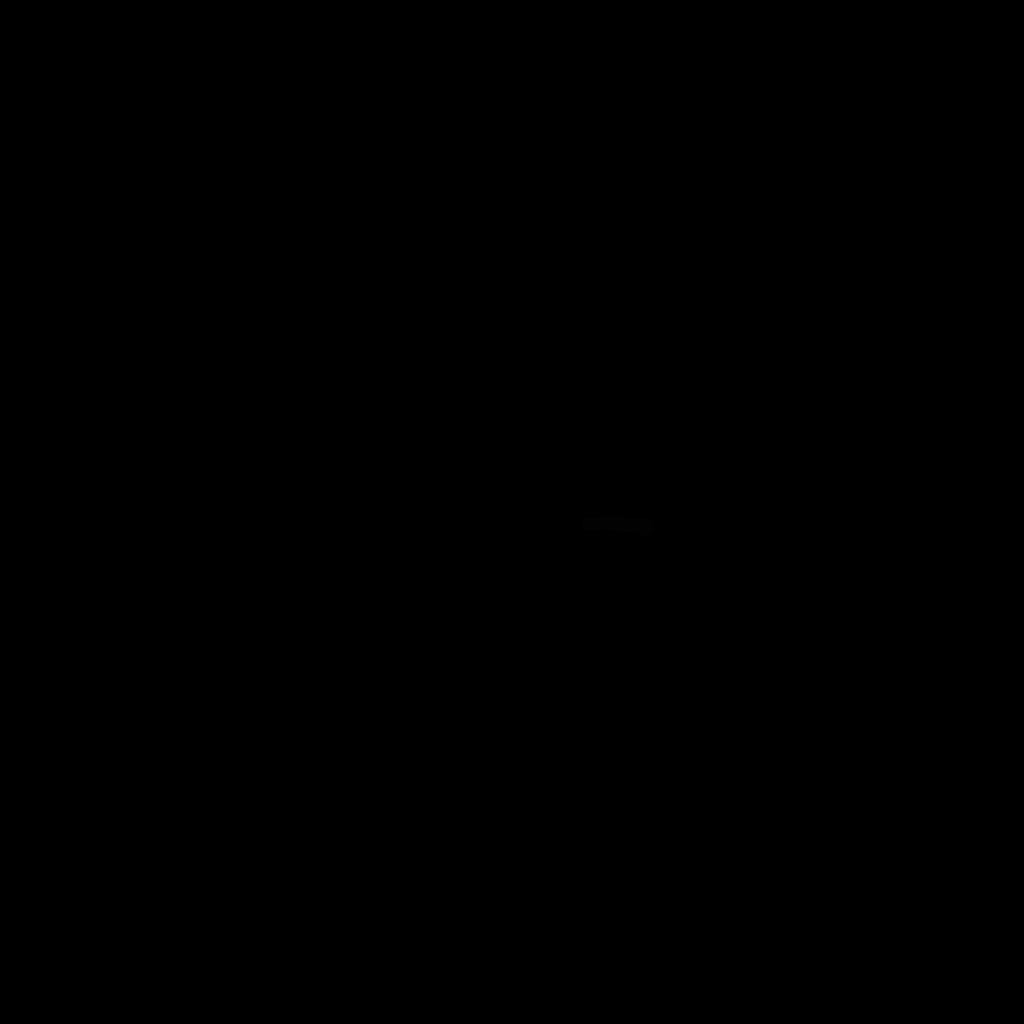

Supplement: Supplemental Information 1 [file peerj-cs-10-2097-s001.zip › IIT-AFF VL/masks/02_00000321.png]

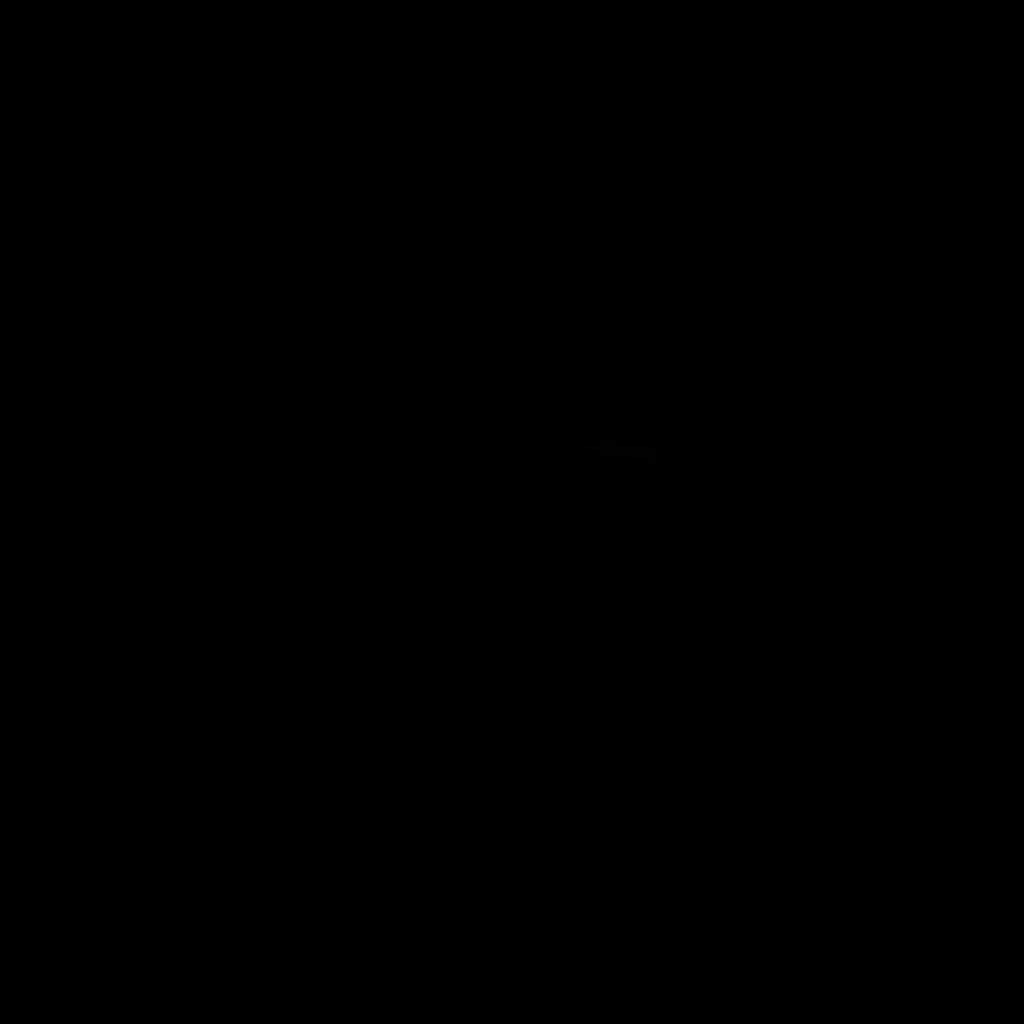

Supplement: Supplemental Information 1 [file peerj-cs-10-2097-s001.zip › IIT-AFF VL/masks/02_00000325.png]

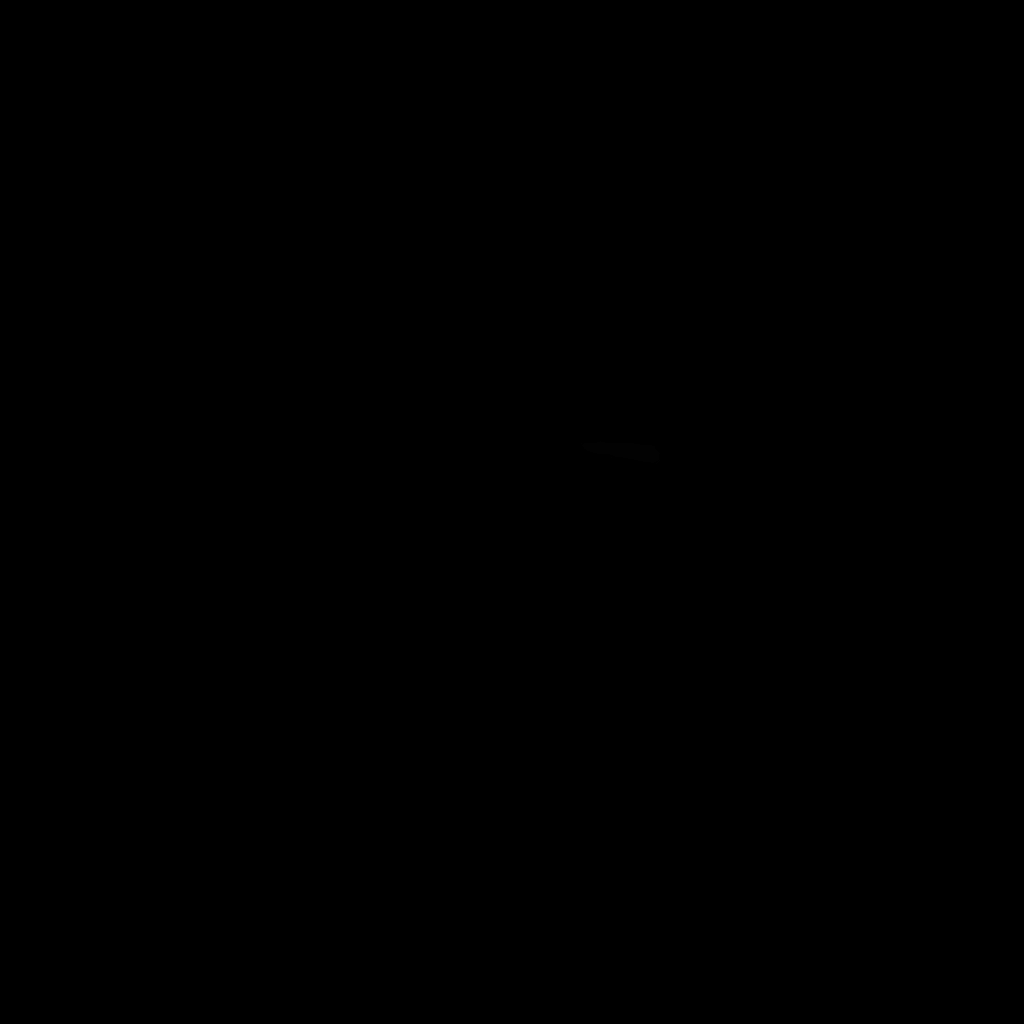

Supplement: Supplemental Information 1 [file peerj-cs-10-2097-s001.zip › IIT-AFF VL/masks/02_00000330.png]

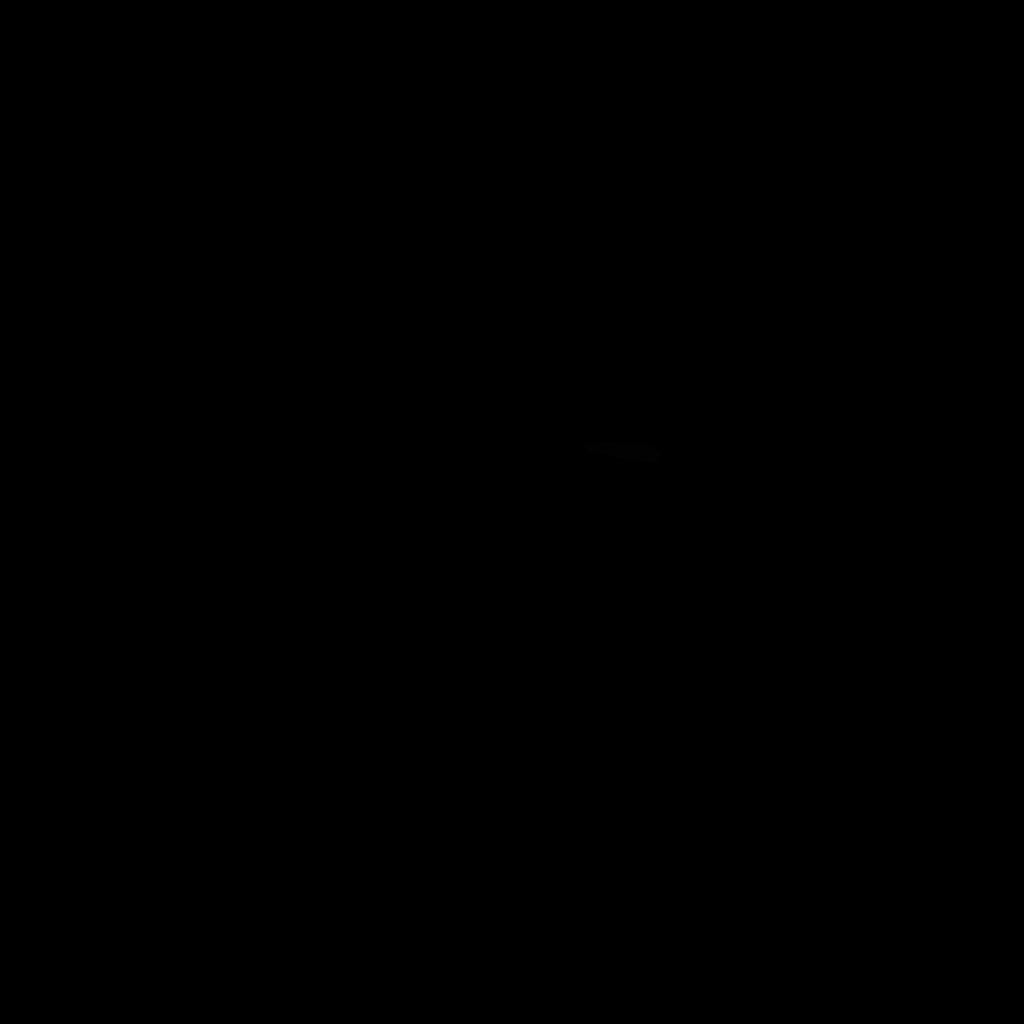

Supplement: Supplemental Information 1 [file peerj-cs-10-2097-s001.zip › IIT-AFF VL/masks/02_00000334.png]

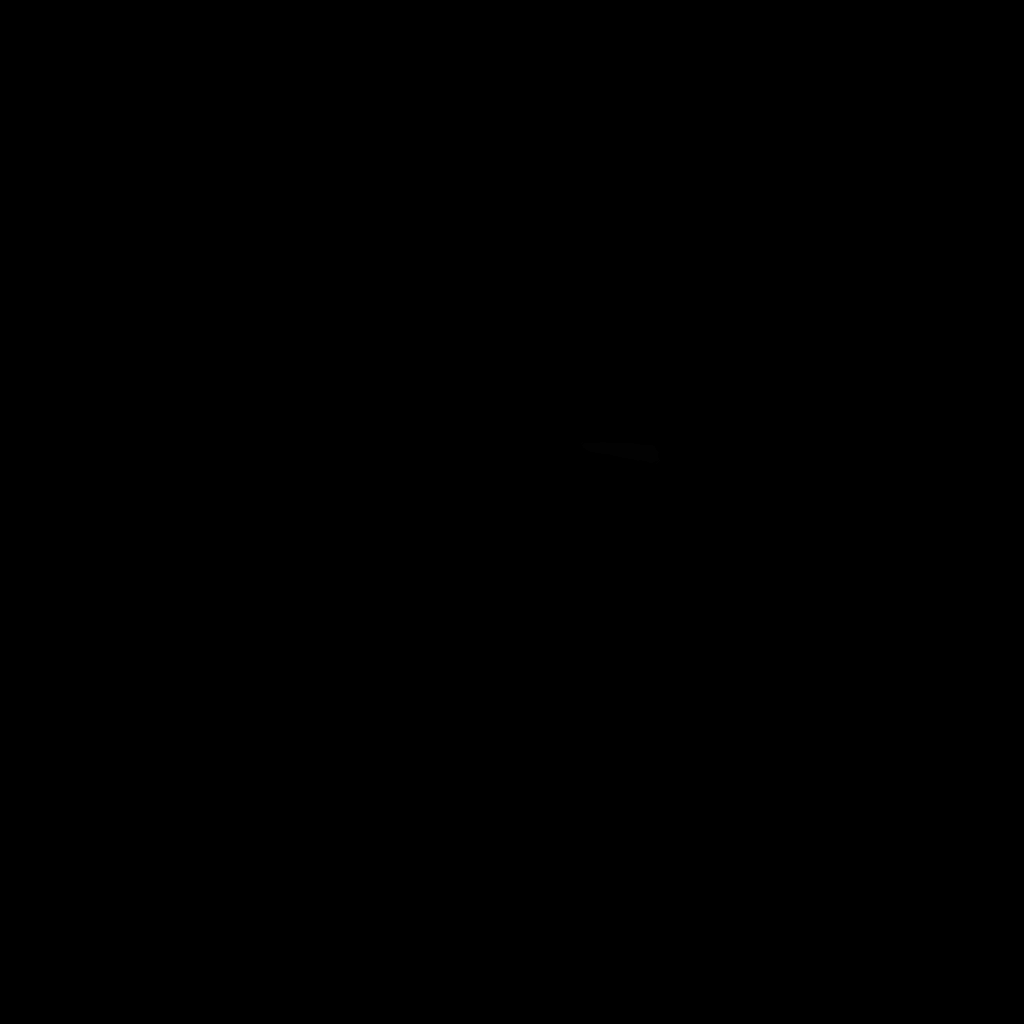

Supplement: Supplemental Information 1 [file peerj-cs-10-2097-s001.zip › IIT-AFF VL/masks/02_00000338.png]

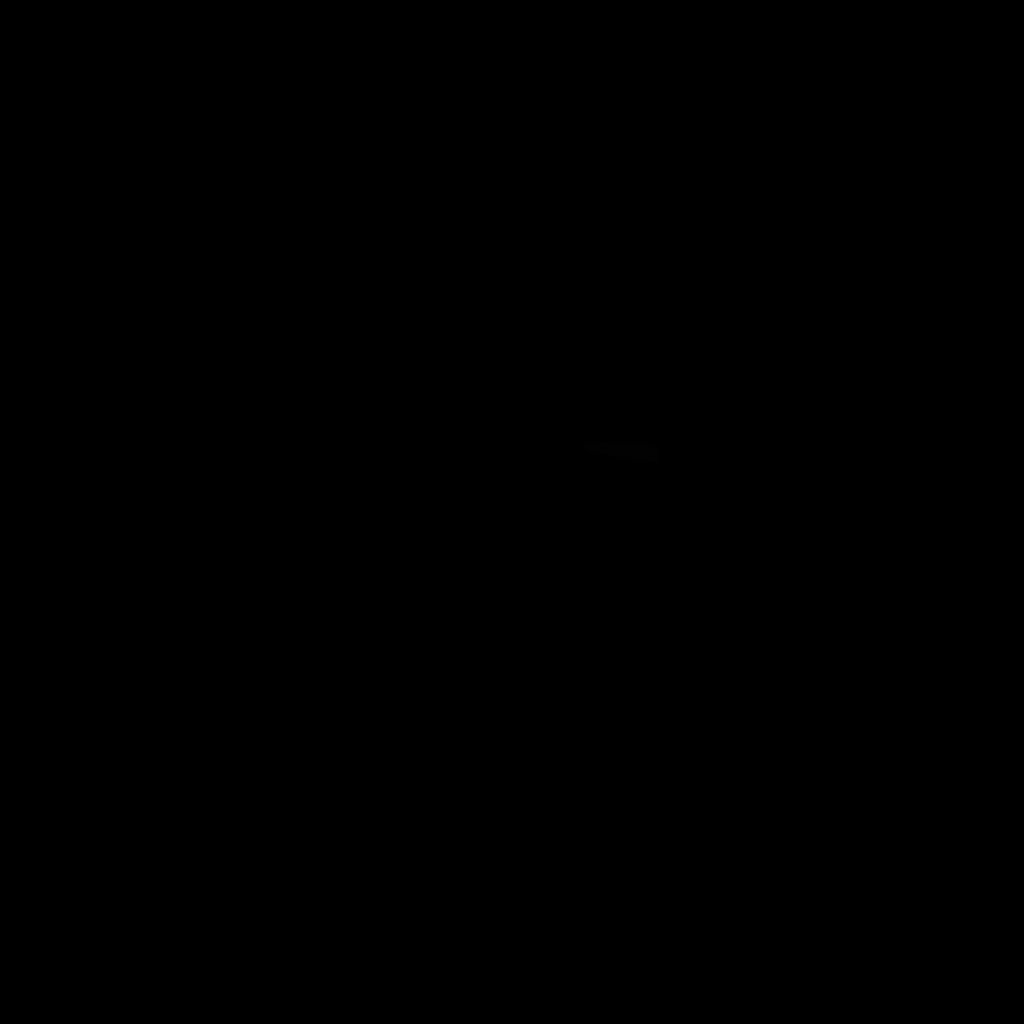

Supplement: Supplemental Information 1 [file peerj-cs-10-2097-s001.zip › IIT-AFF VL/masks/02_00000343.png]

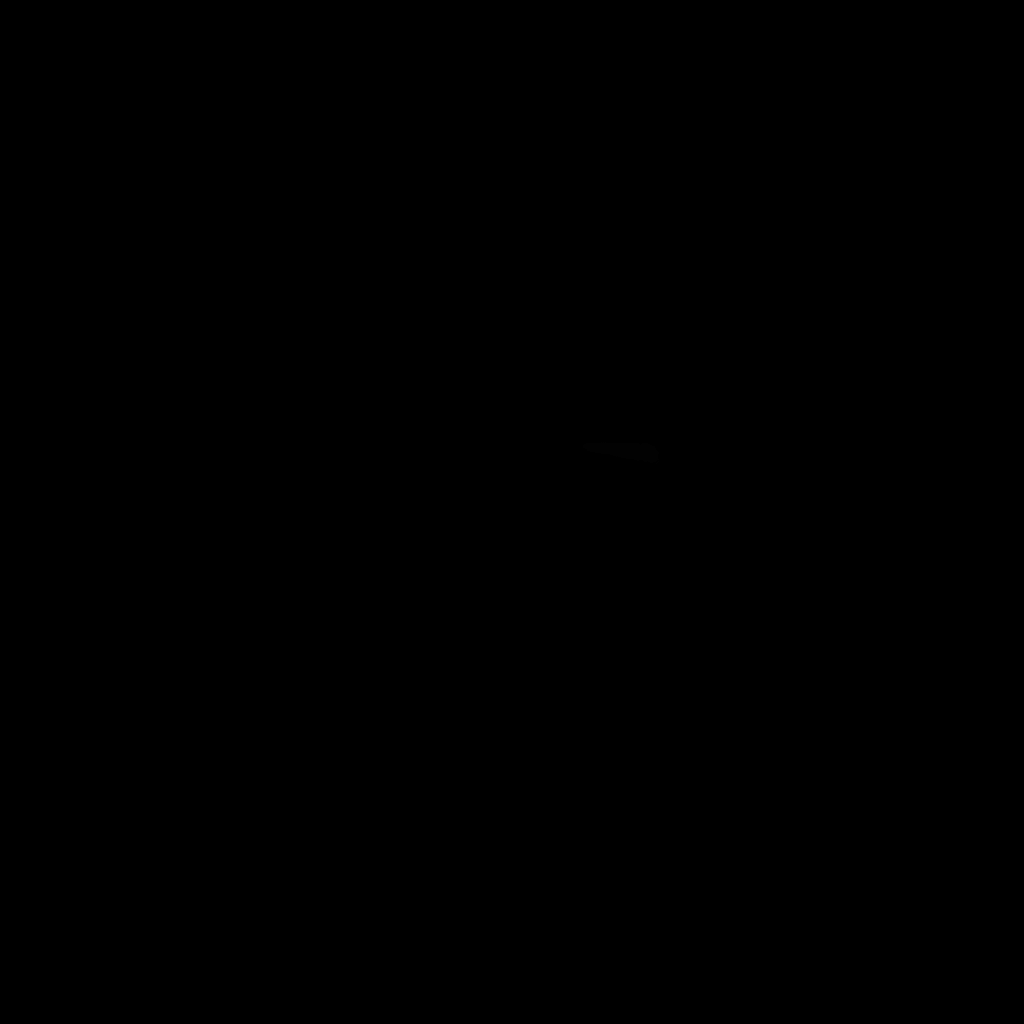

Supplement: Supplemental Information 1 [file peerj-cs-10-2097-s001.zip › IIT-AFF VL/masks/02_00000347.png]

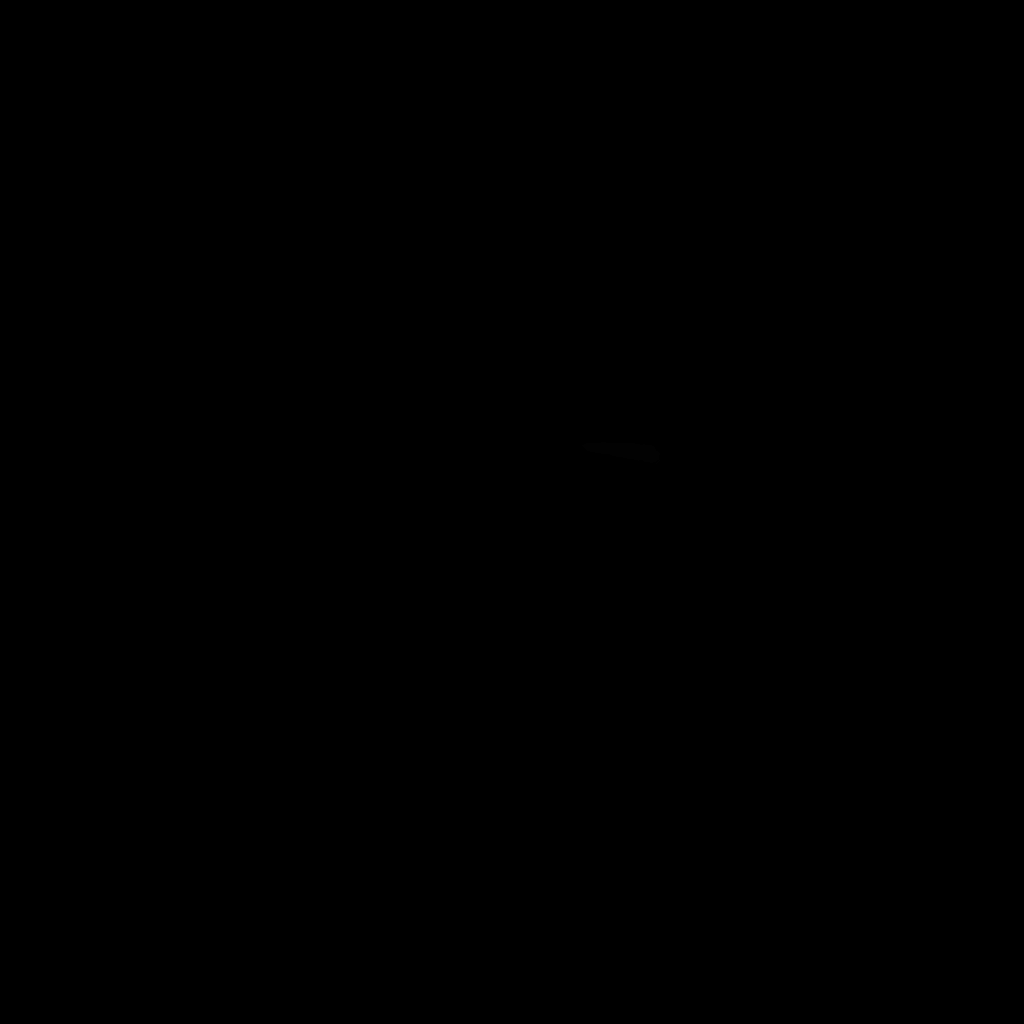

Supplement: Supplemental Information 1 [file peerj-cs-10-2097-s001.zip › IIT-AFF VL/masks/02_00000351.png]

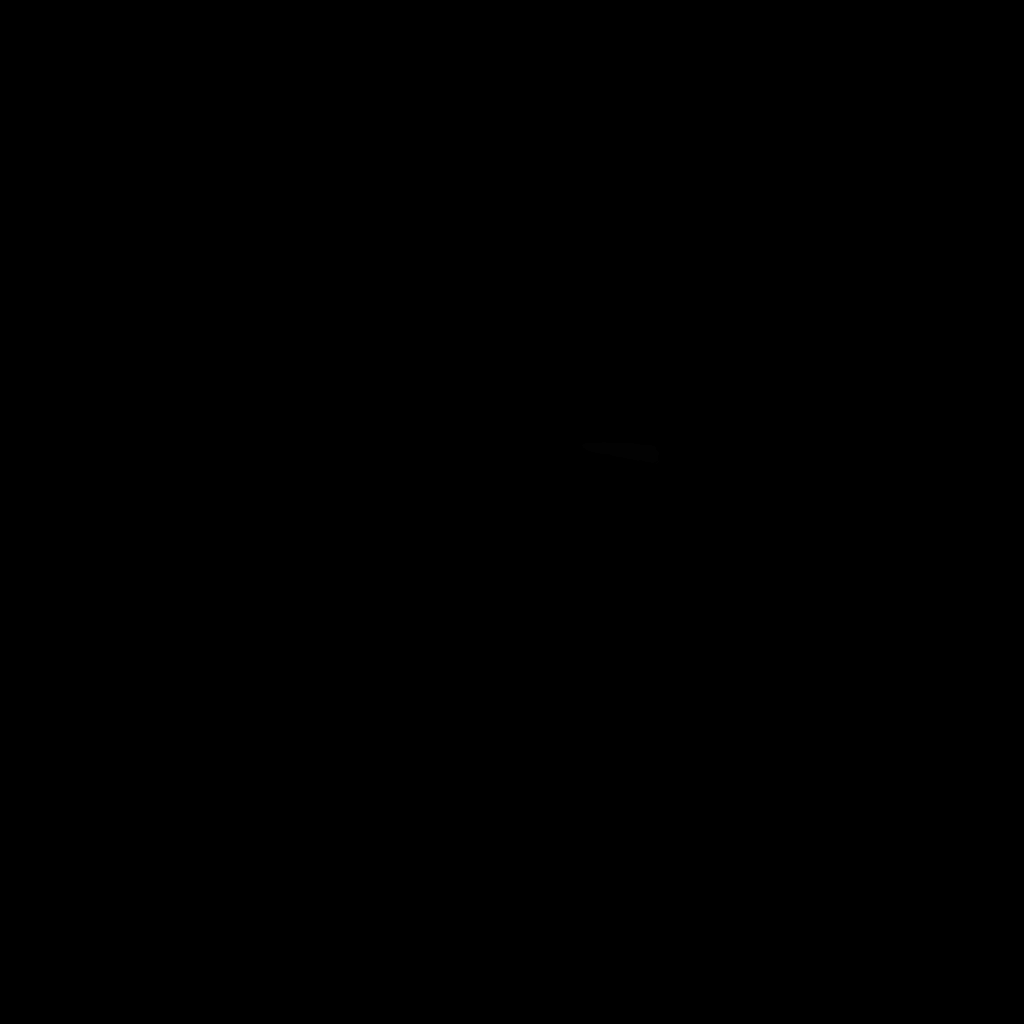

Supplement: Supplemental Information 1 [file peerj-cs-10-2097-s001.zip › IIT-AFF VL/masks/02_00000356.png]

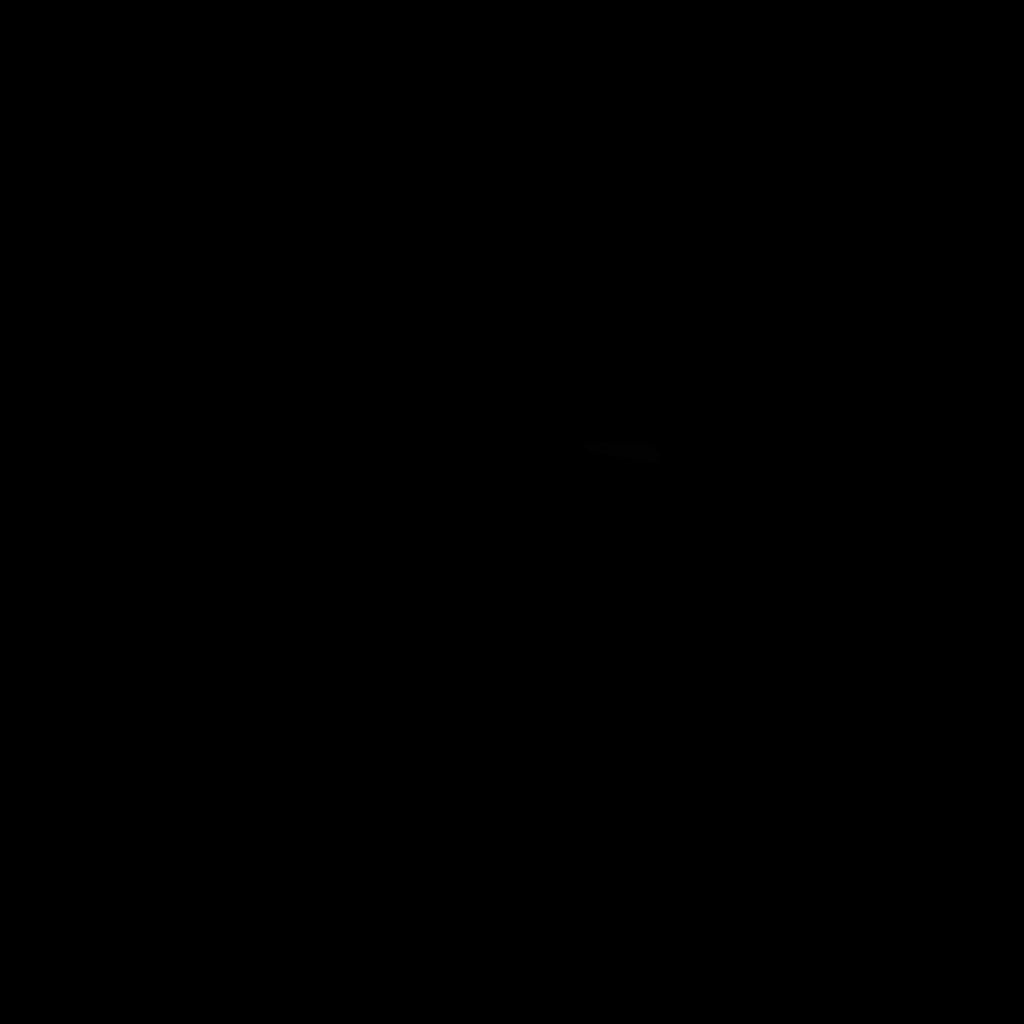

Supplement: Supplemental Information 1 [file peerj-cs-10-2097-s001.zip › IIT-AFF VL/masks/02_00000360.png]

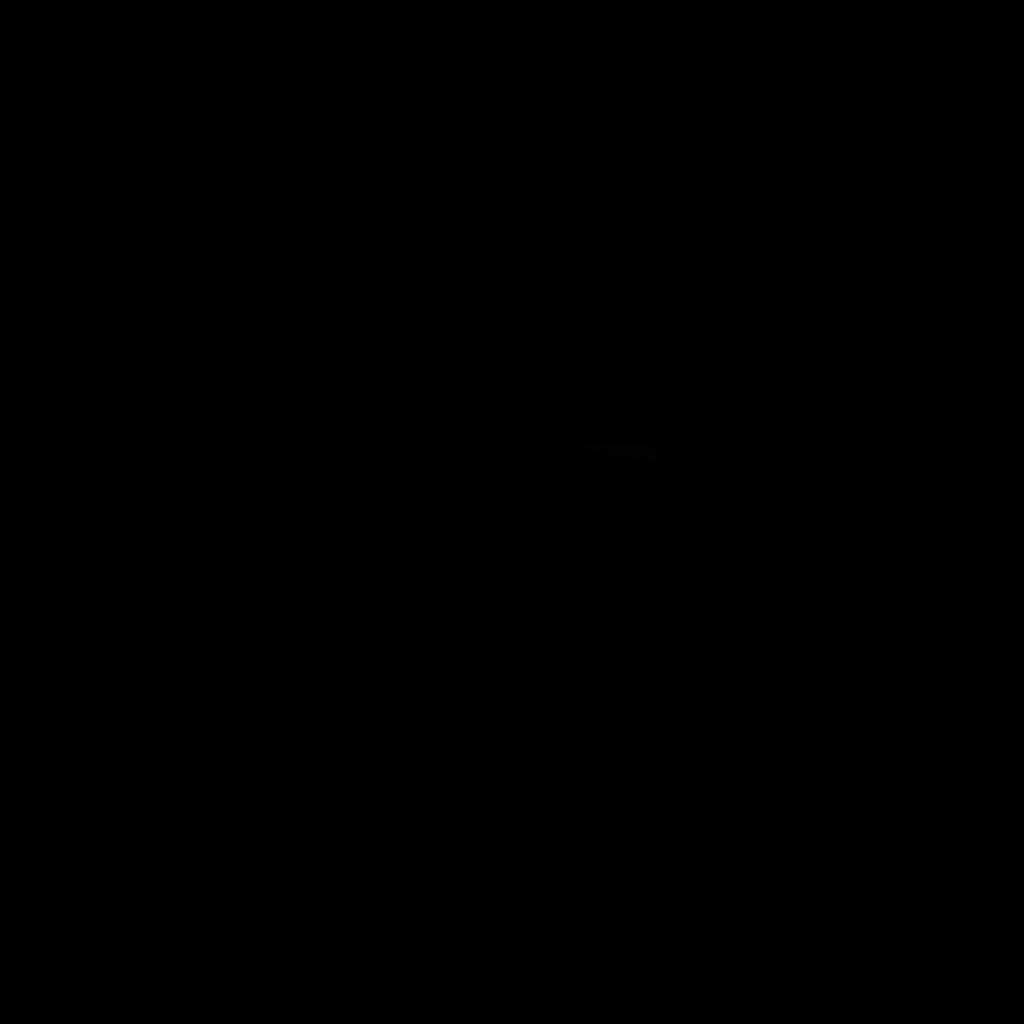

Supplement: Supplemental Information 1 [file peerj-cs-10-2097-s001.zip › IIT-AFF VL/masks/02_00000364.png]

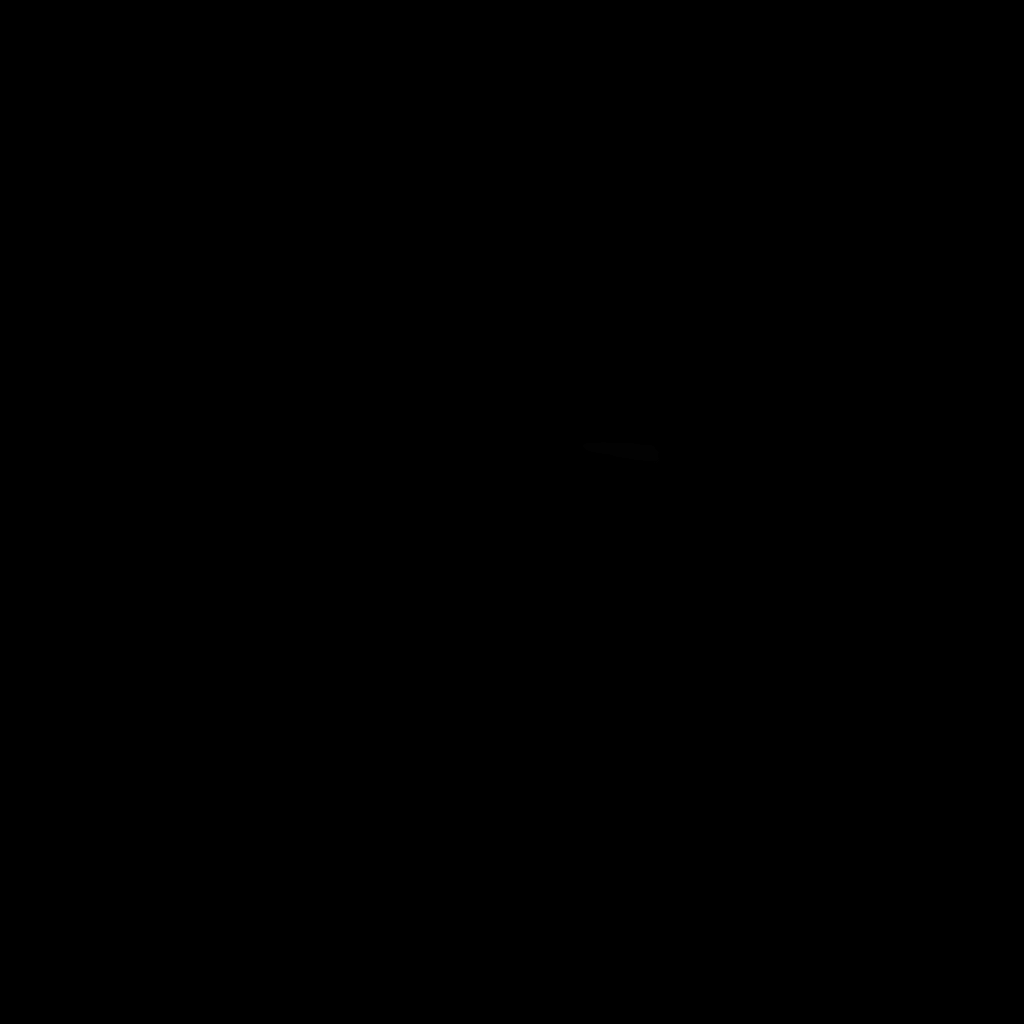

Supplement: Supplemental Information 1 [file peerj-cs-10-2097-s001.zip › IIT-AFF VL/masks/03_00000030.png]

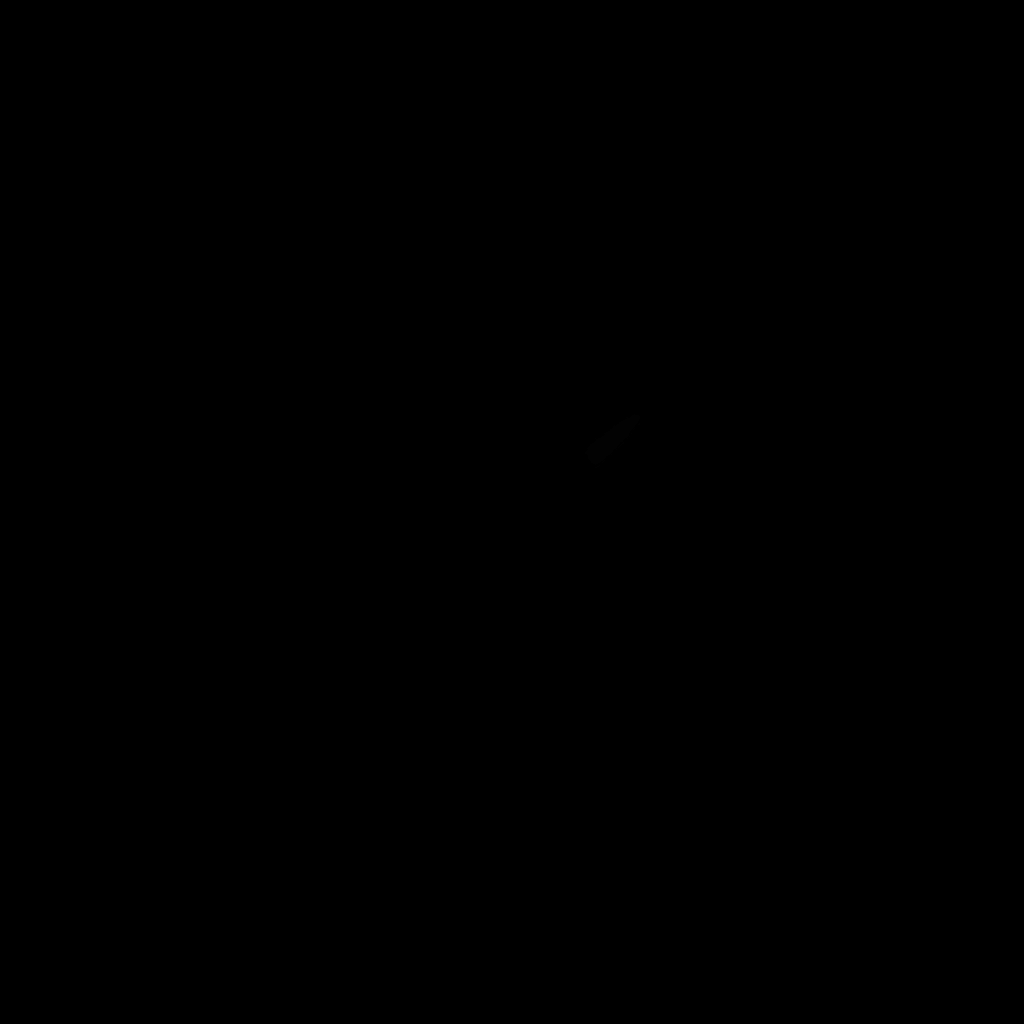

Supplement: Supplemental Information 1 [file peerj-cs-10-2097-s001.zip › IIT-AFF VL/masks/03_00000054.png]

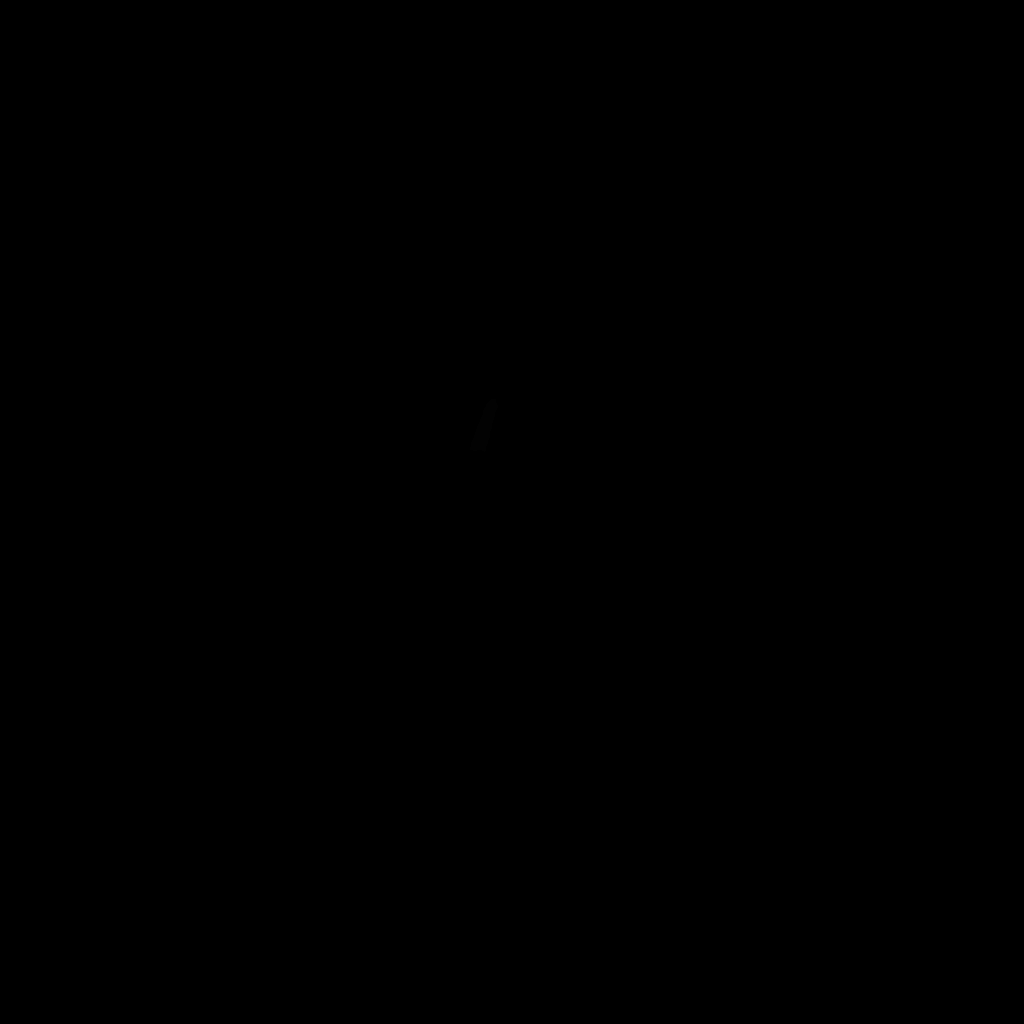

Supplement: Supplemental Information 1 [file peerj-cs-10-2097-s001.zip › IIT-AFF VL/masks/03_00000082.png]

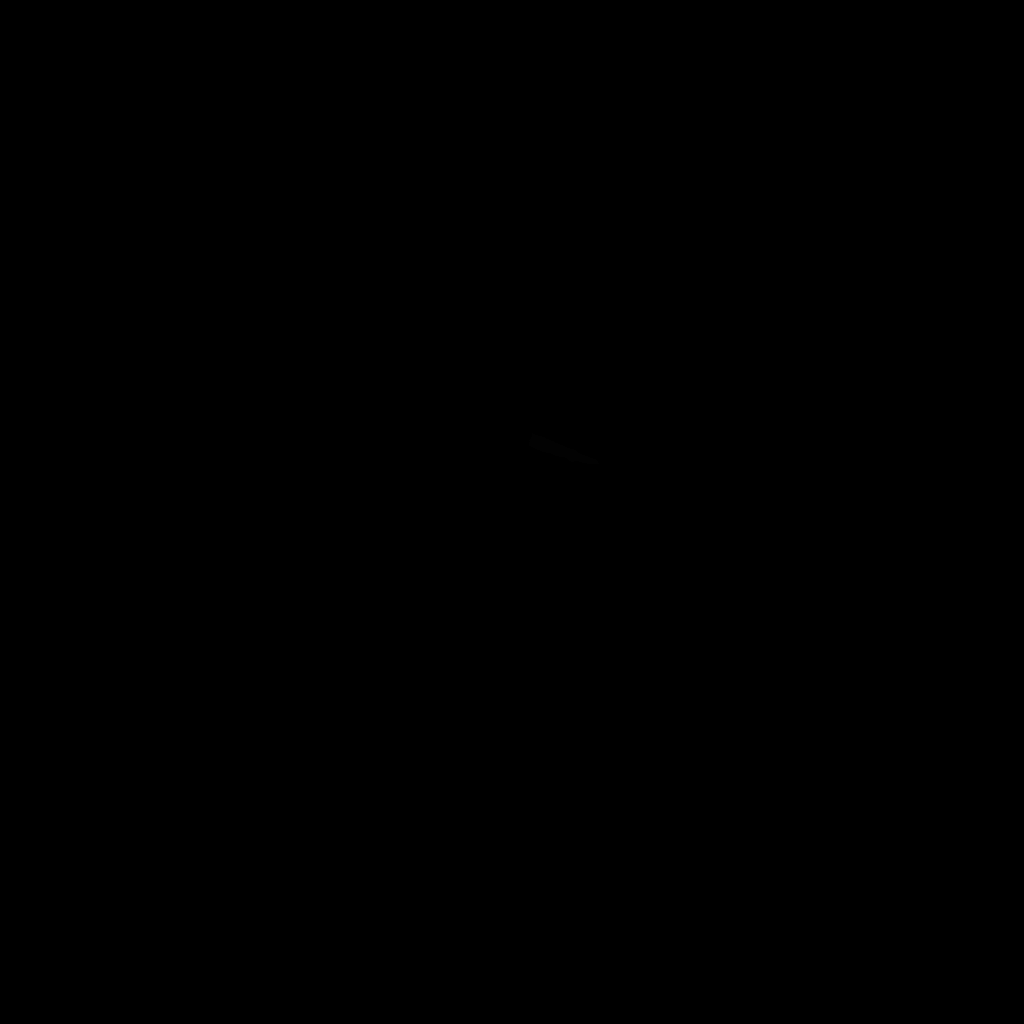

Supplement: Supplemental Information 1 [file peerj-cs-10-2097-s001.zip › IIT-AFF VL/masks/03_00000119.png]

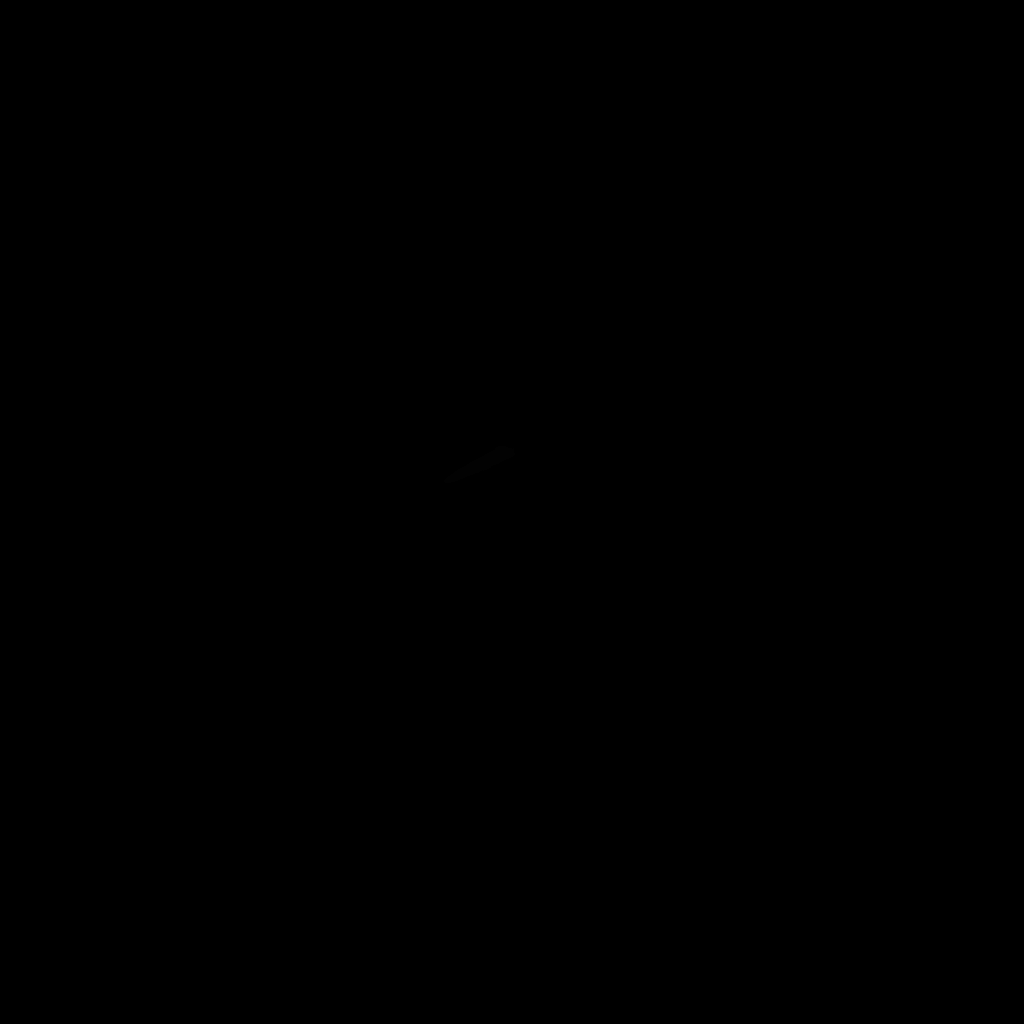

Supplement: Supplemental Information 1 [file peerj-cs-10-2097-s001.zip › IIT-AFF VL/masks/03_00000143.png]

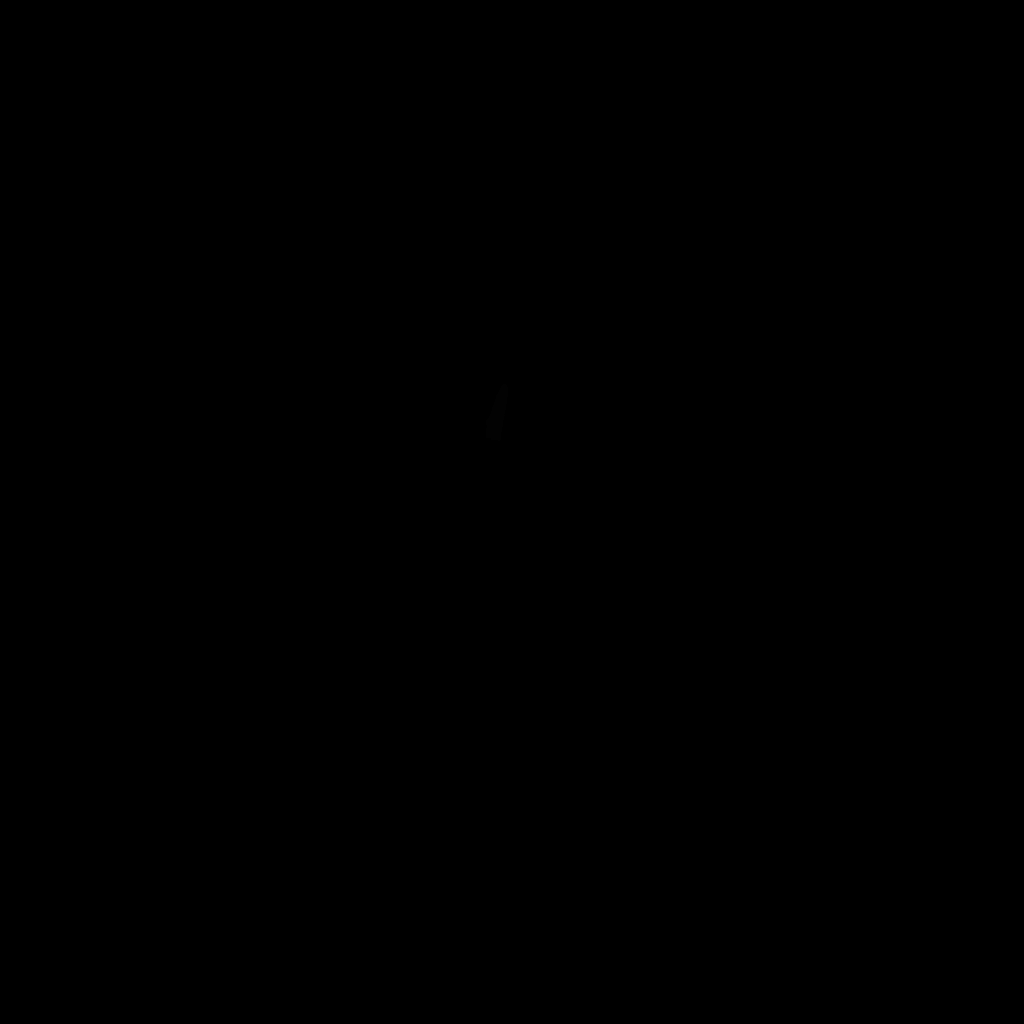

Supplement: Supplemental Information 1 [file peerj-cs-10-2097-s001.zip › IIT-AFF VL/masks/03_00000171.png]
